# Molecular and topographic mapping of antipsychotic effects: a meta-analysis of postsynaptic density proteins in animal models with translational implications

**DOI:** 10.1038/s41380-025-03351-z

**Published:** 2025-11-19

**Authors:** Giuseppe De Simone, Michele De Prisco, Vincenzo Oliva, Licia Vellucci, Mariateresa Ciccarelli, Benedetta Mazza, Giovanna Fico, Michele Fornaro, Felice Iasevoli, Eduard Vieta, Andrea de Bartolomeis

**Affiliations:** 1 Section of Psychiatry. Laboratory of Molecular and Translational Psychiatry. Unit of Treatment-Resistant Psychiatric Disorders. Department of Neuroscience, Reproductive Sciences and Dentistry, University of Naples “Federico II”, School of Medicine, via Pansini 5, 80131, Naples, Italy; 2 Bipolar and Depressive Disorders Unit, Hospìtal Clinic de Barcelona, c. Villarroel, 170, 08036 Barcelona, Spain; 3 Departament de Medicina, Facultat de Medicina i Ciències de la Salut, Institut de Neurociències, Universitat de Barcelona (UB), c. Casanova, 143, 08036 Barcelona, Spain; 4 Institut d’Investigacions Biomèdiques August Pi i Sunyer (IDIBAPS), c. Villarroel, 170, 08036 Barcelona, Spain; 5 Biomedical Research Networking Centre Consortium on Mental Health (CIBERSAM), Instituto de Salud Carlos III, Madrid, Spain

**Keywords:** Schizophrenia, Molecular biology

## Abstract

**Background:**

While antipsychotics primarily target dopamine D_2_ receptor, the putative synaptic mechanisms underlying their therapeutic effects remain unclear. Postsynaptic density (PSD) at glutamatergic synapses represents a dynamic protein network involved in synaptic plasticity and neurotransmission, whose dysfunction has been implicated in the pathophysiology of schizophrenia and bipolar disorder. This study aims to explore, for the first time, with a quantitative meta-analytical approach, how antipsychotic treatments affect PSD molecules across various brain regions in preclinical settings, which may overcome the heterogeneity of human studies.

**Methods:**

We systematically reviewed and meta-analyzed peer-reviewed preclinical studies reporting quantitative effects of typical and atypical antipsychotics on PSD-related outcomes. Statistical analyses used random-effects models, with heterogeneity assessed via τ², I², and Cochran’s Q. Separate meta-analyses were conducted for PSD protein type, brain region, animal type, and treatment paradigms. Meta-regressions assessed the impact of cofounding variables.

**Results:**

We included 81 studies (n = 2542; rodents=2510; monkeys=26), resulting in 226 meta-analyses. Both typical and atypical antipsychotics modulated PSD molecules albeit with different degree due to the drug receptor profile, dose, specific protein detected, brain region, and administration regimen. Haloperidol, amisulpride, and aripiprazole significantly increased PSD protein expression in the striatum. Acute olanzapine increased PSD molecules levels in striatal regions, reducing Arc expression in frontal cortex (SMD [95%CIs]=-2.15 [-2.7;-1.61], p < 0.0001); chronic treatment had opposite effects. Clozapine increased Homer1a levels in dorsal (1.13 [0.41;1.86], *p* = 0.002) and ventral striatum (1.48 [0.72;2.24], *p* = 0.0001), and cingulate cortex (2.12 [1.12;3.12], p < 0.0001), as well as increased NMDAR subunits levels in frontal cortex.

## Introduction

Antipsychotics represent the cornerstone of pharmacological treatment for several psychiatric conditions, including schizophrenia, bipolar disorder, and psychotic depression [1–5]. Beyond these primary indications, antipsychotics serve as an augmentation strategy in treatment-resistant major depression and are used to manage agitation and psychotic symptoms in other acute and chronic behavioral disorders, including Alzheimer’s disease, autism spectrum disorder, and delirium [6–9]. Given the high incidence of these conditions, the global trend of antipsychotic usage has been increasing over time [10].

Except for a few recently developed compounds, such as xanomeline and others currently under clinical trial [11–14], all antipsychotics share the common mechanism of dopamine D_2_ receptor (D_2_R) occupancy, which may include either antagonism or partial agonism [15, 16]. Despite this common feature, several other neurotransmitter pathways, such as serotonin, glutamate, GABA, and acetylcholine, have been implicated in the antipsychotic mechanism of action, explaining a part of their clinical response and side effects [12, 17]. However, different neurotransmitter pathways usually converge on the same neuronal populations, generating synergic intracellular signaling cascades that are both timely and spatially integrated at the postsynaptic site [18, 19].

The integration of multiple signaling pathways occurs in the “quantal” structures of the brain architecture: the synapse and dendritic spine. In this regard, the postsynaptic density (PSD), a disc-shaped structure of approximately 100 nm in diameter, localized at glutamatergic synapses and identifiable via electron microscopy, has gained increasing interest in the pathophysiology of psychiatric disorders and their treatment [20]. The PSD serves as an integrative hub where multiple neurotransmitter pathways converge, with a relevant role especially in dopamine-glutamate interactions, which are critically involved in the pathophysiology and treatment of psychotic disorders [21–23].

The relevance of PSD in the context of antipsychotic treatment is warranted for multiple reasons. First, the PSD has been directly implicated in the pathophysiology and genetics of schizophrenia and bipolar disorder by genome-wide association studies [24–26], postmortem studies [27], preclinical paradigms [28], and clinical reports [29, 30]. Second, both schizophrenia and bipolar disorder have been increasingly conceptualized as disorders of synaptic plasticity and brain connectivity, thus drawing attention to the PSD, a structure critically involved in organizing synaptic microdomains that support large-scale neural networks [31–35]. Finally, the delayed onset of clinical improvements associated with antipsychotic drugs, typically within 6 weeks, along with their ability to affect neuroplasticity, suggests that their effects on synaptic remodeling may play a significant role in their long-term action [36–38]. In this regard, several studies have shown that the administration of antipsychotics may alter the gene expression of PSD molecules [39–43].

The pleiotropic nature of antipsychotics, which influence several neurotransmitter pathways and modulate widespread connectivity across multiple neural systems, often in a disorder-specific manner, may be captured by investigating their impact on PSD molecules [44–47]. Within this framework, and acknowledging that no single receptor, molecular target, or brain region could be considered as the “holy grail” substrate of antipsychotic efficacy, the PSD, with its role in integrating diverse neurochemical pathways, may represent a key point of convergence for their molecular effects [48]. Because of this integrative function, the PSD may offer a preferred perspective for drawing molecular topographical maps of antipsychotic effects, as it lies downstream of multiple neurotransmitter pathways and reflects more stable, time-integrated responses.

To date, no systematic and quantitative study has been designed to characterize how antipsychotic drugs impact the PSD across different brain regions in a preclinical setting. Although individual studies have explored specific effects, a comprehensive overview of their influence is missing. The primary aim of this meta-analysis was to investigate how and to what extent antipsychotic treatment impacts PSD molecules compared with untreated controls. Secondary aims were to examine whether treatment duration, brain region, animal species, receptor profile, and type of PSD protein influenced antipsychotics’ effects. To address these aspects, we stratified the analyses accordingly.

## Methods

A systematic review and meta-analysis was performed according to the Preferred Reporting Items for Systematic Reviews and Meta-Analysis (PRISMA) [49] and the Collaborative Approach to Meta-Analysis and Review of Animal Data from Experimental Studies (CAMARADES) (http://camarades.de/) guidelines. The study protocol was preregistered on the Open Science Framework and is available at this link (OSF: https://osf.io/fq2w4/?view_only=b8c853281d6844db845ad440720b6717). Deviations from the original protocol were reported in Supplementary Information (Appendix 1).

### Search strategy

The PubMed, Embase, Web of Science, and Scopus databases were systematically searched for relevant references from inception to January 8^th^, 2025. The search strategy was based on a combination of antipsychotic drug names and PSD protein names. Detailed search strings for each database are provided in the Supplementary Information (Appendix 1). Further, relevant cross-references, textbooks, and other sources were manually searched to identify any additional references not captured within the initial database.

### Inclusion and exclusion criteria

#### Population

We included original experimental studies conducted on animals (e.g., rats, mice, monkeys), regardless of age. Studies considering animal models of psychosis (e.g., psychomimetic drug administration, genetic models, maternal stress) or dopamine supersensitivity (e.g., chronic haloperidol exposure) were also included.

#### Intervention

Any drug classified as an antipsychotic, administered as monotherapy or augmentation therapy at any dose or regimen (acute or chronic), was considered eligible. A complete list of included antipsychotic drugs is reported in Supplementary Information (Appendix 2).

#### Comparison

Control animals matched the experimental group in all characteristics except for receiving vehicle treatment instead of the antipsychotic.

#### Outcome

Only studies reporting quantitative data on PSD molecules in brain tissue, either mRNA or protein levels, were included. A list of eligible PSD molecules is provided in Supplementary Information (Appendix 2).

#### Exclusion Criteria

We excluded reviews, case reports, case series, commentaries, and letters to the editor. Studies lacking a control group or not reporting quantitative outcome data were also excluded. To minimize confounding effects, studies in which antipsychotics were co-administered with other pharmacological agents (outside augmentation protocols) were excluded. Studies conducted on humans, as well as studies assessing PSD-related outcomes in non-brain tissues, were excluded.

### Data extraction

Title and abstract screening of eligible studies was independently performed by seven investigators (GDS, MDP, VO, LV, MC, BM, GF). Any disagreement was solved through discussion and consensus. In cases of uncertainty, a senior author (AdB) was consulted.

Full-text assessment and data extraction were independently performed by two investigators (GDS, LV). Any disagreement was solved through discussion and consensus among the authors. In cases of uncertainty, a senior author (AdB) was consulted. The following variables were extracted (when applicable): first author, publication year, country, study design (randomized or not), animal model of psychosis or dopamine supersensitivity, animal species (e.g., rodents or monkey), animal type (e.g., Wistar rats, Sprague-Dawley rats, C57BL/6 mice), animal weight, antipsychotic name, antipsychotic class, antipsychotic dose, drug administration route, PSD molecule type, PSD expression type (mRNA or protein), brain region considered, mean and standard deviation (SD) of the PSD component expression level, sample size, mean age or developmental stage, % of females, molecular techniques adopted to measure the outcome of interest (e.g., Western blot, PCR), administration model (acute or chronic), duration of the trial, time from the last drug administration.

Due to the different nomenclature adopted and the evaluation of outcomes in small brain areas embedded within larger, functionally related systems, brain regions were grouped into broader categories (e.g., associative cortex, hippocampus, dorsal and ventral striatum, limbic system, frontal cortex) to enhance comparability across studies and allow for a more integrative interpretation of the results. A comprehensive description of the specific brain regions included within each system is provided in Supplementary Information (Appendix 2). Furthermore, drug administration paradigms were classified as acute if antipsychotics were administered for a maximum of five days, and chronic if treatment lasted longer. This classification was adopted in light of the high heterogeneity in treatment definitions across studies, with the term “subchronic” referring to durations ranging from 3 to 7 days [50, 51].

### Risk of bias / Methodological quality appraisal

Two authors (MC, BM) independently assessed the risk of bias of included studies. Disagreements were addressed through discussion among the authors, with the involvement of a senior author (AdB) when consensus could not be reached. The methodological quality of individual studies was assessed using the Systematic Review Centre for Laboratory Animal Experimentation (SYRCLE) Tool for animal studies and the CAMARADES checklist [52].

### Statistical analysis

Meta-analyses were performed using a random-effects model [53]. The standardized mean difference (SMD) together with its confidence intervals (95%CIs) was used as the effect size measure. Heterogeneity was assessed using τ² and I² statistics [54], as well as Cochran’s Q test [55]. Prediction intervals were calculated. Heterogeneity was considered high if Cochran’s Q test yielded p < 0.10 or if the I² statistic exceeded 50%. When at least ten studies were available, publication bias was evaluated using funnel plots, and the Egger’s test was applied [56].

Meta-analyses were conducted based on PSD protein type, outcome type (gene expression or protein levels), brain region, and administration paradigm (acute or chronic). Data from different animal species were analyzed separately (rodents and monkeys), while data from rodents (mice and rats) were pooled together, as done in previous meta-analyses [57–59].

Whenever individual studies included multiple experimental conditions in which the same outcome was assessed under different variables, such as the presence or absence of animal models of psychosis or dopamine supersensitivity, sex differences (male and female rodents), or different sacrifice time points, outcomes were meta-analyzed together.

Meta-regressions were performed for pre-defined variables (mean age, percentage of female subjects, rodent type, drug dose, molecular techniques, drug administration route, treatment duration, and the use of animal models of psychosis or dopamine supersensitivity) whenever at least ten studies providing this information were available. Leave-one-out sensitivity analyses were conducted by systematically excluding one study at a time from the main analysis to detect potential outliers that could bias the pooled effect size estimate. Further, we performed sensitivity analyses based on treatment duration by excluding studies in which antipsychotics were provided for a time comprised between 3 and 7 days, in order to address the heterogeneity due to different definitions of administration regimens.

Statistical analyses were performed in RStudio (R version 4.1.2) [60] using the “metafor” package [61], while graphical representations were generated using the “seaborn” [62] and “mayavi” [63] packages in Python. Effect sizes were plotted on the SIGMA brain templates [64].

## Results

A total of 14,561 publications were retrieved from the literature search. After removing duplicates and screening titles, abstracts, and full texts, 256 studies were assessed for eligibility. Of these, 175 were excluded, leaving 81 studies for inclusion in the systematic review, of which 52 were meta-analyzed. Findings were extracted from 81 studies, including a total of 2510 rodents and 26 monkeys, resulting in 226 meta-analyses.

**Fig. 1:**
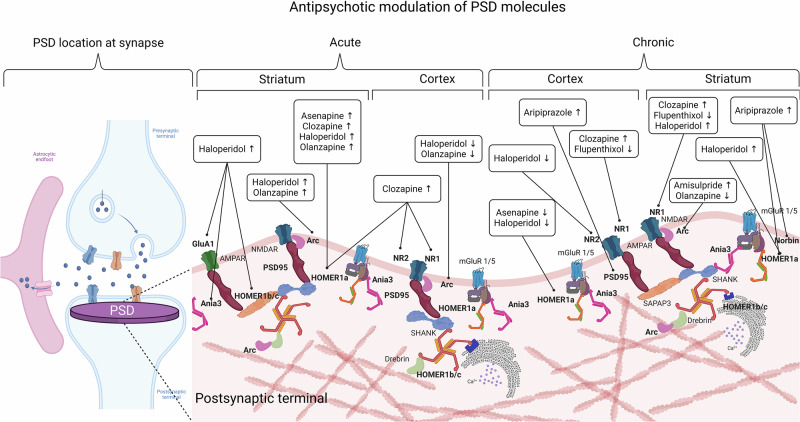
Main outcomes are depicted within the PSD. On the left, the PSD is represented in its synaptic location at glutamatergic synapses. On the right, antipsychotic-induced modifications of main PSD molecules are drawn by splitting the results according to the administration paradigm (acute or chronic) and the brain region (striatum or cortex) considered. Created with BioRender.com.

Data from 18 antipsychotics were retrieved, with haloperidol as the most studied drug (n = 55, 67.9%), followed by clozapine (n = 27, 33.3%), olanzapine (n = 21, 25.9%), and aripiprazole (n = 10, 12.3%). Fifty-three molecular components of the PSD were assessed as outcomes in preclinical studies employing antipsychotic treatments. Of these, the subunit 1 of the N-methyl-D-Aspartate (NMDA) Receptor (NR1) was the most studied molecule (n = 28, 34.6%), followed by Homer1a (n = 20, 24.7%), Arc (n = 18, 22.2%), NR2B (n = 16, 19.8%), NR2A (n = 15, 18.5%), PSD95 (n = 14, 17.3%), and the subunits of the alpha-amino-3-hydroxy-5-methyl-4-isoxazole propionate (AMPA) receptor, GluA1 (n = 10, 12.3%) and GluA2 (n = 9, 11.1%). A chronic administration paradigm was employed in 45 (55.6%) studies, while acute treatment in 24 (29.6%); a combination of acute and chronic regimens was provided in 12 (14.8%) studies. Eleven studies explored antipsychotic effects on PSD outcomes in pharmacological models of psychotic-like behaviors, mostly induced through NMDAR blockers, such as ketamine [39, 40, 65, 66], MK-801 [67–70], and phencyclidine (PCP) [71, 72]. Other non-pharmacological models were adopted to induce cognitive and psychotic-like behaviors in rodents, including chronic, prenatal and perinatal stress [73–75], and genetic disruption, such as dopamine- and cAMP-regulated neuronal phosphoprotein (DARPP-32) knockout [76], Fyn-deficiency, and dominant-negative Disrupted in Schizophrenia 1 (DISC1) [77] transgenic mice. Only three studies explored outcomes in female specimens [71, 78, 79]. Overall, 31 (38.3%) studies were considered as high risk of bias (SYRCLE or CAMARADES score < 5). Primary outcomes are summarized in Fig. 1 while comprehensive results are provided in Supplementary Information (Appendix 5).

### Antipsychotic drugs with high D_2_R affinity

**Fig. 2:**
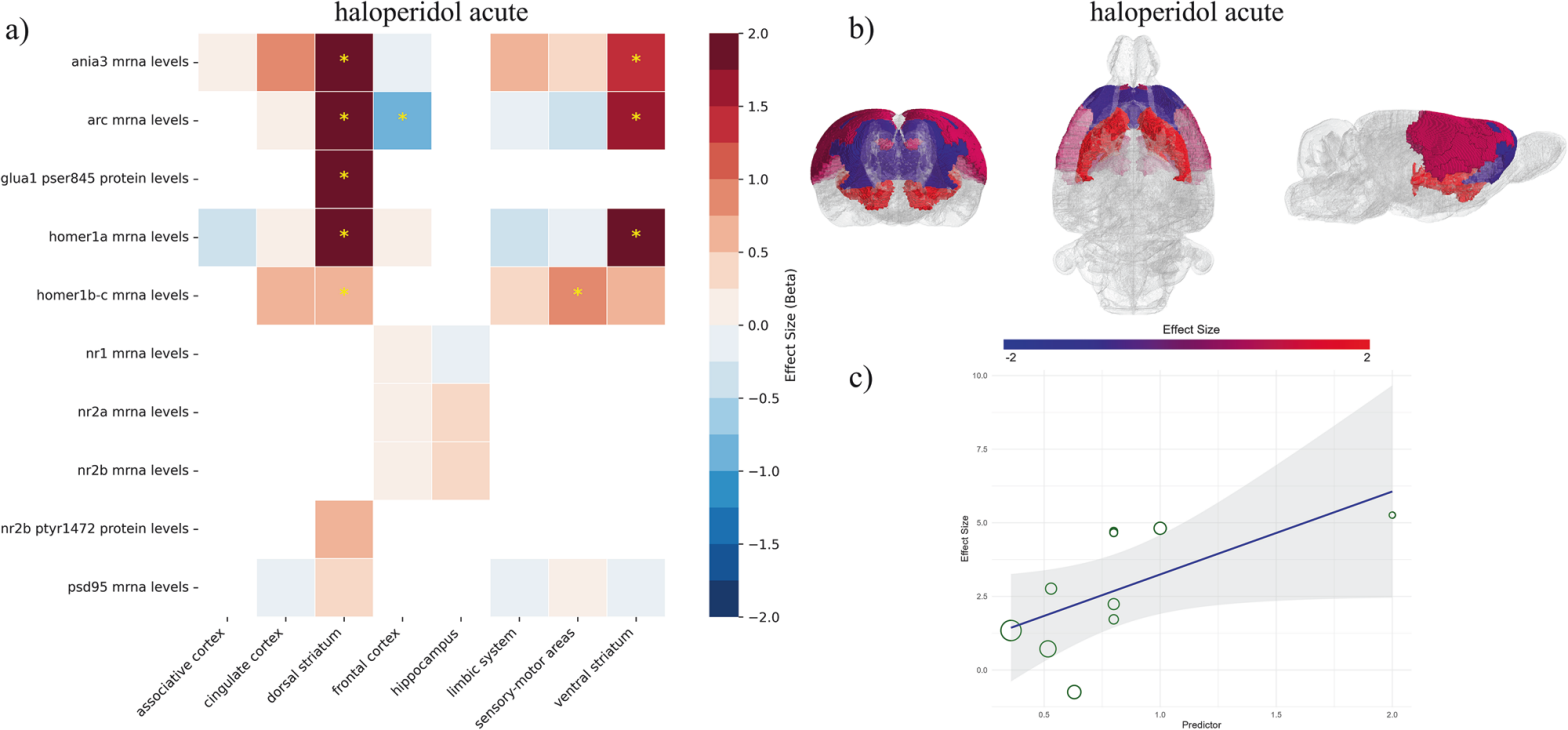
Effects of acute treatment with haloperidol on PSD outcomes. (**a**) A heatmap was employed to describe the modulation of each molecule across different brain regions. The color codes the effect size, with positive values in red and negative values in blue. Yellow stars were used to mark significant values. (**b**) A spatial representation of brain regions modulated by acute treatment with haloperidol was employed in the SIGMA rat brain template. Effect sizes are coded in red or blue to depict only significant values. Notably, acute haloperidol significantly affected the ventral and dorsal striatum by increasing IEGs levels, whereas it reduced Arc mRNA levels in frontal areas. Effects on sensory-motor areas were not preserved after leave-one-out sensitivity analyses. To produce topographical representation of effect sizes, data from different molecules measured in the same brain region were pooled by adjusting for sample sizes. (**c**) Meta-regression revealed the significant relationship between IEGs levels in the dorsal striatum and haloperidol doses, with increased mRNA levels following higher drug doses. Effect sizes are represented on the y-axis, while drug doses are on the x-axis. Each dot represents an individual study with dimensions proportional to its weight (inverse of variance).

Acute administration of haloperidol was found to significantly impact the PSD by affecting mRNA and protein levels of several molecules across different brain regions (Fig. 2). Specifically, haloperidol significantly increased mRNA levels of immediate early genes (IEGs) in the dorsal and ventral striatum, including Ania3 (SMD[95%CIs]=2.47 [1.08; 3.87], *p* < 0.01, k = 5 in dorsal striatum; SMD = 1.43[0.61; 2.24], *p* < 0.01, k = 5 in ventral striatum), Homer1a (SMD = 2.11[1.43; 2.8], *p* < 0.01, k = 15 in dorsal striatum; SMD = 1.81[1.14; 2.48], *p* < 0.01, k = 14 in ventral striatum), and Arc (SMD = 2.6[1.34; 3.86], *p* < 0.01, k = 10 in dorsal striatum; SMD = 1.73[1.14; 2.32], *p* < 0.01, k = 7 in ventral striatum). Haloperidol also increased phospho-AMPA Receptor 1 (pSer845-GluA1) protein levels in the dorsal striatum (SMD = 2.56[0.68; 4.44], *p* < 0.01, k = 4) while reducing Arc mRNA levels in the frontal cortex (SMD = -0.95[-1.33; -0.57], *p* < 0.01; k = 5). Acute treatment with haloperidol increased Homer1b/c in the dorsal striatum (SMD = 0.69[0.01; 1.38], *p* = 0.046; k = 3) and sensory-motor areas (SMD = 0.78[0.2; 1.36], *p* < 0.01; k = 3). Changes in Homer1b/c did not survive to leave-one-out sensitivity analyses nor to p-value correction for multiple testing (Supplementary Information, Appendix 5-6).

Publication bias was significantly detected for haloperidol’s modulation of Arc mRNA in the dorsal striatum (Egger’s test: *beta* = 2.68, *p* < 0.01), and Homer1a mRNA levels in the dorsal (Egger’s test: *beta* = 6.54, *p* < 0.01) and ventral (Egger’s test: *beta* = 5.08, *p* < 0.01) striatum.

Meta-regressions (Supplementary Information, Appendix 7) revealed a significant relationship between drug doses and Arc mRNA levels in the dorsal striatum (*beta* = 3.09, *p* = 0.025), with higher antipsychotic doses correlating with increased Arc expression. Differences in lab techniques (*beta* = -0.41, *p* = 0.186) and in rodent specimens (*beta* = 0.45, *p* = 0.37) did not significantly affect the outcomes. Meta-regressions also revealed a lower ability of haloperidol to influence Homer1a mRNA levels in both the dorsal (*beta* = -0.48, *p* = 0.023) and ventral (*beta* = -0.46, *p* = 0.027) striatum in ketamine-induced psychotic-like animal models. In the cingulate cortex, Homer1a mRNA levels were influenced by the administration route (*beta* = -0.99, *p* = 0.027), with lower levels observed after subcutaneous versus intraperitoneal injections.

**Fig. 3:**
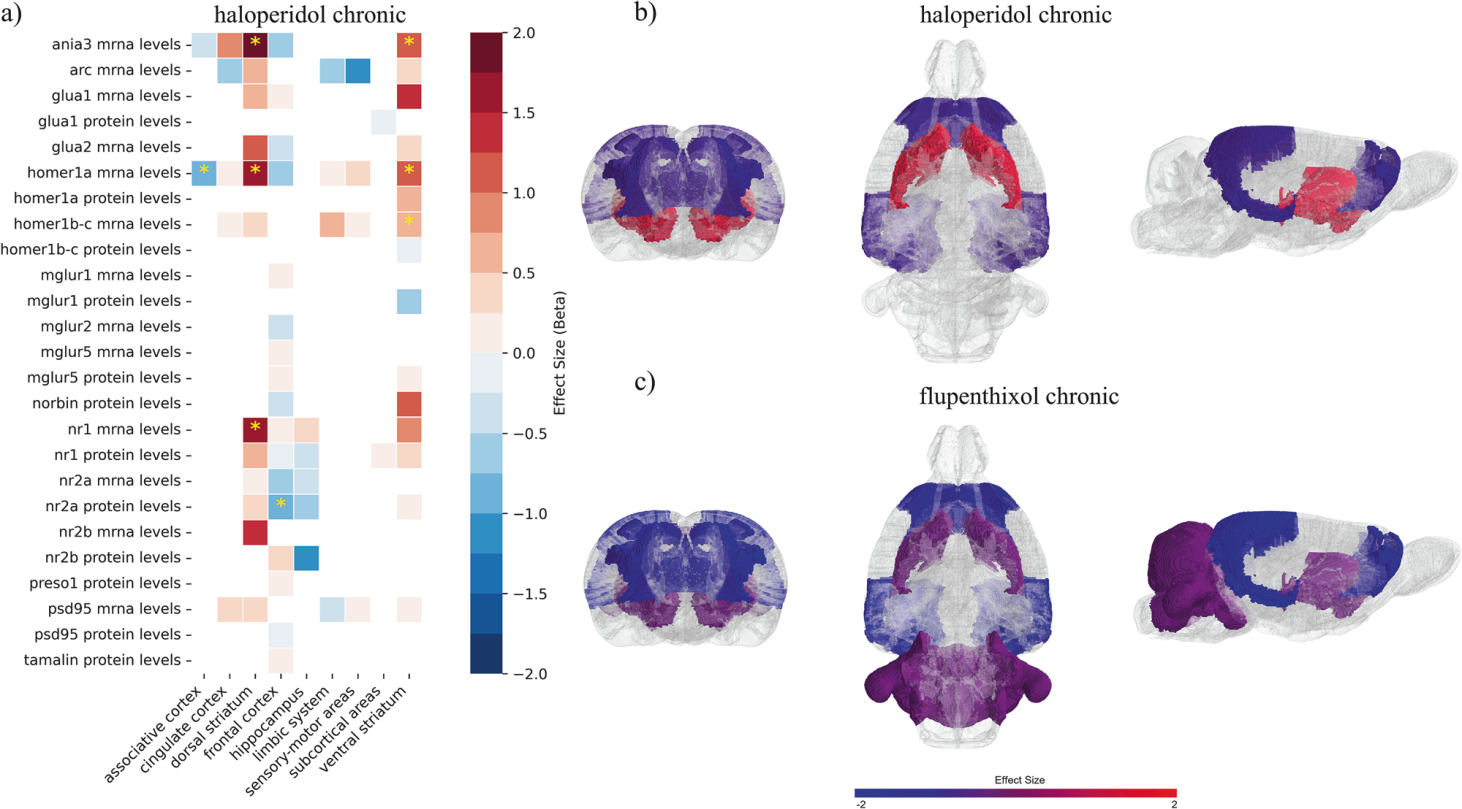
Effects of chronic treatment with first-generation antipsychotics on PSD outcomes. (**a**) A heatmap was employed to describe the modulation of each molecule across different brain regions after chronic administration of haloperidol. The color codes the effect size, with positive values in red and negative values in blue. Yellow stars were used to mark significant values. (**b**) Brain regions significantly modulated by chronic haloperidol administration were plotted in the SIGMA rat brain template. Effect sizes are coded in red or blue to depict only significant values. Notably, haloperidol significantly increased PSD outcomes in the ventral and dorsal striatum. Outcomes were significantly decreased in frontal and associative areas, although results could be affected by biases, as discussed in the main text. Spatial representation of effect sizes was achieved by pooling results from different molecules assessed in the same brain area. (**c**) Chronic treatment with flupenthixol significantly affected molecules’ levels in the cerebellum, subcortical areas, and associative and frontal cortex.

Chronic treatment with haloperidol significantly modulated NMDAR subunits (Fig. 3), increasing NR1 mRNA levels in the dorsal striatum (SMD = 1.59[0.49; 2.69], *p* < 0.01, k = 5) and decreasing NR2A protein levels in the frontal areas (SMD = -0.95[-1.83; -0.07], *p* = 0.034, k = 3), although the latter did not survive to sensitivity analyses. Further, haloperidol effectively increased mRNA levels of Ania3 and Homer1a in both the dorsal (SMD = 2.94[1.93; 3.95], *p* < 0.01, k = 3; SMD = 1.69[1.15; 2.24], *p* < 0.01, k = 10) and ventral striatum (SMD = 1.12[0.23; 2.02], *p* = 0.014, k = 2; SMD = 1.18[0.77; 1.59], *p* < 0.01, k = 9). Otherwise, Homer1a mRNA levels were decreased in the associative cortex (SMD = -0.92[-1.76; -0.073], *p* = 0.033, k = 2). Further, Homer1b/c mRNA levels increased in the ventral striatum (SMD = 0.68[0.03; 1.33], *p* = 0.039, k = 6).

A significant publication bias was detected for haloperidol modulation of Homer1a mRNA levels in the dorsal striatum (Egger’s test: *beta* = 2.08, *p* = 0.038). Similar to the acute administration paradigm, meta-regression revealed a significant dose-dependent effect on Homer1a mRNA levels in the dorsal striatum (beta=5.1, *p* < 0.01), with higher doses correlating with increased gene expression. However, in contrast to acute administration, chronic haloperidol treatment showed no significant influence based on drug administration route or animal models of psychosis.

In both acute and chronic treatments, haloperidol did not significantly affect PSD95 mRNA levels, probably due to the high heterogeneity detected (Supplementary Information, Appendix 5).

After chronic treatment, flupenthixol was seen to significantly decrease NR1 mRNA levels in the associative (SMD = -1.69[-2.5; -0.88], *p* < 0.01, k = 2) and frontal cortex (SMD = -1.5[-2.37; -0.64], *p* < 0.01, k = 2), subcortical areas (SMD = -1.34[-2.14; -0.54], *p* < 0.01, k = 2), and cerebellum (SMD = -1.44[-2.22; -0.66], *p* < 0.01, k = 2). These results come from different experiments within the same study [80], which investigated both cis- and trans-flupenthixol and measured outcomes at different time points. Sensitivity analyses were not feasible and the study showed poor quality at the risk of bias assessment.

### Antipsychotics with high D_2_R affinity and D_2_R partial agonism

**Fig. 4:**
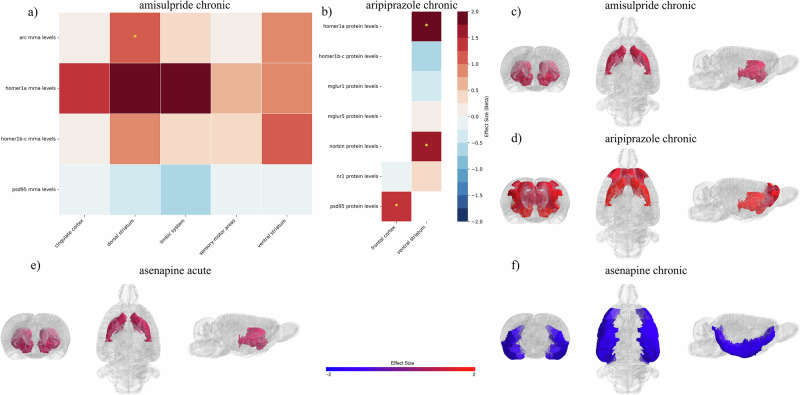
Effects of second-generation antipsychotics on PSD outcomes. (**a**, **b**) Heatmaps were employed to represent effect sizes, ranging from blue (negative values) to red (positive values). Yellow stars marked statistically significant results. (**c**-**f**) Brain regions were mapped onto the SIGMA rat brain template. Effect sizes were pooled across different PSD-related molecules within the same brain region, highlighting striatal modulation by chronic treatment with amisulpride (**c**) and aripiprazole (**d**), and acute treatment with asenapine (**e**). Aripiprazole also increased the levels of molecules in the frontal cortex (**d**). Chronic treatment with asenapine was shown to decrease outcome in the limbic system without significantly affecting the striatum (**f**).

Chronic administration of aripiprazole was associated with elevated Homer1a (SMD = 2.57[0.26;4.89]; *p* = 0.03; k = 2) and Norbin (SMD = 1.73[0.78;2.67]; *p* < 0.01; k = 2) protein levels in the ventral striatum, as well as increased PSD95 protein levels in the frontal cortex (SMD = 1.46[0.47;2.45]; *p* < 0.01; k = 2). Representation of results is provided in Fig. 4.

### Antipsychotics with high/moderate D_2_R affinity

Chronic treatment with amisulpride significantly increased Arc mRNA levels in the dorsal striatum (SMD = 1.24[0.36;2.11]; *p* < 0.01; k = 2).

After acute administration, asenapine significantly increased Homer1a mRNA levels in the dorsal striatum (SMD = 1.02[0.46; 1.57], *p* < 0.01, k = 4). Moreover, sensitivity analyses based on model type revealed a significant increase in Homer1a mRNA levels in the ventral striatum following the removal of results from animal models of psychosis (SMD = 0.86[0.07; 1.64], *p* = 0.03, k = 3). In chronic administration paradigms, asenapine did not significantly affect PSD-related outcomes in the striatum but led to a decrease in Homer1a mRNA levels in the limbic system (SMD = -1.23[-2.08; -0.39], *p* < 0.01, k = 2).

Olanzapine significantly increased Arc mRNA levels in the dorsal striatum (SMD = 1.41[0.37; 2.45], *p* < 0.01, k = 3) following acute administration, whereas chronic treatment led to a reduction in its expression (SMD = -1.58[-2.33; -0.87], *p* < 0.01, k = 2). Additionally, acute olanzapine administration upregulated Homer1a (SMD = 1.85[0.27; 3.42], *p* = 0.02, k = 4) and Arc (SMD = 1.49[0.88; 2.09], *p* < 0.01, k = 2) mRNA levels in the ventral striatum while downregulating Arc mRNA expression in the frontal cortex (SMD = -2.15[-2.7; -1.61], *p* < 0.01, k = 2). Leave-one-out sensitivity analyses showed that chronic administration of olanzapine could also increase NR1 protein levels in the frontal cortex when studies with the highest dose were removed (SMD = 1.00[0.06; 1.93], *p* = 0.037, k = 2).

**Fig. 5:**
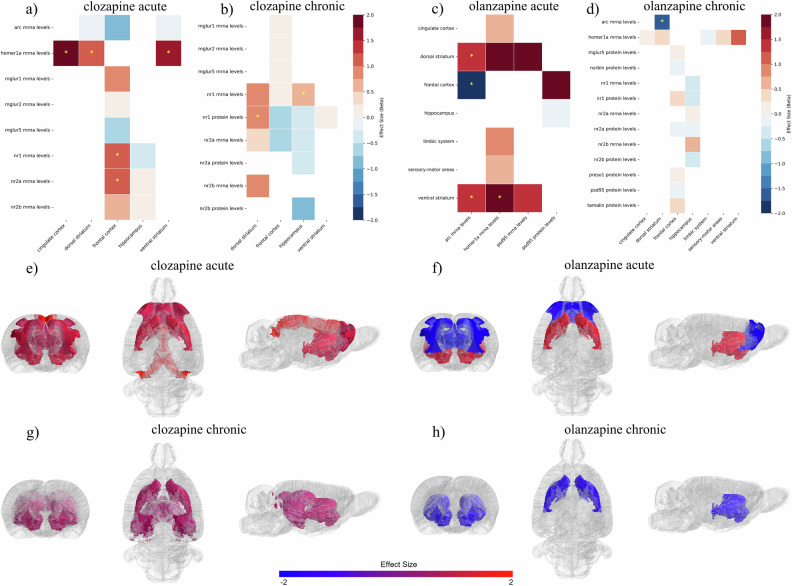
Effects of acute and chronic treatment with both clozapine and olanzapine on PSD molecules. (**a**-**d**) Heatmaps were provided to summarize the effect of acute (**a**) and chronic (**b**) treatment with antipsychotics on PSD mRNA and protein levels across different brain regions. Effect sizes were represented through colors, ranging from blue (negative values) to red (positive values). Statistically significant outcomes were highlighted through yellow stars. (**e**-**h**) Results were produced by pooling effect sizes of different molecules assessed in the same brain area and were plotted in a SIGMA rat brain template. Acute treatment with clozapine (**d**) induced increased PSD outcomes in the cingulate and frontal cortex, as well as in the dorsal and ventral striatum. In acute administration, olanzapine (**e**) was shown to modulate the dorsal and ventral striatum, as well as the frontal cortex. Chronic administration of clozapine (**g**) affected in a minor way dorsal striatum and hippocampus. Following chronic treatment with olanzapine (**h**), reduced outcomes in the dorsal striatum were detected.

Representation of results is provided in Figs. 4,5.

### Antipsychotics with low D_2_R affinity

Acute treatment with clozapine was found to increase Homer1a mRNA levels in the dorsal (SMD = 1.13[0.41; 1.86], *p* < 0.01, k = 3) and ventral striatum (SMD = 1.48[0.72; 2.24], *p* < 0.01, k = 3), as well as in the cingulate cortex (SMD = 2.12[1.12; 3.12], *p* < 0.01, k = 2). Further, clozapine significantly increased mRNA levels of NMDAR subunits, NR1 (SMD = 1.19[0.4; 1.97], *p* < 0.01, k = 3) and NR2A (SMD = 1.01[0.24; 1.78], *p* = 0.01, k = 3), in the frontal cortex. Chronic treatment with clozapine increased NR1 protein levels in the dorsal striatum (SMD = 0.76[0.07; 1.46], *p* = 0.03, k = 2) and NR1 mRNA levels in the hippocampus (SMD = 0.66[0.007; 1.3], *p* = 0.047, k = 3). Of interest, clozapine effects, particularly on NR2A and hippocampal NR1, were more pronounced when animal models of psychosis were considered, as shown by sensitivity analysis based on model type (Appendix 6). The full results are shown in Fig. 5.

Although limited data, quetiapine did not significantly modify PSD protein expression. Specifically, Homer1a mRNA levels in the dorsal striatum were unaffected under both acute (SMD = 0.44[-0.26; 1.14], *p* = 0.97, k = 2) and chronic (SMD = 0.65[-0.8; 2.12], *p* = 0.08, k = 2) administration.

## Discussion

The present systematic review and meta-analysis explored the differential effect of antipsychotics on mRNA and protein levels of PSD molecules in animal models of acute and chronic antipsychotics’ treatment. Overall, antipsychotics differently impacted gene and protein expression of PSD molecules based on receptor profile, mainly D_2_R affinity, drug dose, administration regimen, brain region considered, and type of PSD protein.

### Antipsychotic regulation of PSD molecules and translational implications

Antipsychotics effectively modulated PSD molecules, especially NR1, Arc, and Homer1a, at both mRNA and protein levels. Acute treatments tended to preferentially induce PSD protein with IEGs features [42, 81–83] (i.e., Homer1a and Arc [84]) while chronic exposure was more often associated with changes in constitutive proteins, such as NR1 [85–90], consistent with their role in long-term synaptic plasticity [91].

The receptor binding profile of antipsychotics also influenced the expression pattern of PSD molecules. Compounds with high D_2_R antagonism, such as haloperidol, impacted on a larger number of PSD molecules and exhibited higher effect sizes compared to drugs with lower D_2_R receptor affinity, such as clozapine or quetiapine [43, 81, 92–96]. This evidence highlights the relevance of dopaminergic pathway modulation for antipsychotic-induced synaptic effects. Further, possible dose-dependent relationship was suggested by meta-regressions, with higher doses of haloperidol associated with increased gene expression of PSD proteins. Globally, these findings suggest that increased D_2_R occupancy could be responsible for a progressive recruitment of synaptic components at the molecular level.

Clozapine, the antipsychotic compound with the lowest D_2_R affinity, mainly affected Homer1a expression (as discussed below) whereas amisulpride and olanzapine mostly impacted on Arc mRNA levels [83, 94, 97–99]. These differences may be due to different modulations of the dopaminergic pathway. Specifically, increased Arc levels have been detected after both D_2_R antagonism and D_1_R agonism [100, 101]. In this regard, the D_2_R antagonism exerted by amisulpride and olanzapine could explain their effect on Arc induction in the striatum [82, 83, 93, 94, 99]. Although olanzapine has a low affinity for D_1_R, it has been shown to putatively act as a receptor antagonist under dopamine depletion states, which could account for its ability to decrease Arc expression in the frontal cortex [102]. These region-specific effects on Arc regulation may have translational relevance, as the application of a D_1_R agonist in the prefrontal cortex or a D_2_R antagonist in the ventral striatum were found to rescue cognitive and psychomotor abnormalities in Arc knockout mice, supporting the clinical relevance of dopaminergic modulation and its downstream effects on Arc-related pathways [103].

Antipsychotics-induced effects on PSD protein expression could have translational implications. Specifically, both Homer1a and Arc play essential roles in cognitive functions and have been implicated in the pathophysiology of schizophrenia and affective disorders [104–106]. Rare variants and hypermethylation of the Arc gene [106], as well as, polymorphisms in Homer1 gene [107], were found in patients affected by schizophrenia, correlated to both positive and negative symptoms of psychosis, and associated with treatment response to antipsychotic drugs [107]. Furthermore, Homer1a genotypes significantly affected grey matter volume, brain connectivity, and treatment response during depressive episodes in subjects with bipolar disorder [29]. Similarly, NR1 mRNA and protein levels were found to be reduced in the postmortem prefrontal cortex of individuals with schizophrenia [108, 109]. In first-episode psychosis patients, decreased serum NR1 concentrations were also observed, with peripheral levels negatively correlating with cognitive performances [110]. Reductions of NR1 transcripts were also detected in the postmortem hippocampus of subjects affected by bipolar disorder I [111].

Evidence of synaptic alterations in severe mental illnesses have also been collected, albeit indirectly, by in vivo human neuroimaging studies [112, 113]. Specifically, reduced levels of synaptic proteins, as measured by positron emission tomography (PET) detection of synaptic vesicle glycoprotein 2 A (SV2A), have been correlated to cognitive dysfunction, especially in working memory and executive function, as well as, to mood and anxiety symptoms [112]. Dopamine-glutamate interaction, assessed through a combination of ^18^F-DOPA-PET and magnetic resonance spectroscopy (MRS), in which the PSD has a well-known relevant role, has been found to be altered in first-episode psychosis patients compared to healthy controls [113]. The onset of antipsychotic treatment normalized dopamine-glutamate associations in patients, along with clinical improvement in positive, negative, and general psychopathological symptoms [113].

Overall, the well-documented involvement of PSD molecules in the pathophysiology of severe mental disorders, such as schizophrenia and bipolar disorder, along with their changes in the course of antipsychotic treatment may strengthen their relevance as molecular targets in psychiatric diseases and potential biomarkers of treatment response/resistance. Moreover, the detection of short-term changes in IEGs, markers of regional neuronal activation, could explain the early symptomatic improvements observed after acute antipsychotic administration [114]. Conversely, the modulation of proteins critically involved in long-term synaptic plasticity, such as NR1, under chronic but not acute administration may underlie the necessity of prolonged treatment to achieve sustained clinical effects and relapse prevention [115, 116].

### Topographic targets of antipsychotic drug effects

Most antipsychotics included in this meta-analysis showed significant modulation of PSD molecules in both the dorsal and ventral striatum, which were the most affected brain areas. The striatum has been consistently linked to the pathogenesis of psychotic disorders, as well as to the therapeutic efficacy and side effects of antipsychotic drugs, in coherence with its role in dopaminergic neurotransmission. Furthermore, the striatum has been considered a relevant locus for dopamine-glutamate interactions, which largely depend on PSD molecules and are essential to attend learning processes and cognitive functions [117, 118]. In this regard, antipsychotics, with their ability to occupy the D_2_R at the striatal level, may affect PSD outcomes through the modulation of the dopaminergic pathway.

A cornerstone of the neurobiology underlying psychotic disorders lies on the observation of increased striatal dopamine release in schizophrenia subjects compared to healthy controls after stimulant administration (e.g., amphetamines), as assessed by ^11^C-raclopride- or ^11^C-NPA-PET [119–122]. Striatal dopamine function has also been associated with the severity of psychotic symptoms and their progression in the course of schizophrenia and bipolar disorder [123–125]. Clinical studies have shown that haloperidol, amisulpride, and aripiprazole increase volume, metabolic activity, and functional connectivity of striatal areas in patients affected by schizophrenia, along with improvements in clinical symptoms and the occurrence of extrapyramidal symptoms [126–130]. Globally, this evidence is aligned with our results, highlighting the striatum as a primary target of antipsychotic action and supporting its translational relevance in the treatment of psychotic symptoms [131].

Different from other antipsychotic drugs, olanzapine showed a pattern suggestive of reduced PSD-related outcomes after chronic administration [82, 93], while acute treatment appeared to increase expression of these molecules in striatal regions [82, 83, 94]. Interestingly, this pattern mirrors translational results of clinical trials, in which a single acute dose of olanzapine was found to significantly increase blood flow in the dorsal and ventral striatum, while one-year treatment led to a significant reduction in caudate nucleus volume in patients with psychotic disorders [132, 133]. These findings suggest that, while the acute effects of olanzapine may resemble those of haloperidol and reflect dopaminergic mechanisms, its long-term effects might be driven by alternative neurotransmitter pathways.

Aripiprazole, haloperidol, and olanzapine were effective in modulating PSD molecules in frontal areas. Haloperidol, under both acute and chronic administration regimens, downregulated PSD molecules in the frontal cortex, similarly to acute olanzapine [42, 43, 82, 83, 90, 134–136]. Otherwise, chronic aripiprazole increased the expression of PSD molecules in frontal regions [74]. In patients with schizophrenia, chronic treatment with haloperidol was associated with decreased cerebral blood flow in the orbitofrontal and prefrontal cortices, probably through the indirect striatal-thalamo-cortical loop modulation, which may account for haloperidol-induced impairments on working memory [126, 137]. Consistently, in non-human primates, chronic haloperidol administration significantly reduced NR1 and spinophilin protein levels in frontal regions, mirroring our observation of decreased NR2A protein levels in the same areas of rodents [43, 78, 90, 136, 138]. Olanzapine has been shown to modulate prefrontal cortex activity during emotional processing in patients with schizophrenia, with reduced activation observed after short-term treatment, along with an improvement in both positive and negative psychotic symptoms [139]. Aripiprazole administration has been associated with structural neuroplastic changes in the prefrontal cortex, which underlay an attenuation of psychotic-like symptoms in rats [140]. In humans, aripiprazole has also been shown to induce prefrontal cortex activation, along with improvements in cognitive performances, including enhanced discriminability and speeded reaction times [141].

**Fig. 6:**
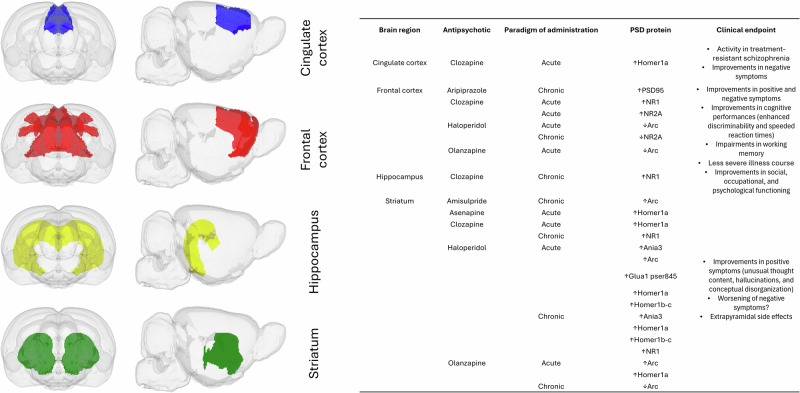
Possible clinical endpoints associated with each antipsychotic drug. On the left, the four brain regions primarily modulated by antipsychotics are shown according to the SIGMA rat brain template. On the right, potential clinical endpoints are summarized for each brain region, providing a comprehensive overview of the results from the present meta-analysis. The figure disentangles the effects of individual antipsychotics on different PSD proteins, considering both acute and chronic paradigms of administration.

Overall, regional differences in antipsychotics’ loci of action may explain the variability in clinical responses and drug side effects. Compounds with more pronounced striatal effects may be effective in normalizing subcortical-cortical connectivity and counteracting acute psychotic symptoms, and, at the same time, accounting for the higher incidence of extrapyramidal symptoms [142–145]. Otherwise, antipsychotics with an impact on the frontal cortex may affect cognitive symptoms [146–148]. Possible clinical endpoints associated with each antipsychotic, specific for each brain region, have been provided in Fig. 6.

### The unique molecular profile of clozapine

Among the antipsychotics included in the present meta-analysis, clozapine exhibited a unique molecular signature.

First, clozapine showed to acutely modulate glutamatergic neurotransmission through its action on NR1 and Homer1a mRNA levels [75, 85, 89, 90]. The NR1 subunit has been found to be downregulated in the prefrontal cortex of patients with schizophrenia [108, 109], suggesting that at least a part of the clinical efficacy of clozapine may depend on a rapid and strong modulation of the NMDAR pathway. Supporting this hypothesis, clinical trials have shown that augmentation strategies with NMDAR modulators are generally ineffective in patients already receiving clozapine, likely due to a “ceiling effect” on NMDAR-mediated transmission [149].

Clozapine induced Homer1a mRNA expression in the cingulate cortex, and dorsal and ventral striatum [97, 98, 150]. Homer1 has been involved in the mGluR5 signaling, whose modulation may be related to clozapine administration [151]. In mice exposed to chronic stress, Homer1/mGluR5 coupling was found to be disrupted and behavioral alterations mirroring psychotic-like symptoms were detected in animals with Homer1 gene deletion [151]. In the same paradigm, the administration of mGluR5 pharmacological modulators was able to restore the observed behavioral alterations [151]. A recent clinical study has revealed a significant contribution from mGluR5 in structural covariance networks of individuals affected by TRS, which may explain clozapine’s unique efficacy in these refractory cases [152]. Recent findings suggest that clozapine’s improvements in sensorimotor gating, an endophenotype of schizophrenia characterized by deficits in information processing, depend on the modulation of p11, which regulates mGluR5 synaptic clustering [153] and has also been implicated in antidepressant effects and suicide risk reduction [154], potentially explaining the observed efficacy of clozapine in reducing suicide risk [155].

Most of the pharmacological agents used to mirror psychotic-like symptoms in a preclinical setting, such as PCP, ketamine, and MK-801, act mainly albeit not exclusively by blocking NMDAR [156–158]. Moreover, non-pharmacological models like perinatal or prenatal stress have also been shown to alter glutamatergic signaling, particularly within frontal regions [159–161]. The robust modulation of the glutamatergic signaling exerted by clozapine, through the inhibition of the glycine transporter 1 and possible agonist activity at the glycine binding site of NMDARs [162], could explain our observation of its greater effects in studies employing an animal model of psychosis [67]. In addition, clozapine has been shown to restore the intrinsic electrophysiological and synaptic properties of glutamatergic neurons derived from individuals with schizophrenia. These effects have been potentially correlated with clinical response, providing a possible translational support for the hypothesis that modulation of the glutamatergic pathway contributes to the therapeutic efficacy of clozapine [163].

Clozapine was found to modulate PSD molecules across the frontal cortex, striatum, and cingulate cortex [97, 98, 150]. Longitudinal studies suggest that the structural integrity of the frontal cortex may be a prerequisite for clozapine’s therapeutic efficacy, and its volume could be a predictor of symptom improvement [164–166]. Alterations in frontal areas have been specifically observed in individuals with ultra-treatment-resistant schizophrenia (UTRS), who fail to respond even to clozapine, when compared to TRS patients responsive to clozapine [167]. In patients with schizophrenia, treatment with clozapine has also been associated with increased cortico-striatal connectivity, which positively correlated with clinical responsiveness [168]. In preclinical models, clozapine has been shown to reverse antipsychotic-induced behavioral supersensitivity, an animal proxy of clinical treatment resistance and tardive dyskinesia, through glutamatergic modulation of striatal regions [169, 170].

In the present study, clozapine was the only antipsychotic that showed a significant effect on PSD molecules in the cingulate cortex, although the limited number of studies warrants cautious interpretation [97, 98]. A hyperglutamatergic state in the anterior cingulate cortex has been consistently reported as a neurobiological hallmark of TRS, for which clozapine remains the only approved and effective treatment option [171–175]. Neuroimaging studies have further identified functional alterations in the anterior cingulate cortex in TRS patients compared to treatment-responsive individuals (nTRS), including findings from ^18^F-fluorodeoxyglucose (^18^F-FDG-PET) imaging, where glucose metabolism serves as an indirect marker of glutamatergic neurotransmission [45, 176]. In this regard, clozapine, but not other antipsychotic drugs, has been shown to rescue alterations in task-evoked regional cerebral blood flow in the anterior cingulate cortex of patients with schizophrenia [177]. Collectively, these previous findings, along with our observations, highlight the relevance of striatal-frontal regions and cingulate cortex modulation in mediating clozapine’s therapeutic effects.

### Limitations and conclusion

The present meta-analysis has limitations that should be acknowledged. First, high heterogeneity was observed across studies. However, conducting analyses stratified by brain region and type of molecule, and applying meta-regressions, we could identify the main contributors to this heterogeneity [178]. Second, many of the included comparisons were based on small sample sizes [179], which is often observed in preclinical meta-analyses (http://camarades.de/). Third, publication bias was detected in some comparisons (i.e., haloperidol’s modulation of IEGs in striatal areas). Fourth, findings related to acute administration were based on a limited number of studies and should therefore be interpreted with caution, requiring further replication to strengthen their reliability and generalizability. Finally, as animal experimentation has traditionally relied on males to avoid hormonal fluctuations as a putative source of bias, sex could not be adequately evaluated as a source of heterogeneity. Although sensitivity analyses excluding studies on female animals did not show a relevant impact on the results (Appendix 6), the very limited evidence warrants caution.

In conclusion, this meta-analysis highlights that acute and chronic antipsychotics’ administration may significantly change the expression of PSD proteins based on the drug receptor profile and its D_2_R affinity/occupancy, the duration of drug administration, the brain region analyzed, and the specific PSD protein detected. Considering the structural and functional role of some proteins whose expression was found changed under drug treatment, it is conceivable that antipsychotics may profoundly affect the architecture and function of the dendritic spine. Further research is needed to better understand if and how these changes are related to the therapeutic effects of antipsychotics.

## Supplementary information

Supplementary Information

## Data Availability

Data can be provided upon request.

## Code availability

Code can be provided upon request.
