# Supplementary material for: Molecular and topographic mapping of antipsychotic effects: a meta-analysis of postsynaptic density proteins in animal models with translational implications
Source: Mol Psychiatry. 2025 Nov 19;31(2):1142–55. doi: 10.1038/s41380-025-03351-z (PMC12815681; doi:10.1038/s41380-025-03351-z)
Supplement: Supplementary file 1 — Supplementary Information [file 41380_2025_3351_MOESM1_ESM.docx]

**Molecular and topographic mapping of antipsychotic effects: a meta-analysis of postsynaptic density proteins in animal models with translational implications**

Giuseppe De Simone^1,2,3,4*^, MD

Michele De Prisco^2,3,4*^, MD

Vincenzo Oliva^2,3,4^, MD

Licia Vellucci^1^, MD

Mariateresa Ciccarelli^1^, MD, PhD

Benedetta Mazza^1^, MD,

Giovanna Fico^2,3,4^ MD, PhD

Michele Fornaro^1^, MD, PhD

Felice Iasevoli^1^, MD, PhD

Eduard Vieta^2,3,4^ MD, PhD

Andrea de Bartolomeis^1§^, MD, PhD

^1^ Section of Psychiatry. Laboratory of Molecular and Translational Psychiatry. Unit of Treatment-Resistant Psychiatric Disorders. Department of Neuroscience, Reproductive Sciences and Dentistry, University of Naples "Federico II", School of Medicine, Naples, Italy. Via Pansini 5, 80131 Naples, Italy

^2^ Bipolar and Depressive Disorders Unit, Hospìtal Clinic de Barcelona, c. Villarroel, 170, 08036 Barcelona, Spain

^3^ Departament de Medicina, Facultat de Medicina i Ciències de la Salut, Institut de Neurociències, Universitat de Barcelona (UB), c. Casanova, 143, 08036 Barcelona, Spain

^4^ Institut d’Investigacions Biomèdiques August Pi i Sunyer (IDIBAPS), c. Villarroel, 170, 08036 Barcelona, Spain

***^*^These authors contributed equally to the work***

**^§^Please address correspondence to:** Andrea de Bartolomeis MD, PhD, Laboratory of Molecular and Translational Psychiatry and Unit of Treatment-Resistant Psychiatric Disorders, Section of Psychiatry, Department of Neuroscience, Reproductive Science and Dentistry. University School of Medicine "Federico II," Building 18, 3rd floor, Via Pansini 5, Naples, Italy. ZIP 80131. Email to [adebarto@unina.it](mailto:adebarto@unina.it)

Summary

[Appendix 1 3](#_Toc209350545)

[Deviations from protocol 3](#_Toc209350546)

[Search strategy 4](#_Toc209350547)

[Appendix 2 10](#_Toc209350548)

[Antipsychotics included 10](#_Toc209350549)

[PSD molecules included 11](#_Toc209350550)

[Macroregions 12](#_Toc209350551)

[Appendix 3 13](#_Toc209350552)

[PRISMA flow diagram 13](#_Toc209350553)

[Overview of included studies 14](#_Toc209350554)

[Risk of bias 20](#_Toc209350555)

[CAMARADES Checklist 24](#_Toc209350556)

[Appendix 4 28](#_Toc209350557)

[Studies excluded 28](#_Toc209350558)

[Appendix 5 33](#_Toc209350559)

[Antipsychotics retrieved 33](#_Toc209350560)

[PSD molecules retrieved 34](#_Toc209350561)

[Results 35](#_Toc209350562)

[Results after adjustment of p-values for multiple testing 48](#_Toc209350563)

[Highlight on significant results 65](#_Toc209350564)

[Appendix 6 67](#_Toc209350565)

[Leave-one-out sensitivity analyses 67](#_Toc209350566)

[Sensitivity analyses based on quality assessment 105](#_Toc209350567)

[Sensitivity analyses based on treatment duration 124](#_Toc209350568)

[Sensitivity analyses based on animal models of psychosis 144](#_Toc209350569)

[Sensitivity analyses based on animal sex 159](#_Toc209350570)

[Appendix 7 176](#_Toc209350571)

[Meta-regressions 176](#_Toc209350572)

[References 178](#_Toc209350573)

Appendix 1

## Deviations from protocol

Originally, sensitivity analyses were planned based on study quality and using a leave-one-out approach. However, given the variability in how acute and chronic treatment were defined across studies, to ensure robustness of results, we also conducted sensitivity analyses excluding studies in which treatment duration ranged from 3 to 7 days.

## Search strategy

PubMed/MEDLINE (up to 08/01/2025: 2915)

("synaptophysin"[Title/Abstract] OR "sap97"[Title/Abstract] OR "psd95"[Title/Abstract] OR "postsynaptic density"[Title/Abstract] OR "post synaptic density"[Title/Abstract] OR "n methyl d aspartate receptor"[Title/Abstract] OR "nmda"[Title/Abstract] OR "metabotropic glutamate receptor"[Title/Abstract] OR "mglur"[Title/Abstract] OR "amino 3 hydroxy 5 methyl 4 isoxazole propionate receptor"[Title/Abstract] OR "ampa"[Title/Abstract] OR "kainate receptor"[Title/Abstract] OR "disrupted in schizophrenia 1"[Title/Abstract] OR "disc1"[Title/Abstract] OR "stargazin"[Title/Abstract] OR "homer1"[Title/Abstract] OR "homer2"[Title/Abstract] OR "homer3"[Title/Abstract] OR "homer"[Title/Abstract] OR "synapse associated protein"[Title/Abstract] OR "shank"[Title/Abstract] OR "tank"[Title/Abstract] OR "traf family member associated nfkb activator"[Title/Abstract] OR "psd"[Title/Abstract] OR "sap102"[Title/Abstract] OR "dlg"[Title/Abstract] OR "disc large homolog"[Title/Abstract] OR "arc"[Title/Abstract] OR "grin"[Title/Abstract] OR "glur"[Title/Abstract] OR "glun"[Title/Abstract] OR "nr1"[Title/Abstract] OR "nr2"[Title/Abstract] OR "ankyrin"[Title/Abstract] OR "actin"[Title/Abstract] OR "tanc2"[Title/Abstract] OR "norbin"[Title/Abstract] OR "spinophilin"[Title/Abstract] OR "tamalin"[Title/Abstract] OR "syngap"[Title/Abstract] OR "psd93"[Title/Abstract] OR "kalirin"[Title/Abstract] OR "sap102"[Title/Abstract] OR "shank1"[Title/Abstract] OR "shank2"[Title/Abstract] OR "shank3"[Title/Abstract] OR "sapap"[Title/Abstract] OR "sapap1"[Title/Abstract] OR "sapap2"[Title/Abstract] OR "sapap3"[Title/Abstract] OR "sapap4"[Title/Abstract] OR "dlgap"[Title/Abstract] OR "dlgap1"[Title/Abstract] OR "dlgap2"[Title/Abstract] OR "dlgap3"[Title/Abstract] OR "dlgap4"[Title/Abstract] OR "glur1"[Title/Abstract] OR "glur2"[Title/Abstract] OR "glur3"[Title/Abstract] OR "glun1"[Title/Abstract] OR "glun2a"[Title/Abstract] OR "glun2b"[Title/Abstract]) AND ("amisulpride"[Title/Abstract] OR "aripiprazole"[Title/Abstract] OR "asenapine"[Title/Abstract] OR "benperidol"[Title/Abstract] OR "brexpiprazole"[Title/Abstract] OR "cariprazine"[Title/Abstract] OR "chlorpromazine"[Title/Abstract] OR "clozapine"[Title/Abstract] OR "flupentixol"[Title/Abstract] OR "fluphenazine"[Title/Abstract] OR "haloperidol"[Title/Abstract] OR "levomepromazine"[Title/Abstract] OR "lurasidone"[Title/Abstract] OR "olanzapine"[Title/Abstract] OR "paliperidone"[Title/Abstract] OR "pericyazine"[Title/Abstract] OR "pimozide"[Title/Abstract] OR "prochlorperazine"[Title/Abstract] OR "promazine"[Title/Abstract] OR "quetiapine"[Title/Abstract] OR "risperidone"[Title/Abstract] OR "sulpiride"[Title/Abstract] OR "trifluoperazine"[Title/Abstract] OR "zuclopenthixol"[Title/Abstract] OR "xanomeline"[Title/Abstract] OR "sertindole"[Title/Abstract] OR "sep 363856"[Title/Abstract] OR "antipsychotic agents"[MeSH Terms] OR "clotiapine"[Title/Abstract] OR "tiapride"[Title/Abstract] OR "antipsychotic"[Title/Abstract] OR "antipsychotics"[Title/Abstract] OR "ziprasidone"[Title/Abstract])

EMBASE (up to 08/01/2025: 5927)

(synaptophysin:ti,ab OR sap97:ti,ab OR psd95:ti,ab OR 'postsynaptic density':ti,ab OR 'post synaptic density':ti,ab OR 'n methyl d aspartate receptor':ti,ab OR nmda:ti,ab OR 'metabotropic glutamate receptor':ti,ab OR mglur:ti,ab OR 'amino 3 hydroxy 5 methyl 4 isoxazole propionate receptor':ti,ab OR ampa:ti,ab OR 'kainate receptor':ti,ab OR 'disrupted in schizophrenia 1':ti,ab OR disc1:ti,ab OR stargazin:ti,ab OR homer1:ti,ab OR homer2:ti,ab OR homer3:ti,ab OR homer:ti,ab OR 'synapse associated protein':ti,ab OR shank:ti,ab OR tank:ti,ab OR 'traf family member associated nfkb activator':ti,ab OR psd:ti,ab OR sap102:ti,ab OR dlg:ti,ab OR 'disc large homolog':ti,ab OR arc:ti,ab OR grin:ti,ab OR glur:ti,ab OR glun:ti,ab OR nr1:ti,ab OR nr2:ti,ab OR ankyrin:ti,ab OR actin:ti,ab OR tanc2:ti,ab OR norbin:ti,ab OR spinophilin:ti,ab OR tamalin:ti,ab OR syngap:ti,ab OR psd93:ti,ab OR kalirin:ti,ab OR sap102:ti,ab OR shank1:ti,ab OR shank2:ti,ab OR shank3:ti,ab OR sapap:ti,ab OR sapap1:ti,ab OR sapap2:ti,ab OR sapap3:ti,ab OR sapap4:ti,ab OR dlgap:ti,ab OR dlgap1:ti,ab OR dlgap2:ti,ab OR dlgap3:ti,ab OR dlgap4:ti,ab OR glur1:ti,ab OR glur2:ti,ab OR glur3:ti,ab OR glun1:ti,ab OR glun2a:ti,ab OR glun2b:ti,ab) AND (amisulpride:ti,ab OR aripiprazole:ti,ab OR asenapine:ti,ab OR benperidol:ti,ab OR brexpiprazole:ti,ab OR cariprazine:ti,ab OR chlorpromazine:ti,ab OR clozapine:ti,ab OR flupentixol:ti,ab OR fluphenazine:ti,ab OR haloperidol:ti,ab OR levomepromazine:ti,ab OR lurasidone:ti,ab OR olanzapine:ti,ab OR paliperidone:ti,ab OR pericyazine:ti,ab OR pimozide:ti,ab OR prochlorperazine:ti,ab OR promazine:ti,ab OR quetiapine:ti,ab OR risperidone:ti,ab OR sulpiride:ti,ab OR trifluoperazine:ti,ab OR zuclopenthixol:ti,ab OR xanomeline:ti,ab OR sertindole:ti,ab OR 'sep 363856':ti,ab OR 'antipsychotic agents'/exp OR clotiapine:ti,ab OR tiapride:ti,ab OR antipsychotic:ti,ab OR antipsychotics:ti,ab OR ziprasidone:ti,ab)

Scopus (up to 08/01/2025: 3147)

(TITLE-ABS(synaptophysin) OR TITLE-ABS(sap97) OR TITLE-ABS(psd95) OR TITLE-ABS("postsynaptic density") OR TITLE-ABS("post synaptic density") OR TITLE-ABS("n methyl d aspartate receptor") OR TITLE-ABS(nmda) OR TITLE-ABS("metabotropic glutamate receptor") OR TITLE-ABS(mglur) OR TITLE-ABS("amino 3 hydroxy 5 methyl 4 isoxazole propionate receptor") OR TITLE-ABS(ampa) OR TITLE-ABS("kainate receptor") OR TITLE-ABS("disrupted in schizophrenia 1") OR TITLE-ABS(disc1) OR TITLE-ABS(stargazin) OR TITLE-ABS(homer1) OR TITLE-ABS(homer2) OR TITLE-ABS(homer3) OR TITLE-ABS(homer) OR TITLE-ABS("synapse associated protein") OR TITLE-ABS(shank) OR TITLE-ABS(tank) OR TITLE-ABS("traf family member associated nfkb activator") OR TITLE-ABS(psd) OR TITLE-ABS(sap102) OR TITLE-ABS(dlg) OR TITLE-ABS("disc large homolog") OR TITLE-ABS(arc) OR TITLE-ABS(grin) OR TITLE-ABS(glur) OR TITLE-ABS(glun) OR TITLE-ABS(nr1) OR TITLE-ABS(nr2) OR TITLE-ABS(ankyrin) OR TITLE-ABS(actin) OR TITLE-ABS(tanc2) OR TITLE-ABS(norbin) OR TITLE-ABS(spinophilin) OR TITLE-ABS(tamalin) OR TITLE-ABS(syngap) OR TITLE-ABS(psd93) OR TITLE-ABS(kalirin) OR TITLE-ABS(sap102) OR TITLE-ABS(shank1) OR TITLE-ABS(shank2) OR TITLE-ABS(shank3) OR TITLE-ABS(sapap) OR TITLE-ABS(sapap1) OR TITLE-ABS(sapap2) OR TITLE-ABS(sapap3) OR TITLE-ABS(sapap4) OR TITLE-ABS(dlgap) OR TITLE-ABS(dlgap1) OR TITLE-ABS(dlgap2) OR TITLE-ABS(dlgap3) OR TITLE-ABS(dlgap4) OR TITLE-ABS(glur1) OR TITLE-ABS(glur2) OR TITLE-ABS(glur3) OR TITLE-ABS(glun1) OR TITLE-ABS(glun2a) OR TITLE-ABS(glun2b)) AND (TITLE-ABS(amisulpride) OR TITLE-ABS(aripiprazole) OR TITLE-ABS(asenapine) OR TITLE-ABS(benperidol) OR TITLE-ABS(brexpiprazole) OR TITLE-ABS(cariprazine) OR TITLE-ABS(chlorpromazine) OR TITLE-ABS(clozapine) OR TITLE-ABS(flupentixol) OR TITLE-ABS(fluphenazine) OR TITLE-ABS(haloperidol) OR TITLE-ABS(levomepromazine) OR TITLE-ABS(lurasidone) OR TITLE-ABS(olanzapine) OR TITLE-ABS(paliperidone) OR TITLE-ABS(pericyazine) OR TITLE-ABS(pimozide) OR TITLE-ABS(prochlorperazine) OR TITLE-ABS(promazine) OR TITLE-ABS(quetiapine) OR TITLE-ABS(risperidone) OR TITLE-ABS(sulpiride) OR TITLE-ABS(trifluoperazine) OR TITLE-ABS(zuclopenthixol) OR TITLE-ABS(xanomeline) OR TITLE-ABS(sertindole) OR TITLE-ABS("sep 363856") OR INDEXTERMS("antipsychotic agents") OR TITLE-ABS(clotiapine) OR TITLE-ABS(tiapride) OR TITLE-ABS(antipsychotic) OR TITLE-ABS(antipsychotics) OR TITLE-ABS(ziprasidone))

WoS (up to 08/01/2025: 2570)

((TI=synaptophysin OR AB=synaptophysin) OR (TI=sap97 OR AB=sap97) OR (TI=psd95 OR AB=psd95) OR (TI="postsynaptic density" OR AB="postsynaptic density") OR (TI="post synaptic density" OR AB="post synaptic density") OR (TI="n methyl d aspartate receptor" OR AB="n methyl d aspartate receptor") OR (TI=nmda OR AB=nmda) OR (TI="metabotropic glutamate receptor" OR AB="metabotropic glutamate receptor") OR (TI=mglur OR AB=mglur) OR (TI="amino 3 hydroxy 5 methyl 4 isoxazole propionate receptor" OR AB="amino 3 hydroxy 5 methyl 4 isoxazole propionate receptor") OR (TI=ampa OR AB=ampa) OR (TI="kainate receptor" OR AB="kainate receptor") OR (TI="disrupted in schizophrenia 1" OR AB="disrupted in schizophrenia 1") OR (TI=disc1 OR AB=disc1) OR (TI=stargazin OR AB=stargazin) OR (TI=homer1 OR AB=homer1) OR (TI=homer2 OR AB=homer2) OR (TI=homer3 OR AB=homer3) OR (TI=homer OR AB=homer) OR (TI="synapse associated protein" OR AB="synapse associated protein") OR (TI=shank OR AB=shank) OR (TI=tank OR AB=tank) OR (TI="traf family member associated nfkb activator" OR AB="traf family member associated nfkb activator") OR (TI=psd OR AB=psd) OR (TI=sap102 OR AB=sap102) OR (TI=dlg OR AB=dlg) OR (TI="disc large homolog" OR AB="disc large homolog") OR (TI=arc OR AB=arc) OR (TI=grin OR AB=grin) OR (TI=glur OR AB=glur) OR (TI=glun OR AB=glun) OR (TI=nr1 OR AB=nr1) OR (TI=nr2 OR AB=nr2) OR (TI=ankyrin OR AB=ankyrin) OR (TI=actin OR AB=actin) OR (TI=tanc2 OR AB=tanc2) OR (TI=norbin OR AB=norbin) OR (TI=spinophilin OR AB=spinophilin) OR (TI=tamalin OR AB=tamalin) OR (TI=syngap OR AB=syngap) OR (TI=psd93 OR AB=psd93) OR (TI=kalirin OR AB=kalirin) OR (TI=sap102 OR AB=sap102) OR (TI=shank1 OR AB=shank1) OR (TI=shank2 OR AB=shank2) OR (TI=shank3 OR AB=shank3) OR (TI=sapap OR AB=sapap) OR (TI=sapap1 OR AB=sapap1) OR (TI=sapap2 OR AB=sapap2) OR (TI=sapap3 OR AB=sapap3) OR (TI=sapap4 OR AB=sapap4) OR (TI=dlgap OR AB=dlgap) OR (TI=dlgap1 OR AB=dlgap1) OR (TI=dlgap2 OR AB=dlgap2) OR (TI=dlgap3 OR AB=dlgap3) OR (TI=dlgap4 OR AB=dlgap4) OR (TI=glur1 OR AB=glur1) OR (TI=glur2 OR AB=glur2) OR (TI=glur3 OR AB=glur3) OR (TI=glun1 OR AB=glun1) OR (TI=glun2a OR AB=glun2a) OR (TI=glun2b OR AB=glun2b)) AND ((TI=amisulpride OR AB=amisulpride) OR (TI=aripiprazole OR AB=aripiprazole) OR (TI=asenapine OR AB=asenapine) OR (TI=benperidol OR AB=benperidol) OR (TI=brexpiprazole OR AB=brexpiprazole) OR (TI=cariprazine OR AB=cariprazine) OR (TI=chlorpromazine OR AB=chlorpromazine) OR (TI=clozapine OR AB=clozapine) OR (TI=flupentixol OR AB=flupentixol) OR (TI=fluphenazine OR AB=fluphenazine) OR (TI=haloperidol OR AB=haloperidol) OR (TI=levomepromazine OR AB=levomepromazine) OR (TI=lurasidone OR AB=lurasidone) OR (TI=olanzapine OR AB=olanzapine) OR (TI=paliperidone OR AB=paliperidone) OR (TI=pericyazine OR AB=pericyazine) OR (TI=pimozide OR AB=pimozide) OR (TI=prochlorperazine OR AB=prochlorperazine) OR (TI=promazine OR AB=promazine) OR (TI=quetiapine OR AB=quetiapine) OR (TI=risperidone OR AB=risperidone) OR (TI=sulpiride OR AB=sulpiride) OR (TI=trifluoperazine OR AB=trifluoperazine) OR (TI=zuclopenthixol OR AB=zuclopenthixol) OR (TI=xanomeline OR AB=xanomeline) OR (TI=sertindole OR AB=sertindole) OR (TI="sep 363856" OR AB="sep 363856") OR (TI=clotiapine OR AB=clotiapine) OR (TI=tiapride OR AB=tiapride) OR (TI=antipsychotic OR AB=antipsychotic) OR (TI=antipsychotics OR AB=antipsychotics) OR (TI=ziprasidone OR AB=ziprasidone))

Appendix 2

## Antipsychotics included

| Amisulpride |
| --- |
| Aripiprazole |
| Asenapine |
| Benperidol |
| Brexpiprazole |
| Cariprazine |
| Chlorpromazine |
| Clotiapine |
| Clozapine |
| Flupentixol |
| Fluphenazine |
| Haloperidol |
| Levomepromazine |
| Lurasidone |
| Olanzapine |
| Paliperidone |
| Pericyazine |
| Pimozide |
| Prochlorperazine |
| Promazine |
| Quetiapine |
| Risperidone |
| Sertindole |
| SEP-363856 |
| Sulpiride |
| Tiapride |
| Trifluoperazine |
| Xanomeline |
| Ziprasidone |
| Zuclopenthixol |

## PSD molecules included

| Actin |
| --- |
| ADP/ATP translocase |
| AMPAR |
| Ania3 |
| Arc |
| BRAG1 |
| BRAG2b |
| CaMKII |
| Citron |
| CPG2 |
| Cylindromatosis |
| Densin 180 |
| DISC1 |
| DLGAP |
| GluA1 |
| GluA2 |
| GluA3 |
| GluA4 |
| GluK1 |
| GluK2 |
| GluK3 |
| Glutamine synthetase |
| Heat shock cognate 71 |
| Homer |
| IRSp53 |
| KA1 |
| KA2 |
| Kalirin |
| mGlur |
| Myosin |
| Neuroligin |
| NMDAR |
| NMDAR1 |
| NMDAR2A |
| NMDAR2B |
| Norbin |
| Plectin |
| PP1 |
| Preso1 |
| PSD93 |
| PSD95 |
| SAP102 |
| SAP97 |
| SAPAP1 |
| SAPAP2 |
| SAPAP3 |
| SAPAP4 |
| Shank1 |
| Shank2 |
| Shank3 |
| SNIP |
| Spinophilin |
| Stargazin |
| SynGAP |
| SynGAP |
| Tamalin |
| TANC2 |
| TANK |
| TARP |
| Yotiao |
| VDAC1 |
| α-Actinin |
| α-Catenin |
| α-Tubulin |
| β-Tubulin |

## Macroregions

| **Macroregion** | **Microregion** |
| --- | --- |
| Associative Cortex | Parietal, Occipital, primary visual cortex, Superior Temporal gyrus |
| Cerebellum | Cerebellum |
| Cingulate Cortex | cg1, cg2, ACC, PCC, Retrosplenial gyrus |
| Diencephalon | Thalamus, Hypothalamus, Habenula |
| Dorsal Striatum | Caudate, Putamen |
| Frontal Cortex | Frontal cortex, PFC, mPFC, DMPFC, DLPFC, Orbitofrontal cortex, Prelimbic cortex |
| Hippocampus | CA, DG, indusium griseum, hippocampus |
| Limbic System | Entorhinal cortex, Piriform cortex, Dorsal endopiriform nucleus, Septal nuclei, Nucleus of the vertical limb of the diagonal band, Olfactory tubercle, Insular cortex |
| Neocortex | Neocortex |
| Sensory-Motor Areas | medial agranular cortex, SS, motor cortex, premotor cortex, supplementary motor area, fronto-parietal cortex, claustrum |
| Subcortical Areas | Subcortical regions not specified, VTA |
| Ventral Striatum | Nucleus Accumbens Core & Shell, Ventral Pallidum, Islands of Calleja |
| Whole Brain | Whole Brain |

Appendix 3

## PRISMA flow diagram


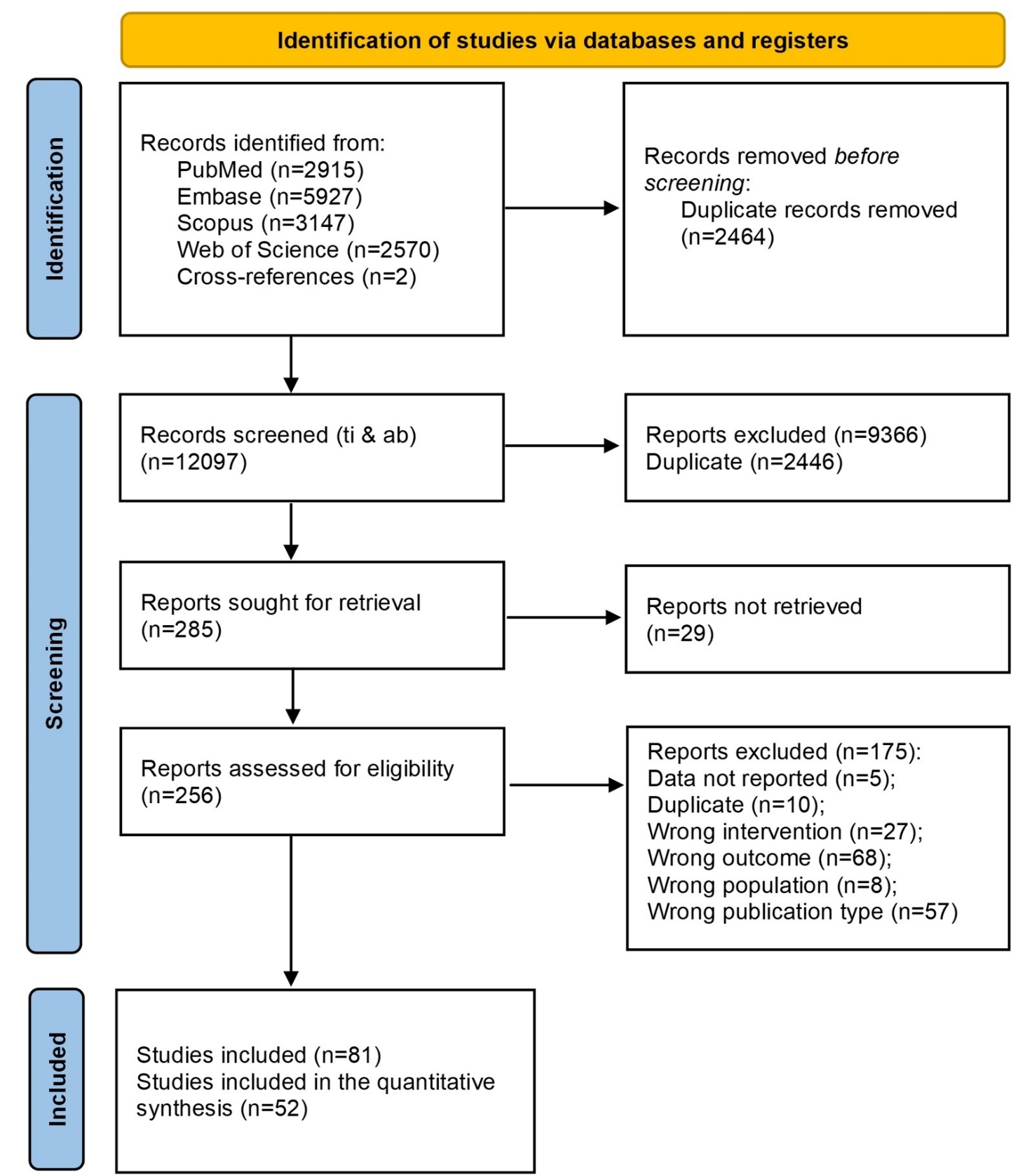


## Overview of included studies

| **Overview of included studies** | | | | | | | | |
| --- | --- | --- | --- | --- | --- | --- | --- | --- |
| **author** | **year** | **country** | **animal type** | **drug type** | **drug administration route** | **paradigm of administration** | **lab techniques** | **SYRCLE TOT** |
| Ambesi-Impiombato^1^ | 2007 | Italy | Sprague-Dawley rats | haloperidol, quetiapine | intraperitoneal | Acute, Chronic | ISH | 6 |
| Barone^2^ | 2021 | Italy | Sprague-Dawley rats | haloperidol | intraperitoneal | Acute | ISH | 6 |
| Barone^3^ | 2023 | Italy | Sprague-Dawley rats | asenapine | intraperitoneal | Acute | ISH | 7 |
| Baturina^4^ | 2020 | Russian federation | Wistar rats | haloperidol, risperidone | intraperitoneal | Chronic | ELISA | 3 |
| Brené^5^ | 1998 | USA | Sprague-Dawley rats | haloperidol | oral | Chronic | ISH | 4 |
| Bruins Slot^6^ | 2009 | France | Sprague-Dawley rats | haloperidol, clozapine | intraperitoneal | Acute | RT-PCR | 7 |
| Buck^7^ | 2024 | USA | mice | clozapine, haloperidol | subcutaneous | Acute | PCR | 6 |
| Buonaguro (a)^8^ | 2017 | Italy | Sprague-Dawley rats | asenapine, haloperidol, olanzapine | intraperitoneal | Chronic | ISH | 6 |
| Buonaguro (b)^9^ | 2017 | Italy | Sprague-Dawley rats | haloperidol | intraperitoneal | Acute | ISH | 6 |
| Chen^10^ | 1998 | UK | Sprague-Dawley rats | flupenthixol | oral | Chronic | MOSH | 4 |
| Chen^11^ | 1999 | UK | Sprague-Dawley rats | flupenthixol | oral | Chronic | MOSH, Western Blot | 6 |
| Chiba^12^ | 2006 | Japan | C57BL/6 | risperidone, haloperidol, clozapine, olanzapine | intraperitoneal | Chronic | RT-PCR | 5 |
| Choi^13^ | 2009 | USA | Sprague-Dawley rats | risperidone | intraperitoneal | Chronic | Autoradiography | 4 |
| Choi^14^ | 2017 | USA | Sprague-Dawley rats | cariprazine, aripiprazole | intraperitoneal | Chronic | Autoradiography | 4 |
| Collins^15^ | 2014 | UK | Sprague-Dawley rats | clozapine, Haloperidol | intraperitoneal | Acute, Chronic | Immunohistochemistry | 8 |
| de Bartolomeis^16^ | 2002 | Italy | Sprague-Dawley rats | olanzapine, haloperidol | intraperitoneal | Acute | ISH | 6 |
| de Bartolomeis^17^ | 2013 | Italy | Sprague-Dawley rats | haloperidol, amisulpride | intraperitoneal | Acute | ISH | 5 |
| de Bartolomeis^18^ | 2015 | Italy | Sprague-Dawley rats | asenapine, olanzapine, haloperidol | intraperitoneal | Acute | ISH | 6 |
| de Bartolomeis^19^ | 2016 | Italy | Sprague-Dawley rats | haloperidol, amisulpride | intraperitoneal | Chronic | ISH | 7 |
| de Bartolomeis^20^ | 2018 | Italy | Sprague-Dawley rats | haloperidol | intraperitoneal | Acute | Western Blot | 6 |
| Dell'Aversano^21^ | 2009 | Italy | Sprague-Dawley rats | haloperidol | intraperitoneal | Acute | ISH | 6 |
| Dutra-Tavares^22^ | 2023 | Brazil | C57BL/6 | olanzapine | subcutaneous | Acute | Western Blot | 6 |
| Eastwood^23^ | 1994 | UK | Sprague-Dawley rats | haloperidol | intraperitoneal | Chronic | ISH | 4 |
| Eastwood^24^ | 1996 | UK | Sprague-Dawley rats | haloperidol decanoate | intramuscolar | Chronic | ISH | 3 |
| Fatemi^25^ | 2006 | Japan | Sprague-Dawley rats | olanzapine | intraperitoneal | Chronic | RT-PCR, Western Blot | 7 |
| Fehér^26^ | 2005 | Hungary | Sprague-Dawley rats | haloperidol | intraperitoneal | Acute | RT-PCR | 4 |
| Fitzgerald^27^ | 1995 | USA | Sprague-Dawley rats | haloperidol, clozapine | intraperitoneal | Chronic | Western Blot | 6 |
| Fitzgerald^28^ | 1996 | USA | Sprague-Dawley rats | haloperidol | intraperitoneal | Chronic | Western Blot | 7 |
| Fumagalli^29^ | 2008 | Italy | Sprague-Dawley rats | haloperidol, olanzapine | subcutaneous | Chronic | Western Blot | 6 |
| Fumagalli^30^ | 2009 | Italy | Sprague-Dawley rats | haloperidol, olanzapine, quetiapine | subcutaneous | Chronic, Acute | Autoradiography | 6 |
| Funk^31^ | 2017 | USA | Sprague-Dawley rats | haloperidol decanoate | intramuscolar | Chronic | RT-PCR | 4 |
| Girgenti^32^ | 2010 | USA | Sprague-Dawley rats | haloperidol decanoate, olanzapine palmoate | subcutaneous | Chronic | ISH | 5 |
| Gomes^33^ | 2014 | Brazil | C57BL/6 | clozapine | intraperitoneal | Chronic | RT-PCR | 7 |
| Håkansson^34^ | 2006 | Sweden | C57BL/6 | haloperidol | intraperitoneal | Acute | Western Blot | 3 |
| Hanaoka^35^ | 2003 | Japan | Wistar rats | haloperidol, clozapine | intraperitoneal | Chronic | Immunoblotting | 6 |
| Hattori^36^ | 2006 | Japan | C57BL/6 | haloperidol | intraperitoneal | Acute | Western Blot | 3 |
| Healy^37^ | 1997 | USA | Sprague-Dawley rats | haloperidol, clozapine | subcutaneous | Chronic | ISH | 6 |
| Hida^38^ | 2015 | Japan | ICR mice | blonanserin | oral | Acute | Western Blot | 8 |
| Hiraoka^39^ | 2010 | Japan | Wistar rats | haloperidol | subcutaneous | Acute | RT-PCR | 5 |
| Iasevoli (a)^40^ | 2010 | Italy | Sprague-Dawley rats | haloperidol, olanzapine, risperidone, sulpiride | intraperitoneal | Acute | ISH | 6 |
| Iasevoli (b)^41^ | 2010 | Italy | Sprague-Dawley rats | haloperidol, sertindole | subcutaneous | Acute, Chronic | ISH | 6 |
| Iasevoli^42^ | 2011 | Italy | Sprague-Dawley rats | haloperidol, ziprasidone, clozapine | intraperitoneal | Chronic, Acute | ISH | 7 |
| Iasevoli^43^ | 2014 | Italy | Sprague-Dawley rats | haloperidol | intraperitoneal | Chronic | ISH | 8 |
| Iasevoli^44^ | 2020 | Italy | Sprague-Dawley rats | asenapine, haloperidol, olanzapine | intraperitoneal | Acute, Chronic | ISH | 8 |
| Kao^45^ | 2018 | UK | Sprague-Dawley rats | olanzapine | intraperitoneal | Chronic | Immunoblotting, ISH | 5 |
| Korlatowicz^46^ | 2021 | Poland | C57BL/6 | haloperidol, clozapine | intraperitoneal | Chronic, Acute | RT-PCR | 5 |
| Krzystanek^47^ | 2015 | Poland | Sprague-Dawley rats | haloperidol, olanzapine, clozapine | intraperitoneal | Chronic | Immunohistochemistry | 4 |
| Krzystanek^48^ | 2016 | Poland | Sprague-Dawley rats | haloperidol, olanzapine, clozapine | intraperitoneal | Chronic | Immunohistochemistry | 6 |
| Lan^49^ | 2024 | China | Sprague-Dawley rats | quetiapine | intraperitoneal | Chronic | Western Blot | 5 |
| Law^50^ | 2004 | UK | Sprague-Dawley rats | risperidone, haloperidol, olanzapine, chlorpromazine, clozapine | intraperitoneal | Chronic | ISH | 4 |
| Lidow^51^ | 2001 | USA | Rhesus Monkeys | haloperidol | oral | Chronic | Slot blot | 6 |
| Liu^52^ | 2022 | China | Sprague-Dawley rats | clozapine | intragastrical | Chronic | Western Blot | 4 |
| Lum^53^ | 2018 | Australia | Sprague-Dawley rats | haloperidol, aripiprazole | oral | Chronic | Western Blot | 5 |
| Luoni^54^ | 2014 | Italy | Sprague-Dawley rats | aripiprazole | intraperitoneal | Chronic | RT-PCR | 4 |
| Luoni^55^ | 2014 | Italy | Sprague-Dawley rats | lurasidone | oral | Chronic, Acute | RT-PCR | 4 |
| Mahmoud^56^ | 2021 | Egypt | Albino rats | olanzapine | oral | Chronic | Immunohistochemistry | 5 |
| Marchisella^57^ | 2020 | Italy | Sprague-Dawley rats | blonanserin | oral | Chronic | RT-PCR | 6 |
| Matosin^58^ | 2015 | Australia | Sprague-Dawley rats | haloperidol, olanzapine | oral | Chronic | Immunoblotting | 5 |
| Matsumoto^59^ | 2017 | Japan | Mice transgenic | clozapine | intraperitoneal | Acute | Western Blot | 6 |
| Mavrikaki^60^ | 2014 | Greece | Sprague-Dawley rats | aripiprazole | intraperitoneal | Acute, Chronic | Immunoblotting | 5 |
| McCullumsmith^61^ | 2003 | USA | Sprague-Dawley rats | haloperidol, olanzapine, clozapine | subcutaneous | Chronic | ISH | 4 |
| Meshul^62^ | 1996 | USA | Sprague-Dawley rats | haloperidol, clozapine | subcutaneous | Chronic | ISH | 7 |
| Nakahara^63^ | 2000 | Japan | Wistar rats | haloperidol, clozapine | intraperitoneal | Acute | RT-PCR | 4 |
| O'Connor^64^ | 2006 | USA | Rhesus Monkeys | haloperidol, clozapine | oral | Chronic | Western Blot | 5 |
| Orsetti^65^ | 2009 | Italy | Albino rats | quetiapine | intraperitoneal | Chronic | RT-PCR | 6 |
| Pan^66^ | 2016 | Australia | Sprague-Dawley rats | haloperidol, aripiprazole | oral | Chronic | Western Blot | 6 |
| Pei^67^ | 2003 | UK | Sprague-Dawley rats | chlorpromazine | intraperitoneal | Chronic | ISH | 5 |
| Polese^68^ | 2002 | Italy | Sprague-Dawley rats | haloperidol, clozapine | intraperitoneal | Acute | ISH | 8 |
| Riva^69^ | 1997 | Italy | Sprague-Dawley rats | haloperidol, clozapine | intraperitoneal | Acute, Chronic | Autoradiography | 4 |
| Robbins^70^ | 2008 | UK | Charles River rats | clozapine, haloperidol | intraperitoneal | Acute | RT-PCR | 6 |
| Sakuma^71^ | 2015 | Japan | ICR mice | aripiprazole, haloperidol, olanzapine, clozapine | oral | Acute | RT-PCR | 6 |
| Schmitt^72^ | 2003 | Germany | Sprague-Dawley rats | haloperidol, clozapine | oral | Chronic | ISH | 4 |
| Segnitz^73^ | 2011 | Germany | Sprague-Dawley rats | aripiprazole | oral | Chronic | ISH | 5 |
| Seo^74^ | 2015 | Korea | Sprague-Dawley rats | haloperidol, aripiprazole, olanzapine | intraperitoneal | Chronic | Western Blot | 5 |
| Stan^75^ | 2015 | Sweden | C57BL/6 | lurasidone | oral | Chronic | Western Blot | 8 |
| Tascedda^76^ | 2001 | Italy | Sprague-Dawley rats | olanzapine, haloperidol, clozapine | subcutaneous | Chronic | Autoradiography | 5 |
| Tomasetti^77^ | 2007 | Italy | Sprague-Dawley rats | haloperidol, aripiprazole, clozapine | intraperitoneal | Acute, Chronic | ISH | 8 |
| Tomasetti^78^ | 2011 | Italy | Sprague-Dawley rats | haloperidol, quetiapine | intraperitoneal | Acute, Chronic | ISH | 9 |
| Waters^79^ | 2014 | Sweden | Sprague-Dawley rats | aripiprazole, haloperidol | subcutaneous | Acute | RT-PCR | 6 |
| Zhang^80^ | 2014 | China | Sprague-Dawley rats | haloperidol, olanzapine | intraperitoneal | Acute | Western Blot | 9 |
| Zhou^81^ | 2020 | China | Sprague-Dawley rats | clozapine | subcutaneous | Chronic | Western Blot | 4 |

## Risk of bias

| **Quality assessment (SYRCLE)** | | | | | | | | | | | | |
| --- | --- | --- | --- | --- | --- | --- | --- | --- | --- | --- | --- | --- |
| **author** | **year** | **allocation (randomization)** | **groups' similarity** | **allocation concealement** | **random housing** | **blinding of the investigators** | **random outcome assessment** | **blinding of the outcome assessor** | **incomplete outcome data** | **selective outcome reporting** | **other sources of bias** | **TOT** |
| Ambesi-Impiombato^1^ | 2007 | Yes | Yes | Yes | Unclear | Unclear | Yes | Yes | No | Yes | No | 6 |
| Barone^2^ | 2021 | Yes | Yes | Yes | Unclear | No | Yes | No | Yes | Yes | Unclear | 6 |
| Barone^3^ | 2023 | Yes | Yes | Yes | Yes | No | Yes | No | Yes | Yes | Unclear | 7 |
| Baturina^4^ | 2020 | Yes | Yes | No | No | No | No | No | No | Yes | No | 3 |
| Brené^5^ | 1998 | Yes | Yes | Yes | Unclear | No | No | No | No | Yes | No | 4 |
| Bruins Slot^6^ | 2009 | Yes | Yes | Yes | Yes | No | Yes | No | Yes | Yes | No | 7 |
| Buck^7^ | 2024 | Yes | Yes | Yes | Unclear | Unclear | Unclear | Unclear | Yes | Yes | Yes | 6 |
| Buonaguro (a)^8^ | 2017 | Yes | Yes | Yes | Yes | No | Yes | No | No | Yes | No | 6 |
| Buonaguro (b)^9^ | 2017 | Yes | Yes | Yes | Unclear | No | Yes | No | Yes | Yes | No | 6 |
| Chen^10^ | 1998 | Yes | Yes | Yes | Unclear | No | No | No | No | Yes | Unclear | 4 |
| Chen^11^ | 1999 | Yes | Yes | Yes | Yes | No | Unclear | No | Yes | Yes | Unclear | 6 |
| Chiba^12^ | 2006 | Yes | Yes | No | No | No | Yes | No | Yes | Yes | Unclear | 5 |
| Choi^13^ | 2009 | Yes | Yes | No | No | No | No | No | Yes | Yes | Unclear | 4 |
| Choi^14^ | 2017 | Yes | Yes | Yes | Unclear | No | Unclear | No | No | Yes | No | 4 |
| Collins^15^ | 2014 | Yes | Yes | Yes | Yes | No | Yes | Yes | Yes | Yes | Unclear | 8 |
| de Bartolomeis^16^ | 2002 | Yes | Yes | Yes | Yes | No | Unclear | No | Yes | Yes | Unclear | 6 |
| de Bartolomeis^17^ | 2013 | Yes | Yes | Yes | No | No | No | No | Yes | Yes | Unclear | 5 |
| de Bartolomeis^18^ | 2015 | Yes | Yes | Yes | Unclear | No | Yes | No | Yes | Yes | Unclear | 6 |
| de Bartolomeis^19^ | 2016 | Yes | Yes | Yes | Unclear | No | Yes | No | Yes | Yes | Yes | 7 |
| de Bartolomeis^20^ | 2018 | Yes | Yes | Yes | Unclear | No | Yes | No | No | Yes | Yes | 6 |
| Dell'Aversano^21^ | 2009 | Yes | Yes | Yes | Yes | No | No | No | Yes | Yes | No | 6 |
| Dutra-Tavares^22^ | 2023 | Yes | Yes | Yes | Unclear | No | Unclear | No | Yes | Yes | Yes | 6 |
| Eastwood^23^ | 1994 | Yes | Yes | No | No | No | Yes | No | Unclear | Yes | Unclear | 4 |
| Eastwood^24^ | 1996 | Yes | Yes | No | No | No | No | No | No | Yes | Unclear | 3 |
| Fatemi^25^ | 2006 | Yes | Yes | Yes | Yes | No | Yes | No | Yes | Yes | No | 7 |
| Fehér^26^ | 2005 | Yes | Yes | No | Unclear | No | Yes | No | No | Yes | Unclear | 4 |
| Fitzgerald^27^ | 1995 | Yes | Yes | Yes | Yes | No | Unclear | No | Yes | Yes | No | 6 |
| Fitzgerald^28^ | 1996 | Yes | Yes | Yes | Yes | No | Yes | No | Yes | Yes | Unclear | 7 |
| Fumagalli^29^ | 2008 | Yes | Yes | Yes | Unclear | No | No | No | Yes | Yes | Yes | 6 |
| Fumagalli^30^ | 2009 | Yes | Yes | Yes | Yes | No | No | No | Yes | Yes | No | 6 |
| Funk^31^ | 2017 | Yes | Yes | No | No | No | No | No | Unclear | Yes | Yes | 4 |
| Girgenti^32^ | 2010 | Yes | Yes | Yes | Yes | No | No | No | No | Yes | No | 5 |
| Gomes^33^ | 2014 | Yes | Yes | Yes | Yes | Unclear | Unclear | Yes | Yes | Yes | No | 7 |
| Håkansson^34^ | 2006 | Yes | Yes | No | No | No | No | No | No | Yes | No | 3 |
| Hanaoka^35^ | 2003 | Yes | Yes | Yes | Yes | No | Unclear | No | No | Yes | Yes | 6 |
| Hattori^36^ | 2006 | Yes | Yes | Unclear | Unclear | No | No | No | Unclear | Yes | No | 3 |
| Healy^37^ | 1997 | Yes | Yes | Yes | Yes | No | No | No | Unclear | Yes | Yes | 6 |
| Hida^38^ | 2015 | Yes | Yes | Yes | Yes | Yes | Unclear | Yes | Yes | Yes | No | 8 |
| Hiraoka^39^ | 2010 | Yes | Yes | Unclear | Unclear | No | Yes | No | Yes | Yes | No | 5 |
| Iasevoli (a)^40^ | 2010 | Yes | Yes | Yes | Yes | Unclear | Yes | Unclear | Unclear | Yes | Unclear | 6 |
| Iasevoli (b)^41^ | 2010 | Yes | Yes | Yes | Yes | Unclear | Yes | Unclear | Unclear | Yes | Unclear | 6 |
| Iasevoli^42^ | 2011 | Yes | Yes | Yes | Unclear | No | Yes | Yes | Yes | Yes | Unclear | 7 |
| Iasevoli^43^ | 2014 | Yes | Yes | Yes | Yes | Yes | Yes | Yes | Unclear | Yes | Unclear | 8 |
| Iasevoli^44^ | 2020 | Yes | Yes | Yes | Yes | Yes | Yes | Yes | Unclear | Yes | Unclear | 8 |
| Kao^45^ | 2018 | Yes | Yes | Yes | Yes | No | Unclear | No | No | Yes | Unclear | 5 |
| Korlatowicz^46^ | 2021 | Yes | Yes | Yes | Yes | Unclear | Unclear | No | No | Yes | No | 5 |
| Krzystanek^47^ | 2015 | Yes | Yes | Yes | Unclear | Unclear | Unclear | Unclear | Unclear | Yes | No | 4 |
| Krzystanek^48^ | 2016 | Yes | Yes | Yes | Yes | Unclear | Yes | Unclear | Unclear | Yes | No | 6 |
| Lan^49^ | 2024 | Unclear | Yes | Yes | Unclear | Unclear | Unclear | Unclear | Yes | Yes | Yes | 5 |
| Law^50^ | 2004 | Yes | Yes | Yes | Yes | Unclear | Unclear | Unclear | Unclear | Yes | No | 4 |
| Lidow^51^ | 2001 | Yes | Yes | Unclear | Yes | Unclear | Yes | Unclear | Yes | Yes | No | 6 |
| Liu^52^ | 2022 | Yes | Yes | Unclear | Yes | Unclear | Unclear | Unclear | No | Yes | Unclear | 4 |
| Lum^53^ | 2018 | Yes | Yes | Yes | Yes | No | Unclear | Unclear | No | Yes | No | 5 |
| Luoni^54^ | 2014 | Yes | Yes | Yes | Unclear | Unclear | Unclear | Unclear | Unclear | Yes | No | 4 |
| Luoni^55^ | 2014 | Yes | Yes | Yes | Unclear | Unclear | Unclear | Unclear | Unclear | Yes | No | 4 |
| Mahmoud^56^ | 2021 | Yes | Yes | Yes | Unclear | Unclear | Unclear | Unclear | Yes | Yes | No | 5 |
| Marchisella^57^ | 2020 | Yes | Yes | Yes | Yes | Unclear | Yes | No | No | Yes | Unclear | 6 |
| Matosin^58^ | 2015 | Yes | Yes | Unclear | Unclear | Yes | Unclear | Yes | No | Yes | No | 5 |
| Matsumoto^59^ | 2017 | Yes | Yes | Unclear | Yes | Unclear | Yes | No | Unclear | Yes | Yes | 6 |
| Mavrikaki^60^ | 2014 | Yes | Yes | Yes | Unclear | No | Unclear | Unclear | Yes | Yes | No | 5 |
| McCullumsmith^61^ | 2003 | Yes | Yes | Yes | Unclear | No | Unclear | No | No | Yes | Unclear | 4 |
| Meshul^62^ | 1996 | Yes | Yes | Yes | Yes | Yes | Unclear | Yes | No | Yes | No | 7 |
| Nakahara^63^ | 2000 | Yes | Yes | Yes | Unclear | Unclear | Unclear | No | No | Yes | No | 4 |
| O'Connor^64^ | 2006 | Yes | Yes | Unclear | Yes | Unclear | Yes | No | No | Yes | No | 5 |
| Orsetti^65^ | 2009 | Yes | Yes | Yes | Yes | No | Yes | Unclear | Unclear | Yes | Unclear | 6 |
| Pan^66^ | 2016 | Yes | Yes | Yes | Yes | Unclear | Unclear | Unclear | Unclear | Yes | Yes | 6 |
| Pei^67^ | 2003 | Yes | Yes | Unclear | Unclear | Yes | Unclear | Yes | Unclear | Yes | No | 5 |
| Polese^68^ | 2002 | Yes | Yes | Yes | Yes | Yes | Yes | Yes | Unclear | Yes | No | 8 |
| Riva^69^ | 1997 | Yes | Yes | Yes | Unclear | Unclear | Unclear | Unclear | Unclear | Yes | No | 4 |
| Robbins^70^ | 2008 | Yes | Yes | Yes | Yes | Unclear | Yes | No | Unclear | Yes | No | 6 |
| Sakuma^71^ | 2015 | Yes | Yes | Yes | Yes | Unclear | Yes | No | No | Yes | No | 6 |
| Schmitt^72^ | 2003 | Yes | Yes | Yes | Unclear | No | No | No | Unclear | Yes | No | 4 |
| Segnitz^73^ | 2011 | Yes | Yes | Yes | Yes | Unclear | Unclear | Unclear | Unclear | Yes | No | 5 |
| Seo^74^ | 2015 | Yes | Yes | Yes | Yes | No | Unclear | No | No | Yes | No | 5 |
| Stan^75^ | 2015 | Yes | Yes | Yes | Yes | Yes | Yes | Yes | Unclear | Yes | No | 8 |
| Tascedda^76^ | 2001 | Yes | Yes | Yes | Unclear | Unclear | Unclear | Unclear | Yes | Yes | Unclear | 5 |
| Tomasetti^77^ | 2007 | Yes | Yes | Yes | Yes | Yes | Yes | Yes | Unclear | Yes | No | 8 |
| Tomasetti^78^ | 2011 | Yes | Yes | Yes | Yes | Yes | Yes | Yes | No | Yes | Yes | 9 |
| Waters^79^ | 2014 | Yes | Yes | Yes | Yes | Unclear | Yes | No | Unclear | Yes | No | 6 |
| Zhang^80^ | 2014 | Yes | Yes | Yes | Yes | Yes | Yes | Yes | Unclear | Yes | Yes | 9 |
| Zhou^81^ | 2020 | Yes | Yes | Yes | Unclear | Unclear | Unclear | Unclear | Unclear | Yes | No | 4 |

## CAMARADES Checklist

| **CAMARADES Checklist** | | | | | | | | | | | | |
| --- | --- | --- | --- | --- | --- | --- | --- | --- | --- | --- | --- | --- |
| **author** | **year** | **peer reviewed** | **control of temperature** | **allocation concealment** | **blinded treatment** | **blinded assessment of outcome** | **no psychoactive drug to induce death** | **animal model of psychosis** | **sample size calculation** | **compliance with animal welfare regulations** | **no potential conflict of interests** | **TOT** |
| Ambesi-Impiombato^1^ | 2007 | Yes | Yes | Yes | Unclear | Yes | Yes | No | No | Yes | Yes | 7 |
| Barone^2^ | 2021 | Yes | Yes | Yes | No | No | Yes | No | No | Yes | Yes | 6 |
| Barone^3^ | 2023 | Yes | Yes | Yes | No | No | Yes | Yes | No | Yes | Yes | 7 |
| Baturina^4^ | 2020 | Yes | Unclear | No | No | No | Yes | No | No | Yes | Yes | 4 |
| Brené^5^ | 1998 | Yes | Unclear | Yes | No | No | Yes | No | No | Yes | Unclear | 4 |
| Bruins Slot^6^ | 2009 | Yes | Yes | Yes | No | No | Yes | No | No | Yes | Unclear | 5 |
| Buck^7^ | 2024 | Yes | Unclear | Yes | Unclear | Unclear | Yes | Yes | No | Yes | Yes | 6 |
| Buonaguro (a)^8^ | 2017 | Yes | Yes | Yes | No | No | Yes | No | No | Yes | Yes | 6 |
| Buonaguro (b)^9^ | 2017 | Yes | Yes | Yes | No | No | Yes | Yes | No | Yes | Yes | 7 |
| Chen^10^ | 1998 | Yes | No | Yes | No | No | Yes | No | No | Unclear | Yes | 4 |
| Chen^11^ | 1999 | Yes | No | Yes | No | No | Yes | No | No | Unclear | Yes | 4 |
| Chiba^12^ | 2006 | Yes | Unclear | No | No | No | Yes | No | No | Yes | Yes | 4 |
| Choi^13^ | 2009 | Yes | Yes | No | No | No | Yes | No | No | Yes | Yes | 5 |
| Choi^14^ | 2017 | Yes | Yes | Yes | No | No | Yes | No | No | Yes | Yes | 6 |
| Collins^15^ | 2014 | Yes | Yes | Yes | No | Yes | No | No | No | Yes | Yes | 6 |
| de Bartolomeis^16^ | 2002 | Yes | Yes | Yes | No | No | Yes | No | No | Yes | Yes | 6 |
| de Bartolomeis^17^ | 2013 | Yes | Yes | Yes | No | No | Yes | No | No | Yes | Yes | 6 |
| de Bartolomeis^18^ | 2015 | Yes | Yes | Yes | No | No | Yes | No | No | Yes | Yes | 6 |
| de Bartolomeis^19^ | 2016 | Yes | Yes | Yes | No | No | Yes | No | No | Yes | Yes | 6 |
| de Bartolomeis^20^ | 2018 | Yes | Yes | Yes | No | No | Yes | No | No | Yes | Yes | 6 |
| Dell'Aversano^21^ | 2009 | Yes | Yes | Yes | No | No | Yes | No | No | Yes | Yes | 6 |
| Dutra-Tavares^22^ | 2023 | Yes | Unclear | Yes | No | No | Yes | Yes | No | Yes | Unclear | 5 |
| Eastwood^23^ | 1994 | Yes | Unclear | No | No | No | No | No | No | Unclear | Unclear | 1 |
| Eastwood^24^ | 1996 | Yes | Unclear | No | No | No | Yes | No | No | Unclear | Unclear | 2 |
| Fatemi^25^ | 2006 | Yes | Yes | Yes | No | No | No | No | No | Yes | Yes | 5 |
| Fehér^26^ | 2005 | Yes | Unclear | No | No | No | Yes | No | No | Yes | Yes | 4 |
| Fitzgerald^27^ | 1995 | Yes | Unclear | Yes | No | No | Yes | No | No | Unclear | Unclear | 3 |
| Fitzgerald^28^ | 1996 | Yes | Unclear | Yes | No | No | Yes | No | No | Unclear | Unclear | 3 |
| Fumagalli^29^ | 2008 | Yes | Unclear | Yes | No | No | Yes | No | No | Yes | Yes | 5 |
| Fumagalli^30^ | 2009 | Yes | Unclear | Yes | No | No | Yes | No | No | Yes | Yes | 5 |
| Funk^31^ | 2017 | Yes | Unclear | No | No | No | Yes | No | No | Yes | Yes | 4 |
| Girgenti^32^ | 2010 | Yes | Unclear | Yes | No | No | Yes | No | No | Yes | Yes | 5 |
| Gomes^33^ | 2014 | Yes | Yes | Yes | Unclear | Yes | Yes | Yes | No | Yes | Yes | 8 |
| Håkansson^34^ | 2006 | Yes | Unclear | No | No | No | Yes | Yes | No | Yes | Unclear | 4 |
| Hanaoka^35^ | 2003 | Yes | Yes | Yes | No | No | Yes | No | No | Unclear | Yes | 5 |
| Hattori^36^ | 2006 | Yes | Yes | Unclear | No | No | Unclear | Yes | No | Yes | Unclear | 4 |
| Healy^37^ | 1997 | Yes | Unclear | Yes | No | No | Yes | No | No | Yes | Yes | 5 |
| Hida^38^ | 2015 | Yes | Yes | Yes | Yes | Yes | Yes | Yes | No | Yes | Yes | 9 |
| Hiraoka^39^ | 2010 | Yes | Unclear | Unclear | No | No | Unclear | No | No | Yes | Yes | 3 |
| Iasevoli (a)^40^ | 2010 | Yes | Yes | Yes | Unclear | Unclear | Yes | No | No | Yes | Yes | 6 |
| Iasevoli (b)^41^ | 2010 | Yes | Yes | Yes | Unclear | Unclear | Yes | No | No | Yes | Yes | 6 |
| Iasevoli^42^ | 2011 | Yes | Yes | Yes | No | Yes | Yes | No | No | Yes | Yes | 7 |
| Iasevoli^43^ | 2014 | Yes | Yes | Yes | Yes | Yes | Yes | No | No | Yes | Yes | 8 |
| Iasevoli^44^ | 2020 | Yes | Yes | Yes | Yes | Yes | Yes | No | No | Yes | Yes | 6 |
| Kao^45^ | 2018 | Yes | Yes | Yes | No | No | Yes | No | No | Yes | Unclear | 5 |
| Korlatowicz^46^ | 2021 | Yes | Yes | Yes | Unclear | No | Yes | Yes | No | Yes | Yes | 7 |
| Krzystanek^47^ | 2015 | Yes | Yes | Yes | Unclear | Unclear | No | No | No | Unclear | Yes | 4 |
| Krzystanek^48^ | 2016 | Yes | Yes | Yes | Unclear | Unclear | No | No | No | Unclear | Yes | 4 |
| Lan^49^ | 2024 | Yes | Yes | Yes | Unclear | Unclear | Unclear | Yes | No | Yes | Yes | 6 |
| Law^50^ | 2004 | Yes | Unclear | Yes | Unclear | Unclear | No | No | No | Yes | No | 3 |
| Lidow^51^ | 2001 | Yes | Unclear | Unclear | Unclear | Unclear | No | No | No | Yes | Yes | 3 |
| Liu^52^ | 2022 | Yes | Yes | Unclear | Unclear | Unclear | No | Yes | No | Yes | Yes | 5 |
| Lum^53^ | 2018 | Yes | Yes | Yes | No | Unclear | Yes | No | No | Yes | Yes | 6 |
| Luoni^54^ | 2014 | Yes | Yes | Yes | Unclear | Unclear | Unclear | No | No | Yes | Yes | 5 |
| Luoni^55^ | 2014 | Yes | Yes | Yes | Unclear | Unclear | Unclear | No | No | Yes | Yes | 5 |
| Mahmoud^56^ | 2021 | Yes | Yes | Yes | Unclear | Unclear | Unclear | Yes | No | Yes | Yes | 6 |
| Marchisella^57^ | 2020 | Yes | Yes | Yes | Unclear | No | Yes | No | No | Yes | Yes | 6 |
| Matosin^58^ | 2015 | Yes | Yes | Unclear | Yes | Yes | Yes | No | No | Yes | Yes | 7 |
| Matsumoto^59^ | 2017 | Yes | Unclear | Unclear | Unclear | No | Unclear | Yes | No | Yes | Yes | 4 |
| Mavrikaki^60^ | 2014 | Yes | Unclear | Yes | No | Unclear | Yes | No | No | Yes | Yes | 5 |
| McCullumsmith^61^ | 2003 | Yes | Unclear | Yes | No | No | Yes | No | No | Unclear | Yes | 4 |
| Meshul^62^ | 1996 | Yes | Unclear | Yes | Yes | Yes | Yes | No | No | Yes | Yes | 7 |
| Nakahara^63^ | 2000 | Yes | Unclear | Yes | Unclear | No | Yes | No | No | Yes | Unclear | 4 |
| O'Connor^64^ | 2006 | Yes | Unclear | Unclear | Unclear | No | No | No | No | Yes | Yes | 3 |
| Orsetti^65^ | 2009 | Yes | Yes | Yes | No | Unclear | Yes | Yes | No | Yes | Unclear | 5 |
| Pan^66^ | 2016 | Yes | Yes | Yes | Unclear | Unclear | Yes | No | No | Yes | Yes | 6 |
| Pei^67^ | 2003 | Yes | Yes | Unclear | Yes | Yes | No | No | No | Yes | Yes | 6 |
| Polese^68^ | 2002 | Yes | Yes | Yes | Yes | Yes | Yes | No | No | Yes | Yes | 8 |
| Riva^69^ | 1997 | Yes | Unclear | Yes | Unclear | Unclear | Yes | No | No | Unclear | Yes | 4 |
| Robbins^70^ | 2008 | Yes | Yes | Yes | Unclear | No | Yes | No | No | Yes | Unclear | 5 |
| Sakuma^71^ | 2015 | Yes | Yes | Yes | Unclear | No | Yes | No | No | Yes | Unclear | 5 |
| Schmitt^72^ | 2003 | Yes | Unclear | Yes | No | No | Yes | No | No | Yes | Unclear | 4 |
| Segnitz^73^ | 2011 | Yes | Unclear | Yes | Unclear | Unclear | Yes | No | No | Yes | Yes | 5 |
| Seo^74^ | 2015 | Yes | Yes | Yes | No | No | Yes | Yes | No | Yes | Yes | 6 |
| Stan^75^ | 2015 | Yes | Yes | Yes | Yes | Yes | Yes | No | No | Yes | Yes | 8 |
| Tascedda^76^ | 2001 | Yes | Unclear | Yes | Unclear | Unclear | Yes | No | No | Yes | Unclear | 4 |
| Tomasetti^77^ | 2007 | Yes | Yes | Yes | Yes | Yes | Yes | No | No | Yes | Yes | 8 |
| Tomasetti^78^ | 2011 | Yes | Yes | Yes | Yes | Yes | Yes | No | No | Yes | Yes | 8 |
| Waters^79^ | 2014 | Yes | Yes | Yes | Unclear | No | Yes | No | No | Yes | Yes | 6 |
| Zhang^80^ | 2014 | Yes | Yes | Yes | Yes | Yes | Unclear | Yes | No | Yes | Yes | 8 |
| Zhou^81^ | 2020 | Yes | Yes | Yes | Unclear | Unclear | No | Yes | No | Yes | Unclear | 5 |

Appendix 4

## Studies excluded

| **Studies excluded** | | |
| --- | --- | --- |
| **author** | **year** | **reasons for exclusion** |
| Abbas^82^ | 2009 | wrong outcome |
| Abiero^83^ | 2021 | wrong outcome |
| Adham^84^ | 2015 | wrong publication type |
| Ahn^85^ | 2002 | wrong interventation |
| Ahn^86^ | 2009 | wrong interventation |
| Ambesi-Impiombato^87^ | 2003 | duplicate |
| Amin^88^ | 2014 | wrong outcome |
| Amiri^89^ | 2020 | wrong interventation |
| Anastasio^90^ | 2008 | wrong outcome |
| Apam-Castillejos^91^ | 2022 | wrong outcome |
| Avagliano^92^ | 2018 | wrong publication type |
| Avvisati^93^ | 2013 | wrong publication type |
| Bardgett^94^ | 2006 | wrong outcome |
| Barr^95^ | 2006 | wrong outcome |
| Begni^96^ | 2019 | wrong publication type |
| Bektas^97^ | 2014 | wrong publication type |
| Bertran-Gonzalez^98^ | 2008 | wrong outcome |
| Bjarnadottir^99^ | 2007 | wrong interventation |
| Borrell^100^ | 2009 | wrong publication type |
| Boulay^101^ | 2011 | wrong publication type |
| Bragina^102^ | 2006 | wrong outcome |
| Bristow^103^ | 2021 | wrong publication type |
| Bristow^104^ | 2017 | wrong publication type |
| Buonaguro^105^ | 2012 | wrong publication type |
| Buonaguro^106^ | 2013 | wrong publication type |
| Buonaguro^107^ | 2014 | wrong publication type |
| Buonaguro^108^ | 2016 | wrong publication type |
| Buonaguro^109^ | 2016 | wrong publication type |
| Buonanno^110^ | 2017 | wrong publication type |
| Bustos^111^ | 2004 | wrong publication type |
| Castillo-Gómez^112^ | 2008 | wrong outcome |
| Chartoff^113^ | 1999 | wrong outcome |
| Chen^10^ | 1998 | duplicate |
| Chen^11^ | 1999 | duplicate |
| Chen^114^ | 2017 | wrong outcome |
| Chen^115^ | 2020 | wrong population |
| Chhabra^116^ | 2023 | wrong outcome |
| Chih-Hui Chen^10^ | 1998 | duplicate |
| Clapcote^117^ | 2010 | wrong publication type |
| Critchlow^118^ | 2006 | wrong population |
| de Bartolomeis^119^ | 2000 | wrong publication type |
| de Bartolomeis^120^ | 2004 | wrong publication type |
| de Bartolomeis^121^ | 2013 | wrong interventation |
| de Bartolomeis^122^ | 2015 | wrong interventation |
| de Bartolomeis^19^ | 2016 | wrong publication type |
| de Bartolomeis^123^ | 2019 | wrong publication type |
| de la Fuente Revenga^124^ | 2018 | wrong outcome |
| Diana^125^ | 2015 | wrong outcome |
| Eastwood^23^ | 1994 | duplicate |
| Eastwood^126^ | 1994 | duplicate |
| Eastwood^126^ | 1994 | wrong outcome |
| Eastwood^127^ | 1997 | wrong outcome |
| Eastwood^128^ | 2000 | wrong outcome |
| Engel^129^ | 2016 | wrong outcome |
| Engmann^130^ | 2016 | wrong outcome |
| Ertuĝrul^131^ | 2011 | wrong outcome |
| Fiore^132^ | 2003 | wrong publication type |
| Fitzgerald^27^ | 1995 | duplicate |
| Fosnaugh^133^ | 1995 | data not reported |
| Fry^134^ | 2020 | wrong outcome |
| Fu^135^ | 2004 | wrong interventation |
| Fujimura^136^ | 2000 | wrong outcome |
| Fukuyama^137^ | 2018 | wrong outcome |
| Gardoni^138^ | 2008 | data not reported |
| Gentzel^139^ | 2015 | wrong outcome |
| Gnegy^140^ | 1994 | wrong outcome |
| Guo^141^ | 2009 | wrong outcome |
| Halff^142^ | 2021 | wrong outcome |
| Hamid^143^ | 1998 | wrong outcome |
| Hara^144^ | 2009 | wrong publication type |
| Hashimoto^145^ | 1997 | wrong outcome |
| Healy^146^ | 1996 | wrong publication type |
| Hentschel^147^ | 2000 | wrong outcome |
| Hussain^148^ | 2001 | wrong outcome |
| Hussain^149^ | 2002 | wrong outcome |
| Iasevoli^150^ | 2005 | wrong publication type |
| Iasevoli^151^ | 2011 | wrong publication type |
| Iasevoli^152^ | 2012 | wrong publication type |
| Iasevoli^153^ | 2014 | wrong publication type |
| Inada^154^ | 2007 | wrong outcome |
| Ito^155^ | 2023 | wrong outcome |
| Jevtic^156^ | 2015 | wrong publication type |
| Johnson^157^ | 1998 | wrong outcome |
| Kabbani^158^ | 2006 | wrong outcome |
| Kálmán^159^ | 2010 | wrong interventation |
| Kalman Jr^160^ | 2010 | wrong publication type |
| Kessas^161^ | 2010 | wrong outcome |
| Kim^162^ | 2014 | wrong publication type |
| Klintzova^163^ | 1989 | wrong publication type |
| Kontkanen^164^ | 2000 | wrong interventation |
| Korostynski^165^ | 2012 | wrong publication type |
| Kosugi^166^ | 2010 | wrong publication type |
| Kruyer^167^ | 2021 | wrong outcome |
| Latte^168^ | 2016 | wrong publication type |
| Lee^169^ | 2003 | wrong outcome |
| Lee^170^ | 2007 | wrong outcome |
| Lee^171^ | 2020 | wrong interventation |
| Leggieri^172^ | 2022 | wrong interventation |
| Lévesque^173^ | 2017 | wrong outcome |
| Lidow^174^ | 2003 | wrong publication type |
| Lidsky^175^ | 1997 | wrong outcome |
| Liu^176^ | 2017 | wrong interventation |
| Loessner^177^ | 1988 | wrong population |
| MacDonald^178^ | 2017 | wrong publication type |
| Marchisella^179^ | 2019 | wrong publication type |
| Marin^180^ | 1997 | wrong outcome |
| Marmo^181^ | 2009 | wrong publication type |
| Marmo^182^ | 2014 | wrong publication type |
| Martin^183^ | 2015 | wrong outcome |
| McCoy^184^ | 1996 | wrong outcome |
| McCoy^185^ | 1998 | wrong population |
| McKerchar^186^ | 2002 | wrong publication type |
| McKerchar^186^ | 2002 | wrong publication type |
| McLeod^187^ | 2009 | wrong publication type |
| Meltzer^188^ | 2019 | wrong publication type |
| Meshul^189^ | 1989 | wrong outcome |
| Meshul^190^ | 1994 | wrong outcome |
| Meshul^191^ | 1994 | wrong outcome |
| Meshul^192^ | 1995 | wrong outcome |
| Meshul^193^ | 1996 | wrong outcome |
| Mielnik^194^ | 2015 | wrong publication type |
| Mishra^195^ | 2022 | wrong interventation |
| Mohn^196^ | 1999 | wrong outcome |
| Nakahara^197^ | 1998 | wrong outcome |
| Noda^38^ | 2014 | duplicate |
| Oda^198^ | 2017 | wrong interventation |
| O'Neill^199^ | 2018 | wrong interventation |
| Onimus^200^ | 2022 | wrong outcome |
| Onishi^201^ | 2018 | wrong outcome |
| Oretti^202^ | 1994 | data not reported |
| Ossowska^203^ | 1996 | wrong outcome |
| Ossowska^204^ | 1999 | wrong outcome |
| Oyagi^205^ | 2009 | wrong interventation |
| Oyagi^206^ | 2010 | wrong publication type |
| Ozdemir^207^ | 2012 | wrong outcome |
| Pan^208^ | 2023 | wrong interventation |
| Park^209^ | 2013 | wrong population |
| Pathak^210^ | 2015 | data not reported |
| Pei^211^ | 2004 | wrong interventation |
| Petronijevic^212^ | 2015 | wrong publication type |
| Picconi^213^ | 2004 | wrong interventation |
| Pillai-Nair^214^ | 2005 | wrong interventation |
| Pisano^215^ | 2021 | wrong publication type |
| Pizzagalli^216^ | 2012 | wrong publication type |
| Porteous^217^ | 2009 | wrong publication type |
| Purkayastha^218^ | 2012 | wrong population |
| Robinson^219^ | 2001 | wrong interventation |
| Rodriguez^220^ | 2010 | wrong interventation |
| Sanson^221^ | 2020 | wrong publication type |
| Santana^222^ | 2011 | wrong outcome |
| Scarr^223^ | 2012 | wrong outcome |
| Schalbetter^224^ | 2021 | wrong interventation |
| Schmitt^225^ | 2004 | wrong publication type |
| Schmitt^226^ | 2011 | wrong publication type |
| Schroeder^227^ | 2000 | data not reported |
| Sharp^228^ | 1994 | wrong interventation |
| Shen^229^ | 2018 | wrong outcome |
| Stan^230^ | 2012 | wrong publication type |
| Svane^231^ | 2018 | wrong outcome |
| Svenningsson^75^ | 2014 | duplicate |
| Tarazi^232^ | 2003 | wrong outcome |
| Toua^233^ | 2010 | duplicate |
| Toua^233^ | 2010 | wrong outcome |
| Uslaner^234^ | 2009 | wrong interventation |
| Vaisburd^235^ | 2015 | wrong outcome |
| Vellucci^236^ | 2020 | wrong publication type |
| Waters^237^ | 2011 | wrong publication type |
| Yabuki^238^ | 2013 | wrong interventation |
| Yabuki^239^ | 2019 | wrong interventation |
| Yanahashi^240^ | 2004 | wrong outcome |
| Yang^241^ | 2010 | wrong outcome |
| Yasugawa^242^ | 1991 | wrong population |
| Zhang^243^ | 2016 | wrong population |
| Zuo^244^ | 2009 | wrong outcome |
| Zygmunt^245^ | 2016 | wrong publication type |

Appendix 5

Antipsychotics retrieved

| antipsychotic | number of studies | percentage (%) on the total number of included studies (81) |
| --- | --- | --- |
| amisulpride | 2 | 2.47 |
| aripiprazole | 10 | 12.35 |
| asenapine | 4 | 4.94 |
| blonanserin | 2 | 2.47 |
| cariprazine | 1 | 1.23 |
| chlorpromazine | 2 | 2.47 |
| clozapine | 27 | 33.33 |
| flupenthixol | 2 | 2.47 |
| haloperidol | 55 | 67.9 |
| haloperidol decanoate | 3 | 3.7 |
| lurasidone | 2 | 2.47 |
| olanzapine | 21 | 25.93 |
| olanzapine palmoate | 1 | 1.23 |
| quetiapine | 5 | 6.17 |
| risperidone | 5 | 6.17 |
| sertindole | 1 | 1.23 |
| sulpiride | 1 | 1.23 |
| ziprasidone | 1 | 1.23 |

PSD molecules retrieved

| PSD molecule | number of studies | percentage (%) on the total number of included studies (81) |
| --- | --- | --- |
| AMPAR | 2 | 2.47 |
| Ania3 | 5 | 6.17 |
| Arc | 18 | 22.22 |
| B-Actin | 1 | 1.23 |
| CaMKII | 4 | 4.94 |
| CaMKII pThr286 | 2 | 2.47 |
| CaMKIIp | 1 | 1.23 |
| DISC1 | 1 | 1.23 |
| GluA1 | 10 | 12.35 |
| GluA1 pSer831 | 3 | 3.7 |
| GluA1 pSer845 | 3 | 3.7 |
| GluA2 | 9 | 11.11 |
| GluA2_3 | 1 | 1.23 |
| GluA3 | 3 | 3.7 |
| GluA4 | 3 | 3.7 |
| GluK1 | 2 | 2.47 |
| GluK2 | 2 | 2.47 |
| GluK3 | 2 | 2.47 |
| GluR6-7 | 1 | 1.23 |
| Homer1 | 2 | 2.47 |
| Homer1a | 20 | 24.69 |
| Homer1b-c | 8 | 9.88 |
| Homer2 | 1 | 1.23 |
| KA1 | 2 | 2.47 |
| KA2 | 2 | 2.47 |
| mGlur1 | 3 | 3.7 |
| mGlur2 | 2 | 2.47 |
| mGlur3 | 2 | 2.47 |
| mGlur4 | 1 | 1.23 |
| mGlur5 | 5 | 6.17 |
| mGlur7 | 1 | 1.23 |
| NMDAR | 2 | 2.47 |
| Norbin | 3 | 3.7 |
| NR1 | 28 | 34.57 |
| NR1 pSer896 | 3 | 3.7 |
| NR1 pSer897 | 3 | 3.7 |
| NR2A | 15 | 18.52 |
| NR2A pSer1232 | 1 | 1.23 |
| NR2B | 16 | 19.75 |
| NR2B pSer1303 | 3 | 3.7 |
| NR2B pSer1472 | 1 | 1.23 |
| NR2B pTyr1472 | 2 | 2.47 |
| NR2C | 6 | 7.41 |
| NR2D | 3 | 3.7 |
| Preso1 | 1 | 1.23 |
| PSD95 | 14 | 17.28 |
| SAP102 | 1 | 1.23 |
| SAP97 | 1 | 1.23 |
| Shank1 | 1 | 1.23 |
| Spinophilin | 3 | 3.7 |
| Tamalin | 1 | 1.23 |
| Yotiao | 1 | 1.23 |

## Results

| **Results** | | | | | | | | | | | | | | |
| --- | --- | --- | --- | --- | --- | --- | --- | --- | --- | --- | --- | --- | --- | --- |
| **drug** | **model** | **psd type** | **region** | **outcome type** | **n_studies** | **n_group_1** | **n_group_2** | **beta** | **CI** | **p_val** | **PI** | **i_sq** | **tau_sq** | **q_p_val** |
| amisulpride | chronic | arc | cingulate cortex | mrna levels | 2 | 12 | 12 | 0.22 | -0.583, 1.024 | 0.59115941 | -0.583, 1.024 | 0 | 0 | 0.7398 |
| amisulpride | chronic | arc | dorsal striatum | mrna levels | 2 | 12 | 12 | 1.235 | 0.359, 2.111 | 0.00573881 | 0.359, 2.111 | 0 | 0 | 0.5518 |
| amisulpride | chronic | arc | limbic system | mrna levels | 2 | 12 | 12 | 0.454 | -0.485, 1.393 | 0.34349765 | -0.684, 1.592 | 23.38 | 0.11 | 0.2533 |
| amisulpride | chronic | arc | sensory-motor areas | mrna levels | 2 | 12 | 12 | 0.12 | -0.685, 0.925 | 0.77032363 | -0.685, 0.925 | 0 | 0 | 0.476 |
| amisulpride | chronic | arc | ventral striatum | mrna levels | 2 | 12 | 12 | 0.825 | -0.01, 1.66 | 0.05270135 | -0.01, 1.66 | 0 | 0 | 0.6841 |
| amisulpride | chronic | homer1a | cingulate cortex | mrna levels | 2 | 12 | 12 | 1.355 | -1.757, 4.466 | 0.39347591 | -3.848, 6.557 | 89.66 | 4.53 | 0.0019 |
| amisulpride | chronic | homer1a | dorsal striatum | mrna levels | 2 | 12 | 12 | 1.804 | -0.412, 4.02 | 0.11065992 | -1.764, 5.372 | 79.2 | 2.04 | 0.0283 |
| amisulpride | chronic | homer1a | limbic system | mrna levels | 2 | 12 | 12 | 2.678 | -3.045, 8.401 | 0.3590189 | -7.041, 12.397 | 94.06 | 16.06 | 0 |
| amisulpride | chronic | homer1a | sensory-motor areas | mrna levels | 2 | 12 | 12 | 0.575 | -2.134, 3.285 | 0.67732768 | -3.94, 5.091 | 88.81 | 3.4 | 0.0028 |
| amisulpride | chronic | homer1a | ventral striatum | mrna levels | 2 | 12 | 12 | 0.982 | -0.103, 2.067 | 0.07613854 | -0.449, 2.413 | 36.76 | 0.23 | 0.2086 |
| amisulpride | chronic | homer1b-c | cingulate cortex | mrna levels | 2 | 12 | 12 | 0.189 | -1.742, 2.12 | 0.84805566 | -2.933, 3.31 | 80.66 | 1.57 | 0.023 |
| amisulpride | chronic | homer1b-c | dorsal striatum | mrna levels | 2 | 12 | 12 | 0.972 | -2.829, 4.772 | 0.61623365 | -5.454, 7.398 | 92.92 | 6.99 | 0.0002 |
| amisulpride | chronic | homer1b-c | limbic system | mrna levels | 2 | 12 | 12 | 0.388 | -2.039, 2.814 | 0.7542967 | -3.627, 4.402 | 86.82 | 2.66 | 0.0059 |
| amisulpride | chronic | homer1b-c | sensory-motor areas | mrna levels | 2 | 12 | 12 | 0.282 | -1.513, 2.078 | 0.75789314 | -2.589, 3.154 | 77.85 | 1.31 | 0.0336 |
| amisulpride | chronic | homer1b-c | ventral striatum | mrna levels | 2 | 12 | 12 | 1.215 | -0.913, 3.344 | 0.26305651 | -2.232, 4.663 | 80.98 | 1.91 | 0.0218 |
| amisulpride | chronic | psd95 | cingulate cortex | mrna levels | 2 | 12 | 12 | -0.006 | -0.806, 0.794 | 0.9882636 | -0.806, 0.794 | 0 | 0 | 0.883 |
| amisulpride | chronic | psd95 | dorsal striatum | mrna levels | 2 | 12 | 12 | -0.438 | -1.825, 0.949 | 0.53623677 | -2.53, 1.655 | 63.79 | 0.64 | 0.0966 |
| amisulpride | chronic | psd95 | limbic system | mrna levels | 2 | 12 | 12 | -0.507 | -1.337, 0.324 | 0.23176349 | -1.355, 0.341 | 2.11 | 0.01 | 0.3121 |
| amisulpride | chronic | psd95 | sensory-motor areas | mrna levels | 2 | 12 | 12 | -0.063 | -0.864, 0.737 | 0.87678829 | -0.864, 0.737 | 0 | 0 | 0.8767 |
| amisulpride | chronic | psd95 | ventral striatum | mrna levels | 2 | 12 | 12 | -0.08 | -0.882, 0.722 | 0.844924 | -0.882, 0.722 | 0 | 0 | 0.6302 |
| aripiprazole | acute | arc | dorsal striatum | mrna levels | 2 | 35 | 25 | 1.006 | -2.546, 4.558 | 0.5788137 | -5.071, 7.083 | 96.33 | 6.33 | 0 |
| aripiprazole | acute | arc | frontal cortex | mrna levels | 2 | 35 | 25 | -2.268 | -4.586, 0.051 | 0.05521776 | -6.151, 1.616 | 90.29 | 2.53 | 0.0013 |
| aripiprazole | chronic | homer1a | ventral striatum | protein levels | 2 | 12 | 12 | 2.574 | 0.261, 4.886 | 0.02914093 | -1.097, 6.245 | 75.35 | 2.12 | 0.044 |
| aripiprazole | chronic | homer1b-c | ventral striatum | protein levels | 2 | 12 | 12 | -0.623 | -3.312, 2.065 | 0.6495305 | -5.101, 3.854 | 88.6 | 3.34 | 0.0031 |
| aripiprazole | chronic | mglur1 | ventral striatum | protein levels | 2 | 12 | 12 | -0.371 | -1.178, 0.436 | 0.36756978 | -1.178, 0.436 | 0 | 0 | 0.9855 |
| aripiprazole | chronic | mglur5 | ventral striatum | protein levels | 2 | 12 | 12 | 0.12 | -1.864, 2.104 | 0.90559419 | -3.1, 3.34 | 81.63 | 1.67 | 0.0197 |
| aripiprazole | chronic | norbin | ventral striatum | protein levels | 2 | 12 | 12 | 1.725 | 0.782, 2.667 | 0.00033353 | 0.782, 2.667 | 0 | 0 | 0.4768 |
| aripiprazole | chronic | nr1 | frontal cortex | protein levels | 2 | 12 | 12 | -0.002 | -0.802, 0.798 | 0.99583721 | -0.802, 0.798 | 0 | 0 | 0.9958 |
| aripiprazole | chronic | nr1 | ventral striatum | protein levels | 2 | 12 | 12 | 0.321 | -1.858, 2.501 | 0.77254555 | -3.25, 3.892 | 84.25 | 2.08 | 0.0117 |
| aripiprazole | chronic | psd95 | frontal cortex | protein levels | 2 | 10 | 10 | 1.46 | 0.47, 2.45 | 0.00385209 | 0.47, 2.45 | 0 | 0 | 0.5864 |
| asenapine | acute | homer1a | cingulate cortex | mrna levels | 4 | 46 | 22 | 0.122 | -0.794, 1.038 | 0.79451752 | -1.593, 1.836 | 63.3 | 0.55 | 0.0438 |
| asenapine | acute | homer1a | dorsal striatum | mrna levels | 4 | 46 | 22 | 1.019 | 0.464, 1.574 | 0.00031943 | 0.464, 1.574 | 0 | 0 | 0.4963 |
| asenapine | acute | homer1a | hippocampus | mrna levels | 2 | 10 | 10 | -0.751 | -3.629, 2.127 | 0.60926092 | -5.524, 4.023 | 87.51 | 3.78 | 0.0047 |
| asenapine | acute | homer1a | limbic system | mrna levels | 4 | 46 | 22 | 0.274 | -0.344, 0.893 | 0.38436891 | -0.584, 1.133 | 22.98 | 0.09 | 0.2417 |
| asenapine | acute | homer1a | sensory-motor areas | mrna levels | 4 | 46 | 22 | 0.21 | -0.557, 0.978 | 0.59128363 | -1.104, 1.525 | 48.69 | 0.3 | 0.1174 |
| asenapine | acute | homer1a | ventral striatum | mrna levels | 4 | 46 | 22 | 0.57 | -0.239, 1.379 | 0.16711397 | -0.857, 1.997 | 53.13 | 0.36 | 0.0923 |
| asenapine | chronic | homer1a | cingulate cortex | mrna levels | 2 | 30 | 10 | -0.064 | -0.78, 0.652 | 0.86107453 | -0.78, 0.652 | 0 | 0 | 0.861 |
| asenapine | chronic | homer1a | dorsal striatum | mrna levels | 2 | 30 | 10 | 0.137 | -0.782, 1.057 | 0.76951282 | -1.083, 1.358 | 38.08 | 0.17 | 0.2038 |
| asenapine | chronic | homer1a | limbic system | mrna levels | 2 | 30 | 10 | -1.231 | -2.076, -0.385 | 0.0043296 | -2.208, -0.253 | 16.8 | 0.06 | 0.2729 |
| asenapine | chronic | homer1a | sensory-motor areas | mrna levels | 2 | 30 | 10 | -0.06 | -0.776, 0.657 | 0.86965689 | -0.776, 0.657 | 0 | 0 | 0.6934 |
| asenapine | chronic | homer1a | ventral striatum | mrna levels | 2 | 30 | 10 | 0.431 | -0.458, 1.32 | 0.34183304 | -0.713, 1.576 | 32.81 | 0.14 | 0.2225 |
| clozapine | acute | arc | dorsal striatum | mrna levels | 3 | 32 | 32 | -0.135 | -0.703, 0.434 | 0.64262337 | -0.843, 0.574 | 16.22 | 0.05 | 0.3683 |
| clozapine | acute | arc | frontal cortex | mrna levels | 3 | 32 | 32 | -0.914 | -2.08, 0.252 | 0.12460827 | -3.011, 1.183 | 75.01 | 0.79 | 0.0149 |
| clozapine | acute | arc | ventral striatum | mrna levels | 2 | 25 | 25 | -0.019 | -1.872, 1.834 | 0.98390548 | -3.065, 3.027 | 84.34 | 1.52 | 0.0115 |
| clozapine | acute | homer1a | cingulate cortex | mrna levels | 2 | 12 | 12 | 2.119 | 1.119, 3.119 | 3.2667E-05 | 1.119, 3.119 | 0 | 0 | 0.9358 |
| clozapine | acute | homer1a | dorsal striatum | mrna levels | 3 | 17 | 17 | 1.131 | 0.406, 1.856 | 0.002230667 | 0.406, 1.856 | 0 | 0 | 0.9033 |
| clozapine | acute | homer1a | ventral striatum | mrna levels | 3 | 17 | 17 | 1.478 | 0.716, 2.239 | 0.000142789 | 0.716, 2.239 | 0 | 0 | 0.7652 |
| clozapine | acute | mglur1 | frontal cortex | mrna levels | 2 | 10 | 10 | 0.931 | -0.036, 1.898 | 0.05904279 | -0.095, 1.957 | 6.29 | 0.03 | 0.3016 |
| clozapine | acute | mglur2 | frontal cortex | mrna levels | 2 | 10 | 10 | 0.042 | -0.839, 0.923 | 0.92540925 | -0.839, 0.923 | 0 | 0 | 0.5292 |
| clozapine | acute | mglur5 | frontal cortex | mrna levels | 2 | 10 | 10 | -0.651 | -1.551, 0.249 | 0.15624973 | -1.551, 0.249 | 0 | 0 | 0.7929 |
| clozapine | acute | nr1 | frontal cortex | mrna levels | 3 | 15 | 15 | 1.186 | 0.406, 1.966 | 0.00288038 | 0.406, 1.966 | 0 | 0 | 0.7299 |
| clozapine | acute | nr1 | hippocampus | mrna levels | 3 | 15 | 15 | -0.336 | -1.067, 0.394 | 0.36697868 | -1.067, 0.394 | 0 | 0 | 0.4477 |
| clozapine | acute | nr2a | frontal cortex | mrna levels | 3 | 15 | 15 | 1.01 | 0.241, 1.778 | 0.01000978 | 0.241, 1.778 | 0 | 0 | 0.5164 |
| clozapine | acute | nr2a | hippocampus | mrna levels | 3 | 15 | 15 | 0.049 | -0.677, 0.776 | 0.89395175 | -0.677, 0.776 | 0 | 0 | 0.4126 |
| clozapine | acute | nr2b | frontal cortex | mrna levels | 3 | 15 | 15 | 0.742 | -0.001, 1.485 | 0.0504208 | -0.001, 1.485 | 0 | 0 | 0.7696 |
| clozapine | acute | nr2b | hippocampus | mrna levels | 3 | 15 | 15 | 0.016 | -0.701, 0.734 | 0.96426836 | -0.701, 0.734 | 0 | 0 | 0.8651 |
| clozapine | chronic | mglur1 | frontal cortex | mrna levels | 2 | 20 | 10 | 0.189 | -0.572, 0.95 | 0.62714207 | -0.572, 0.95 | 0 | 0 | 0.7714 |
| clozapine | chronic | mglur2 | frontal cortex | mrna levels | 3 | 25 | 15 | 0.106 | -0.552, 0.764 | 0.75178475 | -0.552, 0.764 | 0 | 0 | 0.3195 |
| clozapine | chronic | mglur5 | frontal cortex | mrna levels | 3 | 25 | 15 | 0.079 | -0.568, 0.727 | 0.81067085 | -0.568, 0.727 | 0 | 0 | 0.9975 |
| clozapine | chronic | nr1 | dorsal striatum | mrna levels | 4 | 29 | 29 | 0.89 | -0.876, 2.656 | 0.3230521 | -2.828, 4.609 | 88.91 | 2.79 | 0.0019 |
| clozapine | chronic | nr1 | dorsal striatum | protein levels | 2 | 17 | 17 | 0.761 | 0.065, 1.457 | 0.03219395 | 0.065, 1.457 | 0 | 0 | 0.9034 |
| clozapine | chronic | nr1 | frontal cortex | mrna levels | 4 | 35 | 35 | 0.138 | -0.545, 0.822 | 0.69196945 | -1.043, 1.319 | 49.79 | 0.24 | 0.1153 |
| clozapine | chronic | nr1 | frontal cortex | protein levels | 2 | 17 | 17 | -0.566 | -1.255, 0.123 | 0.10753823 | -1.255, 0.123 | 0 | 0 | 0.4038 |
| clozapine | chronic | nr1 | hippocampus | mrna levels | 3 | 24 | 24 | 0.656 | 0.007, 1.304 | 0.04744702 | -0.14, 1.451 | 16.55 | 0.06 | 0.2679 |
| clozapine | chronic | nr1 | hippocampus | protein levels | 4 | 28 | 28 | -0.285 | -0.989, 0.418 | 0.42680278 | -1.395, 0.824 | 37.1 | 0.19 | 0.1764 |
| clozapine | chronic | nr1 | ventral striatum | protein levels | 2 | 17 | 17 | 0.076 | -0.736, 0.888 | 0.85489505 | -0.929, 1.08 | 23.83 | 0.09 | 0.2519 |
| clozapine | chronic | nr2a | dorsal striatum | mrna levels | 2 | 15 | 15 | 0.445 | -0.719, 1.609 | 0.45363294 | -1.26, 2.15 | 56.34 | 0.4 | 0.1302 |
| clozapine | chronic | nr2a | frontal cortex | mrna levels | 2 | 21 | 21 | -0.565 | -1.182, 0.052 | 0.07251751 | -1.182, 0.052 | 0 | 0 | 0.9514 |
| clozapine | chronic | nr2a | hippocampus | mrna levels | 2 | 21 | 21 | -0.344 | -0.953, 0.266 | 0.26964694 | -0.953, 0.266 | 0 | 0 | 0.685 |
| clozapine | chronic | nr2a | hippocampus | protein levels | 2 | 11 | 11 | -0.29 | -1.137, 0.556 | 0.50104712 | -1.137, 0.556 | 0 | 0 | 0.4319 |
| clozapine | chronic | nr2b | dorsal striatum | mrna levels | 2 | 15 | 15 | 0.895 | -0.007, 1.797 | 0.0517365 | -0.218, 2.008 | 23.35 | 0.11 | 0.2534 |
| clozapine | chronic | nr2b | hippocampus | protein levels | 2 | 11 | 11 | -0.773 | -2.516, 0.969 | 0.38441126 | -3.501, 1.955 | 72.22 | 1.15 | 0.0578 |
| flupenthixol | chronic | nr1 | associative cortex | mrna levels | 2 | 16 | 16 | -1.687 | -2.495, -0.88 | 4.1643E-05 | -2.495, -0.88 | 0 | 0 | 0.8821 |
| flupenthixol | chronic | nr1 | cerebellum | mrna levels | 2 | 16 | 16 | -1.441 | -2.22, -0.662 | 0.0002901 | -2.22, -0.662 | 0 | 0 | 0.6117 |
| flupenthixol | chronic | nr1 | frontal cortex | mrna levels | 2 | 16 | 16 | -1.505 | -2.367, -0.643 | 0.00062435 | -2.492, -0.518 | 15.35 | 0.06 | 0.2771 |
| flupenthixol | chronic | nr1 | subcortical areas | mrna levels | 2 | 16 | 16 | -1.338 | -2.139, -0.538 | 0.00105306 | -2.191, -0.485 | 6.69 | 0.02 | 0.3006 |
| flupenthixol | chronic | nr2b | associative cortex | mrna levels | 2 | 16 | 16 | -0.116 | -1.377, 1.144 | 0.8566824 | -2.054, 1.821 | 68.16 | 0.56 | 0.0764 |
| flupenthixol | chronic | nr2b | cerebellum | mrna levels | 2 | 16 | 16 | 0.593 | -0.12, 1.305 | 0.10316132 | -0.12, 1.305 | 0 | 0 | 0.3593 |
| flupenthixol | chronic | nr2b | frontal cortex | mrna levels | 2 | 16 | 16 | -0.063 | -0.76, 0.634 | 0.85994183 | -0.76, 0.634 | 0 | 0 | 0.3978 |
| flupenthixol | chronic | nr2b | subcortical areas | mrna levels | 2 | 16 | 16 | 0.076 | -1.266, 1.417 | 0.91200128 | -2.017, 2.169 | 71.73 | 0.67 | 0.06 |
| flupenthixol | chronic | nr2c | associative cortex | mrna levels | 2 | 16 | 16 | -0.067 | -0.762, 0.628 | 0.84984417 | -0.762, 0.628 | 0 | 0 | 0.5402 |
| flupenthixol | chronic | nr2c | cerebellum | mrna levels | 2 | 16 | 16 | -0.047 | -1.612, 1.519 | 0.95318765 | -2.56, 2.466 | 78.84 | 1.01 | 0.0297 |
| flupenthixol | chronic | nr2c | frontal cortex | mrna levels | 2 | 16 | 16 | -0.305 | -1.671, 1.061 | 0.66166713 | -2.441, 1.832 | 72.38 | 0.7 | 0.0571 |
| flupenthixol | chronic | nr2c | subcortical areas | mrna levels | 2 | 16 | 16 | 0.779 | 0.056, 1.503 | 0.03474787 | 0.056, 1.503 | 0 | 0 | 0.3628 |
| flupenthixol | chronic | nr2d | associative cortex | mrna levels | 2 | 16 | 16 | -1.595 | -4.331, 1.142 | 0.25337054 | -6.173, 2.984 | 89.86 | 3.51 | 0.0017 |
| flupenthixol | chronic | nr2d | cerebellum | mrna levels | 2 | 16 | 16 | 0.899 | 0.041, 1.757 | 0.0399452 | -0.163, 1.961 | 26.48 | 0.1 | 0.2435 |
| flupenthixol | chronic | nr2d | frontal cortex | mrna levels | 2 | 16 | 16 | -0.019 | -0.713, 0.675 | 0.95734427 | -0.713, 0.675 | 0 | 0 | 0.6725 |
| flupenthixol | chronic | nr2d | subcortical areas | mrna levels | 2 | 16 | 16 | 0.669 | -0.1, 1.438 | 0.08834587 | -0.193, 1.531 | 12.74 | 0.04 | 0.2844 |
| haloperidol | acute | ania3 | associative cortex | mrna levels | 3 | 17 | 17 | 0.018 | -0.977, 1.013 | 0.97178106 | -1.565, 1.601 | 51.03 | 0.39 | 0.1294 |
| haloperidol | acute | ania3 | cingulate cortex | mrna levels | 4 | 23 | 23 | 0.997 | -0.261, 2.255 | 0.12030827 | -1.478, 3.472 | 72.81 | 1.18 | 0.0125 |
| haloperidol | acute | ania3 | dorsal striatum | mrna levels | 5 | 28 | 29 | 2.471 | 1.076, 3.866 | 0.00051852 | -0.521, 5.463 | 73.64 | 1.82 | 0.0053 |
| haloperidol | acute | ania3 | frontal cortex | mrna levels | 3 | 17 | 17 | -0.136 | -0.817, 0.545 | 0.69530376 | -0.817, 0.545 | 0 | 0 | 0.4424 |
| haloperidol | acute | ania3 | limbic system | mrna levels | 2 | 11 | 11 | 0.615 | -0.241, 1.471 | 0.15884517 | -0.241, 1.471 | 0 | 0 | 0.8625 |
| haloperidol | acute | ania3 | sensory-motor areas | mrna levels | 2 | 11 | 11 | 0.384 | -0.901, 1.67 | 0.5578257 | -1.454, 2.223 | 50.8 | 0.45 | 0.154 |
| haloperidol | acute | ania3 | ventral striatum | mrna levels | 5 | 28 | 29 | 1.425 | 0.607, 2.243 | 0.00064121 | -0.056, 2.906 | 45.76 | 0.4 | 0.1156 |
| haloperidol | acute | arc | cingulate cortex | mrna levels | 5 | 46 | 32 | 0.022 | -0.932, 0.976 | 0.96388064 | -2.026, 2.07 | 72.9 | 0.85 | 0.0035 |
| haloperidol | acute | arc | dorsal striatum | mrna levels | 10 | 114 | 90 | 2.598 | 1.338, 3.857 | 5.2739E-05 | -1.292, 6.488 | 89.96 | 3.53 | 0 |
| haloperidol | acute | arc | frontal cortex | mrna levels | 5 | 68 | 58 | -0.949 | -1.327, -0.57 | 9.0674E-07 | -1.327, -0.57 | 0 | 0 | 0.3895 |
| haloperidol | acute | arc | limbic system | mrna levels | 5 | 46 | 32 | -0.212 | -1.183, 0.759 | 0.66867532 | -2.303, 1.879 | 73.57 | 0.89 | 0.0051 |
| haloperidol | acute | arc | sensory-motor areas | mrna levels | 5 | 46 | 32 | -0.36 | -1.163, 0.442 | 0.3789775 | -1.984, 1.263 | 62.44 | 0.52 | 0.0278 |
| haloperidol | acute | arc | ventral striatum | mrna levels | 7 | 71 | 57 | 1.73 | 1.138, 2.321 | 9.8977E-09 | 0.55, 2.909 | 43.97 | 0.27 | 0.0779 |
| haloperidol | acute | glua1 pser845 | dorsal striatum | protein levels | 4 | 66 | 30 | 2.562 | 0.681, 4.442 | 0.00758779 | -1.381, 6.505 | 89.74 | 3.13 | 0.0009 |
| haloperidol | acute | homer1a | associative cortex | mrna levels | 3 | 17 | 17 | -0.361 | -1.47, 0.749 | 0.52395279 | -2.203, 1.482 | 58.76 | 0.56 | 0.0945 |
| haloperidol | acute | homer1a | cingulate cortex | mrna levels | 11 | 98 | 74 | 0.151 | -0.266, 0.568 | 0.47875811 | -0.8, 1.101 | 39.03 | 0.19 | 0.0559 |
| haloperidol | acute | homer1a | dorsal striatum | mrna levels | 15 | 118 | 94 | 2.114 | 1.428, 2.8 | 1.57187E-09 | -0.097, 4.325 | 70.36 | 1.15 | 0 |
| haloperidol | acute | homer1a | frontal cortex | mrna levels | 3 | 17 | 17 | 0.033 | -0.647, 0.713 | 0.92422709 | -0.647, 0.713 | 0 | 0 | 0.4486 |
| haloperidol | acute | homer1a | limbic system | mrna levels | 9 | 86 | 62 | -0.488 | -1.101, 0.125 | 0.11839857 | -2.057, 1.081 | 64.51 | 0.54 | 0.0057 |
| haloperidol | acute | homer1a | sensory-motor areas | mrna levels | 9 | 86 | 62 | -0.081 | -0.526, 0.364 | 0.72227488 | -0.994, 0.832 | 36.58 | 0.17 | 0.0507 |
| haloperidol | acute | homer1a | ventral striatum | mrna levels | 14 | 113 | 89 | 1.81 | 1.143, 2.477 | 1.03685E-07 | -0.34, 3.96 | 72.04 | 1.09 | 0 |
| haloperidol | acute | homer1b-c | cingulate cortex | mrna levels | 3 | 35 | 21 | 0.527 | -0.319, 1.374 | 0.22198505 | -0.828, 1.883 | 52.17 | 0.29 | 0.1239 |
| haloperidol | acute | homer1b-c | dorsal striatum | mrna levels | 3 | 35 | 21 | 0.693 | 0.011, 1.375 | 0.04647557 | -0.223, 1.608 | 26.56 | 0.1 | 0.2561 |
| haloperidol | acute | homer1b-c | limbic system | mrna levels | 3 | 35 | 21 | 0.466 | -0.386, 1.318 | 0.28390108 | -0.905, 1.837 | 53.01 | 0.3 | 0.1196 |
| haloperidol | acute | homer1b-c | sensory-motor areas | mrna levels | 3 | 35 | 21 | 0.78 | 0.2, 1.359 | 0.0083775 | 0.2, 1.359 | 0 | 0 | 0.5732 |
| haloperidol | acute | homer1b-c | ventral striatum | mrna levels | 3 | 35 | 21 | 0.634 | -0.266, 1.534 | 0.16734847 | -0.848, 2.116 | 57.14 | 0.36 | 0.0967 |
| haloperidol | acute | nr1 | frontal cortex | mrna levels | 3 | 15 | 15 | 0.173 | -0.547, 0.893 | 0.63795846 | -0.547, 0.893 | 0 | 0 | 0.7545 |
| haloperidol | acute | nr1 | hippocampus | mrna levels | 3 | 15 | 15 | -0.067 | -0.783, 0.649 | 0.85489032 | -0.783, 0.649 | 0 | 0 | 0.9714 |
| haloperidol | acute | nr2a | frontal cortex | mrna levels | 3 | 15 | 15 | 0.104 | -0.818, 1.026 | 0.82474506 | -1.217, 1.425 | 34.85 | 0.23 | 0.2292 |
| haloperidol | acute | nr2a | hippocampus | mrna levels | 3 | 15 | 15 | 0.288 | -0.982, 1.559 | 0.65652057 | -1.878, 2.454 | 63.64 | 0.8 | 0.0607 |
| haloperidol | acute | nr2b | frontal cortex | mrna levels | 3 | 15 | 15 | 0.132 | -0.59, 0.854 | 0.7207997 | -0.59, 0.854 | 0 | 0 | 0.6226 |
| haloperidol | acute | nr2b | hippocampus | mrna levels | 3 | 15 | 15 | 0.485 | -0.252, 1.222 | 0.19671704 | -0.252, 1.222 | 0 | 0 | 0.4179 |
| haloperidol | acute | nr2b ptyr1472 | dorsal striatum | protein levels | 2 | 10 | 10 | 0.611 | -0.784, 2.006 | 0.39087515 | -1.422, 2.644 | 56.07 | 0.57 | 0.1314 |
| haloperidol | acute | psd95 | cingulate cortex | mrna levels | 2 | 28 | 14 | -0.027 | -2.344, 2.29 | 0.98171794 | -3.906, 3.852 | 90.1 | 2.52 | 0.0015 |
| haloperidol | acute | psd95 | dorsal striatum | mrna levels | 3 | 33 | 19 | 0.388 | -0.802, 1.578 | 0.52284253 | -1.725, 2.5 | 72.12 | 0.79 | 0.0255 |
| haloperidol | acute | psd95 | limbic system | mrna levels | 2 | 28 | 14 | -0.155 | -0.818, 0.509 | 0.64800273 | -0.818, 0.509 | 0 | 0 | 0.898 |
| haloperidol | acute | psd95 | sensory-motor areas | mrna levels | 2 | 28 | 14 | 0.044 | -1.827, 1.914 | 0.96354758 | -3.037, 3.124 | 85.61 | 1.56 | 0.0084 |
| haloperidol | acute | psd95 | ventral striatum | mrna levels | 3 | 33 | 19 | -0.221 | -1.842, 1.399 | 0.78885952 | -3.254, 2.811 | 83.82 | 1.71 | 0.0037 |
| haloperidol | chronic | ania3 | associative cortex | mrna levels | 2 | 12 | 12 | -0.453 | -1.266, 0.36 | 0.275061 | -1.266, 0.36 | 0 | 0 | 0.5593 |
| haloperidol | chronic | ania3 | cingulate cortex | mrna levels | 2 | 11 | 11 | 0.825 | -0.053, 1.704 | 0.065472 | -0.053, 1.704 | 0 | 0 | 0.3797 |
| haloperidol | chronic | ania3 | dorsal striatum | mrna levels | 3 | 16 | 16 | 2.942 | 1.934, 3.951 | 1.0805E-08 | 1.934, 3.951 | 0 | 0 | 0.5769 |
| haloperidol | chronic | ania3 | frontal cortex | mrna levels | 2 | 12 | 12 | -0.59 | -1.41, 0.23 | 0.1586649 | -1.41, 0.23 | 0 | 0 | 0.5667 |
| haloperidol | chronic | ania3 | ventral striatum | mrna levels | 2 | 11 | 11 | 1.124 | 0.225, 2.024 | 0.01428023 | 0.225, 2.024 | 0 | 0 | 0.9443 |
| haloperidol | chronic | arc | cingulate cortex | mrna levels | 3 | 27 | 17 | -0.706 | -1.892, 0.48 | 0.24339115 | -2.782, 1.37 | 68.75 | 0.76 | 0.0413 |
| haloperidol | chronic | arc | dorsal striatum | mrna levels | 4 | 41 | 31 | 0.636 | -0.88, 2.151 | 0.41107803 | -2.551, 3.823 | 86.73 | 2.05 | 0 |
| haloperidol | chronic | arc | limbic system | mrna levels | 3 | 27 | 17 | -0.62 | -1.697, 0.457 | 0.2589732 | -2.449, 1.209 | 62.88 | 0.57 | 0.0679 |
| haloperidol | chronic | arc | sensory-motor areas | mrna levels | 3 | 27 | 17 | -1.186 | -2.82, 0.448 | 0.15500049 | -4.22, 1.848 | 81.71 | 1.7 | 0.0065 |
| haloperidol | chronic | arc | ventral striatum | mrna levels | 3 | 27 | 17 | 0.451 | -0.185, 1.087 | 0.16483476 | -0.185, 1.087 | 0 | 0 | 0.8622 |
| haloperidol | chronic | glua1 | dorsal striatum | mrna levels | 3 | 19 | 19 | 0.653 | -0.582, 1.888 | 0.30028948 | -1.497, 2.803 | 68.38 | 0.81 | 0.0599 |
| haloperidol | chronic | glua1 | frontal cortex | mrna levels | 2 | 13 | 13 | 0.03 | -0.74, 0.799 | 0.93986102 | -0.74, 0.799 | 0 | 0 | 0.7232 |
| haloperidol | chronic | glua1 | subcortical areas | protein levels | 2 | 14 | 14 | -0.246 | -0.99, 0.498 | 0.51739157 | -0.99, 0.498 | 0 | 0 | 0.7722 |
| haloperidol | chronic | glua1 | ventral striatum | mrna levels | 2 | 13 | 13 | 1.476 | -1.117, 4.069 | 0.26466368 | -2.79, 5.742 | 84.74 | 2.99 | 0.0105 |
| haloperidol | chronic | glua2 | dorsal striatum | mrna levels | 3 | 19 | 19 | 1.198 | -1.442, 3.838 | 0.37376296 | -3.864, 6.261 | 91.54 | 4.86 | 0.0012 |
| haloperidol | chronic | glua2 | frontal cortex | mrna levels | 3 | 19 | 19 | -0.401 | -1.182, 0.38 | 0.31473858 | -1.471, 0.67 | 29.24 | 0.14 | 0.2514 |
| haloperidol | chronic | glua2 | ventral striatum | mrna levels | 3 | 19 | 19 | 0.455 | -1.658, 2.568 | 0.67281193 | -3.567, 4.477 | 87.95 | 3.05 | 0.0003 |
| haloperidol | chronic | homer1a | associative cortex | mrna levels | 2 | 12 | 12 | -0.916 | -1.758, -0.073 | 0.03312873 | -1.758, -0.073 | 0 | 0 | 0.6848 |
| haloperidol | chronic | homer1a | cingulate cortex | mrna levels | 9 | 81 | 61 | 0.139 | -0.69, 0.967 | 0.74260251 | -2.192, 2.47 | 79.55 | 1.24 | 0.0001 |
| haloperidol | chronic | homer1a | dorsal striatum | mrna levels | 10 | 86 | 66 | 1.692 | 1.146, 2.238 | 1.2267E-09 | 0.413, 2.972 | 46.47 | 0.35 | 0.0581 |
| haloperidol | chronic | homer1a | frontal cortex | mrna levels | 2 | 12 | 12 | -0.74 | -1.568, 0.088 | 0.07992892 | -1.568, 0.088 | 0 | 0 | 0.7272 |
| haloperidol | chronic | homer1a | limbic system | mrna levels | 8 | 74 | 54 | 0.145 | -0.738, 1.027 | 0.74798984 | -2.246, 2.535 | 79.91 | 1.28 | 0 |
| haloperidol | chronic | homer1a | sensory-motor areas | mrna levels | 8 | 74 | 54 | 0.296 | -0.745, 1.338 | 0.57722923 | -2.579, 3.171 | 84.78 | 1.87 | 0 |
| haloperidol | chronic | homer1a | ventral striatum | mrna levels | 9 | 81 | 61 | 1.182 | 0.771, 1.593 | 1.7094E-08 | 0.547, 1.817 | 15.48 | 0.06 | 0.342 |
| haloperidol | chronic | homer1a | ventral striatum | protein levels | 2 | 12 | 12 | 0.501 | -0.315, 1.317 | 0.22904963 | -0.315, 1.317 | 0 | 0 | 0.5335 |
| haloperidol | chronic | homer1b-c | cingulate cortex | mrna levels | 6 | 52 | 42 | 0.025 | -0.406, 0.456 | 0.90882645 | -0.406, 0.456 | 0 | 0 | 0.023 |
| haloperidol | chronic | homer1b-c | dorsal striatum | mrna levels | 6 | 52 | 42 | 0.418 | -0.213, 1.049 | 0.19438711 | -0.833, 1.668 | 50.07 | 0.3 | 0.0694 |
| haloperidol | chronic | homer1b-c | limbic system | mrna levels | 6 | 52 | 42 | 0.517 | -0.294, 1.329 | 0.21142841 | -1.294, 2.329 | 68.43 | 0.68 | 0.0063 |
| haloperidol | chronic | homer1b-c | sensory-motor areas | mrna levels | 6 | 52 | 42 | 0.117 | -0.309, 0.543 | 0.59035646 | -0.309, 0.543 | 0 | 0 | 0.1156 |
| haloperidol | chronic | homer1b-c | ventral striatum | mrna levels | 6 | 52 | 42 | 0.684 | 0.034, 1.334 | 0.03906996 | -0.623, 1.991 | 51.75 | 0.33 | 0.059 |
| haloperidol | chronic | homer1b-c | ventral striatum | protein levels | 2 | 12 | 12 | -0.13 | -1.579, 1.318 | 0.85999021 | -2.349, 2.088 | 67.33 | 0.74 | 0.0802 |
| haloperidol | chronic | mglur1 | frontal cortex | mrna levels | 2 | 10 | 10 | 0.083 | -0.796, 0.962 | 0.85313923 | -0.796, 0.962 | 0 | 0 | 0.6545 |
| haloperidol | chronic | mglur1 | ventral striatum | protein levels | 2 | 12 | 12 | -0.518 | -1.333, 0.296 | 0.21198665 | -1.333, 0.296 | 0 | 0 | 0.7791 |
| haloperidol | chronic | mglur2 | frontal cortex | mrna levels | 3 | 15 | 15 | -0.289 | -1.013, 0.436 | 0.43491399 | -1.013, 0.436 | 0 | 0 | 0.6678 |
| haloperidol | chronic | mglur5 | frontal cortex | mrna levels | 3 | 15 | 15 | 0.031 | -0.692, 0.754 | 0.93287782 | -0.692, 0.754 | 0 | 0 | 0.5543 |
| haloperidol | chronic | mglur5 | frontal cortex | protein levels | 2 | 12 | 12 | 0.103 | -0.698, 0.904 | 0.80090662 | -0.698, 0.904 | 0 | 0 | 0.7728 |
| haloperidol | chronic | mglur5 | ventral striatum | protein levels | 2 | 12 | 12 | 0.138 | -0.668, 0.945 | 0.73677055 | -0.668, 0.945 | 0 | 0 | 0.4317 |
| haloperidol | chronic | norbin | frontal cortex | protein levels | 2 | 12 | 12 | -0.469 | -2.552, 1.614 | 0.65892406 | -3.863, 2.925 | 82.72 | 1.87 | 0.0161 |
| haloperidol | chronic | norbin | ventral striatum | protein levels | 2 | 12 | 12 | 1.004 | -0.129, 2.137 | 0.0823441 | -0.531, 2.539 | 41.51 | 0.28 | 0.191 |
| haloperidol | chronic | nr1 | dorsal striatum | mrna levels | 5 | 30 | 30 | 1.586 | 0.487, 2.684 | 0.00466178 | -0.708, 3.879 | 68.57 | 1.06 | 0.0153 |
| haloperidol | chronic | nr1 | dorsal striatum | protein levels | 3 | 23 | 23 | 0.666 | -0.379, 1.711 | 0.2115891 | -1.113, 2.445 | 63.49 | 0.54 | 0.0577 |
| haloperidol | chronic | nr1 | frontal cortex | mrna levels | 4 | 32 | 32 | 0.205 | -0.289, 0.7 | 0.4152762 | -0.289, 0.7 | 0 | 0 | 0.6654 |
| haloperidol | chronic | nr1 | frontal cortex | protein levels | 4 | 29 | 29 | -0.142 | -0.983, 0.7 | 0.74175033 | -1.661, 1.378 | 57.09 | 0.42 | 0.0795 |
| haloperidol | chronic | nr1 | hippocampus | mrna levels | 2 | 16 | 16 | 0.477 | -0.653, 1.606 | 0.40797822 | -1.187, 2.141 | 58.14 | 0.39 | 0.1222 |
| haloperidol | chronic | nr1 | hippocampus | protein levels | 4 | 29 | 29 | -0.264 | -0.786, 0.259 | 0.32257201 | -0.786, 0.259 | 0 | 0 | 0.4909 |
| haloperidol | chronic | nr1 | subcortical areas | protein levels | 2 | 14 | 14 | 0.092 | -0.65, 0.833 | 0.8087153 | -0.65, 0.833 | 0 | 0 | 0.8083 |
| haloperidol | chronic | nr1 | ventral striatum | mrna levels | 4 | 25 | 25 | 0.782 | -0.364, 1.927 | 0.18108743 | -1.433, 2.996 | 70.19 | 0.93 | 0.0245 |
| haloperidol | chronic | nr1 | ventral striatum | protein levels | 3 | 23 | 23 | 0.357 | -0.642, 1.357 | 0.48376684 | -1.315, 2.029 | 60.1 | 0.47 | 0.084 |
| haloperidol | chronic | nr2a | dorsal striatum | mrna levels | 2 | 15 | 15 | 0.148 | -2.97, 3.267 | 0.92566932 | -5.113, 5.41 | 92.28 | 4.67 | 0.0003 |
| haloperidol | chronic | nr2a | dorsal striatum | protein levels | 2 | 11 | 11 | 0.42 | -0.425, 1.265 | 0.32985018 | -0.425, 1.265 | 0 | 0 | 0.899 |
| haloperidol | chronic | nr2a | frontal cortex | mrna levels | 2 | 21 | 21 | -0.544 | -1.16, 0.072 | 0.08333199 | -1.16, 0.072 | 0 | 0 | 0.8752 |
| haloperidol | chronic | nr2a | frontal cortex | protein levels | 3 | 17 | 17 | -0.95 | -1.828, -0.072 | 0.03386986 | -2.176, 0.275 | 31.61 | 0.19 | 0.2228 |
| haloperidol | chronic | nr2a | hippocampus | mrna levels | 2 | 21 | 21 | -0.443 | -1.056, 0.169 | 0.15594529 | -1.056, 0.169 | 0 | 0 | 0.8803 |
| haloperidol | chronic | nr2a | hippocampus | protein levels | 3 | 17 | 17 | -0.551 | -1.33, 0.228 | 0.16574759 | -1.527, 0.426 | 18.89 | 0.09 | 0.2559 |
| haloperidol | chronic | nr2a | ventral striatum | protein levels | 2 | 11 | 11 | 0.086 | -0.755, 0.927 | 0.84077202 | -0.755, 0.927 | 0 | 0 | 0.4828 |
| haloperidol | chronic | nr2b | dorsal striatum | mrna levels | 2 | 15 | 15 | 1.346 | -0.218, 2.909 | 0.09155766 | -1.093, 3.785 | 71.56 | 0.91 | 0.0608 |
| haloperidol | chronic | nr2b | frontal cortex | protein levels | 2 | 11 | 11 | 0.35 | -0.936, 1.635 | 0.5939771 | -1.508, 2.207 | 54.2 | 0.47 | 0.1395 |
| haloperidol | chronic | nr2b | hippocampus | protein levels | 3 | 17 | 17 | -1.031 | -2.132, 0.069 | 0.06625326 | -2.817, 0.754 | 54.49 | 0.51 | 0.1101 |
| haloperidol | chronic | preso1 | frontal cortex | protein levels | 2 | 12 | 12 | 0.041 | -0.76, 0.842 | 0.91960294 | -0.76, 0.842 | 0 | 0 | 0.73 |
| haloperidol | chronic | psd95 | cingulate cortex | mrna levels | 5 | 45 | 35 | 0.251 | -1.452, 1.954 | 0.7726198 | -3.719, 4.221 | 90.03 | 3.35 | 0 |
| haloperidol | chronic | psd95 | dorsal striatum | mrna levels | 5 | 45 | 35 | 0.351 | -0.821, 1.524 | 0.55668069 | -2.261, 2.964 | 81.2 | 1.42 | 0.0002 |
| haloperidol | chronic | psd95 | frontal cortex | protein levels | 3 | 16 | 16 | -0.25 | -0.947, 0.446 | 0.48087516 | -0.947, 0.446 | 0 | 0 | 0.9381 |
| haloperidol | chronic | psd95 | limbic system | mrna levels | 5 | 45 | 35 | -0.301 | -1.306, 0.704 | 0.55702181 | -2.478, 1.876 | 75.36 | 0.97 | 0.0016 |
| haloperidol | chronic | psd95 | sensory-motor areas | mrna levels | 5 | 45 | 35 | 0.09 | -1.156, 1.337 | 0.88692017 | -2.723, 2.904 | 83.33 | 1.66 | 0.0001 |
| haloperidol | chronic | psd95 | ventral striatum | mrna levels | 5 | 45 | 35 | 0.162 | -0.739, 1.064 | 0.72411068 | -1.746, 2.071 | 70.57 | 0.74 | 0.0037 |
| haloperidol | chronic | tamalin | frontal cortex | protein levels | 2 | 12 | 12 | 0.135 | -0.666, 0.936 | 0.74079611 | -0.666, 0.936 | 0 | 0 | 0.9777 |
| haloperidol decanoate | chronic | glua2 | dorsal striatum | mrna levels | 2 | 13 | 13 | 0.973 | -0.106, 2.052 | 0.07706322 | -0.482, 2.429 | 40.37 | 0.25 | 0.1953 |
| olanzapine | acute | arc | dorsal striatum | mrna levels | 3 | 48 | 48 | 1.41 | 0.374, 2.446 | 0.00766289 | -0.482, 3.302 | 78.54 | 0.65 | 0.0162 |
| olanzapine | acute | arc | frontal cortex | mrna levels | 2 | 41 | 41 | -2.154 | -2.699, -1.61 | 8.71E-15 | -2.699, -1.61 | 0 | 0 | 0.737 |
| olanzapine | acute | arc | ventral striatum | mrna levels | 2 | 27 | 27 | 1.486 | 0.882, 2.091 | 1.4558E-06 | 0.882, 2.091 | 0 | 0 | 0.387 |
| olanzapine | acute | homer1a | cingulate cortex | mrna levels | 2 | 12 | 12 | 0.701 | -0.967, 2.368 | 0.41011729 | -1.918, 3.32 | 73.34 | 1.06 | 0.0528 |
| olanzapine | acute | homer1a | dorsal striatum | mrna levels | 4 | 22 | 22 | 3.442 | -1.378, 8.261 | 0.16162121 | -6.974, 13.857 | 97.2 | 22.19 | 0.0001 |
| olanzapine | acute | homer1a | limbic system | mrna levels | 2 | 12 | 12 | 0.947 | -0.16, 2.053 | 0.09368945 | -0.535, 2.428 | 39.54 | 0.25 | 0.1984 |
| olanzapine | acute | homer1a | sensory-motor areas | mrna levels | 2 | 12 | 12 | 0.641 | -0.674, 1.957 | 0.33920261 | -1.298, 2.581 | 58.66 | 0.53 | 0.1199 |
| olanzapine | acute | homer1a | ventral striatum | mrna levels | 4 | 22 | 22 | 1.845 | 0.27, 3.419 | 0.02164256 | -1.318, 5.007 | 77.74 | 1.96 | 0.0086 |
| olanzapine | acute | psd95 | dorsal striatum | mrna levels | 2 | 12 | 12 | 2.066 | -0.591, 4.723 | 0.12756396 | -2.294, 6.426 | 84.44 | 3.11 | 0.0113 |
| olanzapine | acute | psd95 | frontal cortex | protein levels | 6 | 36 | 36 | 2.827 | -0.45, 6.105 | 0.09088374 | -5.553, 11.208 | 96.66 | 15.49 | 0 |
| olanzapine | acute | psd95 | hippocampus | protein levels | 6 | 36 | 36 | -0.136 | -0.619, 0.348 | 0.58238693 | -0.685, 0.414 | 4.85 | 0.02 | 0.3512 |
| olanzapine | acute | psd95 | ventral striatum | mrna levels | 2 | 12 | 12 | 1.438 | -1.025, 3.9 | 0.25247242 | -2.608, 5.483 | 84.92 | 2.68 | 0.01 |
| olanzapine | chronic | arc | dorsal striatum | mrna levels | 2 | 19 | 19 | -1.597 | -2.328, -0.867 | 1.8322E-05 | -2.328, -0.867 | 0 | 0 | 0.7685 |
| olanzapine | chronic | homer1a | cingulate cortex | mrna levels | 2 | 10 | 10 | 0.2 | -0.686, 1.085 | 0.65825126 | -0.686, 1.085 | 0 | 0 | 0.4347 |
| olanzapine | chronic | homer1a | dorsal striatum | mrna levels | 2 | 10 | 10 | 0.342 | -0.631, 1.314 | 0.49105315 | -0.767, 1.45 | 15 | 0.07 | 0.2781 |
| olanzapine | chronic | homer1a | limbic system | mrna levels | 2 | 10 | 10 | -0.245 | -1.127, 0.636 | 0.58547176 | -1.127, 0.636 | 0 | 0 | 0.6992 |
| olanzapine | chronic | homer1a | sensory-motor areas | mrna levels | 2 | 10 | 10 | 0.322 | -0.789, 1.432 | 0.57038596 | -1.121, 1.764 | 34.37 | 0.22 | 0.2171 |
| olanzapine | chronic | homer1a | ventral striatum | mrna levels | 2 | 10 | 10 | 1.185 | -1.506, 3.875 | 0.38814612 | -3.237, 5.606 | 84.85 | 3.2 | 0.0102 |
| olanzapine | chronic | mglur5 | frontal cortex | protein levels | 2 | 12 | 12 | 0.222 | -0.581, 1.025 | 0.58811116 | -0.581, 1.025 | 0 | 0 | 0.7776 |
| olanzapine | chronic | norbin | frontal cortex | protein levels | 2 | 12 | 12 | -0.039 | -0.84, 0.763 | 0.92462699 | -0.84, 0.763 | 0 | 0 | 0.6702 |
| olanzapine | chronic | nr1 | frontal cortex | protein levels | 3 | 16 | 16 | 0.481 | -0.539, 1.501 | 0.35529501 | -1.121, 2.083 | 48.86 | 0.4 | 0.1423 |
| olanzapine | chronic | nr1 | hippocampus | mrna levels | 2 | 11 | 11 | -0.403 | -2.188, 1.381 | 0.65770231 | -3.225, 2.418 | 74.89 | 1.24 | 0.046 |
| olanzapine | chronic | nr1 | hippocampus | protein levels | 3 | 18 | 18 | -0.292 | -1.01, 0.426 | 0.42533578 | -1.143, 0.559 | 13.47 | 0.05 | 0.3056 |
| olanzapine | chronic | nr2a | frontal cortex | protein levels | 2 | 12 | 12 | -0.064 | -1.2, 1.071 | 0.91150552 | -1.656, 1.528 | 48.25 | 0.32 | 0.1645 |
| olanzapine | chronic | nr2a | hippocampus | mrna levels | 2 | 11 | 11 | 0.247 | -0.599, 1.093 | 0.56745235 | -0.599, 1.093 | 0 | 0 | 0.3813 |
| olanzapine | chronic | nr2a | hippocampus | protein levels | 3 | 18 | 18 | -0.089 | -0.749, 0.57 | 0.7907244 | -0.749, 0.57 | 0 | 0 | 0.5197 |
| olanzapine | chronic | nr2b | hippocampus | mrna levels | 2 | 11 | 11 | 0.659 | -0.203, 1.52 | 0.13399263 | -0.203, 1.52 | 0 | 0 | 0.5523 |
| olanzapine | chronic | nr2b | hippocampus | protein levels | 3 | 18 | 18 | -0.278 | -1.261, 0.704 | 0.57862304 | -1.856, 1.299 | 52.55 | 0.4 | 0.122 |
| olanzapine | chronic | preso1 | frontal cortex | protein levels | 2 | 12 | 12 | 0.065 | -0.736, 0.865 | 0.87449228 | -0.736, 0.865 | 0 | 0 | 0.8204 |
| olanzapine | chronic | psd95 | frontal cortex | protein levels | 3 | 16 | 16 | -0.095 | -1.294, 1.103 | 0.87593782 | -2.136, 1.945 | 63.3 | 0.71 | 0.0667 |
| olanzapine | chronic | tamalin | frontal cortex | protein levels | 2 | 12 | 12 | 0.399 | -0.413, 1.212 | 0.33558894 | -0.413, 1.212 | 0 | 0 | 0.4663 |
| quetiapine | acute | homer1a | dorsal striatum | mrna levels | 2 | 24 | 12 | 0.436 | -0.264, 1.136 | 0.22239809 | -0.264, 1.136 | 0 | 0 | 0.9673 |
| quetiapine | chronic | homer1a | dorsal striatum | mrna levels | 2 | 19 | 12 | 0.648 | -0.822, 2.119 | 0.38764893 | -1.624, 2.921 | 68.25 | 0.78 | 0.076 |

## Results after adjustment of p-values for multiple testing

| **drug** | **model** | **psd type** | **region** | **outcome type** | **n_studies** | **n_group_1** | **n_group_2** | **beta** | **CI** | **adj_p_BH** | **PI** | **i_sq** | **tau_sq** | **q_p_val** |
| --- | --- | --- | --- | --- | --- | --- | --- | --- | --- | --- | --- | --- | --- | --- |
| amisulpride | chronic | arc | cingulate cortex | mrna levels | 2 | 12 | 12 | 0.22 | -0.583, 1.024 | 0.908857 | -0.583, 1.024 | 0 | 0 | 0.7398 |
| amisulpride | chronic | arc | dorsal striatum | mrna levels | 2 | 12 | 12 | 1.235 | 0.359, 2.111 | 0.048036 | 0.359, 2.111 | 0 | 0 | 0.5518 |
| amisulpride | chronic | arc | limbic system | mrna levels | 2 | 12 | 12 | 0.454 | -0.485, 1.393 | 0.739338 | -0.684, 1.592 | 23.38 | 0.11 | 0.2533 |
| amisulpride | chronic | arc | sensory-motor areas | mrna levels | 2 | 12 | 12 | 0.12 | -0.685, 0.925 | 0.972587 | -0.685, 0.925 | 0 | 0 | 0.476 |
| amisulpride | chronic | arc | ventral striatum | mrna levels | 2 | 12 | 12 | 0.825 | -0.01, 1.66 | 0.264678 | -0.01, 1.66 | 0 | 0 | 0.6841 |
| amisulpride | chronic | homer1a | cingulate cortex | mrna levels | 2 | 12 | 12 | 1.355 | -1.757, 4.466 | 0.760047 | -3.848, 6.557 | 89.66 | 4.53 | 0.0019 |
| amisulpride | chronic | homer1a | dorsal striatum | mrna levels | 2 | 12 | 12 | 1.804 | -0.412, 4.02 | 0.403373 | -1.764, 5.372 | 79.2 | 2.04 | 0.0283 |
| amisulpride | chronic | homer1a | limbic system | mrna levels | 2 | 12 | 12 | 2.678 | -3.045, 8.401 | 0.758302 | -7.041, 12.397 | 94.06 | 16.06 | 0 |
| amisulpride | chronic | homer1a | sensory-motor areas | mrna levels | 2 | 12 | 12 | 0.575 | -2.134, 3.285 | 0.933391 | -3.94, 5.091 | 88.81 | 3.4 | 0.0028 |
| amisulpride | chronic | homer1a | ventral striatum | mrna levels | 2 | 12 | 12 | 0.982 | -0.103, 2.067 | 0.337398 | -0.449, 2.413 | 36.76 | 0.23 | 0.2086 |
| amisulpride | chronic | homer1b-c | cingulate cortex | mrna levels | 2 | 12 | 12 | 0.189 | -1.742, 2.12 | 1 | -2.933, 3.31 | 80.66 | 1.57 | 0.023 |
| amisulpride | chronic | homer1b-c | dorsal striatum | mrna levels | 2 | 12 | 12 | 0.972 | -2.829, 4.772 | 0.92231 | -5.454, 7.398 | 92.92 | 6.99 | 0.0002 |
| amisulpride | chronic | homer1b-c | limbic system | mrna levels | 2 | 12 | 12 | 0.388 | -2.039, 2.814 | 0.968586 | -3.627, 4.402 | 86.82 | 2.66 | 0.0059 |
| amisulpride | chronic | homer1b-c | sensory-motor areas | mrna levels | 2 | 12 | 12 | 0.282 | -1.513, 2.078 | 0.967705 | -2.589, 3.154 | 77.85 | 1.31 | 0.0336 |
| amisulpride | chronic | homer1b-c | ventral striatum | mrna levels | 2 | 12 | 12 | 1.215 | -0.913, 3.344 | 0.646204 | -2.232, 4.663 | 80.98 | 1.91 | 0.0218 |
| amisulpride | chronic | psd95 | cingulate cortex | mrna levels | 2 | 12 | 12 | -0.006 | -0.806, 0.794 | 0.992656 | -0.806, 0.794 | 0 | 0 | 0.883 |
| amisulpride | chronic | psd95 | dorsal striatum | mrna levels | 2 | 12 | 12 | -0.438 | -1.825, 0.949 | 0.904399 | -2.53, 1.655 | 63.79 | 0.64 | 0.0966 |
| amisulpride | chronic | psd95 | limbic system | mrna levels | 2 | 12 | 12 | -0.507 | -1.337, 0.324 | 0.602052 | -1.355, 0.341 | 2.11 | 0.01 | 0.3121 |
| amisulpride | chronic | psd95 | sensory-motor areas | mrna levels | 2 | 12 | 12 | -0.063 | -0.864, 0.737 | 0.976129 | -0.864, 0.737 | 0 | 0 | 0.8767 |
| amisulpride | chronic | psd95 | ventral striatum | mrna levels | 2 | 12 | 12 | -0.08 | -0.882, 0.722 | 1 | -0.882, 0.722 | 0 | 0 | 0.6302 |
| aripiprazole | acute | arc | dorsal striatum | mrna levels | 2 | 35 | 25 | 1.006 | -2.546, 4.558 | 0.921211 | -5.071, 7.083 | 96.33 | 6.33 | 0 |
| aripiprazole | acute | arc | frontal cortex | mrna levels | 2 | 35 | 25 | -2.268 | -4.586, 0.051 | 0.271287 | -6.151, 1.616 | 90.29 | 2.53 | 0.0013 |
| aripiprazole | chronic | homer1a | ventral striatum | protein levels | 2 | 12 | 12 | 2.574 | 0.261, 4.886 | 0.193701 | -1.097, 6.245 | 75.35 | 2.12 | 0.044 |
| aripiprazole | chronic | homer1b-c | ventral striatum | protein levels | 2 | 12 | 12 | -0.623 | -3.312, 2.065 | 0.940986 | -5.101, 3.854 | 88.6 | 3.34 | 0.0031 |
| aripiprazole | chronic | mglur1 | ventral striatum | protein levels | 2 | 12 | 12 | -0.371 | -1.178, 0.436 | 0.762117 | -1.178, 0.436 | 0 | 0 | 0.9855 |
| aripiprazole | chronic | mglur5 | ventral striatum | protein levels | 2 | 12 | 12 | 0.12 | -1.864, 2.104 | 0.993516 | -3.1, 3.34 | 81.63 | 1.67 | 0.0197 |
| aripiprazole | chronic | norbin | ventral striatum | protein levels | 2 | 12 | 12 | 1.725 | 0.782, 2.667 | 0.004434 | 0.782, 2.667 | 0 | 0 | 0.4768 |
| aripiprazole | chronic | nr1 | frontal cortex | protein levels | 2 | 12 | 12 | -0.002 | -0.802, 0.798 | 0.995837 | -0.802, 0.798 | 0 | 0 | 0.9958 |
| aripiprazole | chronic | nr1 | ventral striatum | protein levels | 2 | 12 | 12 | 0.321 | -1.858, 2.501 | 0.969974 | -3.25, 3.892 | 84.25 | 2.08 | 0.0117 |
| aripiprazole | chronic | psd95 | frontal cortex | protein levels | 2 | 10 | 10 | 1.46 | 0.47, 2.45 | 0.036274 | 0.47, 2.45 | 0 | 0 | 0.5864 |
| asenapine | acute | homer1a | cingulate cortex | mrna levels | 4 | 46 | 22 | 0.122 | -0.794, 1.038 | 0.975875 | -1.593, 1.836 | 63.3 | 0.55 | 0.0438 |
| asenapine | acute | homer1a | dorsal striatum | mrna levels | 4 | 46 | 22 | 1.019 | 0.464, 1.574 | 0.004512 | 0.464, 1.574 | 0 | 0 | 0.4963 |
| asenapine | acute | homer1a | hippocampus | mrna levels | 2 | 10 | 10 | -0.751 | -3.629, 2.127 | 0.917953 | -5.524, 4.023 | 87.51 | 3.78 | 0.0047 |
| asenapine | acute | homer1a | limbic system | mrna levels | 4 | 46 | 22 | 0.274 | -0.344, 0.893 | 0.775602 | -0.584, 1.133 | 22.98 | 0.09 | 0.2417 |
| asenapine | acute | homer1a | sensory-motor areas | mrna levels | 4 | 46 | 22 | 0.21 | -0.557, 0.978 | 0.902906 | -1.104, 1.525 | 48.69 | 0.3 | 0.1174 |
| asenapine | acute | homer1a | ventral striatum | mrna levels | 4 | 46 | 22 | 0.57 | -0.239, 1.379 | 0.496944 | -0.857, 1.997 | 53.13 | 0.36 | 0.0923 |
| asenapine | chronic | homer1a | cingulate cortex | mrna levels | 2 | 30 | 10 | -0.064 | -0.78, 0.652 | 0.977904 | -0.78, 0.652 | 0 | 0 | 0.861 |
| asenapine | chronic | homer1a | dorsal striatum | mrna levels | 2 | 30 | 10 | 0.137 | -0.782, 1.057 | 0.977022 | -1.083, 1.358 | 38.08 | 0.17 | 0.2038 |
| asenapine | chronic | homer1a | limbic system | mrna levels | 2 | 30 | 10 | -1.231 | -2.076, -0.385 | 0.03914 | -2.208, -0.253 | 16.8 | 0.06 | 0.2729 |
| asenapine | chronic | homer1a | sensory-motor areas | mrna levels | 2 | 30 | 10 | -0.06 | -0.776, 0.657 | 0.982712 | -0.776, 0.657 | 0 | 0 | 0.6934 |
| asenapine | chronic | homer1a | ventral striatum | mrna levels | 2 | 30 | 10 | 0.431 | -0.458, 1.32 | 0.74283 | -0.713, 1.576 | 32.81 | 0.14 | 0.2225 |
| clozapine | acute | arc | dorsal striatum | mrna levels | 3 | 32 | 32 | -0.135 | -0.703, 0.434 | 0.943071 | -0.843, 0.574 | 16.22 | 0.05 | 0.3683 |
| clozapine | acute | arc | frontal cortex | mrna levels | 3 | 32 | 32 | -0.914 | -2.08, 0.252 | 0.433253 | -3.011, 1.183 | 75.01 | 0.79 | 0.0149 |
| clozapine | acute | arc | ventral striatum | mrna levels | 2 | 25 | 25 | -0.019 | -1.872, 1.834 | 0.99269 | -3.065, 3.027 | 84.34 | 1.52 | 0.0115 |
| clozapine | acute | homer1a | cingulate cortex | mrna levels | 2 | 12 | 12 | 2.119 | 1.119, 3.119 | 0.000671 | 1.119, 3.119 | 0 | 0 | 0.9358 |
| clozapine | acute | homer1a | dorsal striatum | mrna levels | 3 | 17 | 17 | 1.131 | 0.406, 1.856 | 0.022915 | 0.406, 1.856 | 0 | 0 | 0.9033 |
| clozapine | acute | homer1a | ventral striatum | mrna levels | 3 | 17 | 17 | 1.478 | 0.716, 2.239 | 0.002305 | 0.716, 2.239 | 0 | 0 | 0.7652 |
| clozapine | acute | mglur1 | frontal cortex | mrna levels | 2 | 10 | 10 | 0.931 | -0.036, 1.898 | 0.283908 | -0.095, 1.957 | 6.29 | 0.03 | 0.3016 |
| clozapine | acute | mglur2 | frontal cortex | mrna levels | 2 | 10 | 10 | 0.042 | -0.839, 0.923 | 0.98189 | -0.839, 0.923 | 0 | 0 | 0.5292 |
| clozapine | acute | mglur5 | frontal cortex | mrna levels | 2 | 10 | 10 | -0.651 | -1.551, 0.249 | 0.504463 | -1.551, 0.249 | 0 | 0 | 0.7929 |
| clozapine | acute | nr1 | frontal cortex | mrna levels | 3 | 15 | 15 | 1.186 | 0.406, 1.966 | 0.028303 | 0.406, 1.966 | 0 | 0 | 0.7299 |
| clozapine | acute | nr1 | hippocampus | mrna levels | 3 | 15 | 15 | -0.336 | -1.067, 0.394 | 0.767937 | -1.067, 0.394 | 0 | 0 | 0.4477 |
| clozapine | acute | nr2a | frontal cortex | mrna levels | 3 | 15 | 15 | 1.01 | 0.241, 1.778 | 0.072975 | 0.241, 1.778 | 0 | 0 | 0.5164 |
| clozapine | acute | nr2a | hippocampus | mrna levels | 3 | 15 | 15 | 0.049 | -0.677, 0.776 | 0.985527 | -0.677, 0.776 | 0 | 0 | 0.4126 |
| clozapine | acute | nr2b | frontal cortex | mrna levels | 3 | 15 | 15 | 0.742 | -0.001, 1.485 | 0.265002 | -0.001, 1.485 | 0 | 0 | 0.7696 |
| clozapine | acute | nr2b | hippocampus | mrna levels | 3 | 15 | 15 | 0.016 | -0.701, 0.734 | 0.986084 | -0.701, 0.734 | 0 | 0 | 0.8651 |
| clozapine | chronic | mglur1 | frontal cortex | mrna levels | 2 | 20 | 10 | 0.189 | -0.572, 0.95 | 0.932461 | -0.572, 0.95 | 0 | 0 | 0.7714 |
| clozapine | chronic | mglur2 | frontal cortex | mrna levels | 3 | 25 | 15 | 0.106 | -0.552, 0.764 | 0.970876 | -0.552, 0.764 | 0 | 0 | 0.3195 |
| clozapine | chronic | mglur5 | frontal cortex | mrna levels | 3 | 25 | 15 | 0.079 | -0.568, 0.727 | 0.979741 | -0.568, 0.727 | 0 | 0 | 0.9975 |
| clozapine | chronic | nr1 | dorsal striatum | mrna levels | 4 | 29 | 29 | 0.89 | -0.876, 2.656 | 0.730098 | -2.828, 4.609 | 88.91 | 2.79 | 0.0019 |
| clozapine | chronic | nr1 | dorsal striatum | protein levels | 2 | 17 | 17 | 0.761 | 0.065, 1.457 | 0.207881 | 0.065, 1.457 | 0 | 0 | 0.9034 |
| clozapine | chronic | nr1 | frontal cortex | mrna levels | 4 | 35 | 35 | 0.138 | -0.545, 0.822 | 0.947788 | -1.043, 1.319 | 49.79 | 0.24 | 0.1153 |
| clozapine | chronic | nr1 | frontal cortex | protein levels | 2 | 17 | 17 | -0.566 | -1.255, 0.123 | 0.39842 | -1.255, 0.123 | 0 | 0 | 0.4038 |
| clozapine | chronic | nr1 | hippocampus | mrna levels | 3 | 24 | 24 | 0.656 | 0.007, 1.304 | 0.25531 | -0.14, 1.451 | 16.55 | 0.06 | 0.2679 |
| clozapine | chronic | nr1 | hippocampus | protein levels | 4 | 28 | 28 | -0.285 | -0.989, 0.418 | 0.784207 | -1.395, 0.824 | 37.1 | 0.19 | 0.1764 |
| clozapine | chronic | nr1 | ventral striatum | protein levels | 2 | 17 | 17 | 0.076 | -0.736, 0.888 | 0.990801 | -0.929, 1.08 | 23.83 | 0.09 | 0.2519 |
| clozapine | chronic | nr2a | dorsal striatum | mrna levels | 2 | 15 | 15 | 0.445 | -0.719, 1.609 | 0.820168 | -1.26, 2.15 | 56.34 | 0.4 | 0.1302 |
| clozapine | chronic | nr2a | frontal cortex | mrna levels | 2 | 21 | 21 | -0.565 | -1.182, 0.052 | 0.327779 | -1.182, 0.052 | 0 | 0 | 0.9514 |
| clozapine | chronic | nr2a | hippocampus | mrna levels | 2 | 21 | 21 | -0.344 | -0.953, 0.266 | 0.6483 | -0.953, 0.266 | 0 | 0 | 0.685 |
| clozapine | chronic | nr2a | hippocampus | protein levels | 2 | 11 | 11 | -0.29 | -1.137, 0.556 | 0.871051 | -1.137, 0.556 | 0 | 0 | 0.4319 |
| clozapine | chronic | nr2b | dorsal striatum | mrna levels | 2 | 15 | 15 | 0.895 | -0.007, 1.797 | 0.265737 | -0.218, 2.008 | 23.35 | 0.11 | 0.2534 |
| clozapine | chronic | nr2b | hippocampus | protein levels | 2 | 11 | 11 | -0.773 | -2.516, 0.969 | 0.768823 | -3.501, 1.955 | 72.22 | 1.15 | 0.0578 |
| flupenthixol | chronic | nr1 | associative cortex | mrna levels | 2 | 16 | 16 | -1.687 | -2.495, -0.88 | 0.000784 | -2.495, -0.88 | 0 | 0 | 0.8821 |
| flupenthixol | chronic | nr1 | cerebellum | mrna levels | 2 | 16 | 16 | -1.441 | -2.22, -0.662 | 0.004371 | -2.22, -0.662 | 0 | 0 | 0.6117 |
| flupenthixol | chronic | nr1 | frontal cortex | mrna levels | 2 | 16 | 16 | -1.505 | -2.367, -0.643 | 0.007426 | -2.492, -0.518 | 15.35 | 0.06 | 0.2771 |
| flupenthixol | chronic | nr1 | subcortical areas | mrna levels | 2 | 16 | 16 | -1.338 | -2.139, -0.538 | 0.011333 | -2.191, -0.485 | 6.69 | 0.02 | 0.3006 |
| flupenthixol | chronic | nr2b | associative cortex | mrna levels | 2 | 16 | 16 | -0.116 | -1.377, 1.144 | 0.987807 | -2.054, 1.821 | 68.16 | 0.56 | 0.0764 |
| flupenthixol | chronic | nr2b | cerebellum | mrna levels | 2 | 16 | 16 | 0.593 | -0.12, 1.305 | 0.388574 | -0.12, 1.305 | 0 | 0 | 0.3593 |
| flupenthixol | chronic | nr2b | frontal cortex | mrna levels | 2 | 16 | 16 | -0.063 | -0.76, 0.634 | 0.986532 | -0.76, 0.634 | 0 | 0 | 0.3978 |
| flupenthixol | chronic | nr2b | subcortical areas | mrna levels | 2 | 16 | 16 | 0.076 | -1.266, 1.417 | 0.986183 | -2.017, 2.169 | 71.73 | 0.67 | 0.06 |
| flupenthixol | chronic | nr2c | associative cortex | mrna levels | 2 | 16 | 16 | -0.067 | -0.762, 0.628 | 1 | -0.762, 0.628 | 0 | 0 | 0.5402 |
| flupenthixol | chronic | nr2c | cerebellum | mrna levels | 2 | 16 | 16 | -0.047 | -1.612, 1.519 | 0.992721 | -2.56, 2.466 | 78.84 | 1.01 | 0.0297 |
| flupenthixol | chronic | nr2c | frontal cortex | mrna levels | 2 | 16 | 16 | -0.305 | -1.671, 1.061 | 0.9288 | -2.441, 1.832 | 72.38 | 0.7 | 0.0571 |
| flupenthixol | chronic | nr2c | subcortical areas | mrna levels | 2 | 16 | 16 | 0.779 | 0.056, 1.503 | 0.206658 | 0.056, 1.503 | 0 | 0 | 0.3628 |
| flupenthixol | chronic | nr2d | associative cortex | mrna levels | 2 | 16 | 16 | -1.595 | -4.331, 1.142 | 0.636242 | -6.173, 2.984 | 89.86 | 3.51 | 0.0017 |
| flupenthixol | chronic | nr2d | cerebellum | mrna levels | 2 | 16 | 16 | 0.899 | 0.041, 1.757 | 0.22569 | -0.163, 1.961 | 26.48 | 0.1 | 0.2435 |
| flupenthixol | chronic | nr2d | frontal cortex | mrna levels | 2 | 16 | 16 | -0.019 | -0.713, 0.675 | 0.992476 | -0.713, 0.675 | 0 | 0 | 0.6725 |
| flupenthixol | chronic | nr2d | subcortical areas | mrna levels | 2 | 16 | 16 | 0.669 | -0.1, 1.438 | 0.356539 | -0.193, 1.531 | 12.74 | 0.04 | 0.2844 |
| haloperidol | acute | ania3 | associative cortex | mrna levels | 3 | 17 | 17 | 0.018 | -0.977, 1.013 | 0.989291 | -1.565, 1.601 | 51.03 | 0.39 | 0.1294 |
| haloperidol | acute | ania3 | cingulate cortex | mrna levels | 4 | 23 | 23 | 0.997 | -0.261, 2.255 | 0.424839 | -1.478, 3.472 | 72.81 | 1.18 | 0.0125 |
| haloperidol | acute | ania3 | dorsal striatum | mrna levels | 5 | 28 | 29 | 2.471 | 1.076, 3.866 | 0.00651 | -0.521, 5.463 | 73.64 | 1.82 | 0.0053 |
| haloperidol | acute | ania3 | frontal cortex | mrna levels | 3 | 17 | 17 | -0.136 | -0.817, 0.545 | 0.946618 | -0.817, 0.545 | 0 | 0 | 0.4424 |
| haloperidol | acute | ania3 | limbic system | mrna levels | 2 | 11 | 11 | 0.615 | -0.241, 1.471 | 0.498597 | -0.241, 1.471 | 0 | 0 | 0.8625 |
| haloperidol | acute | ania3 | sensory-motor areas | mrna levels | 2 | 11 | 11 | 0.384 | -0.901, 1.67 | 0.920209 | -1.454, 2.223 | 50.8 | 0.45 | 0.154 |
| haloperidol | acute | ania3 | ventral striatum | mrna levels | 5 | 28 | 29 | 1.425 | 0.607, 2.243 | 0.007246 | -0.056, 2.906 | 45.76 | 0.4 | 0.1156 |
| haloperidol | acute | arc | cingulate cortex | mrna levels | 5 | 46 | 32 | 0.022 | -0.932, 0.976 | 0.990168 | -2.026, 2.07 | 72.9 | 0.85 | 0.0035 |
| haloperidol | acute | arc | dorsal striatum | mrna levels | 10 | 114 | 90 | 2.598 | 1.338, 3.857 | 0.000917 | -1.292, 6.488 | 89.96 | 3.53 | 0 |
| haloperidol | acute | arc | frontal cortex | mrna levels | 5 | 68 | 58 | -0.949 | -1.327, -0.57 | 2.56E-05 | -1.327, -0.57 | 0 | 0 | 0.3895 |
| haloperidol | acute | arc | limbic system | mrna levels | 5 | 46 | 32 | -0.212 | -1.183, 0.759 | 0.932843 | -2.303, 1.879 | 73.57 | 0.89 | 0.0051 |
| haloperidol | acute | arc | sensory-motor areas | mrna levels | 5 | 46 | 32 | -0.36 | -1.163, 0.442 | 0.771612 | -1.984, 1.263 | 62.44 | 0.52 | 0.0278 |
| haloperidol | acute | arc | ventral striatum | mrna levels | 7 | 71 | 57 | 1.73 | 1.138, 2.321 | 5.59E-07 | 0.55, 2.909 | 43.97 | 0.27 | 0.0779 |
| haloperidol | acute | glua1 pser845 | dorsal striatum | protein levels | 4 | 66 | 30 | 2.562 | 0.681, 4.442 | 0.061244 | -1.381, 6.505 | 89.74 | 3.13 | 0.0009 |
| haloperidol | acute | homer1a | associative cortex | mrna levels | 3 | 17 | 17 | -0.361 | -1.47, 0.749 | 0.890326 | -2.203, 1.482 | 58.76 | 0.56 | 0.0945 |
| haloperidol | acute | homer1a | cingulate cortex | mrna levels | 11 | 98 | 74 | 0.151 | -0.266, 0.568 | 0.858725 | -0.8, 1.101 | 39.03 | 0.19 | 0.0559 |
| haloperidol | acute | homer1a | dorsal striatum | mrna levels | 15 | 118 | 94 | 2.114 | 1.428, 2.8 | 1.18E-07 | -0.097, 4.325 | 70.36 | 1.15 | 0 |
| haloperidol | acute | homer1a | frontal cortex | mrna levels | 3 | 17 | 17 | 0.033 | -0.647, 0.713 | 0.98993 | -0.647, 0.713 | 0 | 0 | 0.4486 |
| haloperidol | acute | homer1a | limbic system | mrna levels | 9 | 86 | 62 | -0.488 | -1.101, 0.125 | 0.424731 | -2.057, 1.081 | 64.51 | 0.54 | 0.0057 |
| haloperidol | acute | homer1a | sensory-motor areas | mrna levels | 9 | 86 | 62 | -0.081 | -0.526, 0.364 | 0.971632 | -0.994, 0.832 | 36.58 | 0.17 | 0.0507 |
| haloperidol | acute | homer1a | ventral striatum | mrna levels | 14 | 113 | 89 | 1.81 | 1.143, 2.477 | 3.35E-06 | -0.34, 3.96 | 72.04 | 1.09 | 0 |
| haloperidol | acute | homer1b-c | cingulate cortex | mrna levels | 3 | 35 | 21 | 0.527 | -0.319, 1.374 | 0.597245 | -0.828, 1.883 | 52.17 | 0.29 | 0.1239 |
| haloperidol | acute | homer1b-c | dorsal striatum | mrna levels | 3 | 35 | 21 | 0.693 | 0.011, 1.375 | 0.256182 | -0.223, 1.608 | 26.56 | 0.1 | 0.2561 |
| haloperidol | acute | homer1b-c | limbic system | mrna levels | 3 | 35 | 21 | 0.466 | -0.386, 1.318 | 0.66835 | -0.905, 1.837 | 53.01 | 0.3 | 0.1196 |
| haloperidol | acute | homer1b-c | sensory-motor areas | mrna levels | 3 | 35 | 21 | 0.78 | 0.2, 1.359 | 0.063111 | 0.2, 1.359 | 0 | 0 | 0.5732 |
| haloperidol | acute | homer1b-c | ventral striatum | mrna levels | 3 | 35 | 21 | 0.634 | -0.266, 1.534 | 0.491179 | -0.848, 2.116 | 57.14 | 0.36 | 0.0967 |
| haloperidol | acute | nr1 | frontal cortex | mrna levels | 3 | 15 | 15 | 0.173 | -0.547, 0.893 | 0.942344 | -0.547, 0.893 | 0 | 0 | 0.7545 |
| haloperidol | acute | nr1 | hippocampus | mrna levels | 3 | 15 | 15 | -0.067 | -0.783, 0.649 | 0.995903 | -0.783, 0.649 | 0 | 0 | 0.9714 |
| haloperidol | acute | nr2a | frontal cortex | mrna levels | 3 | 15 | 15 | 0.104 | -0.818, 1.026 | 0.991449 | -1.217, 1.425 | 34.85 | 0.23 | 0.2292 |
| haloperidol | acute | nr2a | hippocampus | mrna levels | 3 | 15 | 15 | 0.288 | -0.982, 1.559 | 0.945055 | -1.878, 2.454 | 63.64 | 0.8 | 0.0607 |
| haloperidol | acute | nr2b | frontal cortex | mrna levels | 3 | 15 | 15 | 0.132 | -0.59, 0.854 | 0.975453 | -0.59, 0.854 | 0 | 0 | 0.6226 |
| haloperidol | acute | nr2b | hippocampus | mrna levels | 3 | 15 | 15 | 0.485 | -0.252, 1.222 | 0.555726 | -0.252, 1.222 | 0 | 0 | 0.4179 |
| haloperidol | acute | nr2b ptyr1472 | dorsal striatum | protein levels | 2 | 10 | 10 | 0.611 | -0.784, 2.006 | 0.761533 | -1.422, 2.644 | 56.07 | 0.57 | 0.1314 |
| haloperidol | acute | psd95 | cingulate cortex | mrna levels | 2 | 28 | 14 | -0.027 | -2.344, 2.29 | 0.994925 | -3.906, 3.852 | 90.1 | 2.52 | 0.0015 |
| haloperidol | acute | psd95 | dorsal striatum | mrna levels | 3 | 33 | 19 | 0.388 | -0.802, 1.578 | 0.89517 | -1.725, 2.5 | 72.12 | 0.79 | 0.0255 |
| haloperidol | acute | psd95 | limbic system | mrna levels | 2 | 28 | 14 | -0.155 | -0.818, 0.509 | 0.94483 | -0.818, 0.509 | 0 | 0 | 0.898 |
| haloperidol | acute | psd95 | sensory-motor areas | mrna levels | 2 | 28 | 14 | 0.044 | -1.827, 1.914 | 0.994346 | -3.037, 3.124 | 85.61 | 1.56 | 0.0084 |
| haloperidol | acute | psd95 | ventral striatum | mrna levels | 3 | 33 | 19 | -0.221 | -1.842, 1.399 | 0.979573 | -3.254, 2.811 | 83.82 | 1.71 | 0.0037 |
| haloperidol | chronic | ania3 | associative cortex | mrna levels | 2 | 12 | 12 | -0.453 | -1.266, 0.36 | 0.654356 | -1.266, 0.36 | 0 | 0 | 0.5593 |
| haloperidol | chronic | ania3 | cingulate cortex | mrna levels | 2 | 11 | 11 | 0.825 | -0.053, 1.704 | 0.308264 | -0.053, 1.704 | 0 | 0 | 0.3797 |
| haloperidol | chronic | ania3 | dorsal striatum | mrna levels | 3 | 16 | 16 | 2.942 | 1.934, 3.951 | 4.88E-07 | 1.934, 3.951 | 0 | 0 | 0.5769 |
| haloperidol | chronic | ania3 | frontal cortex | mrna levels | 2 | 12 | 12 | -0.59 | -1.41, 0.23 | 0.505046 | -1.41, 0.23 | 0 | 0 | 0.5667 |
| haloperidol | chronic | ania3 | ventral striatum | mrna levels | 2 | 11 | 11 | 1.124 | 0.225, 2.024 | 0.100854 | 0.225, 2.024 | 0 | 0 | 0.9443 |
| haloperidol | chronic | arc | cingulate cortex | mrna levels | 3 | 27 | 17 | -0.706 | -1.892, 0.48 | 0.625073 | -2.782, 1.37 | 68.75 | 0.76 | 0.0413 |
| haloperidol | chronic | arc | dorsal striatum | mrna levels | 4 | 41 | 31 | 0.636 | -0.88, 2.151 | 0.774197 | -2.551, 3.823 | 86.73 | 2.05 | 0 |
| haloperidol | chronic | arc | limbic system | mrna levels | 3 | 27 | 17 | -0.62 | -1.697, 0.457 | 0.643164 | -2.449, 1.209 | 62.88 | 0.57 | 0.0679 |
| haloperidol | chronic | arc | sensory-motor areas | mrna levels | 3 | 27 | 17 | -1.186 | -2.82, 0.448 | 0.515149 | -4.22, 1.848 | 81.71 | 1.7 | 0.0065 |
| haloperidol | chronic | arc | ventral striatum | mrna levels | 3 | 27 | 17 | 0.451 | -0.185, 1.087 | 0.503414 | -0.185, 1.087 | 0 | 0 | 0.8622 |
| haloperidol | chronic | glua1 | dorsal striatum | mrna levels | 3 | 19 | 19 | 0.653 | -0.582, 1.888 | 0.699644 | -1.497, 2.803 | 68.38 | 0.81 | 0.0599 |
| haloperidol | chronic | glua1 | frontal cortex | mrna levels | 2 | 13 | 13 | 0.03 | -0.74, 0.799 | 0.983373 | -0.74, 0.799 | 0 | 0 | 0.7232 |
| haloperidol | chronic | glua1 | subcortical areas | protein levels | 2 | 14 | 14 | -0.246 | -0.99, 0.498 | 0.892599 | -0.99, 0.498 | 0 | 0 | 0.7722 |
| haloperidol | chronic | glua1 | ventral striatum | mrna levels | 2 | 13 | 13 | 1.476 | -1.117, 4.069 | 0.643161 | -2.79, 5.742 | 84.74 | 2.99 | 0.0105 |
| haloperidol | chronic | glua2 | dorsal striatum | mrna levels | 3 | 19 | 19 | 1.198 | -1.442, 3.838 | 0.767913 | -3.864, 6.261 | 91.54 | 4.86 | 0.0012 |
| haloperidol | chronic | glua2 | frontal cortex | mrna levels | 3 | 19 | 19 | -0.401 | -1.182, 0.38 | 0.725826 | -1.471, 0.67 | 29.24 | 0.14 | 0.2514 |
| haloperidol | chronic | glua2 | ventral striatum | mrna levels | 3 | 19 | 19 | 0.455 | -1.658, 2.568 | 0.932856 | -3.567, 4.477 | 87.95 | 3.05 | 0.0003 |
| haloperidol | chronic | homer1a | associative cortex | mrna levels | 2 | 12 | 12 | -0.916 | -1.758, -0.073 | 0.207975 | -1.758, -0.073 | 0 | 0 | 0.6848 |
| haloperidol | chronic | homer1a | cingulate cortex | mrna levels | 9 | 81 | 61 | 0.139 | -0.69, 0.967 | 0.970105 | -2.192, 2.47 | 79.55 | 1.24 | 0.0001 |
| haloperidol | chronic | homer1a | dorsal striatum | mrna levels | 10 | 86 | 66 | 1.692 | 1.146, 2.238 | 1.39E-07 | 0.413, 2.972 | 46.47 | 0.35 | 0.0581 |
| haloperidol | chronic | homer1a | frontal cortex | mrna levels | 2 | 12 | 12 | -0.74 | -1.568, 0.088 | 0.340829 | -1.568, 0.088 | 0 | 0 | 0.7272 |
| haloperidol | chronic | homer1a | limbic system | mrna levels | 8 | 74 | 54 | 0.145 | -0.738, 1.027 | 0.971527 | -2.246, 2.535 | 79.91 | 1.28 | 0 |
| haloperidol | chronic | homer1a | sensory-motor areas | mrna levels | 8 | 74 | 54 | 0.296 | -0.745, 1.338 | 0.931813 | -2.579, 3.171 | 84.78 | 1.87 | 0 |
| haloperidol | chronic | homer1a | ventral striatum | mrna levels | 9 | 81 | 61 | 1.182 | 0.771, 1.593 | 6.44E-07 | 0.547, 1.817 | 15.48 | 0.06 | 0.342 |
| haloperidol | chronic | homer1a | ventral striatum | protein levels | 2 | 12 | 12 | 0.501 | -0.315, 1.317 | 0.601921 | -0.315, 1.317 | 0 | 0 | 0.5335 |
| haloperidol | chronic | homer1b-c | cingulate cortex | mrna levels | 6 | 52 | 42 | 0.025 | -0.406, 0.456 | 0.992245 | -0.406, 0.456 | 0 | 0 | 0.023 |
| haloperidol | chronic | homer1b-c | dorsal striatum | mrna levels | 6 | 52 | 42 | 0.418 | -0.213, 1.049 | 0.556095 | -0.833, 1.668 | 50.07 | 0.3 | 0.0694 |
| haloperidol | chronic | homer1b-c | limbic system | mrna levels | 6 | 52 | 42 | 0.517 | -0.294, 1.329 | 0.589911 | -1.294, 2.329 | 68.43 | 0.68 | 0.0063 |
| haloperidol | chronic | homer1b-c | sensory-motor areas | mrna levels | 6 | 52 | 42 | 0.117 | -0.309, 0.543 | 0.913839 | -0.309, 0.543 | 0 | 0 | 0.1156 |
| haloperidol | chronic | homer1b-c | ventral striatum | mrna levels | 6 | 52 | 42 | 0.684 | 0.034, 1.334 | 0.226405 | -0.623, 1.991 | 51.75 | 0.33 | 0.059 |
| haloperidol | chronic | homer1b-c | ventral striatum | protein levels | 2 | 12 | 12 | -0.13 | -1.579, 1.318 | 0.981605 | -2.349, 2.088 | 67.33 | 0.74 | 0.0802 |
| haloperidol | chronic | mglur1 | frontal cortex | mrna levels | 2 | 10 | 10 | 0.083 | -0.796, 0.962 | 0.999013 | -0.796, 0.962 | 0 | 0 | 0.6545 |
| haloperidol | chronic | mglur1 | ventral striatum | protein levels | 2 | 12 | 12 | -0.518 | -1.333, 0.296 | 0.577217 | -1.333, 0.296 | 0 | 0 | 0.7791 |
| haloperidol | chronic | mglur2 | frontal cortex | mrna levels | 3 | 15 | 15 | -0.289 | -1.013, 0.436 | 0.792666 | -1.013, 0.436 | 0 | 0 | 0.6678 |
| haloperidol | chronic | mglur5 | frontal cortex | mrna levels | 3 | 15 | 15 | 0.031 | -0.692, 0.754 | 0.980606 | -0.692, 0.754 | 0 | 0 | 0.5543 |
| haloperidol | chronic | mglur5 | frontal cortex | protein levels | 2 | 12 | 12 | 0.103 | -0.698, 0.904 | 0.978405 | -0.698, 0.904 | 0 | 0 | 0.7728 |
| haloperidol | chronic | mglur5 | ventral striatum | protein levels | 2 | 12 | 12 | 0.138 | -0.668, 0.945 | 0.979471 | -0.668, 0.945 | 0 | 0 | 0.4317 |
| haloperidol | chronic | norbin | frontal cortex | protein levels | 2 | 12 | 12 | -0.469 | -2.552, 1.614 | 0.93073 | -3.863, 2.925 | 82.72 | 1.87 | 0.0161 |
| haloperidol | chronic | norbin | ventral striatum | protein levels | 2 | 12 | 12 | 1.004 | -0.129, 2.137 | 0.344625 | -0.531, 2.539 | 41.51 | 0.28 | 0.191 |
| haloperidol | chronic | nr1 | dorsal striatum | mrna levels | 5 | 30 | 30 | 1.586 | 0.487, 2.684 | 0.040522 | -0.708, 3.879 | 68.57 | 1.06 | 0.0153 |
| haloperidol | chronic | nr1 | dorsal striatum | protein levels | 3 | 23 | 23 | 0.666 | -0.379, 1.711 | 0.58316 | -1.113, 2.445 | 63.49 | 0.54 | 0.0577 |
| haloperidol | chronic | nr1 | frontal cortex | mrna levels | 4 | 32 | 32 | 0.205 | -0.289, 0.7 | 0.77564 | -0.289, 0.7 | 0 | 0 | 0.6654 |
| haloperidol | chronic | nr1 | frontal cortex | protein levels | 4 | 29 | 29 | -0.142 | -0.983, 0.7 | 0.974625 | -1.661, 1.378 | 57.09 | 0.42 | 0.0795 |
| haloperidol | chronic | nr1 | hippocampus | mrna levels | 2 | 16 | 16 | 0.477 | -0.653, 1.606 | 0.781382 | -1.187, 2.141 | 58.14 | 0.39 | 0.1222 |
| haloperidol | chronic | nr1 | hippocampus | protein levels | 4 | 29 | 29 | -0.264 | -0.786, 0.259 | 0.736377 | -0.786, 0.259 | 0 | 0 | 0.4909 |
| haloperidol | chronic | nr1 | subcortical areas | protein levels | 2 | 14 | 14 | 0.092 | -0.65, 0.833 | 0.982633 | -0.65, 0.833 | 0 | 0 | 0.8083 |
| haloperidol | chronic | nr1 | ventral striatum | mrna levels | 4 | 25 | 25 | 0.782 | -0.364, 1.927 | 0.524689 | -1.433, 2.996 | 70.19 | 0.93 | 0.0245 |
| haloperidol | chronic | nr1 | ventral striatum | protein levels | 3 | 23 | 23 | 0.357 | -0.642, 1.357 | 0.854151 | -1.315, 2.029 | 60.1 | 0.47 | 0.084 |
| haloperidol | chronic | nr2a | dorsal striatum | mrna levels | 2 | 15 | 15 | 0.148 | -2.97, 3.267 | 0.977576 | -5.113, 5.41 | 92.28 | 4.67 | 0.0003 |
| haloperidol | chronic | nr2a | dorsal striatum | protein levels | 2 | 11 | 11 | 0.42 | -0.425, 1.265 | 0.738081 | -0.425, 1.265 | 0 | 0 | 0.899 |
| haloperidol | chronic | nr2a | frontal cortex | mrna levels | 2 | 21 | 21 | -0.544 | -1.16, 0.072 | 0.342419 | -1.16, 0.072 | 0 | 0 | 0.8752 |
| haloperidol | chronic | nr2a | frontal cortex | protein levels | 3 | 17 | 17 | -0.95 | -1.828, -0.072 | 0.206881 | -2.176, 0.275 | 31.61 | 0.19 | 0.2228 |
| haloperidol | chronic | nr2a | hippocampus | mrna levels | 2 | 21 | 21 | -0.443 | -1.056, 0.169 | 0.510777 | -1.056, 0.169 | 0 | 0 | 0.8803 |
| haloperidol | chronic | nr2a | hippocampus | protein levels | 3 | 17 | 17 | -0.551 | -1.33, 0.228 | 0.499453 | -1.527, 0.426 | 18.89 | 0.09 | 0.2559 |
| haloperidol | chronic | nr2a | ventral striatum | protein levels | 2 | 11 | 11 | 0.086 | -0.755, 0.927 | 1 | -0.755, 0.927 | 0 | 0 | 0.4828 |
| haloperidol | chronic | nr2b | dorsal striatum | mrna levels | 2 | 15 | 15 | 1.346 | -0.218, 2.909 | 0.356759 | -1.093, 3.785 | 71.56 | 0.91 | 0.0608 |
| haloperidol | chronic | nr2b | frontal cortex | protein levels | 2 | 11 | 11 | 0.35 | -0.936, 1.635 | 0.900932 | -1.508, 2.207 | 54.2 | 0.47 | 0.1395 |
| haloperidol | chronic | nr2b | hippocampus | protein levels | 3 | 17 | 17 | -1.031 | -2.132, 0.069 | 0.305576 | -2.817, 0.754 | 54.49 | 0.51 | 0.1101 |
| haloperidol | chronic | preso1 | frontal cortex | protein levels | 2 | 12 | 12 | 0.041 | -0.76, 0.842 | 0.989668 | -0.76, 0.842 | 0 | 0 | 0.73 |
| haloperidol | chronic | psd95 | cingulate cortex | mrna levels | 5 | 45 | 35 | 0.251 | -1.452, 1.954 | 0.964708 | -3.719, 4.221 | 90.03 | 3.35 | 0 |
| haloperidol | chronic | psd95 | dorsal striatum | mrna levels | 5 | 45 | 35 | 0.351 | -0.821, 1.524 | 0.931925 | -2.261, 2.964 | 81.2 | 1.42 | 0.0002 |
| haloperidol | chronic | psd95 | frontal cortex | protein levels | 3 | 16 | 16 | -0.25 | -0.947, 0.446 | 0.855731 | -0.947, 0.446 | 0 | 0 | 0.9381 |
| haloperidol | chronic | psd95 | limbic system | mrna levels | 5 | 45 | 35 | -0.301 | -1.306, 0.704 | 0.925639 | -2.478, 1.876 | 75.36 | 0.97 | 0.0016 |
| haloperidol | chronic | psd95 | sensory-motor areas | mrna levels | 5 | 45 | 35 | 0.09 | -1.156, 1.337 | 0.982568 | -2.723, 2.904 | 83.33 | 1.66 | 0.0001 |
| haloperidol | chronic | psd95 | ventral striatum | mrna levels | 5 | 45 | 35 | 0.162 | -0.739, 1.064 | 0.968337 | -1.746, 2.071 | 70.57 | 0.74 | 0.0037 |
| haloperidol | chronic | tamalin | frontal cortex | protein levels | 2 | 12 | 12 | 0.135 | -0.666, 0.936 | 0.979064 | -0.666, 0.936 | 0 | 0 | 0.9777 |
| haloperidol decanoate | chronic | glua2 | dorsal striatum | mrna levels | 2 | 13 | 13 | 0.973 | -0.106, 2.052 | 0.334929 | -0.482, 2.429 | 40.37 | 0.25 | 0.1953 |
| olanzapine | acute | arc | dorsal striatum | mrna levels | 3 | 48 | 48 | 1.41 | 0.374, 2.446 | 0.059718 | -0.482, 3.302 | 78.54 | 0.65 | 0.0162 |
| olanzapine | acute | arc | frontal cortex | mrna levels | 2 | 41 | 41 | -2.154 | -2.699, -1.61 | 1.97E-12 | -2.699, -1.61 | 0 | 0 | 0.737 |
| olanzapine | acute | arc | ventral striatum | mrna levels | 2 | 27 | 27 | 1.486 | 0.882, 2.091 | 3.66E-05 | 0.882, 2.091 | 0 | 0 | 0.387 |
| olanzapine | acute | homer1a | cingulate cortex | mrna levels | 2 | 12 | 12 | 0.701 | -0.967, 2.368 | 0.778878 | -1.918, 3.32 | 73.34 | 1.06 | 0.0528 |
| olanzapine | acute | homer1a | dorsal striatum | mrna levels | 4 | 22 | 22 | 3.442 | -1.378, 8.261 | 0.500362 | -6.974, 13.857 | 97.2 | 22.19 | 0.0001 |
| olanzapine | acute | homer1a | limbic system | mrna levels | 2 | 12 | 12 | 0.947 | -0.16, 2.053 | 0.358878 | -0.535, 2.428 | 39.54 | 0.25 | 0.1984 |
| olanzapine | acute | homer1a | sensory-motor areas | mrna levels | 2 | 12 | 12 | 0.641 | -0.674, 1.957 | 0.74427 | -1.298, 2.581 | 58.66 | 0.53 | 0.1199 |
| olanzapine | acute | homer1a | ventral striatum | mrna levels | 4 | 22 | 22 | 1.845 | 0.27, 3.419 | 0.148219 | -1.318, 5.007 | 77.74 | 1.96 | 0.0086 |
| olanzapine | acute | psd95 | dorsal striatum | mrna levels | 2 | 12 | 12 | 2.066 | -0.591, 4.723 | 0.43681 | -2.294, 6.426 | 84.44 | 3.11 | 0.0113 |
| olanzapine | acute | psd95 | frontal cortex | protein levels | 6 | 36 | 36 | 2.827 | -0.45, 6.105 | 0.360346 | -5.553, 11.208 | 96.66 | 15.49 | 0 |
| olanzapine | acute | psd95 | hippocampus | protein levels | 6 | 36 | 36 | -0.136 | -0.619, 0.348 | 0.920416 | -0.685, 0.414 | 4.85 | 0.02 | 0.3512 |
| olanzapine | acute | psd95 | ventral striatum | mrna levels | 2 | 12 | 12 | 1.438 | -1.025, 3.9 | 0.64111 | -2.608, 5.483 | 84.92 | 2.68 | 0.01 |
| olanzapine | chronic | arc | dorsal striatum | mrna levels | 2 | 19 | 19 | -1.597 | -2.328, -0.867 | 0.000414 | -2.328, -0.867 | 0 | 0 | 0.7685 |
| olanzapine | chronic | homer1a | cingulate cortex | mrna levels | 2 | 10 | 10 | 0.2 | -0.686, 1.085 | 0.935628 | -0.686, 1.085 | 0 | 0 | 0.4347 |
| olanzapine | chronic | homer1a | dorsal striatum | mrna levels | 2 | 10 | 10 | 0.342 | -0.631, 1.314 | 0.860295 | -0.767, 1.45 | 15 | 0.07 | 0.2781 |
| olanzapine | chronic | homer1a | limbic system | mrna levels | 2 | 10 | 10 | -0.245 | -1.127, 0.636 | 0.918865 | -1.127, 0.636 | 0 | 0 | 0.6992 |
| olanzapine | chronic | homer1a | sensory-motor areas | mrna levels | 2 | 10 | 10 | 0.322 | -0.789, 1.432 | 0.92739 | -1.121, 1.764 | 34.37 | 0.22 | 0.2171 |
| olanzapine | chronic | homer1a | ventral striatum | mrna levels | 2 | 10 | 10 | 1.185 | -1.506, 3.875 | 0.762792 | -3.237, 5.606 | 84.85 | 3.2 | 0.0102 |
| olanzapine | chronic | mglur5 | frontal cortex | protein levels | 2 | 12 | 12 | 0.222 | -0.581, 1.025 | 0.916642 | -0.581, 1.025 | 0 | 0 | 0.7776 |
| olanzapine | chronic | norbin | frontal cortex | protein levels | 2 | 12 | 12 | -0.039 | -0.84, 0.763 | 0.985687 | -0.84, 0.763 | 0 | 0 | 0.6702 |
| olanzapine | chronic | nr1 | frontal cortex | protein levels | 3 | 16 | 16 | 0.481 | -0.539, 1.501 | 0.757516 | -1.121, 2.083 | 48.86 | 0.4 | 0.1423 |
| olanzapine | chronic | nr1 | hippocampus | mrna levels | 2 | 11 | 11 | -0.403 | -2.188, 1.381 | 0.940764 | -3.225, 2.418 | 74.89 | 1.24 | 0.046 |
| olanzapine | chronic | nr1 | hippocampus | protein levels | 3 | 18 | 18 | -0.292 | -1.01, 0.426 | 0.787917 | -1.143, 0.559 | 13.47 | 0.05 | 0.3056 |
| olanzapine | chronic | nr2a | frontal cortex | protein levels | 2 | 12 | 12 | -0.064 | -1.2, 1.071 | 0.990386 | -1.656, 1.528 | 48.25 | 0.32 | 0.1645 |
| olanzapine | chronic | nr2a | hippocampus | mrna levels | 2 | 11 | 11 | 0.247 | -0.599, 1.093 | 0.929306 | -0.599, 1.093 | 0 | 0 | 0.3813 |
| olanzapine | chronic | nr2a | hippocampus | protein levels | 3 | 18 | 18 | -0.089 | -0.749, 0.57 | 0.976523 | -0.749, 0.57 | 0 | 0 | 0.5197 |
| olanzapine | chronic | nr2b | hippocampus | mrna levels | 2 | 11 | 11 | 0.659 | -0.203, 1.52 | 0.451975 | -0.203, 1.52 | 0 | 0 | 0.5523 |
| olanzapine | chronic | nr2b | hippocampus | protein levels | 3 | 18 | 18 | -0.278 | -1.261, 0.704 | 0.927438 | -1.856, 1.299 | 52.55 | 0.4 | 0.122 |
| olanzapine | chronic | preso1 | frontal cortex | protein levels | 2 | 12 | 12 | 0.065 | -0.736, 0.865 | 0.98326 | -0.736, 0.865 | 0 | 0 | 0.8204 |
| olanzapine | chronic | psd95 | frontal cortex | protein levels | 3 | 16 | 16 | -0.095 | -1.294, 1.103 | 0.98001 | -2.136, 1.945 | 63.3 | 0.71 | 0.0667 |
| olanzapine | chronic | tamalin | frontal cortex | protein levels | 2 | 12 | 12 | 0.399 | -0.413, 1.212 | 0.74356 | -0.413, 1.212 | 0 | 0 | 0.4663 |
| quetiapine | acute | homer1a | dorsal striatum | mrna levels | 2 | 24 | 12 | 0.436 | -0.264, 1.136 | 0.591317 | -0.264, 1.136 | 0 | 0 | 0.9673 |
| quetiapine | chronic | homer1a | dorsal striatum | mrna levels | 2 | 19 | 12 | 0.648 | -0.822, 2.119 | 0.768497 | -1.624, 2.921 | 68.25 | 0.78 | 0.076 |

## Highlight on significant results

| **drug** | **model** | **psd type** | **region** | **outcome type** | **p_val** | **adj_p_BH** |
| --- | --- | --- | --- | --- | --- | --- |
| amisulpride | chronic | arc | dorsal striatum | mrna levels | **0.005739** | **0.048036** |
| aripiprazole | chronic | homer1a | ventral striatum | protein levels | **0.029141** | 0.193701 |
| aripiprazole | chronic | norbin | ventral striatum | protein levels | **0.000334** | **0.004434** |
| aripiprazole | chronic | psd95 | frontal cortex | protein levels | **0.003852** | **0.036274** |
| asenapine | acute | homer1a | dorsal striatum | mrna levels | **0.000319** | **0.004512** |
| asenapine | chronic | homer1a | limbic system | mrna levels | **0.00433** | **0.03914** |
| clozapine | acute | homer1a | cingulate cortex | mrna levels | **3.27E-05** | **0.000671** |
| clozapine | acute | homer1a | dorsal striatum | mrna levels | **0.002231** | **0.022915** |
| clozapine | acute | homer1a | ventral striatum | mrna levels | **0.000143** | **0.002305** |
| clozapine | acute | nr1 | frontal cortex | mrna levels | **0.00288** | **0.028303** |
| clozapine | acute | nr2a | frontal cortex | mrna levels | **0.01001** | 0.072975 |
| clozapine | chronic | nr1 | dorsal striatum | protein levels | **0.032194** | 0.207881 |
| clozapine | chronic | nr1 | hippocampus | mrna levels | **0.047447** | 0.25531 |
| flupenthixol | chronic | nr1 | associative cortex | mrna levels | **4.16E-05** | **0.000784** |
| flupenthixol | chronic | nr1 | cerebellum | mrna levels | **0.00029** | **0.004371** |
| flupenthixol | chronic | nr1 | frontal cortex | mrna levels | **0.000624** | **0.007426** |
| flupenthixol | chronic | nr1 | subcortical areas | mrna levels | **0.001053** | **0.011333** |
| flupenthixol | chronic | nr2c | subcortical areas | mrna levels | **0.034748** | 0.206658 |
| flupenthixol | chronic | nr2d | cerebellum | mrna levels | **0.039945** | 0.22569 |
| haloperidol | acute | ania3 | dorsal striatum | mrna levels | **0.000519** | **0.00651** |
| haloperidol | acute | ania3 | ventral striatum | mrna levels | **0.000641** | **0.007246** |
| haloperidol | acute | arc | dorsal striatum | mrna levels | **5.27E-05** | **0.000917** |
| haloperidol | acute | arc | frontal cortex | mrna levels | **9.07E-07** | **2.56E-05** |
| haloperidol | acute | arc | ventral striatum | mrna levels | **9.9E-09** | **5.59E-07** |
| haloperidol | acute | glua1 pser845 | dorsal striatum | protein levels | **0.007588** | 0.061244 |
| haloperidol | acute | homer1a | dorsal striatum | mrna levels | **1.57E-09** | **1.18E-07** |
| haloperidol | acute | homer1a | ventral striatum | mrna levels | **1.04E-07** | **3.35E-06** |
| haloperidol | acute | homer1b-c | dorsal striatum | mrna levels | **0.046476** | 0.256182 |
| haloperidol | acute | homer1b-c | sensory-motor areas | mrna levels | **0.008378** | 0.063111 |
| haloperidol | chronic | ania3 | dorsal striatum | mrna levels | **1.08E-08** | **4.88E-07** |
| haloperidol | chronic | ania3 | ventral striatum | mrna levels | **0.01428** | 0.100854 |
| haloperidol | chronic | homer1a | associative cortex | mrna levels | **0.033129** | 0.207975 |
| haloperidol | chronic | homer1a | dorsal striatum | mrna levels | **1.23E-09** | **1.39E-07** |
| haloperidol | chronic | homer1a | ventral striatum | mrna levels | **1.71E-08** | **6.44E-07** |
| haloperidol | chronic | homer1b-c | ventral striatum | mrna levels | **0.03907** | 0.226405 |
| haloperidol | chronic | nr1 | dorsal striatum | mrna levels | **0.004662** | **0.040522** |
| haloperidol | chronic | nr2a | frontal cortex | protein levels | **0.03387** | 0.206881 |
| olanzapine | acute | arc | dorsal striatum | mrna levels | **0.007663** | 0.059718 |
| olanzapine | acute | arc | frontal cortex | mrna levels | **8.71E-15** | **1.97E-12** |
| olanzapine | acute | arc | ventral striatum | mrna levels | **1.46E-06** | **3.66E-05** |
| olanzapine | acute | homer1a | ventral striatum | mrna levels | **0.021643** | 0.148219 |
| olanzapine | chronic | arc | dorsal striatum | mrna levels | **1.83E-05** | **0.000414** |

##

Appendix 6

Leave-one-out sensitivity analyses

| **Leave-one-out sensitivity analyses** | | | | | | | | | | | |
| --- | --- | --- | --- | --- | --- | --- | --- | --- | --- | --- | --- |
| **drug** | **model** | **psd type** | **region** | **outcome type** | **author** | **estimate** | **CI** | **p_val** | **i_sq** | **tau_sq** | **q_p_val** |
| asenapine | acute | homer1a | cingulate cortex | mrna levels | -barone_2023_ | 0.076 | -1.21, 1.361 | 0.908 | 76.71 | 0.98 | 0.018 |
| asenapine | acute | homer1a | cingulate cortex | mrna levels | -barone_2023_ketamine | 0.526 | -0.168, 1.22 | 0.137 | 25.69 | 0.1 | 0.286 |
| asenapine | acute | homer1a | cingulate cortex | mrna levels | -de bartolomeis_2015 | -0.24 | -1.048, 0.569 | 0.561 | 27.96 | 0.14 | 0.23 |
| asenapine | acute | homer1a | cingulate cortex | mrna levels | -iasevoli_2020 | 0.08 | -1.25, 1.41 | 0.906 | 74.8 | 1.03 | 0.018 |
| asenapine | acute | homer1a | dorsal striatum | mrna levels | -barone_2023_ | 1.018 | 0.329, 1.706 | 0.004 | 19.85 | 0.07 | 0.306 |
| asenapine | acute | homer1a | dorsal striatum | mrna levels | -barone_2023_ketamine | 1.14 | 0.522, 1.758 | 0 | 0 | 0 | 0.443 |
| asenapine | acute | homer1a | dorsal striatum | mrna levels | -de bartolomeis_2015 | 0.72 | 0.037, 1.402 | 0.039 | 0 | 0 | 0.899 |
| asenapine | acute | homer1a | dorsal striatum | mrna levels | -iasevoli_2020 | 1.13 | 0.442, 1.817 | 0.001 | 7.74 | 0.03 | 0.382 |
| asenapine | acute | homer1a | limbic system | mrna levels | -barone_2023_ | 0.161 | -0.698, 1.02 | 0.713 | 50.35 | 0.29 | 0.133 |
| asenapine | acute | homer1a | limbic system | mrna levels | -barone_2023_ketamine | 0.546 | -0.042, 1.133 | 0.069 | 0 | 0 | 0.819 |
| asenapine | acute | homer1a | limbic system | mrna levels | -de bartolomeis_2015 | 0.037 | -0.732, 0.805 | 0.925 | 21.6 | 0.1 | 0.256 |
| asenapine | acute | homer1a | limbic system | mrna levels | -iasevoli_2020 | 0.21 | -0.737, 1.157 | 0.664 | 52.36 | 0.37 | 0.123 |
| asenapine | acute | homer1a | sensory-motor areas | mrna levels | -barone_2023_ | 0.114 | -0.952, 1.18 | 0.834 | 67.01 | 0.59 | 0.054 |
| asenapine | acute | homer1a | sensory-motor areas | mrna levels | -barone_2023_ketamine | 0.558 | -0.032, 1.148 | 0.064 | 0 | 0 | 0.527 |
| asenapine | acute | homer1a | sensory-motor areas | mrna levels | -de bartolomeis_2015 | -0.09 | -0.867, 0.688 | 0.821 | 23.01 | 0.11 | 0.247 |
| asenapine | acute | homer1a | sensory-motor areas | mrna levels | -iasevoli_2020 | 0.181 | -0.948, 1.31 | 0.753 | 65.77 | 0.65 | 0.055 |
| asenapine | acute | homer1a | ventral striatum | mrna levels | -barone_2023_ | 0.572 | -0.526, 1.671 | 0.307 | 68.62 | 0.64 | 0.042 |
| asenapine | acute | homer1a | ventral striatum | mrna levels | -barone_2023_ketamine | 0.858 | 0.073, 1.643 | 0.032 | 38.78 | 0.19 | 0.205 |
| asenapine | acute | homer1a | ventral striatum | mrna levels | -de bartolomeis_2015 | 0.194 | -0.475, 0.863 | 0.57 | 0 | 0 | 0.55 |
| asenapine | acute | homer1a | ventral striatum | mrna levels | -iasevoli_2020 | 0.606 | -0.532, 1.744 | 0.296 | 66.18 | 0.67 | 0.047 |
| clozapine | acute | arc | dorsal striatum | mrna levels | -bruins slot_2009 | -0.029 | -0.725, 0.666 | 0.934 | 21.01 | 0.07 | 0.261 |
| clozapine | acute | arc | dorsal striatum | mrna levels | -nakahara_2000 | -0.018 | -0.624, 0.588 | 0.954 | 14.77 | 0.03 | 0.279 |
| clozapine | acute | arc | dorsal striatum | mrna levels | -sakuma_2015 | -0.555 | -1.371, 0.261 | 0.182 | 0 | 0 | 0.877 |
| clozapine | acute | arc | frontal cortex | mrna levels | -bruins slot_2009 | -0.958 | -2.916, 1.001 | 0.338 | 86.4 | 1.73 | 0.007 |
| clozapine | acute | arc | frontal cortex | mrna levels | -nakahara_2000 | -1.366 | -2.525, -0.207 | 0.021 | 68.35 | 0.48 | 0.075 |
| clozapine | acute | arc | frontal cortex | mrna levels | -sakuma_2015 | -0.355 | -1.169, 0.46 | 0.393 | 0 | 0 | 0.333 |
| clozapine | acute | homer1a | dorsal striatum | mrna levels | -iasevoli_2011 | 1.125 | 0.262, 1.988 | 0.011 | 0 | 0 | 0.653 |
| clozapine | acute | homer1a | dorsal striatum | mrna levels | -polese_2002 | 1.039 | 0.186, 1.892 | 0.017 | 0 | 0 | 0.839 |
| clozapine | acute | homer1a | dorsal striatum | mrna levels | -tomasetti_2007 | 1.255 | 0.296, 2.215 | 0.01 | 0 | 0 | 0.817 |
| clozapine | acute | homer1a | ventral striatum | mrna levels | -iasevoli_2011 | 1.467 | 0.561, 2.374 | 0.002 | 0 | 0 | 0.465 |
| clozapine | acute | homer1a | ventral striatum | mrna levels | -polese_2002 | 1.66 | 0.731, 2.588 | 0 | 0 | 0 | 0.771 |
| clozapine | acute | homer1a | ventral striatum | mrna levels | -tomasetti_2007 | 1.293 | 0.327, 2.259 | 0.009 | 0 | 0 | 0.686 |
| clozapine | acute | nr1 | frontal cortex | mrna levels | -buck_2024_ | 1.388 | 0.456, 2.319 | 0.003 | 0 | 0 | 0.868 |
| clozapine | acute | nr1 | frontal cortex | mrna levels | -buck_2024_prenatal stress | 1.085 | 0.185, 1.984 | 0.018 | 0 | 0 | 0.511 |
| clozapine | acute | nr1 | frontal cortex | mrna levels | -riva_1997 | 1.067 | 0.011, 2.123 | 0.048 | 0 | 0 | 0.47 |
| clozapine | acute | nr1 | hippocampus | mrna levels | -buck_2024_ | -0.542 | -1.401, 0.317 | 0.216 | 0 | 0 | 0.367 |
| clozapine | acute | nr1 | hippocampus | mrna levels | -buck_2024_prenatal stress | -0.417 | -1.435, 0.602 | 0.423 | 26.82 | 0.15 | 0.242 |
| clozapine | acute | nr1 | hippocampus | mrna levels | -riva_1997 | 0.08 | -0.902, 1.061 | 0.874 | 0 | 0 | 0.809 |
| clozapine | acute | nr2a | frontal cortex | mrna levels | -buck_2024_ | 1.307 | 0.386, 2.227 | 0.005 | 0 | 0 | 0.943 |
| clozapine | acute | nr2a | frontal cortex | mrna levels | -buck_2024_prenatal stress | 0.89 | -0.029, 1.809 | 0.058 | 6.35 | 0.03 | 0.301 |
| clozapine | acute | nr2a | frontal cortex | mrna levels | -riva_1997 | 0.791 | -0.242, 1.824 | 0.133 | 0 | 0 | 0.334 |
| clozapine | acute | nr2a | hippocampus | mrna levels | -buck_2024_ | -0.128 | -1.185, 0.929 | 0.813 | 31.79 | 0.19 | 0.226 |
| clozapine | acute | nr2a | hippocampus | mrna levels | -buck_2024_prenatal stress | 0.337 | -0.504, 1.179 | 0.432 | 0 | 0 | 0.932 |
| clozapine | acute | nr2a | hippocampus | mrna levels | -riva_1997 | -0.19 | -1.345, 0.964 | 0.746 | 24.49 | 0.17 | 0.25 |
| clozapine | acute | nr2b | frontal cortex | mrna levels | -buck_2024_ | 0.671 | -0.192, 1.534 | 0.128 | 0 | 0 | 0.515 |
| clozapine | acute | nr2b | frontal cortex | mrna levels | -buck_2024_prenatal stress | 0.631 | -0.228, 1.49 | 0.15 | 0 | 0 | 0.603 |
| clozapine | acute | nr2b | frontal cortex | mrna levels | -riva_1997 | 1.007 | -0.034, 2.047 | 0.058 | 0 | 0 | 0.905 |
| clozapine | acute | nr2b | hippocampus | mrna levels | -buck_2024_ | -0.09 | -0.926, 0.747 | 0.834 | 0 | 0 | 0.811 |
| clozapine | acute | nr2b | hippocampus | mrna levels | -buck_2024_prenatal stress | 0.104 | -0.734, 0.941 | 0.808 | 0 | 0 | 0.716 |
| clozapine | acute | nr2b | hippocampus | mrna levels | -riva_1997 | 0.042 | -0.942, 1.027 | 0.933 | 0 | 0 | 0.594 |
| clozapine | chronic | mglur2 | frontal cortex | mrna levels | -korlatowicz_2018_ | 0.38 | -0.597, 1.357 | 0.446 | 26.56 | 0.13 | 0.243 |
| clozapine | chronic | mglur2 | frontal cortex | mrna levels | -korlatowicz_2018_ketamine | 0.266 | -0.973, 1.504 | 0.674 | 53.47 | 0.43 | 0.143 |
| clozapine | chronic | mglur2 | frontal cortex | mrna levels | -tascedda_2001 | -0.181 | -0.943, 0.58 | 0.64 | 0 | 0 | 0.737 |
| clozapine | chronic | mglur5 | frontal cortex | mrna levels | -korlatowicz_2018_ | 0.07 | -0.742, 0.881 | 0.866 | 0 | 0 | 0.953 |
| clozapine | chronic | mglur5 | frontal cortex | mrna levels | -korlatowicz_2018_ketamine | 0.097 | -0.715, 0.909 | 0.815 | 0 | 0 | 0.998 |
| clozapine | chronic | mglur5 | frontal cortex | mrna levels | -tascedda_2001 | 0.072 | -0.687, 0.831 | 0.852 | 0 | 0 | 0.951 |
| clozapine | chronic | nr1 | dorsal striatum | mrna levels | -gomes_2014_ | 1.027 | -1.637, 3.69 | 0.45 | 92.82 | 5 | 0.001 |
| clozapine | chronic | nr1 | dorsal striatum | mrna levels | -gomes_2014_mk-801 | 1.424 | -0.833, 3.682 | 0.216 | 89.75 | 3.45 | 0.003 |
| clozapine | chronic | nr1 | dorsal striatum | mrna levels | -meshul_2000 | 0.072 | -0.582, 0.726 | 0.828 | 21.98 | 0.07 | 0.251 |
| clozapine | chronic | nr1 | dorsal striatum | mrna levels | -riva_1997 | 1.289 | -1.235, 3.813 | 0.317 | 90.67 | 4.4 | 0.001 |
| clozapine | chronic | nr1 | frontal cortex | mrna levels | -gomes_2014_ | -0.03 | -0.812, 0.753 | 0.941 | 52.3 | 0.25 | 0.125 |
| clozapine | chronic | nr1 | frontal cortex | mrna levels | -gomes_2014_mk-801 | -0.097 | -0.75, 0.557 | 0.772 | 32.71 | 0.11 | 0.206 |
| clozapine | chronic | nr1 | frontal cortex | mrna levels | -riva_1997 | 0.355 | -0.488, 1.198 | 0.409 | 52.81 | 0.29 | 0.118 |
| clozapine | chronic | nr1 | frontal cortex | mrna levels | -schmitt_2003 | 0.352 | -0.508, 1.213 | 0.422 | 53.32 | 0.31 | 0.116 |
| clozapine | chronic | nr1 | hippocampus | mrna levels | -gomes_2014_ | 0.856 | -0.241, 1.952 | 0.126 | 55.34 | 0.35 | 0.135 |
| clozapine | chronic | nr1 | hippocampus | mrna levels | -gomes_2014_mk-801 | 0.362 | -0.316, 1.039 | 0.296 | 0 | 0 | 0.995 |
| clozapine | chronic | nr1 | hippocampus | mrna levels | -riva_1997 | 0.892 | -0.216, 2 | 0.114 | 48.89 | 0.31 | 0.162 |
| clozapine | chronic | nr1 | hippocampus | protein levels | -fitzgerald_1995 | -0.536 | -1.484, 0.412 | 0.268 | 41.16 | 0.29 | 0.179 |
| clozapine | chronic | nr1 | hippocampus | protein levels | -hanaoka_2003 | -0.493 | -1.38, 0.394 | 0.276 | 49.55 | 0.31 | 0.138 |
| clozapine | chronic | nr1 | hippocampus | protein levels | -krzystanek_2015 | -0.199 | -1.144, 0.747 | 0.681 | 53 | 0.37 | 0.122 |
| clozapine | chronic | nr1 | hippocampus | protein levels | -zhou_2020 | -0.008 | -0.592, 0.575 | 0.978 | 0 | 0 | 0.426 |
| haloperidol | acute | ania3 | associative cortex | mrna levels | -ambesi-impiombato_2007 | 0.223 | -1.266, 1.712 | 0.769 | 68.25 | 0.79 | 0.076 |
| haloperidol | acute | ania3 | associative cortex | mrna levels | -dell'aversano_2009 | 0.282 | -1.093, 1.656 | 0.688 | 63.06 | 0.62 | 0.1 |
| haloperidol | acute | ania3 | associative cortex | mrna levels | -tomasetti_2007 | -0.511 | -1.402, 0.38 | 0.261 | 0 | 0 | 0.898 |
| haloperidol | acute | ania3 | cingulate cortex | mrna levels | -de bartolomeis_2013 | 1.389 | -0.142, 2.92 | 0.075 | 71.18 | 1.3 | 0.025 |
| haloperidol | acute | ania3 | cingulate cortex | mrna levels | -dell'aversano_2009 | 1.371 | -0.157, 2.899 | 0.079 | 73.92 | 1.33 | 0.014 |
| haloperidol | acute | ania3 | cingulate cortex | mrna levels | -iasevoli_2010b | 0.713 | -0.765, 2.191 | 0.344 | 77.72 | 1.32 | 0.015 |
| haloperidol | acute | ania3 | cingulate cortex | mrna levels | -tomasetti_2007 | 0.52 | -0.667, 1.706 | 0.391 | 59.57 | 0.65 | 0.097 |
| haloperidol | acute | ania3 | dorsal striatum | mrna levels | -ambesi-impiombato_2007 | 2.311 | 0.614, 4.008 | 0.008 | 79.38 | 2.34 | 0.005 |
| haloperidol | acute | ania3 | dorsal striatum | mrna levels | -de bartolomeis_2013 | 2.591 | 0.743, 4.439 | 0.006 | 78.94 | 2.77 | 0.002 |
| haloperidol | acute | ania3 | dorsal striatum | mrna levels | -dell'aversano_2009 | 2.969 | 1.757, 4.18 | 0 | 48.75 | 0.74 | 0.118 |
| haloperidol | acute | ania3 | dorsal striatum | mrna levels | -iasevoli_2010b | 2.628 | 0.832, 4.423 | 0.004 | 80.55 | 2.65 | 0.002 |
| haloperidol | acute | ania3 | dorsal striatum | mrna levels | -tomasetti_2007 | 1.899 | 0.779, 3.019 | 0.001 | 54.82 | 0.71 | 0.081 |
| haloperidol | acute | ania3 | frontal cortex | mrna levels | -ambesi-impiombato_2007 | -0.175 | -1.229, 0.879 | 0.745 | 38.68 | 0.23 | 0.202 |
| haloperidol | acute | ania3 | frontal cortex | mrna levels | -dell'aversano_2009 | 0.116 | -0.687, 0.919 | 0.778 | 0 | 0 | 0.594 |
| haloperidol | acute | ania3 | frontal cortex | mrna levels | -tomasetti_2007 | -0.451 | -1.344, 0.443 | 0.323 | 0 | 0 | 0.483 |
| haloperidol | acute | ania3 | ventral striatum | mrna levels | -ambesi-impiombato_2007 | 1.425 | 0.371, 2.48 | 0.008 | 59.84 | 0.69 | 0.061 |
| haloperidol | acute | ania3 | ventral striatum | mrna levels | -de bartolomeis_2013 | 1.425 | 0.334, 2.515 | 0.01 | 59.03 | 0.73 | 0.061 |
| haloperidol | acute | ania3 | ventral striatum | mrna levels | -dell'aversano_2009 | 1.724 | 1.031, 2.417 | 0 | 0.77 | 0 | 0.323 |
| haloperidol | acute | ania3 | ventral striatum | mrna levels | -iasevoli_2010b | 1.511 | 0.48, 2.543 | 0.004 | 59.22 | 0.65 | 0.064 |
| haloperidol | acute | ania3 | ventral striatum | mrna levels | -tomasetti_2007 | 1.082 | 0.433, 1.732 | 0.001 | 0 | 0 | 0.501 |
| haloperidol | acute | arc | cingulate cortex | mrna levels | -buonaguro_2017b_ | -0.253 | -1.266, 0.759 | 0.624 | 70.01 | 0.74 | 0.015 |
| haloperidol | acute | arc | cingulate cortex | mrna levels | -buonaguro_2017b_ketamine | 0.193 | -0.968, 1.354 | 0.744 | 76.63 | 1.07 | 0.002 |
| haloperidol | acute | arc | cingulate cortex | mrna levels | -de bartolomeis_2013 | -0.249 | -1.268, 0.769 | 0.631 | 70.34 | 0.75 | 0.015 |
| haloperidol | acute | arc | cingulate cortex | mrna levels | -de bartolomeis_2015 | 0.383 | -0.503, 1.269 | 0.396 | 56.49 | 0.46 | 0.073 |
| haloperidol | acute | arc | cingulate cortex | mrna levels | -iasevoli_2010b | 0.055 | -1.134, 1.244 | 0.928 | 80.33 | 1.18 | 0.001 |
| haloperidol | acute | arc | dorsal striatum | mrna levels | -bruins slot_2009 | 2.955 | 1.822, 4.088 | 0 | 85.28 | 2.39 | 0 |
| haloperidol | acute | arc | dorsal striatum | mrna levels | -buonaguro_2017b_ | 2.387 | 1.082, 3.692 | 0 | 90.35 | 3.44 | 0 |
| haloperidol | acute | arc | dorsal striatum | mrna levels | -buonaguro_2017b_ketamine | 2.657 | 1.244, 4.069 | 0 | 91.22 | 4.05 | 0 |
| haloperidol | acute | arc | dorsal striatum | mrna levels | -de bartolomeis_2013 | 2.391 | 1.083, 3.7 | 0 | 90.4 | 3.46 | 0 |
| haloperidol | acute | arc | dorsal striatum | mrna levels | -de bartolomeis_2015 | 2.833 | 1.493, 4.173 | 0 | 88.87 | 3.56 | 0 |
| haloperidol | acute | arc | dorsal striatum | mrna levels | -fumagalli_2009 | 2.32 | 1.046, 3.594 | 0 | 88.81 | 3.18 | 0 |
| haloperidol | acute | arc | dorsal striatum | mrna levels | -iasevoli_2010b | 2.708 | 1.313, 4.103 | 0 | 91.31 | 3.96 | 0 |
| haloperidol | acute | arc | dorsal striatum | mrna levels | -nakahara_2000 | 2.368 | 1.094, 3.642 | 0 | 90.17 | 3.3 | 0 |
| haloperidol | acute | arc | dorsal striatum | mrna levels | -sakuma_2015 | 2.768 | 1.377, 4.159 | 0 | 88.71 | 3.87 | 0 |
| haloperidol | acute | arc | dorsal striatum | mrna levels | -waters_2014 | 2.596 | 1.183, 4.009 | 0 | 91.2 | 4.05 | 0 |
| haloperidol | acute | arc | frontal cortex | mrna levels | -bruins slot_2009 | -0.98 | -1.409, -0.551 | 0 | 8.48 | 0.02 | 0.285 |
| haloperidol | acute | arc | frontal cortex | mrna levels | -fumagalli_2009 | -0.872 | -1.46, -0.283 | 0.004 | 31.05 | 0.11 | 0.248 |
| haloperidol | acute | arc | frontal cortex | mrna levels | -nakahara_2000 | -1.043 | -1.44, -0.645 | 0 | 0 | 0 | 0.61 |
| haloperidol | acute | arc | frontal cortex | mrna levels | -sakuma_2015 | -0.752 | -1.205, -0.3 | 0.001 | 0 | 0 | 0.636 |
| haloperidol | acute | arc | frontal cortex | mrna levels | -waters_2014 | -0.936 | -1.405, -0.467 | 0 | 19.92 | 0.05 | 0.254 |
| haloperidol | acute | arc | limbic system | mrna levels | -buonaguro_2017b_ | -0.097 | -1.335, 1.14 | 0.878 | 79.13 | 1.25 | 0.002 |
| haloperidol | acute | arc | limbic system | mrna levels | -buonaguro_2017b_ketamine | -0.212 | -1.479, 1.054 | 0.742 | 79.84 | 1.32 | 0.002 |
| haloperidol | acute | arc | limbic system | mrna levels | -de bartolomeis_2013 | -0.708 | -1.245, -0.172 | 0.01 | 0 | 0 | 0.514 |
| haloperidol | acute | arc | limbic system | mrna levels | -de bartolomeis_2015 | 0.075 | -0.986, 1.137 | 0.889 | 69.16 | 0.81 | 0.024 |
| haloperidol | acute | arc | limbic system | mrna levels | -iasevoli_2010b | -0.121 | -1.329, 1.087 | 0.845 | 80.85 | 1.22 | 0.002 |
| haloperidol | acute | arc | sensory-motor areas | mrna levels | -buonaguro_2017b_ | -0.45 | -1.457, 0.558 | 0.382 | 69.3 | 0.73 | 0.017 |
| haloperidol | acute | arc | sensory-motor areas | mrna levels | -buonaguro_2017b_ketamine | -0.231 | -1.217, 0.754 | 0.646 | 68.64 | 0.69 | 0.018 |
| haloperidol | acute | arc | sensory-motor areas | mrna levels | -de bartolomeis_2013 | -0.717 | -1.345, -0.088 | 0.026 | 24.57 | 0.1 | 0.308 |
| haloperidol | acute | arc | sensory-motor areas | mrna levels | -de bartolomeis_2015 | -0.073 | -0.87, 0.723 | 0.856 | 47.62 | 0.31 | 0.128 |
| haloperidol | acute | arc | sensory-motor areas | mrna levels | -iasevoli_2010b | -0.327 | -1.313, 0.66 | 0.517 | 72.24 | 0.73 | 0.012 |
| haloperidol | acute | arc | ventral striatum | mrna levels | -buonaguro_2017b_ | 1.569 | 1.021, 2.117 | 0 | 31.99 | 0.15 | 0.263 |
| haloperidol | acute | arc | ventral striatum | mrna levels | -buonaguro_2017b_ketamine | 1.811 | 1.124, 2.498 | 0 | 50.83 | 0.36 | 0.061 |
| haloperidol | acute | arc | ventral striatum | mrna levels | -de bartolomeis_2013 | 1.859 | 1.218, 2.5 | 0 | 43.19 | 0.27 | 0.095 |
| haloperidol | acute | arc | ventral striatum | mrna levels | -de bartolomeis_2015 | 1.779 | 1.049, 2.508 | 0 | 52.98 | 0.43 | 0.051 |
| haloperidol | acute | arc | ventral striatum | mrna levels | -iasevoli_2010b | 1.774 | 1.103, 2.445 | 0 | 52.91 | 0.36 | 0.05 |
| haloperidol | acute | arc | ventral striatum | mrna levels | -nakahara_2000 | 1.81 | 1.142, 2.477 | 0 | 50.6 | 0.34 | 0.061 |
| haloperidol | acute | arc | ventral striatum | mrna levels | -sakuma_2015 | 1.483 | 0.984, 1.982 | 0 | 0 | 0 | 0.216 |
| haloperidol | acute | glua1 pser845 | dorsal striatum | protein levels | -håkansson_2006_ _experiment0 | 3.038 | 0.336, 5.74 | 0.028 | 90.73 | 4.97 | 0.001 |
| haloperidol | acute | glua1 pser845 | dorsal striatum | protein levels | -håkansson_2006_ _experiment1 | 2.558 | -0.303, 5.419 | 0.08 | 92.7 | 5.68 | 0.003 |
| haloperidol | acute | glua1 pser845 | dorsal striatum | protein levels | -håkansson_2006_ _experiment2 | 1.799 | 0.652, 2.946 | 0.002 | 74.49 | 0.76 | 0.024 |
| haloperidol | acute | glua1 pser845 | dorsal striatum | protein levels | -håkansson_2006_darpp-32 ko | 3.187 | 0.9, 5.474 | 0.006 | 90.08 | 3.45 | 0.003 |
| haloperidol | acute | homer1a | associative cortex | mrna levels | -de bartolomeis_2002 | -0.664 | -2.459, 1.131 | 0.469 | 75.61 | 1.28 | 0.043 |
| haloperidol | acute | homer1a | associative cortex | mrna levels | -dell'aversano_2009 | 0.171 | -0.63, 0.973 | 0.675 | 0 | 0 | 0.964 |
| haloperidol | acute | homer1a | associative cortex | mrna levels | -tomasetti_2007 | -0.713 | -2.475, 1.05 | 0.428 | 71.07 | 1.15 | 0.063 |
| haloperidol | acute | homer1a | cingulate cortex | mrna levels | -barone_2021 | 0.17 | -0.323, 0.662 | 0.5 | 47.72 | 0.3 | 0.038 |
| haloperidol | acute | homer1a | cingulate cortex | mrna levels | -buonaguro_2017b_ | 0.207 | -0.245, 0.658 | 0.369 | 42.29 | 0.22 | 0.051 |
| haloperidol | acute | homer1a | cingulate cortex | mrna levels | -buonaguro_2017b_ketamine | 0.208 | -0.243, 0.658 | 0.366 | 42.1 | 0.22 | 0.052 |
| haloperidol | acute | homer1a | cingulate cortex | mrna levels | -de bartolomeis_2013 | 0.053 | -0.319, 0.426 | 0.779 | 18.08 | 0.06 | 0.118 |
| haloperidol | acute | homer1a | cingulate cortex | mrna levels | -de bartolomeis_2015 | 0.162 | -0.327, 0.651 | 0.516 | 48.25 | 0.29 | 0.037 |
| haloperidol | acute | homer1a | cingulate cortex | mrna levels | -dell'aversano_2009 | 0.182 | -0.277, 0.64 | 0.438 | 45.76 | 0.24 | 0.04 |
| haloperidol | acute | homer1a | cingulate cortex | mrna levels | -iasevoli_2010b | 0.24 | -0.133, 0.612 | 0.207 | 21.9 | 0.08 | 0.183 |
| haloperidol | acute | homer1a | cingulate cortex | mrna levels | -iasevoli_2011 | 0.127 | -0.336, 0.59 | 0.591 | 46.78 | 0.25 | 0.038 |
| haloperidol | acute | homer1a | cingulate cortex | mrna levels | -iasevoli_2020 | 0.163 | -0.315, 0.641 | 0.504 | 48.04 | 0.28 | 0.037 |
| haloperidol | acute | homer1a | cingulate cortex | mrna levels | -tomasetti_2007 | 0.103 | -0.357, 0.564 | 0.66 | 44.6 | 0.24 | 0.046 |
| haloperidol | acute | homer1a | cingulate cortex | mrna levels | -tomasetti_2011 | 0.036 | -0.295, 0.368 | 0.83 | 0 | 0 | 0.227 |
| haloperidol | acute | homer1a | dorsal striatum | mrna levels | -ambesi-impiombato_2007 | 2.182 | 1.426, 2.938 | 0 | 73.77 | 1.36 | 0 |
| haloperidol | acute | homer1a | dorsal striatum | mrna levels | -barone_2021 | 2.25 | 1.59, 2.91 | 0 | 61.13 | 0.88 | 0 |
| haloperidol | acute | homer1a | dorsal striatum | mrna levels | -buonaguro_2017b_ | 2.142 | 1.385, 2.898 | 0 | 73.49 | 1.35 | 0 |
| haloperidol | acute | homer1a | dorsal striatum | mrna levels | -buonaguro_2017b_ketamine | 2.236 | 1.622, 2.849 | 0 | 58.01 | 0.71 | 0 |
| haloperidol | acute | homer1a | dorsal striatum | mrna levels | -de bartolomeis_2002 | 2.09 | 1.359, 2.822 | 0 | 72.62 | 1.25 | 0 |
| haloperidol | acute | homer1a | dorsal striatum | mrna levels | -de bartolomeis_2013 | 2.12 | 1.37, 2.869 | 0 | 73.15 | 1.32 | 0 |
| haloperidol | acute | homer1a | dorsal striatum | mrna levels | -de bartolomeis_2015 | 2.168 | 1.399, 2.936 | 0 | 72.95 | 1.39 | 0 |
| haloperidol | acute | homer1a | dorsal striatum | mrna levels | -dell'aversano_2009 | 2.14 | 1.39, 2.89 | 0 | 73.66 | 1.33 | 0 |
| haloperidol | acute | homer1a | dorsal striatum | mrna levels | -iasevoli_2010a | 2.016 | 1.359, 2.672 | 0 | 69.07 | 1.01 | 0 |
| haloperidol | acute | homer1a | dorsal striatum | mrna levels | -iasevoli_2010b | 2.06 | 1.349, 2.772 | 0 | 71.65 | 1.18 | 0 |
| haloperidol | acute | homer1a | dorsal striatum | mrna levels | -iasevoli_2011 | 1.908 | 1.296, 2.52 | 0 | 63.29 | 0.78 | 0 |
| haloperidol | acute | homer1a | dorsal striatum | mrna levels | -iasevoli_2020 | 2.225 | 1.474, 2.975 | 0 | 72.02 | 1.31 | 0 |
| haloperidol | acute | homer1a | dorsal striatum | mrna levels | -polese_2002 | 2.133 | 1.385, 2.881 | 0 | 73.56 | 1.33 | 0 |
| haloperidol | acute | homer1a | dorsal striatum | mrna levels | -tomasetti_2007 | 2.122 | 1.372, 2.873 | 0 | 73.2 | 1.33 | 0 |
| haloperidol | acute | homer1a | dorsal striatum | mrna levels | -tomasetti_2011 | 2.01 | 1.316, 2.705 | 0 | 69.42 | 1.08 | 0 |
| haloperidol | acute | homer1a | frontal cortex | mrna levels | -de bartolomeis_2002 | -0.098 | -1.063, 0.866 | 0.842 | 27.82 | 0.14 | 0.239 |
| haloperidol | acute | homer1a | frontal cortex | mrna levels | -dell'aversano_2009 | 0.311 | -0.494, 1.116 | 0.449 | 0 | 0 | 0.952 |
| haloperidol | acute | homer1a | frontal cortex | mrna levels | -tomasetti_2007 | -0.18 | -1.104, 0.744 | 0.702 | 7.12 | 0.03 | 0.299 |
| haloperidol | acute | homer1a | limbic system | mrna levels | -barone_2021 | -0.597 | -1.313, 0.119 | 0.102 | 67.53 | 0.7 | 0.005 |
| haloperidol | acute | homer1a | limbic system | mrna levels | -buonaguro_2017b_ | -0.305 | -0.848, 0.238 | 0.271 | 50.66 | 0.3 | 0.022 |
| haloperidol | acute | homer1a | limbic system | mrna levels | -buonaguro_2017b_ketamine | -0.527 | -1.255, 0.201 | 0.156 | 71.43 | 0.75 | 0.003 |
| haloperidol | acute | homer1a | limbic system | mrna levels | -de bartolomeis_2013 | -0.443 | -1.138, 0.252 | 0.212 | 68.98 | 0.66 | 0.005 |
| haloperidol | acute | homer1a | limbic system | mrna levels | -de bartolomeis_2015 | -0.561 | -1.296, 0.173 | 0.134 | 70.18 | 0.76 | 0.003 |
| haloperidol | acute | homer1a | limbic system | mrna levels | -iasevoli_2010b | -0.303 | -0.812, 0.205 | 0.242 | 48.81 | 0.26 | 0.057 |
| haloperidol | acute | homer1a | limbic system | mrna levels | -iasevoli_2011 | -0.601 | -1.271, 0.069 | 0.079 | 67.6 | 0.6 | 0.005 |
| haloperidol | acute | homer1a | limbic system | mrna levels | -iasevoli_2020 | -0.628 | -1.201, -0.055 | 0.032 | 53.69 | 0.35 | 0.023 |
| haloperidol | acute | homer1a | limbic system | mrna levels | -tomasetti_2011 | -0.48 | -1.197, 0.236 | 0.189 | 70.69 | 0.72 | 0.004 |
| haloperidol | acute | homer1a | sensory-motor areas | mrna levels | -barone_2021 | -0.121 | -0.692, 0.45 | 0.678 | 51.95 | 0.34 | 0.031 |
| haloperidol | acute | homer1a | sensory-motor areas | mrna levels | -buonaguro_2017b_ | 0.04 | -0.333, 0.413 | 0.834 | 3.7 | 0.01 | 0.079 |
| haloperidol | acute | homer1a | sensory-motor areas | mrna levels | -buonaguro_2017b_ketamine | -0.034 | -0.539, 0.472 | 0.896 | 44.23 | 0.23 | 0.041 |
| haloperidol | acute | homer1a | sensory-motor areas | mrna levels | -de bartolomeis_2013 | -0.166 | -0.53, 0.199 | 0.372 | 0 | 0 | 0.105 |
| haloperidol | acute | homer1a | sensory-motor areas | mrna levels | -de bartolomeis_2015 | -0.093 | -0.654, 0.469 | 0.747 | 51.91 | 0.33 | 0.031 |
| haloperidol | acute | homer1a | sensory-motor areas | mrna levels | -iasevoli_2010b | 0.033 | -0.319, 0.385 | 0.853 | 0 | 0 | 0.308 |
| haloperidol | acute | homer1a | sensory-motor areas | mrna levels | -iasevoli_2011 | -0.157 | -0.638, 0.324 | 0.522 | 41.16 | 0.19 | 0.048 |
| haloperidol | acute | homer1a | sensory-motor areas | mrna levels | -iasevoli_2020 | -0.155 | -0.674, 0.365 | 0.559 | 46.6 | 0.25 | 0.04 |
| haloperidol | acute | homer1a | sensory-motor areas | mrna levels | -tomasetti_2011 | -0.126 | -0.667, 0.416 | 0.649 | 51.06 | 0.3 | 0.032 |
| haloperidol | acute | homer1a | ventral striatum | mrna levels | -barone_2021 | 1.949 | 1.285, 2.612 | 0 | 65.87 | 0.92 | 0.001 |
| haloperidol | acute | homer1a | ventral striatum | mrna levels | -buonaguro_2017b_ | 1.885 | 1.156, 2.615 | 0 | 74.24 | 1.24 | 0 |
| haloperidol | acute | homer1a | ventral striatum | mrna levels | -buonaguro_2017b_ketamine | 1.945 | 1.342, 2.548 | 0 | 61.4 | 0.7 | 0.001 |
| haloperidol | acute | homer1a | ventral striatum | mrna levels | -de bartolomeis_2002 | 1.815 | 1.093, 2.537 | 0 | 74.88 | 1.23 | 0 |
| haloperidol | acute | homer1a | ventral striatum | mrna levels | -de bartolomeis_2013 | 1.878 | 1.147, 2.61 | 0 | 74.42 | 1.25 | 0 |
| haloperidol | acute | homer1a | ventral striatum | mrna levels | -de bartolomeis_2015 | 1.844 | 1.105, 2.584 | 0 | 74.12 | 1.28 | 0 |
| haloperidol | acute | homer1a | ventral striatum | mrna levels | -dell'aversano_2009 | 1.724 | 1.045, 2.404 | 0 | 72.2 | 1.05 | 0 |
| haloperidol | acute | homer1a | ventral striatum | mrna levels | -iasevoli_2010a | 1.622 | 1.021, 2.223 | 0 | 65.74 | 0.76 | 0 |
| haloperidol | acute | homer1a | ventral striatum | mrna levels | -iasevoli_2010b | 1.747 | 1.059, 2.435 | 0 | 73.14 | 1.1 | 0 |
| haloperidol | acute | homer1a | ventral striatum | mrna levels | -iasevoli_2011 | 1.733 | 1.048, 2.418 | 0 | 72.6 | 1.08 | 0 |
| haloperidol | acute | homer1a | ventral striatum | mrna levels | -iasevoli_2020 | 1.864 | 1.129, 2.599 | 0 | 74.53 | 1.27 | 0 |
| haloperidol | acute | homer1a | ventral striatum | mrna levels | -polese_2002 | 1.861 | 1.133, 2.59 | 0 | 74.97 | 1.25 | 0 |
| haloperidol | acute | homer1a | ventral striatum | mrna levels | -tomasetti_2007 | 1.823 | 1.093, 2.553 | 0 | 74.75 | 1.25 | 0 |
| haloperidol | acute | homer1a | ventral striatum | mrna levels | -tomasetti_2011 | 1.68 | 1.022, 2.337 | 0 | 69.8 | 0.95 | 0 |
| haloperidol | acute | homer1b-c | cingulate cortex | mrna levels | -buonaguro_2017b_ | 0.949 | 0.255, 1.642 | 0.007 | 0 | 0 | 0.985 |
| haloperidol | acute | homer1b-c | cingulate cortex | mrna levels | -buonaguro_2017b_ketamine | 0.319 | -0.965, 1.604 | 0.626 | 71.15 | 0.61 | 0.063 |
| haloperidol | acute | homer1b-c | cingulate cortex | mrna levels | -de bartolomeis_2015 | 0.283 | -1.016, 1.583 | 0.669 | 65.36 | 0.57 | 0.089 |
| haloperidol | acute | homer1b-c | dorsal striatum | mrna levels | -buonaguro_2017b_ | 1.029 | 0.33, 1.727 | 0.004 | 0 | 0 | 0.924 |
| haloperidol | acute | homer1b-c | dorsal striatum | mrna levels | -buonaguro_2017b_ketamine | 0.548 | -0.514, 1.61 | 0.312 | 57.95 | 0.34 | 0.123 |
| haloperidol | acute | homer1b-c | dorsal striatum | mrna levels | -de bartolomeis_2015 | 0.462 | -0.534, 1.457 | 0.364 | 41.35 | 0.21 | 0.192 |
| haloperidol | acute | homer1b-c | limbic system | mrna levels | -buonaguro_2017b_ | 0.889 | 0.199, 1.578 | 0.012 | 0 | 0 | 0.975 |
| haloperidol | acute | homer1b-c | limbic system | mrna levels | -buonaguro_2017b_ketamine | 0.253 | -1.04, 1.545 | 0.702 | 71.52 | 0.62 | 0.061 |
| haloperidol | acute | homer1b-c | limbic system | mrna levels | -de bartolomeis_2015 | 0.222 | -1.094, 1.539 | 0.741 | 66.36 | 0.6 | 0.085 |
| haloperidol | acute | homer1b-c | sensory-motor areas | mrna levels | -buonaguro_2017b_ | 0.935 | 0.242, 1.627 | 0.008 | 0 | 0 | 0.493 |
| haloperidol | acute | homer1b-c | sensory-motor areas | mrna levels | -buonaguro_2017b_ketamine | 0.833 | 0.133, 1.532 | 0.02 | 2.97 | 0.01 | 0.31 |
| haloperidol | acute | homer1b-c | sensory-motor areas | mrna levels | -de bartolomeis_2015 | 0.531 | -0.224, 1.285 | 0.168 | 0 | 0 | 0.765 |
| haloperidol | acute | homer1b-c | ventral striatum | mrna levels | -buonaguro_2017b_ | 1.091 | 0.388, 1.793 | 0.002 | 0 | 0 | 0.77 |
| haloperidol | acute | homer1b-c | ventral striatum | mrna levels | -buonaguro_2017b_ketamine | 0.467 | -0.972, 1.906 | 0.525 | 76.66 | 0.83 | 0.038 |
| haloperidol | acute | homer1b-c | ventral striatum | mrna levels | -de bartolomeis_2015 | 0.323 | -0.907, 1.553 | 0.607 | 61.46 | 0.48 | 0.107 |
| haloperidol | acute | nr1 | frontal cortex | mrna levels | -buck_2024_ | 0.324 | -0.518, 1.166 | 0.451 | 0 | 0 | 0.748 |
| haloperidol | acute | nr1 | frontal cortex | mrna levels | -buck_2024_prenatal stress | 0.184 | -0.659, 1.027 | 0.669 | 0 | 0 | 0.454 |
| haloperidol | acute | nr1 | frontal cortex | mrna levels | -riva_1997 | -0.047 | -1.03, 0.935 | 0.925 | 0 | 0 | 0.704 |
| haloperidol | acute | nr1 | hippocampus | mrna levels | -buck_2024_ | -0.024 | -0.86, 0.812 | 0.956 | 0 | 0 | 0.889 |
| haloperidol | acute | nr1 | hippocampus | mrna levels | -buck_2024_prenatal stress | -0.111 | -0.948, 0.725 | 0.794 | 0 | 0 | 0.895 |
| haloperidol | acute | nr1 | hippocampus | mrna levels | -riva_1997 | -0.065 | -1.046, 0.916 | 0.896 | 0 | 0 | 0.81 |
| haloperidol | acute | nr2a | frontal cortex | mrna levels | -buck_2024_ | 0.36 | -0.784, 1.505 | 0.537 | 41.13 | 0.29 | 0.192 |
| haloperidol | acute | nr2a | frontal cortex | mrna levels | -buck_2024_prenatal stress | 0.245 | -1.111, 1.602 | 0.723 | 57.17 | 0.55 | 0.127 |
| haloperidol | acute | nr2a | frontal cortex | mrna levels | -riva_1997 | -0.417 | -1.408, 0.575 | 0.41 | 0 | 0 | 0.834 |
| haloperidol | acute | nr2a | hippocampus | mrna levels | -buck_2024_ | 0.784 | -0.534, 2.103 | 0.244 | 52.99 | 0.48 | 0.145 |
| haloperidol | acute | nr2a | hippocampus | mrna levels | -buck_2024_prenatal stress | 0.362 | -1.763, 2.488 | 0.738 | 81.05 | 1.91 | 0.022 |
| haloperidol | acute | nr2a | hippocampus | mrna levels | -riva_1997 | -0.34 | -1.337, 0.657 | 0.504 | 0 | 0 | 0.42 |
| haloperidol | acute | nr2b | frontal cortex | mrna levels | -buck_2024_ | 0.288 | -0.556, 1.133 | 0.504 | 0 | 0 | 0.499 |
| haloperidol | acute | nr2b | frontal cortex | mrna levels | -buck_2024_prenatal stress | 0.215 | -0.631, 1.061 | 0.619 | 0 | 0 | 0.368 |
| haloperidol | acute | nr2b | frontal cortex | mrna levels | -riva_1997 | -0.192 | -1.175, 0.791 | 0.701 | 0 | 0 | 0.84 |
| haloperidol | acute | nr2b | hippocampus | mrna levels | -buck_2024_ | 0.608 | -0.408, 1.624 | 0.241 | 25.15 | 0.14 | 0.248 |
| haloperidol | acute | nr2b | hippocampus | mrna levels | -buck_2024_prenatal stress | 0.669 | -0.242, 1.58 | 0.15 | 8.1 | 0.04 | 0.297 |
| haloperidol | acute | nr2b | hippocampus | mrna levels | -riva_1997 | 0.051 | -0.929, 1.031 | 0.919 | 0 | 0 | 0.919 |
| haloperidol | acute | psd95 | dorsal striatum | mrna levels | -de bartolomeis_2002 | 0.141 | -1.675, 1.956 | 0.879 | 84.89 | 1.46 | 0.01 |
| haloperidol | acute | psd95 | dorsal striatum | mrna levels | -de bartolomeis_2015 | 0.02 | -1.686, 1.726 | 0.982 | 75.23 | 1.14 | 0.045 |
| haloperidol | acute | psd95 | dorsal striatum | mrna levels | -tomasetti_2011 | 1.005 | 0.265, 1.745 | 0.008 | 0 | 0 | 0.891 |
| haloperidol | acute | psd95 | ventral striatum | mrna levels | -de bartolomeis_2002 | -0.695 | -3.025, 1.634 | 0.558 | 89.16 | 2.52 | 0.002 |
| haloperidol | acute | psd95 | ventral striatum | mrna levels | -de bartolomeis_2015 | -0.595 | -3.219, 2.03 | 0.657 | 88.2 | 3.16 | 0.004 |
| haloperidol | acute | psd95 | ventral striatum | mrna levels | -tomasetti_2011 | 0.54 | -0.176, 1.256 | 0.139 | 0 | 0 | 0.704 |
| haloperidol | chronic | ania3 | dorsal striatum | mrna levels | -ambesi-impiombato_2007 | 3.384 | 2.081, 4.687 | 0 | 0 | 0 | 0.999 |
| haloperidol | chronic | ania3 | dorsal striatum | mrna levels | -iasevoli_2010b | 2.82 | 1.679, 3.96 | 0 | 0 | 0 | 0.344 |
| haloperidol | chronic | ania3 | dorsal striatum | mrna levels | -tomasetti_2007 | 2.67 | 1.388, 3.952 | 0 | 0 | 0 | 0.422 |
| haloperidol | chronic | arc | cingulate cortex | mrna levels | -buonaguro_2017a | -0.127 | -0.932, 0.678 | 0.757 | 0 | 0 | 0.471 |
| haloperidol | chronic | arc | cingulate cortex | mrna levels | -de bartolomeis_2016_ | -1.147 | -2.57, 0.275 | 0.114 | 66.99 | 0.71 | 0.082 |
| haloperidol | chronic | arc | cingulate cortex | mrna levels | -de bartolomeis_2016_pretreat_chr_hal | -0.851 | -2.854, 1.153 | 0.405 | 83.51 | 1.75 | 0.014 |
| haloperidol | chronic | arc | dorsal striatum | mrna levels | -buonaguro_2017a | 0.854 | -1.244, 2.951 | 0.425 | 89.64 | 3.06 | 0 |
| haloperidol | chronic | arc | dorsal striatum | mrna levels | -de bartolomeis_2016_ | 0.245 | -1.525, 2.014 | 0.786 | 88.43 | 2.14 | 0 |
| haloperidol | chronic | arc | dorsal striatum | mrna levels | -de bartolomeis_2016_pretreat_chr_hal | 0.213 | -1.501, 1.926 | 0.808 | 87.79 | 1.99 | 0.001 |
| haloperidol | chronic | arc | dorsal striatum | mrna levels | -fumagalli_2009 | 1.261 | -0.025, 2.548 | 0.055 | 68.61 | 0.88 | 0.034 |
| haloperidol | chronic | arc | limbic system | mrna levels | -buonaguro_2017a | -0.074 | -0.874, 0.727 | 0.857 | 0 | 0 | 0.856 |
| haloperidol | chronic | arc | limbic system | mrna levels | -de bartolomeis_2016_ | -0.858 | -2.545, 0.828 | 0.319 | 77.24 | 1.14 | 0.036 |
| haloperidol | chronic | arc | limbic system | mrna levels | -de bartolomeis_2016_pretreat_chr_hal | -0.932 | -2.474, 0.609 | 0.236 | 72.73 | 0.9 | 0.055 |
| haloperidol | chronic | arc | sensory-motor areas | mrna levels | -buonaguro_2017a | -0.371 | -1.178, 0.436 | 0.368 | 0 | 0 | 0.855 |
| haloperidol | chronic | arc | sensory-motor areas | mrna levels | -de bartolomeis_2016_ | -1.585 | -4.163, 0.993 | 0.228 | 88.19 | 3.05 | 0.004 |
| haloperidol | chronic | arc | sensory-motor areas | mrna levels | -de bartolomeis_2016_pretreat_chr_hal | -1.659 | -4.09, 0.772 | 0.181 | 86.64 | 2.67 | 0.006 |
| haloperidol | chronic | arc | ventral striatum | mrna levels | -buonaguro_2017a | 0.464 | -0.35, 1.277 | 0.264 | 0 | 0 | 0.588 |
| haloperidol | chronic | arc | ventral striatum | mrna levels | -de bartolomeis_2016_ | 0.347 | -0.412, 1.107 | 0.37 | 0 | 0 | 0.811 |
| haloperidol | chronic | arc | ventral striatum | mrna levels | -de bartolomeis_2016_pretreat_chr_hal | 0.545 | -0.223, 1.313 | 0.164 | 0 | 0 | 0.738 |
| haloperidol | chronic | glua1 | dorsal striatum | mrna levels | -brené_1998 | 0.079 | -0.662, 0.821 | 0.834 | 0 | 0 | 0.919 |
| haloperidol | chronic | glua1 | dorsal striatum | mrna levels | -eastwood_1994 | 1.049 | -0.961, 3.058 | 0.306 | 79.09 | 1.68 | 0.029 |
| haloperidol | chronic | glua1 | dorsal striatum | mrna levels | -healy_1997 | 1.033 | -1.057, 3.122 | 0.333 | 78.78 | 1.8 | 0.03 |
| haloperidol | chronic | glua2 | dorsal striatum | mrna levels | -brené_1998 | -0.099 | -0.843, 0.646 | 0.795 | 0 | 0 | 0.502 |
| haloperidol | chronic | glua2 | dorsal striatum | mrna levels | -eastwood_1994 | 1.831 | -2.604, 6.267 | 0.418 | 92.5 | 9.5 | 0 |
| haloperidol | chronic | glua2 | dorsal striatum | mrna levels | -healy_1997 | 2.084 | -1.845, 6.014 | 0.298 | 89.95 | 7.26 | 0.002 |
| haloperidol | chronic | glua2 | frontal cortex | mrna levels | -brené_1998 | -0.623 | -1.621, 0.376 | 0.222 | 40.14 | 0.21 | 0.196 |
| haloperidol | chronic | glua2 | frontal cortex | mrna levels | -eastwood_1994 | -0.529 | -1.753, 0.694 | 0.396 | 55.98 | 0.44 | 0.132 |
| haloperidol | chronic | glua2 | frontal cortex | mrna levels | -healy_1997 | 0.015 | -0.822, 0.851 | 0.973 | 0 | 0 | 0.787 |
| haloperidol | chronic | glua2 | ventral striatum | mrna levels | -brené_1998 | -0.364 | -2.578, 1.85 | 0.747 | 86.73 | 2.21 | 0.006 |
| haloperidol | chronic | glua2 | ventral striatum | mrna levels | -eastwood_1994 | 0.333 | -3.316, 3.982 | 0.858 | 93.01 | 6.45 | 0 |
| haloperidol | chronic | glua2 | ventral striatum | mrna levels | -healy_1997 | 1.407 | -0.016, 2.83 | 0.053 | 53.02 | 0.57 | 0.145 |
| haloperidol | chronic | homer1a | cingulate cortex | mrna levels | -buonaguro_2017a | 0.315 | -0.544, 1.173 | 0.472 | 78.29 | 1.16 | 0 |
| haloperidol | chronic | homer1a | cingulate cortex | mrna levels | -de bartolomeis_2016_ | 0.002 | -0.877, 0.881 | 0.996 | 79.99 | 1.24 | 0 |
| haloperidol | chronic | homer1a | cingulate cortex | mrna levels | -de bartolomeis_2016_pretreat_chr_hal | 0.157 | -0.809, 1.123 | 0.75 | 83.12 | 1.56 | 0 |
| haloperidol | chronic | homer1a | cingulate cortex | mrna levels | -iasevoli_2010b | -0.114 | -0.798, 0.571 | 0.745 | 70.18 | 0.68 | 0.001 |
| haloperidol | chronic | homer1a | cingulate cortex | mrna levels | -iasevoli_2011 | 0.04 | -0.892, 0.971 | 0.933 | 79.64 | 1.4 | 0 |
| haloperidol | chronic | homer1a | cingulate cortex | mrna levels | -iasevoli_2014 | 0.144 | -0.824, 1.113 | 0.77 | 82.87 | 1.56 | 0 |
| haloperidol | chronic | homer1a | cingulate cortex | mrna levels | -iasevoli_2020 | 0.156 | -0.815, 1.128 | 0.752 | 82.8 | 1.57 | 0 |
| haloperidol | chronic | homer1a | cingulate cortex | mrna levels | -tomasetti_2007 | 0.291 | -0.606, 1.188 | 0.525 | 80.27 | 1.3 | 0 |
| haloperidol | chronic | homer1a | cingulate cortex | mrna levels | -tomasetti_2011 | 0.328 | -0.493, 1.148 | 0.434 | 76.72 | 1.03 | 0.001 |
| haloperidol | chronic | homer1a | dorsal striatum | mrna levels | -ambesi-impiombato_2007 | 1.644 | 1.066, 2.222 | 0 | 49.36 | 0.37 | 0.048 |
| haloperidol | chronic | homer1a | dorsal striatum | mrna levels | -buonaguro_2017a | 1.776 | 1.171, 2.381 | 0 | 49.49 | 0.41 | 0.048 |
| haloperidol | chronic | homer1a | dorsal striatum | mrna levels | -de bartolomeis_2016_ | 1.664 | 1.071, 2.258 | 0 | 50.71 | 0.4 | 0.042 |
| haloperidol | chronic | homer1a | dorsal striatum | mrna levels | -de bartolomeis_2016_pretreat_chr_hal | 1.73 | 1.122, 2.337 | 0 | 51.92 | 0.43 | 0.037 |
| haloperidol | chronic | homer1a | dorsal striatum | mrna levels | -iasevoli_2010b | 1.676 | 1.091, 2.262 | 0 | 51.12 | 0.4 | 0.039 |
| haloperidol | chronic | homer1a | dorsal striatum | mrna levels | -iasevoli_2011 | 1.71 | 1.08, 2.341 | 0 | 50.72 | 0.46 | 0.037 |
| haloperidol | chronic | homer1a | dorsal striatum | mrna levels | -iasevoli_2014 | 1.635 | 1.05, 2.22 | 0 | 48.79 | 0.38 | 0.052 |
| haloperidol | chronic | homer1a | dorsal striatum | mrna levels | -iasevoli_2020 | 1.868 | 1.448, 2.289 | 0 | 0 | 0 | 0.689 |
| haloperidol | chronic | homer1a | dorsal striatum | mrna levels | -tomasetti_2007 | 1.674 | 1.072, 2.277 | 0 | 51.14 | 0.42 | 0.041 |
| haloperidol | chronic | homer1a | dorsal striatum | mrna levels | -tomasetti_2011 | 1.555 | 1.028, 2.082 | 0 | 39.08 | 0.25 | 0.132 |
| haloperidol | chronic | homer1a | limbic system | mrna levels | -buonaguro_2017a | 0.328 | -0.605, 1.262 | 0.491 | 79.19 | 1.25 | 0 |
| haloperidol | chronic | homer1a | limbic system | mrna levels | -de bartolomeis_2016_ | 0.131 | -0.886, 1.147 | 0.801 | 82.77 | 1.55 | 0 |
| haloperidol | chronic | homer1a | limbic system | mrna levels | -de bartolomeis_2016_pretreat_chr_hal | 0.203 | -0.805, 1.211 | 0.693 | 82.49 | 1.51 | 0 |
| haloperidol | chronic | homer1a | limbic system | mrna levels | -iasevoli_2010b | 0.023 | -0.94, 0.985 | 0.963 | 82.05 | 1.38 | 0 |
| haloperidol | chronic | homer1a | limbic system | mrna levels | -iasevoli_2011 | -0.221 | -0.845, 0.403 | 0.488 | 52.09 | 0.37 | 0.05 |
| haloperidol | chronic | homer1a | limbic system | mrna levels | -iasevoli_2014 | 0.134 | -0.886, 1.155 | 0.796 | 82.51 | 1.55 | 0 |
| haloperidol | chronic | homer1a | limbic system | mrna levels | -iasevoli_2020 | 0.15 | -0.873, 1.172 | 0.774 | 82.37 | 1.56 | 0 |
| haloperidol | chronic | homer1a | limbic system | mrna levels | -tomasetti_2011 | 0.388 | -0.47, 1.245 | 0.376 | 76.16 | 1.01 | 0 |
| haloperidol | chronic | homer1a | sensory-motor areas | mrna levels | -buonaguro_2017a | 0.44 | -0.742, 1.622 | 0.466 | 86.02 | 2.14 | 0 |
| haloperidol | chronic | homer1a | sensory-motor areas | mrna levels | -de bartolomeis_2016_ | 0.201 | -0.989, 1.392 | 0.741 | 86.84 | 2.19 | 0 |
| haloperidol | chronic | homer1a | sensory-motor areas | mrna levels | -de bartolomeis_2016_pretreat_chr_hal | 0.418 | -0.773, 1.609 | 0.491 | 86.68 | 2.19 | 0 |
| haloperidol | chronic | homer1a | sensory-motor areas | mrna levels | -iasevoli_2010b | 0.012 | -0.944, 0.967 | 0.981 | 81.69 | 1.35 | 0 |
| haloperidol | chronic | homer1a | sensory-motor areas | mrna levels | -iasevoli_2011 | 0.052 | -1.02, 1.125 | 0.924 | 81.99 | 1.68 | 0 |
| haloperidol | chronic | homer1a | sensory-motor areas | mrna levels | -iasevoli_2014 | 0.263 | -0.956, 1.482 | 0.673 | 87.01 | 2.3 | 0 |
| haloperidol | chronic | homer1a | sensory-motor areas | mrna levels | -iasevoli_2020 | 0.379 | -0.835, 1.594 | 0.54 | 86.69 | 2.28 | 0 |
| haloperidol | chronic | homer1a | sensory-motor areas | mrna levels | -tomasetti_2011 | 0.614 | -0.263, 1.491 | 0.17 | 76.25 | 1.04 | 0 |
| haloperidol | chronic | homer1a | ventral striatum | mrna levels | -buonaguro_2017a | 1.156 | 0.694, 1.619 | 0 | 23.93 | 0.11 | 0.262 |
| haloperidol | chronic | homer1a | ventral striatum | mrna levels | -de bartolomeis_2016_ | 1.259 | 0.827, 1.691 | 0 | 14.38 | 0.06 | 0.362 |
| haloperidol | chronic | homer1a | ventral striatum | mrna levels | -de bartolomeis_2016_pretreat_chr_hal | 1.245 | 0.803, 1.686 | 0 | 17.98 | 0.07 | 0.322 |
| haloperidol | chronic | homer1a | ventral striatum | mrna levels | -iasevoli_2010b | 1.162 | 0.723, 1.6 | 0 | 21.42 | 0.09 | 0.263 |
| haloperidol | chronic | homer1a | ventral striatum | mrna levels | -iasevoli_2011 | 1.052 | 0.637, 1.468 | 0 | 0 | 0 | 0.439 |
| haloperidol | chronic | homer1a | ventral striatum | mrna levels | -iasevoli_2014 | 1.089 | 0.681, 1.497 | 0 | 7.67 | 0.03 | 0.5 |
| haloperidol | chronic | homer1a | ventral striatum | mrna levels | -iasevoli_2020 | 1.312 | 0.908, 1.715 | 0 | 0 | 0 | 0.52 |
| haloperidol | chronic | homer1a | ventral striatum | mrna levels | -tomasetti_2007 | 1.159 | 0.7, 1.617 | 0 | 23.63 | 0.1 | 0.261 |
| haloperidol | chronic | homer1a | ventral striatum | mrna levels | -tomasetti_2011 | 1.201 | 0.738, 1.664 | 0 | 24.27 | 0.11 | 0.258 |
| haloperidol | chronic | homer1b-c | cingulate cortex | mrna levels | -buonaguro_2017a | 0.159 | -0.315, 0.634 | 0.51 | 0 | 0 | 0.024 |
| haloperidol | chronic | homer1b-c | cingulate cortex | mrna levels | -de bartolomeis_2016_ | 0.397 | -0.676, 1.471 | 0.468 | 78.19 | 1.1 | 0.016 |
| haloperidol | chronic | homer1b-c | cingulate cortex | mrna levels | -de bartolomeis_2016_pretreat_chr_hal | 0.351 | -0.822, 1.525 | 0.557 | 81.7 | 1.38 | 0.011 |
| haloperidol | chronic | homer1b-c | cingulate cortex | mrna levels | -iasevoli_2010b | -0.107 | -0.545, 0.331 | 0.632 | 0 | 0 | 0.716 |
| haloperidol | chronic | homer1b-c | cingulate cortex | mrna levels | -iasevoli_2011 | 0.332 | -0.902, 1.567 | 0.597 | 79.93 | 1.52 | 0.011 |
| haloperidol | chronic | homer1b-c | cingulate cortex | mrna levels | -iasevoli_2014 | 0.284 | -0.906, 1.473 | 0.64 | 81.61 | 1.42 | 0.012 |
| haloperidol | chronic | homer1b-c | dorsal striatum | mrna levels | -buonaguro_2017a | 0.686 | 0.212, 1.16 | 0.005 | 0 | 0 | 0.52 |
| haloperidol | chronic | homer1b-c | dorsal striatum | mrna levels | -de bartolomeis_2016_ | 0.433 | -0.347, 1.213 | 0.277 | 61.21 | 0.47 | 0.037 |
| haloperidol | chronic | homer1b-c | dorsal striatum | mrna levels | -de bartolomeis_2016_pretreat_chr_hal | 0.49 | -0.276, 1.256 | 0.21 | 59.66 | 0.44 | 0.044 |
| haloperidol | chronic | homer1b-c | dorsal striatum | mrna levels | -iasevoli_2010b | 0.268 | -0.357, 0.892 | 0.401 | 46.59 | 0.23 | 0.114 |
| haloperidol | chronic | homer1b-c | dorsal striatum | mrna levels | -iasevoli_2011 | 0.295 | -0.461, 1.051 | 0.444 | 51.27 | 0.38 | 0.08 |
| haloperidol | chronic | homer1b-c | dorsal striatum | mrna levels | -iasevoli_2014 | 0.373 | -0.402, 1.148 | 0.345 | 59.66 | 0.45 | 0.041 |
| haloperidol | chronic | homer1b-c | limbic system | mrna levels | -buonaguro_2017a | 0.689 | -0.255, 1.632 | 0.153 | 70.65 | 0.79 | 0.008 |
| haloperidol | chronic | homer1b-c | limbic system | mrna levels | -de bartolomeis_2016_ | 0.662 | -0.298, 1.622 | 0.176 | 72.95 | 0.85 | 0.005 |
| haloperidol | chronic | homer1b-c | limbic system | mrna levels | -de bartolomeis_2016_pretreat_chr_hal | 0.562 | -0.449, 1.572 | 0.276 | 75.71 | 0.98 | 0.003 |
| haloperidol | chronic | homer1b-c | limbic system | mrna levels | -iasevoli_2010b | 0.29 | -0.468, 1.047 | 0.454 | 63.03 | 0.47 | 0.02 |
| haloperidol | chronic | homer1b-c | limbic system | mrna levels | -iasevoli_2011 | 0.159 | -0.471, 0.79 | 0.62 | 30.04 | 0.15 | 0.102 |
| haloperidol | chronic | homer1b-c | limbic system | mrna levels | -iasevoli_2014 | 0.702 | -0.219, 1.623 | 0.135 | 69.72 | 0.75 | 0.01 |
| haloperidol | chronic | homer1b-c | sensory-motor areas | mrna levels | -buonaguro_2017a | 0.245 | -0.224, 0.714 | 0.306 | 0 | 0 | 0.126 |
| haloperidol | chronic | homer1b-c | sensory-motor areas | mrna levels | -de bartolomeis_2016_ | 0.197 | -0.263, 0.656 | 0.401 | 0 | 0 | 0.091 |
| haloperidol | chronic | homer1b-c | sensory-motor areas | mrna levels | -de bartolomeis_2016_pretreat_chr_hal | 0.182 | -0.463, 0.827 | 0.58 | 43.73 | 0.23 | 0.065 |
| haloperidol | chronic | homer1b-c | sensory-motor areas | mrna levels | -iasevoli_2010b | -0.017 | -0.455, 0.421 | 0.939 | 0 | 0 | 0.759 |
| haloperidol | chronic | homer1b-c | sensory-motor areas | mrna levels | -iasevoli_2011 | 0.2 | -0.559, 0.959 | 0.605 | 50.59 | 0.37 | 0.066 |
| haloperidol | chronic | homer1b-c | sensory-motor areas | mrna levels | -iasevoli_2014 | 0.122 | -0.49, 0.734 | 0.695 | 36.14 | 0.17 | 0.07 |
| haloperidol | chronic | homer1b-c | ventral striatum | mrna levels | -buonaguro_2017a | 0.895 | 0.25, 1.54 | 0.007 | 39.27 | 0.21 | 0.165 |
| haloperidol | chronic | homer1b-c | ventral striatum | mrna levels | -de bartolomeis_2016_ | 0.797 | 0.054, 1.541 | 0.036 | 56.06 | 0.4 | 0.052 |
| haloperidol | chronic | homer1b-c | ventral striatum | mrna levels | -de bartolomeis_2016_pretreat_chr_hal | 0.721 | -0.061, 1.502 | 0.071 | 60.36 | 0.47 | 0.033 |
| haloperidol | chronic | homer1b-c | ventral striatum | mrna levels | -iasevoli_2010b | 0.561 | -0.128, 1.25 | 0.11 | 54.7 | 0.34 | 0.059 |
| haloperidol | chronic | homer1b-c | ventral striatum | mrna levels | -iasevoli_2011 | 0.369 | -0.147, 0.884 | 0.161 | 0 | 0 | 0.359 |
| haloperidol | chronic | homer1b-c | ventral striatum | mrna levels | -iasevoli_2014 | 0.708 | -0.085, 1.5 | 0.08 | 60.32 | 0.48 | 0.032 |
| haloperidol | chronic | mglur2 | frontal cortex | mrna levels | -korlatowicz_2018_ | -0.114 | -0.995, 0.767 | 0.8 | 0 | 0 | 0.559 |
| haloperidol | chronic | mglur2 | frontal cortex | mrna levels | -korlatowicz_2018_ketamine | -0.243 | -1.132, 0.645 | 0.592 | 0 | 0 | 0.378 |
| haloperidol | chronic | mglur2 | frontal cortex | mrna levels | -tascedda_2001 | -0.513 | -1.405, 0.379 | 0.259 | 0 | 0 | 0.763 |
| haloperidol | chronic | mglur5 | frontal cortex | mrna levels | -korlatowicz_2018_ | -0.123 | -1.009, 0.764 | 0.786 | 0 | 0 | 0.361 |
| haloperidol | chronic | mglur5 | frontal cortex | mrna levels | -korlatowicz_2018_ketamine | 0.311 | -0.571, 1.192 | 0.49 | 0 | 0 | 0.955 |
| haloperidol | chronic | mglur5 | frontal cortex | mrna levels | -tascedda_2001 | -0.098 | -0.986, 0.789 | 0.829 | 0 | 0 | 0.333 |
| haloperidol | chronic | nr1 | dorsal striatum | mrna levels | -brené_1998 | 1.24 | 0.235, 2.245 | 0.016 | 60.14 | 0.63 | 0.055 |
| haloperidol | chronic | nr1 | dorsal striatum | mrna levels | -eastwood_1994 | 1.863 | 0.545, 3.181 | 0.006 | 69.93 | 1.24 | 0.022 |
| haloperidol | chronic | nr1 | dorsal striatum | mrna levels | -iasevoli_2010b | 1.842 | 0.532, 3.152 | 0.006 | 72.63 | 1.27 | 0.014 |
| haloperidol | chronic | nr1 | dorsal striatum | mrna levels | -meshul_2000 | 1.784 | 0.388, 3.179 | 0.012 | 74.8 | 1.49 | 0.01 |
| haloperidol | chronic | nr1 | dorsal striatum | mrna levels | -riva_1997 | 1.243 | 0.121, 2.364 | 0.03 | 59.46 | 0.76 | 0.072 |
| haloperidol | chronic | nr1 | dorsal striatum | protein levels | -fitzgerald_1995 | 0.108 | -0.732, 0.948 | 0.801 | 0 | 0 | 0.529 |
| haloperidol | chronic | nr1 | dorsal striatum | protein levels | -hanaoka_2003 | 0.751 | -0.919, 2.42 | 0.378 | 81 | 1.18 | 0.022 |
| haloperidol | chronic | nr1 | dorsal striatum | protein levels | -pan_2016 | 1.07 | -0.058, 2.197 | 0.063 | 53.64 | 0.36 | 0.142 |
| haloperidol | chronic | nr1 | frontal cortex | mrna levels | -brené_1998 | 0.164 | -0.373, 0.702 | 0.549 | 0 | 0 | 0.489 |
| haloperidol | chronic | nr1 | frontal cortex | mrna levels | -eastwood_1994 | 0.144 | -0.404, 0.691 | 0.607 | 0 | 0 | 0.519 |
| haloperidol | chronic | nr1 | frontal cortex | mrna levels | -riva_1997 | 0.111 | -0.485, 0.706 | 0.716 | 0 | 0 | 0.533 |
| haloperidol | chronic | nr1 | frontal cortex | mrna levels | -schmitt_2003 | 0.436 | -0.176, 1.048 | 0.163 | 0 | 0 | 0.997 |
| haloperidol | chronic | nr1 | frontal cortex | protein levels | -fitzgerald_1995 | -0.333 | -1.557, 0.892 | 0.594 | 66.44 | 0.78 | 0.054 |
| haloperidol | chronic | nr1 | frontal cortex | protein levels | -fumagalli_2008 | 0.263 | -0.318, 0.843 | 0.375 | 0 | 0 | 0.984 |
| haloperidol | chronic | nr1 | frontal cortex | protein levels | -hanaoka_2003 | -0.276 | -1.456, 0.904 | 0.647 | 72.55 | 0.78 | 0.037 |
| haloperidol | chronic | nr1 | frontal cortex | protein levels | -pan_2016 | -0.328 | -1.48, 0.825 | 0.578 | 69.34 | 0.71 | 0.045 |
| haloperidol | chronic | nr1 | hippocampus | protein levels | -fitzgerald_1995 | -0.21 | -0.946, 0.525 | 0.575 | 12.86 | 0.05 | 0.307 |
| haloperidol | chronic | nr1 | hippocampus | protein levels | -fumagalli_2008 | -0.307 | -0.896, 0.282 | 0.307 | 0 | 0 | 0.314 |
| haloperidol | chronic | nr1 | hippocampus | protein levels | -hanaoka_2003 | -0.409 | -0.984, 0.165 | 0.163 | 0 | 0 | 0.608 |
| haloperidol | chronic | nr1 | hippocampus | protein levels | -krzystanek_2015 | -0.109 | -0.691, 0.473 | 0.713 | 0 | 0 | 0.604 |
| haloperidol | chronic | nr1 | ventral striatum | mrna levels | -brené_1998 | 0.193 | -0.476, 0.863 | 0.571 | 9.83 | 0.04 | 0.394 |
| haloperidol | chronic | nr1 | ventral striatum | mrna levels | -eastwood_1994 | 0.903 | -0.796, 2.603 | 0.297 | 80.01 | 1.78 | 0.01 |
| haloperidol | chronic | nr1 | ventral striatum | mrna levels | -iasevoli_2010b | 0.914 | -0.736, 2.564 | 0.278 | 81.96 | 1.71 | 0.009 |
| haloperidol | chronic | nr1 | ventral striatum | mrna levels | -riva_1997 | 1.209 | -0.054, 2.473 | 0.061 | 57.98 | 0.72 | 0.1 |
| haloperidol | chronic | nr1 | ventral striatum | protein levels | -fitzgerald_1995 | 0.751 | -0.675, 2.176 | 0.302 | 60.98 | 0.64 | 0.109 |
| haloperidol | chronic | nr1 | ventral striatum | protein levels | -hanaoka_2003 | 0.563 | -1.091, 2.217 | 0.505 | 79.36 | 1.14 | 0.028 |
| haloperidol | chronic | nr1 | ventral striatum | protein levels | -pan_2016 | -0.137 | -0.811, 0.536 | 0.689 | 0 | 0 | 0.75 |
| haloperidol | chronic | nr2a | frontal cortex | protein levels | -fumagalli_2008 | -1.019 | -2.606, 0.567 | 0.208 | 66.5 | 0.87 | 0.084 |
| haloperidol | chronic | nr2a | frontal cortex | protein levels | -hanaoka_2003 | -1.305 | -2.259, -0.35 | 0.007 | 12.53 | 0.06 | 0.285 |
| haloperidol | chronic | nr2a | frontal cortex | protein levels | -pan_2016 | -0.567 | -1.425, 0.29 | 0.195 | 0 | 0 | 0.466 |
| haloperidol | chronic | nr2a | hippocampus | protein levels | -fumagalli_2008 | -0.764 | -2.184, 0.657 | 0.292 | 59.65 | 0.63 | 0.115 |
| haloperidol | chronic | nr2a | hippocampus | protein levels | -hanaoka_2003 | -0.201 | -1.003, 0.602 | 0.624 | 0 | 0 | 0.805 |
| haloperidol | chronic | nr2a | hippocampus | protein levels | -krzystanek_2015 | -0.856 | -2.075, 0.364 | 0.169 | 45.36 | 0.36 | 0.176 |
| haloperidol | chronic | nr2b | hippocampus | protein levels | -fumagalli_2008 | -1.585 | -2.547, -0.624 | 0.001 | 0 | 0 | 0.572 |
| haloperidol | chronic | nr2b | hippocampus | protein levels | -hanaoka_2003 | -0.674 | -1.947, 0.6 | 0.3 | 56.07 | 0.47 | 0.131 |
| haloperidol | chronic | nr2b | hippocampus | protein levels | -krzystanek_2015 | -0.917 | -2.739, 0.904 | 0.324 | 73.6 | 1.28 | 0.052 |
| haloperidol | chronic | psd95 | cingulate cortex | mrna levels | -buonaguro_2017a | 0.846 | -0.737, 2.43 | 0.295 | 85.34 | 2.18 | 0 |
| haloperidol | chronic | psd95 | cingulate cortex | mrna levels | -de bartolomeis_2016_ | 0.293 | -1.924, 2.51 | 0.796 | 92.3 | 4.66 | 0 |
| haloperidol | chronic | psd95 | cingulate cortex | mrna levels | -de bartolomeis_2016_pretreat_chr_hal | 0.583 | -1.472, 2.639 | 0.578 | 91.34 | 3.96 | 0 |
| haloperidol | chronic | psd95 | cingulate cortex | mrna levels | -iasevoli_2010b | -0.238 | -1.987, 1.511 | 0.79 | 90.05 | 2.86 | 0 |
| haloperidol | chronic | psd95 | cingulate cortex | mrna levels | -iasevoli_2011 | -0.217 | -2.086, 1.652 | 0.82 | 87.72 | 3.15 | 0 |
| haloperidol | chronic | psd95 | dorsal striatum | mrna levels | -buonaguro_2017a | 0.684 | -0.602, 1.971 | 0.297 | 79.34 | 1.33 | 0.002 |
| haloperidol | chronic | psd95 | dorsal striatum | mrna levels | -de bartolomeis_2016_ | 0.516 | -0.965, 1.997 | 0.495 | 85.13 | 1.9 | 0 |
| haloperidol | chronic | psd95 | dorsal striatum | mrna levels | -de bartolomeis_2016_pretreat_chr_hal | 0.594 | -0.815, 2.003 | 0.409 | 83.7 | 1.69 | 0 |
| haloperidol | chronic | psd95 | dorsal striatum | mrna levels | -iasevoli_2010b | 0.014 | -1.158, 1.185 | 0.982 | 80.27 | 1.14 | 0.001 |
| haloperidol | chronic | psd95 | dorsal striatum | mrna levels | -iasevoli_2011 | -0.045 | -1.213, 1.122 | 0.94 | 72.07 | 1.01 | 0.029 |
| haloperidol | chronic | psd95 | frontal cortex | protein levels | -fumagalli_2008 | -0.237 | -1.118, 0.644 | 0.598 | 0 | 0 | 0.723 |
| haloperidol | chronic | psd95 | frontal cortex | protein levels | -seo_2015_ | -0.184 | -1.022, 0.654 | 0.666 | 0 | 0 | 0.822 |
| haloperidol | chronic | psd95 | frontal cortex | protein levels | -seo_2015_stress | -0.329 | -1.171, 0.512 | 0.443 | 0 | 0 | 0.885 |
| haloperidol | chronic | psd95 | limbic system | mrna levels | -buonaguro_2017a | -0.082 | -1.251, 1.087 | 0.891 | 76.32 | 1.07 | 0.005 |
| haloperidol | chronic | psd95 | limbic system | mrna levels | -de bartolomeis_2016_ | -0.267 | -1.556, 1.021 | 0.684 | 81.28 | 1.38 | 0.001 |
| haloperidol | chronic | psd95 | limbic system | mrna levels | -de bartolomeis_2016_pretreat_chr_hal | 0.013 | -0.991, 1.017 | 0.98 | 71.13 | 0.73 | 0.011 |
| haloperidol | chronic | psd95 | limbic system | mrna levels | -iasevoli_2010b | -0.525 | -1.67, 0.619 | 0.368 | 78.67 | 1.06 | 0.001 |
| haloperidol | chronic | psd95 | limbic system | mrna levels | -iasevoli_2011 | -0.685 | -1.619, 0.248 | 0.15 | 56.46 | 0.51 | 0.08 |
| haloperidol | chronic | psd95 | sensory-motor areas | mrna levels | -buonaguro_2017a | 0.524 | -0.653, 1.702 | 0.383 | 76.13 | 1.08 | 0.005 |
| haloperidol | chronic | psd95 | sensory-motor areas | mrna levels | -de bartolomeis_2016_ | 0.118 | -1.498, 1.735 | 0.886 | 87.42 | 2.34 | 0 |
| haloperidol | chronic | psd95 | sensory-motor areas | mrna levels | -de bartolomeis_2016_pretreat_chr_hal | 0.343 | -1.14, 1.827 | 0.65 | 85.43 | 1.92 | 0 |
| haloperidol | chronic | psd95 | sensory-motor areas | mrna levels | -iasevoli_2010b | -0.243 | -1.561, 1.076 | 0.718 | 83.93 | 1.51 | 0 |
| haloperidol | chronic | psd95 | sensory-motor areas | mrna levels | -iasevoli_2011 | -0.29 | -1.615, 1.036 | 0.668 | 77.89 | 1.41 | 0.007 |
| haloperidol | chronic | psd95 | ventral striatum | mrna levels | -buonaguro_2017a | 0.45 | -0.48, 1.38 | 0.343 | 64.46 | 0.57 | 0.027 |
| haloperidol | chronic | psd95 | ventral striatum | mrna levels | -de bartolomeis_2016_ | 0.201 | -0.93, 1.331 | 0.728 | 76.9 | 1.01 | 0.002 |
| haloperidol | chronic | psd95 | ventral striatum | mrna levels | -de bartolomeis_2016_pretreat_chr_hal | 0.173 | -0.963, 1.31 | 0.765 | 77.14 | 1.03 | 0.002 |
| haloperidol | chronic | psd95 | ventral striatum | mrna levels | -iasevoli_2010b | 0.229 | -0.862, 1.32 | 0.68 | 77.48 | 0.96 | 0.002 |
| haloperidol | chronic | psd95 | ventral striatum | mrna levels | -iasevoli_2011 | -0.3 | -0.879, 0.278 | 0.309 | 0 | 0 | 0.605 |
| olanzapine | acute | arc | dorsal striatum | mrna levels | -de bartolomeis_2015 | 1.828 | 0.945, 2.712 | 0 | 65.03 | 0.27 | 0.091 |
| olanzapine | acute | arc | dorsal striatum | mrna levels | -fumagalli_2009 | 1.378 | -0.503, 3.26 | 0.151 | 87.6 | 1.62 | 0.005 |
| olanzapine | acute | arc | dorsal striatum | mrna levels | -sakuma_2015 | 0.979 | -0.005, 1.963 | 0.051 | 60.46 | 0.31 | 0.112 |
| olanzapine | acute | homer1a | dorsal striatum | mrna levels | -de bartolomeis_2002 | 4.727 | -1.975, 11.429 | 0.167 | 97.44 | 32.37 | 0 |
| olanzapine | acute | homer1a | dorsal striatum | mrna levels | -de bartolomeis_2015 | 4.101 | -3.217, 11.419 | 0.272 | 98.05 | 39.09 | 0 |
| olanzapine | acute | homer1a | dorsal striatum | mrna levels | -iasevoli_2010a | 1.161 | -0.02, 2.342 | 0.054 | 59.47 | 0.65 | 0.087 |
| olanzapine | acute | homer1a | dorsal striatum | mrna levels | -iasevoli_2020 | 4.635 | -2.214, 11.483 | 0.185 | 97.6 | 33.91 | 0 |
| olanzapine | acute | homer1a | ventral striatum | mrna levels | -de bartolomeis_2002 | 2.205 | 0.007, 4.403 | 0.049 | 83.03 | 3.07 | 0.004 |
| olanzapine | acute | homer1a | ventral striatum | mrna levels | -de bartolomeis_2015 | 1.707 | -0.529, 3.943 | 0.135 | 84.19 | 3.22 | 0.01 |
| olanzapine | acute | homer1a | ventral striatum | mrna levels | -iasevoli_2010a | 1.228 | 0.017, 2.44 | 0.047 | 60.74 | 0.7 | 0.079 |
| olanzapine | acute | homer1a | ventral striatum | mrna levels | -iasevoli_2020 | 2.4 | 0.662, 4.137 | 0.007 | 71.78 | 1.66 | 0.039 |
| olanzapine | acute | psd95 | frontal cortex | protein levels | -dutra-tavares_2023_ _female | 3.472 | -0.405, 7.349 | 0.079 | 96.66 | 18.05 | 0 |
| olanzapine | acute | psd95 | frontal cortex | protein levels | -dutra-tavares_2023_ _male | 3.294 | -0.727, 7.315 | 0.108 | 97 | 19.53 | 0 |
| olanzapine | acute | psd95 | frontal cortex | protein levels | -dutra-tavares_2023_pcp_20_female | 1.733 | -1.158, 4.623 | 0.24 | 95.72 | 9.98 | 0 |
| olanzapine | acute | psd95 | frontal cortex | protein levels | -dutra-tavares_2023_pcp_20_male | 3.517 | -0.309, 7.343 | 0.072 | 96.57 | 17.54 | 0 |
| olanzapine | acute | psd95 | frontal cortex | protein levels | -dutra-tavares_2023_pcp_5_female | 1.63 | -1.052, 4.312 | 0.234 | 95.06 | 8.55 | 0 |
| olanzapine | acute | psd95 | frontal cortex | protein levels | -dutra-tavares_2023_pcp_5_male | 3.366 | -0.607, 7.338 | 0.097 | 96.86 | 19.03 | 0 |
| olanzapine | acute | psd95 | hippocampus | protein levels | -dutra-tavares_2023_ _female | 0.053 | -0.457, 0.564 | 0.838 | 0 | 0 | 0.734 |
| olanzapine | acute | psd95 | hippocampus | protein levels | -dutra-tavares_2023_ _male | -0.209 | -0.79, 0.372 | 0.48 | 20.29 | 0.09 | 0.27 |
| olanzapine | acute | psd95 | hippocampus | protein levels | -dutra-tavares_2023_pcp_20_female | -0.261 | -0.779, 0.256 | 0.322 | 0 | 0 | 0.381 |
| olanzapine | acute | psd95 | hippocampus | protein levels | -dutra-tavares_2023_pcp_20_male | -0.196 | -0.786, 0.395 | 0.516 | 22.69 | 0.1 | 0.256 |
| olanzapine | acute | psd95 | hippocampus | protein levels | -dutra-tavares_2023_pcp_5_female | -0.186 | -0.782, 0.409 | 0.54 | 23.95 | 0.11 | 0.249 |
| olanzapine | acute | psd95 | hippocampus | protein levels | -dutra-tavares_2023_pcp_5_male | -0.036 | -0.573, 0.5 | 0.894 | 7.31 | 0.03 | 0.321 |
| olanzapine | chronic | nr1 | frontal cortex | protein levels | -fumagalli_2008 | 0.996 | 0.06, 1.933 | 0.037 | 0 | 0 | 0.43 |
| olanzapine | chronic | nr1 | frontal cortex | protein levels | -kao_2018 | 0.005 | -0.894, 0.903 | 0.992 | 2.08 | 0.01 | 0.312 |
| olanzapine | chronic | nr1 | frontal cortex | protein levels | -mahmoud_2021 | 0.461 | -1.201, 2.124 | 0.587 | 74.09 | 1.07 | 0.049 |
| olanzapine | chronic | nr1 | hippocampus | protein levels | -fumagalli_2008 | -0.383 | -1.634, 0.867 | 0.548 | 56.2 | 0.46 | 0.131 |
| olanzapine | chronic | nr1 | hippocampus | protein levels | -kao_2018 | -0.569 | -1.439, 0.3 | 0.199 | 9.73 | 0.04 | 0.293 |
| olanzapine | chronic | nr1 | hippocampus | protein levels | -krzystanek_2015 | 0.044 | -0.758, 0.846 | 0.915 | 0 | 0 | 0.635 |
| olanzapine | chronic | nr2a | hippocampus | protein levels | -fumagalli_2008 | -0.158 | -1.059, 0.744 | 0.732 | 18.89 | 0.08 | 0.267 |
| olanzapine | chronic | nr2a | hippocampus | protein levels | -kao_2018 | -0.284 | -1.094, 0.525 | 0.491 | 0 | 0 | 0.421 |
| olanzapine | chronic | nr2a | hippocampus | protein levels | -krzystanek_2015 | 0.167 | -0.635, 0.97 | 0.683 | 0 | 0 | 0.755 |
| olanzapine | chronic | nr2b | hippocampus | protein levels | -fumagalli_2008 | -0.304 | -2.033, 1.425 | 0.73 | 76.23 | 1.19 | 0.04 |
| olanzapine | chronic | nr2b | hippocampus | protein levels | -kao_2018 | -0.697 | -1.62, 0.225 | 0.139 | 18.06 | 0.08 | 0.269 |
| olanzapine | chronic | nr2b | hippocampus | protein levels | -krzystanek_2015 | 0.148 | -0.661, 0.958 | 0.72 | 0 | 0 | 0.32 |
| olanzapine | chronic | psd95 | frontal cortex | protein levels | -fumagalli_2008 | 0.402 | -0.852, 1.657 | 0.53 | 47.63 | 0.39 | 0.167 |
| olanzapine | chronic | psd95 | frontal cortex | protein levels | -seo_2015_ | -0.016 | -2.097, 2.065 | 0.988 | 81.45 | 1.84 | 0.02 |
| olanzapine | chronic | psd95 | frontal cortex | protein levels | -seo_2015_stress | -0.65 | -1.517, 0.216 | 0.141 | 0 | 0 | 0.341 |

Sensitivity analyses based on quality assessment

| **drug** | **model** | **psd type** | **region** | **outcome type** | **n_studies** | **n_group_1** | **n_group_2** | **beta** | **CI** | **p_val** | **PI** | **i_sq** | **tau_sq** | **q_p_val** |
| --- | --- | --- | --- | --- | --- | --- | --- | --- | --- | --- | --- | --- | --- | --- |
| amisulpride | chronic | arc | cingulate cortex | mrna levels | 2 | 12 | 12 | 0.22 | -0.583, 1.024 | 0.59115941 | -0.583, 1.024 | 0 | 0 | 0.7398 |
| amisulpride | chronic | arc | dorsal striatum | mrna levels | 2 | 12 | 12 | 1.235 | 0.359, 2.111 | 0.00573881 | 0.359, 2.111 | 0 | 0 | 0.5518 |
| amisulpride | chronic | arc | limbic system | mrna levels | 2 | 12 | 12 | 0.454 | -0.485, 1.393 | 0.34349765 | -0.684, 1.592 | 23.38 | 0.11 | 0.2533 |
| amisulpride | chronic | arc | sensory-motor areas | mrna levels | 2 | 12 | 12 | 0.12 | -0.685, 0.925 | 0.77032363 | -0.685, 0.925 | 0 | 0 | 0.476 |
| amisulpride | chronic | arc | ventral striatum | mrna levels | 2 | 12 | 12 | 0.825 | -0.01, 1.66 | 0.05270135 | -0.01, 1.66 | 0 | 0 | 0.6841 |
| amisulpride | chronic | homer1a | cingulate cortex | mrna levels | 2 | 12 | 12 | 1.355 | -1.757, 4.466 | 0.39347591 | -3.848, 6.557 | 89.66 | 4.53 | 0.0019 |
| amisulpride | chronic | homer1a | dorsal striatum | mrna levels | 2 | 12 | 12 | 1.804 | -0.412, 4.02 | 0.11065992 | -1.764, 5.372 | 79.2 | 2.04 | 0.0283 |
| amisulpride | chronic | homer1a | limbic system | mrna levels | 2 | 12 | 12 | 2.678 | -3.045, 8.401 | 0.3590189 | -7.041, 12.397 | 94.06 | 16.06 | 0 |
| amisulpride | chronic | homer1a | sensory-motor areas | mrna levels | 2 | 12 | 12 | 0.575 | -2.134, 3.285 | 0.67732768 | -3.94, 5.091 | 88.81 | 3.4 | 0.0028 |
| amisulpride | chronic | homer1a | ventral striatum | mrna levels | 2 | 12 | 12 | 0.982 | -0.103, 2.067 | 0.07613854 | -0.449, 2.413 | 36.76 | 0.23 | 0.2086 |
| amisulpride | chronic | homer1b-c | cingulate cortex | mrna levels | 2 | 12 | 12 | 0.189 | -1.742, 2.12 | 0.84805566 | -2.933, 3.31 | 80.66 | 1.57 | 0.023 |
| amisulpride | chronic | homer1b-c | dorsal striatum | mrna levels | 2 | 12 | 12 | 0.972 | -2.829, 4.772 | 0.61623365 | -5.454, 7.398 | 92.92 | 6.99 | 0.0002 |
| amisulpride | chronic | homer1b-c | limbic system | mrna levels | 2 | 12 | 12 | 0.388 | -2.039, 2.814 | 0.7542967 | -3.627, 4.402 | 86.82 | 2.66 | 0.0059 |
| amisulpride | chronic | homer1b-c | sensory-motor areas | mrna levels | 2 | 12 | 12 | 0.282 | -1.513, 2.078 | 0.75789314 | -2.589, 3.154 | 77.85 | 1.31 | 0.0336 |
| amisulpride | chronic | homer1b-c | ventral striatum | mrna levels | 2 | 12 | 12 | 1.215 | -0.913, 3.344 | 0.26305651 | -2.232, 4.663 | 80.98 | 1.91 | 0.0218 |
| amisulpride | chronic | psd95 | cingulate cortex | mrna levels | 2 | 12 | 12 | -0.006 | -0.806, 0.794 | 0.9882636 | -0.806, 0.794 | 0 | 0 | 0.883 |
| amisulpride | chronic | psd95 | dorsal striatum | mrna levels | 2 | 12 | 12 | -0.438 | -1.825, 0.949 | 0.53623677 | -2.53, 1.655 | 63.79 | 0.64 | 0.0966 |
| amisulpride | chronic | psd95 | limbic system | mrna levels | 2 | 12 | 12 | -0.507 | -1.337, 0.324 | 0.23176349 | -1.355, 0.341 | 2.11 | 0.01 | 0.3121 |
| amisulpride | chronic | psd95 | sensory-motor areas | mrna levels | 2 | 12 | 12 | -0.063 | -0.864, 0.737 | 0.87678829 | -0.864, 0.737 | 0 | 0 | 0.8767 |
| amisulpride | chronic | psd95 | ventral striatum | mrna levels | 2 | 12 | 12 | -0.08 | -0.882, 0.722 | 0.844924 | -0.882, 0.722 | 0 | 0 | 0.6302 |
| aripiprazole | acute | arc | dorsal striatum | mrna levels | 2 | 35 | 25 | 1.006 | -2.546, 4.558 | 0.5788137 | -5.071, 7.083 | 96.33 | 6.33 | 0 |
| aripiprazole | acute | arc | frontal cortex | mrna levels | 2 | 35 | 25 | -2.268 | -4.586, 0.051 | 0.05521776 | -6.151, 1.616 | 90.29 | 2.53 | 0.0013 |
| aripiprazole | chronic | homer1a | ventral striatum | protein levels | 2 | 12 | 12 | 2.574 | 0.261, 4.886 | 0.02914093 | -1.097, 6.245 | 75.35 | 2.12 | 0.044 |
| aripiprazole | chronic | homer1b-c | ventral striatum | protein levels | 2 | 12 | 12 | -0.623 | -3.312, 2.065 | 0.6495305 | -5.101, 3.854 | 88.6 | 3.34 | 0.0031 |
| aripiprazole | chronic | mglur1 | ventral striatum | protein levels | 2 | 12 | 12 | -0.371 | -1.178, 0.436 | 0.36756978 | -1.178, 0.436 | 0 | 0 | 0.9855 |
| aripiprazole | chronic | mglur5 | ventral striatum | protein levels | 2 | 12 | 12 | 0.12 | -1.864, 2.104 | 0.90559419 | -3.1, 3.34 | 81.63 | 1.67 | 0.0197 |
| aripiprazole | chronic | norbin | ventral striatum | protein levels | 2 | 12 | 12 | 1.725 | 0.782, 2.667 | 0.00033353 | 0.782, 2.667 | 0 | 0 | 0.4768 |
| aripiprazole | chronic | nr1 | frontal cortex | protein levels | 2 | 12 | 12 | -0.002 | -0.802, 0.798 | 0.99583721 | -0.802, 0.798 | 0 | 0 | 0.9958 |
| aripiprazole | chronic | nr1 | ventral striatum | protein levels | 2 | 12 | 12 | 0.321 | -1.858, 2.501 | 0.77254555 | -3.25, 3.892 | 84.25 | 2.08 | 0.0117 |
| aripiprazole | chronic | psd95 | frontal cortex | protein levels | 2 | 10 | 10 | 1.46 | 0.47, 2.45 | 0.00385209 | 0.47, 2.45 | 0 | 0 | 0.5864 |
| asenapine | acute | homer1a | cingulate cortex | mrna levels | 4 | 46 | 22 | 0.122 | -0.794, 1.038 | 0.79451752 | -1.593, 1.836 | 63.3 | 0.55 | 0.0438 |
| asenapine | acute | homer1a | dorsal striatum | mrna levels | 4 | 46 | 22 | 1.019 | 0.464, 1.574 | 0.00031943 | 0.464, 1.574 | 0 | 0 | 0.4963 |
| asenapine | acute | homer1a | hippocampus | mrna levels | 2 | 10 | 10 | -0.751 | -3.629, 2.127 | 0.60926092 | -5.524, 4.023 | 87.51 | 3.78 | 0.0047 |
| asenapine | acute | homer1a | limbic system | mrna levels | 4 | 46 | 22 | 0.274 | -0.344, 0.893 | 0.38436891 | -0.584, 1.133 | 22.98 | 0.09 | 0.2417 |
| asenapine | acute | homer1a | sensory-motor areas | mrna levels | 4 | 46 | 22 | 0.21 | -0.557, 0.978 | 0.59128363 | -1.104, 1.525 | 48.69 | 0.3 | 0.1174 |
| asenapine | acute | homer1a | ventral striatum | mrna levels | 4 | 46 | 22 | 0.57 | -0.239, 1.379 | 0.16711397 | -0.857, 1.997 | 53.13 | 0.36 | 0.0923 |
| asenapine | chronic | homer1a | cingulate cortex | mrna levels | 2 | 30 | 10 | -0.064 | -0.78, 0.652 | 0.86107453 | -0.78, 0.652 | 0 | 0 | 0.861 |
| asenapine | chronic | homer1a | dorsal striatum | mrna levels | 2 | 30 | 10 | 0.137 | -0.782, 1.057 | 0.76951282 | -1.083, 1.358 | 38.08 | 0.17 | 0.2038 |
| asenapine | chronic | homer1a | limbic system | mrna levels | 2 | 30 | 10 | -1.231 | -2.076, -0.385 | 0.0043296 | -2.208, -0.253 | 16.8 | 0.06 | 0.2729 |
| asenapine | chronic | homer1a | sensory-motor areas | mrna levels | 2 | 30 | 10 | -0.06 | -0.776, 0.657 | 0.86965689 | -0.776, 0.657 | 0 | 0 | 0.6934 |
| asenapine | chronic | homer1a | ventral striatum | mrna levels | 2 | 30 | 10 | 0.431 | -0.458, 1.32 | 0.34183304 | -0.713, 1.576 | 32.81 | 0.14 | 0.2225 |
| clozapine | acute | arc | dorsal striatum | mrna levels | 2 | 27 | 27 | -0.018 | -0.624, 0.588 | 0.95351885 | -0.724, 0.688 | 14.77 | 0.03 | 0.2787 |
| clozapine | acute | arc | frontal cortex | mrna levels | 2 | 27 | 27 | -1.366 | -2.525, -0.207 | 0.02084698 | -3.156, 0.423 | 68.35 | 0.48 | 0.0755 |
| clozapine | acute | homer1a | cingulate cortex | mrna levels | 2 | 12 | 12 | 2.119 | 1.119, 3.119 | 3.2667E-05 | 1.119, 3.119 | 0 | 0 | 0.9358 |
| clozapine | acute | homer1a | dorsal striatum | mrna levels | 3 | 17 | 17 | 1.131 | 0.406, 1.856 | 0.00223067 | 0.406, 1.856 | 0 | 0 | 0.9033 |
| clozapine | acute | homer1a | ventral striatum | mrna levels | 3 | 17 | 17 | 1.478 | 0.716, 2.239 | 0.00014279 | 0.716, 2.239 | 0 | 0 | 0.7652 |
| clozapine | acute | mglur1 | frontal cortex | mrna levels | 2 | 10 | 10 | 0.931 | -0.036, 1.898 | 0.05904279 | -0.095, 1.957 | 6.29 | 0.03 | 0.3016 |
| clozapine | acute | mglur2 | frontal cortex | mrna levels | 2 | 10 | 10 | 0.042 | -0.839, 0.923 | 0.92540925 | -0.839, 0.923 | 0 | 0 | 0.5292 |
| clozapine | acute | mglur5 | frontal cortex | mrna levels | 2 | 10 | 10 | -0.651 | -1.551, 0.249 | 0.15624973 | -1.551, 0.249 | 0 | 0 | 0.7929 |
| clozapine | acute | nr1 | frontal cortex | mrna levels | 2 | 8 | 8 | 1.067 | 0.011, 2.123 | 0.04763652 | 0.011, 2.123 | 0 | 0 | 0.4699 |
| clozapine | acute | nr1 | hippocampus | mrna levels | 2 | 8 | 8 | 0.08 | -0.902, 1.061 | 0.87354262 | -0.902, 1.061 | 0 | 0 | 0.8086 |
| clozapine | acute | nr2a | frontal cortex | mrna levels | 2 | 8 | 8 | 0.791 | -0.242, 1.824 | 0.13341099 | -0.242, 1.824 | 0 | 0 | 0.3335 |
| clozapine | acute | nr2a | hippocampus | mrna levels | 2 | 8 | 8 | -0.19 | -1.345, 0.964 | 0.74648728 | -1.6, 1.219 | 24.49 | 0.17 | 0.2498 |
| clozapine | acute | nr2b | frontal cortex | mrna levels | 2 | 8 | 8 | 1.007 | -0.034, 2.047 | 0.05784692 | -0.034, 2.047 | 0 | 0 | 0.9051 |
| clozapine | acute | nr2b | hippocampus | mrna levels | 2 | 8 | 8 | 0.042 | -0.942, 1.027 | 0.93296522 | -0.942, 1.027 | 0 | 0 | 0.594 |
| clozapine | chronic | mglur1 | frontal cortex | mrna levels | 2 | 20 | 10 | 0.189 | -0.572, 0.95 | 0.62714207 | -0.572, 0.95 | 0 | 0 | 0.7714 |
| clozapine | chronic | mglur2 | frontal cortex | mrna levels | 2 | 20 | 10 | -0.181 | -0.943, 0.58 | 0.64036278 | -0.943, 0.58 | 0 | 0 | 0.7368 |
| clozapine | chronic | mglur5 | frontal cortex | mrna levels | 2 | 20 | 10 | 0.072 | -0.687, 0.831 | 0.85231239 | -0.687, 0.831 | 0 | 0 | 0.9513 |
| clozapine | chronic | nr1 | dorsal striatum | mrna levels | 3 | 19 | 19 | 1.289 | -1.235, 3.813 | 0.31685543 | -3.536, 6.115 | 90.67 | 4.4 | 0.0009 |
| clozapine | chronic | nr1 | frontal cortex | mrna levels | 2 | 14 | 14 | 0.817 | 0.046, 1.589 | 0.03785003 | 0.046, 1.589 | 0 | 0 | 0.8153 |
| clozapine | chronic | nr1 | hippocampus | mrna levels | 2 | 14 | 14 | 0.892 | -0.216, 2 | 0.11441957 | -0.667, 2.452 | 48.89 | 0.31 | 0.1619 |
| haloperidol | acute | ania3 | associative cortex | mrna levels | 3 | 17 | 17 | 0.018 | -0.977, 1.013 | 0.97178106 | -1.565, 1.601 | 51.03 | 0.39 | 0.1294 |
| haloperidol | acute | ania3 | cingulate cortex | mrna levels | 4 | 23 | 23 | 0.997 | -0.261, 2.255 | 0.12030827 | -1.478, 3.472 | 72.81 | 1.18 | 0.0125 |
| haloperidol | acute | ania3 | dorsal striatum | mrna levels | 5 | 28 | 29 | 2.471 | 1.076, 3.866 | 0.00051852 | -0.521, 5.463 | 73.64 | 1.82 | 0.0053 |
| haloperidol | acute | ania3 | frontal cortex | mrna levels | 3 | 17 | 17 | -0.136 | -0.817, 0.545 | 0.69530376 | -0.817, 0.545 | 0 | 0 | 0.4424 |
| haloperidol | acute | ania3 | limbic system | mrna levels | 2 | 11 | 11 | 0.615 | -0.241, 1.471 | 0.15884517 | -0.241, 1.471 | 0 | 0 | 0.8625 |
| haloperidol | acute | ania3 | sensory-motor areas | mrna levels | 2 | 11 | 11 | 0.384 | -0.901, 1.67 | 0.5578257 | -1.454, 2.223 | 50.8 | 0.45 | 0.154 |
| haloperidol | acute | ania3 | ventral striatum | mrna levels | 5 | 28 | 29 | 1.425 | 0.607, 2.243 | 0.00064121 | -0.056, 2.906 | 45.76 | 0.4 | 0.1156 |
| haloperidol | acute | arc | cingulate cortex | mrna levels | 5 | 46 | 32 | 0.022 | -0.932, 0.976 | 0.96388064 | -2.026, 2.07 | 72.9 | 0.85 | 0.0035 |
| haloperidol | acute | arc | dorsal striatum | mrna levels | 9 | 109 | 85 | 2.368 | 1.094, 3.642 | 0.00027078 | -1.416, 6.152 | 90.17 | 3.3 | 0 |
| haloperidol | acute | arc | frontal cortex | mrna levels | 4 | 63 | 53 | -1.043 | -1.44, -0.645 | 2.7552E-07 | -1.44, -0.645 | 0 | 0 | 0.6097 |
| haloperidol | acute | arc | limbic system | mrna levels | 5 | 46 | 32 | -0.212 | -1.183, 0.759 | 0.66867532 | -2.303, 1.879 | 73.57 | 0.89 | 0.0051 |
| haloperidol | acute | arc | sensory-motor areas | mrna levels | 5 | 46 | 32 | -0.36 | -1.163, 0.442 | 0.3789775 | -1.984, 1.263 | 62.44 | 0.52 | 0.0278 |
| haloperidol | acute | arc | ventral striatum | mrna levels | 6 | 66 | 52 | 1.81 | 1.142, 2.477 | 1.0876E-07 | 0.486, 3.133 | 50.6 | 0.34 | 0.0609 |
| haloperidol | acute | homer1a | associative cortex | mrna levels | 3 | 17 | 17 | -0.361 | -1.47, 0.749 | 0.52395279 | -2.203, 1.482 | 58.76 | 0.56 | 0.0945 |
| haloperidol | acute | homer1a | cingulate cortex | mrna levels | 11 | 98 | 74 | 0.151 | -0.266, 0.568 | 0.47875811 | -0.8, 1.101 | 39.03 | 0.19 | 0.0559 |
| haloperidol | acute | homer1a | dorsal striatum | mrna levels | 15 | 118 | 94 | 2.114 | 1.428, 2.8 | 1.5719E-09 | -0.097, 4.325 | 70.36 | 1.15 | 0 |
| haloperidol | acute | homer1a | frontal cortex | mrna levels | 3 | 17 | 17 | 0.033 | -0.647, 0.713 | 0.92422709 | -0.647, 0.713 | 0 | 0 | 0.4486 |
| haloperidol | acute | homer1a | limbic system | mrna levels | 9 | 86 | 62 | -0.488 | -1.101, 0.125 | 0.11839857 | -2.057, 1.081 | 64.51 | 0.54 | 0.0057 |
| haloperidol | acute | homer1a | sensory-motor areas | mrna levels | 9 | 86 | 62 | -0.081 | -0.526, 0.364 | 0.72227488 | -0.994, 0.832 | 36.58 | 0.17 | 0.0507 |
| haloperidol | acute | homer1a | ventral striatum | mrna levels | 14 | 113 | 89 | 1.81 | 1.143, 2.477 | 1.0368E-07 | -0.34, 3.96 | 72.04 | 1.09 | 0 |
| haloperidol | acute | homer1b-c | cingulate cortex | mrna levels | 3 | 35 | 21 | 0.527 | -0.319, 1.374 | 0.22198505 | -0.828, 1.883 | 52.17 | 0.29 | 0.1239 |
| haloperidol | acute | homer1b-c | dorsal striatum | mrna levels | 3 | 35 | 21 | 0.693 | 0.011, 1.375 | 0.04647557 | -0.223, 1.608 | 26.56 | 0.1 | 0.2561 |
| haloperidol | acute | homer1b-c | limbic system | mrna levels | 3 | 35 | 21 | 0.466 | -0.386, 1.318 | 0.28390108 | -0.905, 1.837 | 53.01 | 0.3 | 0.1196 |
| haloperidol | acute | homer1b-c | sensory-motor areas | mrna levels | 3 | 35 | 21 | 0.78 | 0.2, 1.359 | 0.0083775 | 0.2, 1.359 | 0 | 0 | 0.5732 |
| haloperidol | acute | homer1b-c | ventral striatum | mrna levels | 3 | 35 | 21 | 0.634 | -0.266, 1.534 | 0.16734847 | -0.848, 2.116 | 57.14 | 0.36 | 0.0967 |
| haloperidol | acute | nr1 | frontal cortex | mrna levels | 2 | 8 | 8 | -0.047 | -1.03, 0.935 | 0.9245268 | -1.03, 0.935 | 0 | 0 | 0.7036 |
| haloperidol | acute | nr1 | hippocampus | mrna levels | 2 | 8 | 8 | -0.065 | -1.046, 0.916 | 0.89611645 | -1.046, 0.916 | 0 | 0 | 0.8097 |
| haloperidol | acute | nr2a | frontal cortex | mrna levels | 2 | 8 | 8 | -0.417 | -1.408, 0.575 | 0.41011117 | -1.408, 0.575 | 0 | 0 | 0.8335 |
| haloperidol | acute | nr2a | hippocampus | mrna levels | 2 | 8 | 8 | -0.34 | -1.337, 0.657 | 0.50412495 | -1.337, 0.657 | 0 | 0 | 0.4203 |
| haloperidol | acute | nr2b | frontal cortex | mrna levels | 2 | 8 | 8 | -0.192 | -1.175, 0.791 | 0.70149005 | -1.175, 0.791 | 0 | 0 | 0.8398 |
| haloperidol | acute | nr2b | hippocampus | mrna levels | 2 | 8 | 8 | 0.051 | -0.929, 1.031 | 0.91882367 | -0.929, 1.031 | 0 | 0 | 0.9188 |
| haloperidol | acute | psd95 | cingulate cortex | mrna levels | 2 | 28 | 14 | -0.027 | -2.344, 2.29 | 0.98171794 | -3.906, 3.852 | 90.1 | 2.52 | 0.0015 |
| haloperidol | acute | psd95 | dorsal striatum | mrna levels | 3 | 33 | 19 | 0.388 | -0.802, 1.578 | 0.52284253 | -1.725, 2.5 | 72.12 | 0.79 | 0.0255 |
| haloperidol | acute | psd95 | limbic system | mrna levels | 2 | 28 | 14 | -0.155 | -0.818, 0.509 | 0.64800273 | -0.818, 0.509 | 0 | 0 | 0.898 |
| haloperidol | acute | psd95 | sensory-motor areas | mrna levels | 2 | 28 | 14 | 0.044 | -1.827, 1.914 | 0.96354758 | -3.037, 3.124 | 85.61 | 1.56 | 0.0084 |
| haloperidol | acute | psd95 | ventral striatum | mrna levels | 3 | 33 | 19 | -0.221 | -1.842, 1.399 | 0.78885952 | -3.254, 2.811 | 83.82 | 1.71 | 0.0037 |
| haloperidol | chronic | ania3 | associative cortex | mrna levels | 2 | 12 | 12 | -0.453 | -1.266, 0.36 | 0.275061 | -1.266, 0.36 | 0 | 0 | 0.5593 |
| haloperidol | chronic | ania3 | cingulate cortex | mrna levels | 2 | 11 | 11 | 0.825 | -0.053, 1.704 | 0.065472 | -0.053, 1.704 | 0 | 0 | 0.3797 |
| haloperidol | chronic | ania3 | dorsal striatum | mrna levels | 3 | 16 | 16 | 2.942 | 1.934, 3.951 | 1.0805E-08 | 1.934, 3.951 | 0 | 0 | 0.5769 |
| haloperidol | chronic | ania3 | frontal cortex | mrna levels | 2 | 12 | 12 | -0.59 | -1.41, 0.23 | 0.1586649 | -1.41, 0.23 | 0 | 0 | 0.5667 |
| haloperidol | chronic | ania3 | ventral striatum | mrna levels | 2 | 11 | 11 | 1.124 | 0.225, 2.024 | 0.01428023 | 0.225, 2.024 | 0 | 0 | 0.9443 |
| haloperidol | chronic | arc | cingulate cortex | mrna levels | 3 | 27 | 17 | -0.706 | -1.892, 0.48 | 0.24339115 | -2.782, 1.37 | 68.75 | 0.76 | 0.0413 |
| haloperidol | chronic | arc | dorsal striatum | mrna levels | 4 | 41 | 31 | 0.636 | -0.88, 2.151 | 0.41107803 | -2.551, 3.823 | 86.73 | 2.05 | 0 |
| haloperidol | chronic | arc | limbic system | mrna levels | 3 | 27 | 17 | -0.62 | -1.697, 0.457 | 0.2589732 | -2.449, 1.209 | 62.88 | 0.57 | 0.0679 |
| haloperidol | chronic | arc | sensory-motor areas | mrna levels | 3 | 27 | 17 | -1.186 | -2.82, 0.448 | 0.15500049 | -4.22, 1.848 | 81.71 | 1.7 | 0.0065 |
| haloperidol | chronic | arc | ventral striatum | mrna levels | 3 | 27 | 17 | 0.451 | -0.185, 1.087 | 0.16483476 | -0.185, 1.087 | 0 | 0 | 0.8622 |
| haloperidol | chronic | homer1a | associative cortex | mrna levels | 2 | 12 | 12 | -0.916 | -1.758, -0.073 | 0.03312873 | -1.758, -0.073 | 0 | 0 | 0.6848 |
| haloperidol | chronic | homer1a | cingulate cortex | mrna levels | 9 | 81 | 61 | 0.139 | -0.69, 0.967 | 0.74260251 | -2.192, 2.47 | 79.55 | 1.24 | 0.0001 |
| haloperidol | chronic | homer1a | dorsal striatum | mrna levels | 10 | 86 | 66 | 1.692 | 1.146, 2.238 | 1.2267E-09 | 0.413, 2.972 | 46.47 | 0.35 | 0.0581 |
| haloperidol | chronic | homer1a | frontal cortex | mrna levels | 2 | 12 | 12 | -0.74 | -1.568, 0.088 | 0.07992892 | -1.568, 0.088 | 0 | 0 | 0.7272 |
| haloperidol | chronic | homer1a | limbic system | mrna levels | 8 | 74 | 54 | 0.145 | -0.738, 1.027 | 0.74798984 | -2.246, 2.535 | 79.91 | 1.28 | 0 |
| haloperidol | chronic | homer1a | sensory-motor areas | mrna levels | 8 | 74 | 54 | 0.296 | -0.745, 1.338 | 0.57722923 | -2.579, 3.171 | 84.78 | 1.87 | 0 |
| haloperidol | chronic | homer1a | ventral striatum | mrna levels | 9 | 81 | 61 | 1.182 | 0.771, 1.593 | 1.7094E-08 | 0.547, 1.817 | 15.48 | 0.06 | 0.342 |
| haloperidol | chronic | homer1a | ventral striatum | protein levels | 2 | 12 | 12 | 0.501 | -0.315, 1.317 | 0.22904963 | -0.315, 1.317 | 0 | 0 | 0.5335 |
| haloperidol | chronic | homer1b-c | cingulate cortex | mrna levels | 6 | 52 | 42 | 0.025 | -0.406, 0.456 | 0.90882645 | -0.406, 0.456 | 0 | 0 | 0.023 |
| haloperidol | chronic | homer1b-c | dorsal striatum | mrna levels | 6 | 52 | 42 | 0.418 | -0.213, 1.049 | 0.19438711 | -0.833, 1.668 | 50.07 | 0.3 | 0.0694 |
| haloperidol | chronic | homer1b-c | limbic system | mrna levels | 6 | 52 | 42 | 0.517 | -0.294, 1.329 | 0.21142841 | -1.294, 2.329 | 68.43 | 0.68 | 0.0063 |
| haloperidol | chronic | homer1b-c | sensory-motor areas | mrna levels | 6 | 52 | 42 | 0.117 | -0.309, 0.543 | 0.59035646 | -0.309, 0.543 | 0 | 0 | 0.1156 |
| haloperidol | chronic | homer1b-c | ventral striatum | mrna levels | 6 | 52 | 42 | 0.684 | 0.034, 1.334 | 0.03906996 | -0.623, 1.991 | 51.75 | 0.33 | 0.059 |
| haloperidol | chronic | homer1b-c | ventral striatum | protein levels | 2 | 12 | 12 | -0.13 | -1.579, 1.318 | 0.85999021 | -2.349, 2.088 | 67.33 | 0.74 | 0.0802 |
| haloperidol | chronic | mglur1 | frontal cortex | mrna levels | 2 | 10 | 10 | 0.083 | -0.796, 0.962 | 0.85313923 | -0.796, 0.962 | 0 | 0 | 0.6545 |
| haloperidol | chronic | mglur1 | ventral striatum | protein levels | 2 | 12 | 12 | -0.518 | -1.333, 0.296 | 0.21198665 | -1.333, 0.296 | 0 | 0 | 0.7791 |
| haloperidol | chronic | mglur2 | frontal cortex | mrna levels | 2 | 10 | 10 | -0.513 | -1.405, 0.379 | 0.25928373 | -1.405, 0.379 | 0 | 0 | 0.7632 |
| haloperidol | chronic | mglur5 | frontal cortex | mrna levels | 2 | 10 | 10 | -0.098 | -0.986, 0.789 | 0.82860465 | -0.986, 0.789 | 0 | 0 | 0.3326 |
| haloperidol | chronic | mglur5 | frontal cortex | protein levels | 2 | 12 | 12 | 0.103 | -0.698, 0.904 | 0.80090662 | -0.698, 0.904 | 0 | 0 | 0.7728 |
| haloperidol | chronic | mglur5 | ventral striatum | protein levels | 2 | 12 | 12 | 0.138 | -0.668, 0.945 | 0.73677055 | -0.668, 0.945 | 0 | 0 | 0.4317 |
| haloperidol | chronic | norbin | frontal cortex | protein levels | 2 | 12 | 12 | -0.469 | -2.552, 1.614 | 0.65892406 | -3.863, 2.925 | 82.72 | 1.87 | 0.0161 |
| haloperidol | chronic | norbin | ventral striatum | protein levels | 2 | 12 | 12 | 1.004 | -0.129, 2.137 | 0.0823441 | -0.531, 2.539 | 41.51 | 0.28 | 0.191 |
| haloperidol | chronic | nr1 | dorsal striatum | mrna levels | 2 | 9 | 9 | 0.777 | -0.182, 1.737 | 0.11242595 | -0.182, 1.737 | 0 | 0 | 0.7497 |
| haloperidol | chronic | nr1 | dorsal striatum | protein levels | 2 | 11 | 11 | 0.108 | -0.732, 0.948 | 0.8006356 | -0.732, 0.948 | 0 | 0 | 0.529 |
| haloperidol | chronic | nr1 | frontal cortex | protein levels | 3 | 17 | 17 | -0.333 | -1.557, 0.892 | 0.59435677 | -2.45, 1.785 | 66.44 | 0.78 | 0.0537 |
| haloperidol | chronic | nr1 | hippocampus | protein levels | 2 | 11 | 11 | 0.136 | -0.704, 0.977 | 0.75077067 | -0.704, 0.977 | 0 | 0 | 0.5371 |
| haloperidol | chronic | nr1 | ventral striatum | protein levels | 2 | 11 | 11 | 0.751 | -0.675, 2.176 | 0.30198566 | -1.373, 2.874 | 60.98 | 0.64 | 0.1094 |
| haloperidol | chronic | nr2a | dorsal striatum | protein levels | 2 | 11 | 11 | 0.42 | -0.425, 1.265 | 0.32985018 | -0.425, 1.265 | 0 | 0 | 0.899 |
| haloperidol | chronic | nr2a | frontal cortex | protein levels | 3 | 17 | 17 | -0.95 | -1.828, -0.072 | 0.03386986 | -2.176, 0.275 | 31.61 | 0.19 | 0.2228 |
| haloperidol | chronic | nr2a | hippocampus | protein levels | 2 | 11 | 11 | -0.856 | -2.075, 0.364 | 0.16919393 | -2.546, 0.834 | 45.36 | 0.36 | 0.1761 |
| haloperidol | chronic | nr2a | ventral striatum | protein levels | 2 | 11 | 11 | 0.086 | -0.755, 0.927 | 0.84077202 | -0.755, 0.927 | 0 | 0 | 0.4828 |
| haloperidol | chronic | nr2b | frontal cortex | protein levels | 2 | 11 | 11 | 0.35 | -0.936, 1.635 | 0.5939771 | -1.508, 2.207 | 54.2 | 0.47 | 0.1395 |
| haloperidol | chronic | nr2b | hippocampus | protein levels | 2 | 11 | 11 | -0.917 | -2.739, 0.904 | 0.32368087 | -3.786, 1.951 | 73.6 | 1.28 | 0.0516 |
| haloperidol | chronic | preso1 | frontal cortex | protein levels | 2 | 12 | 12 | 0.041 | -0.76, 0.842 | 0.91960294 | -0.76, 0.842 | 0 | 0 | 0.73 |
| haloperidol | chronic | psd95 | cingulate cortex | mrna levels | 5 | 45 | 35 | 0.251 | -1.452, 1.954 | 0.7726198 | -3.719, 4.221 | 90.03 | 3.35 | 0 |
| haloperidol | chronic | psd95 | dorsal striatum | mrna levels | 5 | 45 | 35 | 0.351 | -0.821, 1.524 | 0.55668069 | -2.261, 2.964 | 81.2 | 1.42 | 0.0002 |
| haloperidol | chronic | psd95 | frontal cortex | protein levels | 3 | 16 | 16 | -0.25 | -0.947, 0.446 | 0.48087516 | -0.947, 0.446 | 0 | 0 | 0.9381 |
| haloperidol | chronic | psd95 | limbic system | mrna levels | 5 | 45 | 35 | -0.301 | -1.306, 0.704 | 0.55702181 | -2.478, 1.876 | 75.36 | 0.97 | 0.0016 |
| haloperidol | chronic | psd95 | sensory-motor areas | mrna levels | 5 | 45 | 35 | 0.09 | -1.156, 1.337 | 0.88692017 | -2.723, 2.904 | 83.33 | 1.66 | 0.0001 |
| haloperidol | chronic | psd95 | ventral striatum | mrna levels | 5 | 45 | 35 | 0.162 | -0.739, 1.064 | 0.72411068 | -1.746, 2.071 | 70.57 | 0.74 | 0.0037 |
| haloperidol | chronic | tamalin | frontal cortex | protein levels | 2 | 12 | 12 | 0.135 | -0.666, 0.936 | 0.74079611 | -0.666, 0.936 | 0 | 0 | 0.9777 |
| olanzapine | acute | arc | dorsal striatum | mrna levels | 3 | 48 | 48 | 1.41 | 0.374, 2.446 | 0.00766289 | -0.482, 3.302 | 78.54 | 0.65 | 0.0162 |
| olanzapine | acute | arc | frontal cortex | mrna levels | 2 | 41 | 41 | -2.154 | -2.699, -1.61 | 8.71E-15 | -2.699, -1.61 | 0 | 0 | 0.737 |
| olanzapine | acute | arc | ventral striatum | mrna levels | 2 | 27 | 27 | 1.486 | 0.882, 2.091 | 1.4558E-06 | 0.882, 2.091 | 0 | 0 | 0.387 |
| olanzapine | acute | homer1a | cingulate cortex | mrna levels | 2 | 12 | 12 | 0.701 | -0.967, 2.368 | 0.41011729 | -1.918, 3.32 | 73.34 | 1.06 | 0.0528 |
| olanzapine | acute | homer1a | dorsal striatum | mrna levels | 4 | 22 | 22 | 3.442 | -1.378, 8.261 | 0.16162121 | -6.974, 13.857 | 97.2 | 22.19 | 0.0001 |
| olanzapine | acute | homer1a | limbic system | mrna levels | 2 | 12 | 12 | 0.947 | -0.16, 2.053 | 0.09368945 | -0.535, 2.428 | 39.54 | 0.25 | 0.1984 |
| olanzapine | acute | homer1a | sensory-motor areas | mrna levels | 2 | 12 | 12 | 0.641 | -0.674, 1.957 | 0.33920261 | -1.298, 2.581 | 58.66 | 0.53 | 0.1199 |
| olanzapine | acute | homer1a | ventral striatum | mrna levels | 4 | 22 | 22 | 1.845 | 0.27, 3.419 | 0.02164256 | -1.318, 5.007 | 77.74 | 1.96 | 0.0086 |
| olanzapine | acute | psd95 | dorsal striatum | mrna levels | 2 | 12 | 12 | 2.066 | -0.591, 4.723 | 0.12756396 | -2.294, 6.426 | 84.44 | 3.11 | 0.0113 |
| olanzapine | acute | psd95 | frontal cortex | protein levels | 6 | 36 | 36 | 2.827 | -0.45, 6.105 | 0.09088374 | -5.553, 11.208 | 96.66 | 15.49 | 0 |
| olanzapine | acute | psd95 | hippocampus | protein levels | 6 | 36 | 36 | -0.136 | -0.619, 0.348 | 0.58238693 | -0.685, 0.414 | 4.85 | 0.02 | 0.3512 |
| olanzapine | acute | psd95 | ventral striatum | mrna levels | 2 | 12 | 12 | 1.438 | -1.025, 3.9 | 0.25247242 | -2.608, 5.483 | 84.92 | 2.68 | 0.01 |
| olanzapine | chronic | arc | dorsal striatum | mrna levels | 2 | 19 | 19 | -1.597 | -2.328, -0.867 | 1.8322E-05 | -2.328, -0.867 | 0 | 0 | 0.7685 |
| olanzapine | chronic | homer1a | cingulate cortex | mrna levels | 2 | 10 | 10 | 0.2 | -0.686, 1.085 | 0.65825126 | -0.686, 1.085 | 0 | 0 | 0.4347 |
| olanzapine | chronic | homer1a | dorsal striatum | mrna levels | 2 | 10 | 10 | 0.342 | -0.631, 1.314 | 0.49105315 | -0.767, 1.45 | 15 | 0.07 | 0.2781 |
| olanzapine | chronic | homer1a | limbic system | mrna levels | 2 | 10 | 10 | -0.245 | -1.127, 0.636 | 0.58547176 | -1.127, 0.636 | 0 | 0 | 0.6992 |
| olanzapine | chronic | homer1a | sensory-motor areas | mrna levels | 2 | 10 | 10 | 0.322 | -0.789, 1.432 | 0.57038596 | -1.121, 1.764 | 34.37 | 0.22 | 0.2171 |
| olanzapine | chronic | homer1a | ventral striatum | mrna levels | 2 | 10 | 10 | 1.185 | -1.506, 3.875 | 0.38814612 | -3.237, 5.606 | 84.85 | 3.2 | 0.0102 |
| olanzapine | chronic | mglur5 | frontal cortex | protein levels | 2 | 12 | 12 | 0.222 | -0.581, 1.025 | 0.58811116 | -0.581, 1.025 | 0 | 0 | 0.7776 |
| olanzapine | chronic | norbin | frontal cortex | protein levels | 2 | 12 | 12 | -0.039 | -0.84, 0.763 | 0.92462699 | -0.84, 0.763 | 0 | 0 | 0.6702 |
| olanzapine | chronic | nr1 | frontal cortex | protein levels | 3 | 16 | 16 | 0.481 | -0.539, 1.501 | 0.35529501 | -1.121, 2.083 | 48.86 | 0.4 | 0.1423 |
| olanzapine | chronic | nr1 | hippocampus | protein levels | 2 | 12 | 12 | 0.044 | -0.758, 0.846 | 0.91493488 | -0.758, 0.846 | 0 | 0 | 0.6353 |
| olanzapine | chronic | nr2a | frontal cortex | protein levels | 2 | 12 | 12 | -0.064 | -1.2, 1.071 | 0.91150552 | -1.656, 1.528 | 48.25 | 0.32 | 0.1645 |
| olanzapine | chronic | nr2a | hippocampus | protein levels | 2 | 12 | 12 | 0.167 | -0.635, 0.97 | 0.68288726 | -0.635, 0.97 | 0 | 0 | 0.7553 |
| olanzapine | chronic | nr2b | hippocampus | protein levels | 2 | 12 | 12 | 0.148 | -0.661, 0.958 | 0.71966027 | -0.661, 0.958 | 0 | 0 | 0.3205 |
| olanzapine | chronic | preso1 | frontal cortex | protein levels | 2 | 12 | 12 | 0.065 | -0.736, 0.865 | 0.87449228 | -0.736, 0.865 | 0 | 0 | 0.8204 |
| olanzapine | chronic | psd95 | frontal cortex | protein levels | 3 | 16 | 16 | -0.095 | -1.294, 1.103 | 0.87593782 | -2.136, 1.945 | 63.3 | 0.71 | 0.0667 |
| olanzapine | chronic | tamalin | frontal cortex | protein levels | 2 | 12 | 12 | 0.399 | -0.413, 1.212 | 0.33558894 | -0.413, 1.212 | 0 | 0 | 0.4663 |
| quetiapine | acute | homer1a | dorsal striatum | mrna levels | 2 | 24 | 12 | 0.436 | -0.264, 1.136 | 0.22239809 | -0.264, 1.136 | 0 | 0 | 0.9673 |
| quetiapine | chronic | homer1a | dorsal striatum | mrna levels | 2 | 19 | 12 | 0.648 | -0.822, 2.119 | 0.38764893 | -1.624, 2.921 | 68.25 | 0.78 | 0.076 |

## Sensitivity analyses based on treatment duration

| **Sensitivity analyses based on treatment duration** | | | | | | | | | | | | | | |
| --- | --- | --- | --- | --- | --- | --- | --- | --- | --- | --- | --- | --- | --- | --- |
| **drug** | **model** | **psd type** | **region** | **outcome type** | **N_studies** | **n_group_1** | **n_group_2** | **beta** | **CI** | **p_val** | **PI** | **i_sq** | **tau_sq** | **q_p_val** |
| amisulpride | chronic | arc | cingulate cortex | mrna levels | 2 | 12 | 12 | 0.22 | -0.583, 1.024 | 0.59115941 | -0.583, 1.024 | 0 | 0 | 0.7398 |
| amisulpride | chronic | arc | dorsal striatum | mrna levels | 2 | 12 | 12 | 1.235 | 0.359, 2.111 | 0.00573881 | 0.359, 2.111 | 0 | 0 | 0.5518 |
| amisulpride | chronic | arc | limbic system | mrna levels | 2 | 12 | 12 | 0.454 | -0.485, 1.393 | 0.34349765 | -0.684, 1.592 | 23.38 | 0.11 | 0.2533 |
| amisulpride | chronic | arc | sensory-motor areas | mrna levels | 2 | 12 | 12 | 0.12 | -0.685, 0.925 | 0.77032363 | -0.685, 0.925 | 0 | 0 | 0.476 |
| amisulpride | chronic | arc | ventral striatum | mrna levels | 2 | 12 | 12 | 0.825 | -0.01, 1.66 | 0.05270135 | -0.01, 1.66 | 0 | 0 | 0.6841 |
| amisulpride | chronic | homer1a | cingulate cortex | mrna levels | 2 | 12 | 12 | 1.355 | -1.757, 4.466 | 0.39347591 | -3.848, 6.557 | 89.66 | 4.53 | 0.0019 |
| amisulpride | chronic | homer1a | dorsal striatum | mrna levels | 2 | 12 | 12 | 1.804 | -0.412, 4.02 | 0.11065992 | -1.764, 5.372 | 79.2 | 2.04 | 0.0283 |
| amisulpride | chronic | homer1a | limbic system | mrna levels | 2 | 12 | 12 | 2.678 | -3.045, 8.401 | 0.3590189 | -7.041, 12.397 | 94.06 | 16.06 | 0 |
| amisulpride | chronic | homer1a | sensory-motor areas | mrna levels | 2 | 12 | 12 | 0.575 | -2.134, 3.285 | 0.67732768 | -3.94, 5.091 | 88.81 | 3.4 | 0.0028 |
| amisulpride | chronic | homer1a | ventral striatum | mrna levels | 2 | 12 | 12 | 0.982 | -0.103, 2.067 | 0.07613854 | -0.449, 2.413 | 36.76 | 0.23 | 0.2086 |
| amisulpride | chronic | homer1b-c | cingulate cortex | mrna levels | 2 | 12 | 12 | 0.189 | -1.742, 2.12 | 0.84805566 | -2.933, 3.31 | 80.66 | 1.57 | 0.023 |
| amisulpride | chronic | homer1b-c | dorsal striatum | mrna levels | 2 | 12 | 12 | 0.972 | -2.829, 4.772 | 0.61623365 | -5.454, 7.398 | 92.92 | 6.99 | 0.0002 |
| amisulpride | chronic | homer1b-c | limbic system | mrna levels | 2 | 12 | 12 | 0.388 | -2.039, 2.814 | 0.7542967 | -3.627, 4.402 | 86.82 | 2.66 | 0.0059 |
| amisulpride | chronic | homer1b-c | sensory-motor areas | mrna levels | 2 | 12 | 12 | 0.282 | -1.513, 2.078 | 0.75789314 | -2.589, 3.154 | 77.85 | 1.31 | 0.0336 |
| amisulpride | chronic | homer1b-c | ventral striatum | mrna levels | 2 | 12 | 12 | 1.215 | -0.913, 3.344 | 0.26305651 | -2.232, 4.663 | 80.98 | 1.91 | 0.0218 |
| amisulpride | chronic | psd95 | cingulate cortex | mrna levels | 2 | 12 | 12 | -0.006 | -0.806, 0.794 | 0.9882636 | -0.806, 0.794 | 0 | 0 | 0.883 |
| amisulpride | chronic | psd95 | dorsal striatum | mrna levels | 2 | 12 | 12 | -0.438 | -1.825, 0.949 | 0.53623677 | -2.53, 1.655 | 63.79 | 0.64 | 0.0966 |
| amisulpride | chronic | psd95 | limbic system | mrna levels | 2 | 12 | 12 | -0.507 | -1.337, 0.324 | 0.23176349 | -1.355, 0.341 | 2.11 | 0.01 | 0.3121 |
| amisulpride | chronic | psd95 | sensory-motor areas | mrna levels | 2 | 12 | 12 | -0.063 | -0.864, 0.737 | 0.87678829 | -0.864, 0.737 | 0 | 0 | 0.8767 |
| amisulpride | chronic | psd95 | ventral striatum | mrna levels | 2 | 12 | 12 | -0.08 | -0.882, 0.722 | 0.844924 | -0.882, 0.722 | 0 | 0 | 0.6302 |
| aripiprazole | acute | arc | dorsal striatum | mrna levels | 2 | 35 | 25 | 1.006 | -2.546, 4.558 | 0.5788137 | -5.071, 7.083 | 96.33 | 6.33 | 0 |
| aripiprazole | acute | arc | frontal cortex | mrna levels | 2 | 35 | 25 | -2.268 | -4.586, 0.051 | 0.05521776 | -6.151, 1.616 | 90.29 | 2.53 | 0.0013 |
| aripiprazole | chronic | nr1 | frontal cortex | protein levels | 2 | 12 | 12 | -0.002 | -0.802, 0.798 | 0.99583721 | -0.802, 0.798 | 0 | 0 | 0.9958 |
| aripiprazole | chronic | nr1 | ventral striatum | protein levels | 2 | 12 | 12 | 0.321 | -1.858, 2.501 | 0.77254555 | -3.25, 3.892 | 84.25 | 2.08 | 0.0117 |
| aripiprazole | chronic | psd95 | frontal cortex | protein levels | 2 | 10 | 10 | 1.46 | 0.47, 2.45 | 0.00385209 | 0.47, 2.45 | 0 | 0 | 0.5864 |
| asenapine | acute | homer1a | cingulate cortex | mrna levels | 4 | 46 | 22 | 0.122 | -0.794, 1.038 | 0.79451752 | -1.593, 1.836 | 63.3 | 0.55 | 0.0438 |
| asenapine | acute | homer1a | dorsal striatum | mrna levels | 4 | 46 | 22 | 1.019 | 0.464, 1.574 | 0.00031943 | 0.464, 1.574 | 0 | 0 | 0.4963 |
| asenapine | acute | homer1a | hippocampus | mrna levels | 2 | 10 | 10 | -0.751 | -3.629, 2.127 | 0.60926092 | -5.524, 4.023 | 87.51 | 3.78 | 0.0047 |
| asenapine | acute | homer1a | limbic system | mrna levels | 4 | 46 | 22 | 0.274 | -0.344, 0.893 | 0.38436891 | -0.584, 1.133 | 22.98 | 0.09 | 0.2417 |
| asenapine | acute | homer1a | sensory-motor areas | mrna levels | 4 | 46 | 22 | 0.21 | -0.557, 0.978 | 0.59128363 | -1.104, 1.525 | 48.69 | 0.3 | 0.1174 |
| asenapine | acute | homer1a | ventral striatum | mrna levels | 4 | 46 | 22 | 0.57 | -0.239, 1.379 | 0.16711397 | -0.857, 1.997 | 53.13 | 0.36 | 0.0923 |
| asenapine | chronic | homer1a | cingulate cortex | mrna levels | 2 | 30 | 10 | -0.064 | -0.78, 0.652 | 0.86107453 | -0.78, 0.652 | 0 | 0 | 0.861 |
| asenapine | chronic | homer1a | dorsal striatum | mrna levels | 2 | 30 | 10 | 0.137 | -0.782, 1.057 | 0.76951282 | -1.083, 1.358 | 38.08 | 0.17 | 0.2038 |
| asenapine | chronic | homer1a | limbic system | mrna levels | 2 | 30 | 10 | -1.231 | -2.076, -0.385 | 0.0043296 | -2.208, -0.253 | 16.8 | 0.06 | 0.2729 |
| asenapine | chronic | homer1a | sensory-motor areas | mrna levels | 2 | 30 | 10 | -0.06 | -0.776, 0.657 | 0.86965689 | -0.776, 0.657 | 0 | 0 | 0.6934 |
| asenapine | chronic | homer1a | ventral striatum | mrna levels | 2 | 30 | 10 | 0.431 | -0.458, 1.32 | 0.34183304 | -0.713, 1.576 | 32.81 | 0.14 | 0.2225 |
| clozapine | acute | arc | dorsal striatum | mrna levels | 3 | 32 | 32 | -0.135 | -0.703, 0.434 | 0.64262337 | -0.843, 0.574 | 16.22 | 0.05 | 0.3683 |
| clozapine | acute | arc | frontal cortex | mrna levels | 3 | 32 | 32 | -0.914 | -2.08, 0.252 | 0.12460827 | -3.011, 1.183 | 75.01 | 0.79 | 0.0149 |
| clozapine | acute | arc | ventral striatum | mrna levels | 2 | 25 | 25 | -0.019 | -1.872, 1.834 | 0.98390548 | -3.065, 3.027 | 84.34 | 1.52 | 0.0115 |
| clozapine | acute | homer1a | cingulate cortex | mrna levels | 2 | 12 | 12 | 2.119 | 1.119, 3.119 | 3.2667E-05 | 1.119, 3.119 | 0 | 0 | 0.9358 |
| clozapine | acute | homer1a | dorsal striatum | mrna levels | 3 | 17 | 17 | 1.131 | 0.406, 1.856 | 0.00223067 | 0.406, 1.856 | 0 | 0 | 0.9033 |
| clozapine | acute | homer1a | ventral striatum | mrna levels | 3 | 17 | 17 | 1.478 | 0.716, 2.239 | 0.00014279 | 0.716, 2.239 | 0 | 0 | 0.7652 |
| clozapine | acute | mglur1 | frontal cortex | mrna levels | 2 | 10 | 10 | 0.931 | -0.036, 1.898 | 0.05904279 | -0.095, 1.957 | 6.29 | 0.03 | 0.3016 |
| clozapine | acute | mglur2 | frontal cortex | mrna levels | 2 | 10 | 10 | 0.042 | -0.839, 0.923 | 0.92540925 | -0.839, 0.923 | 0 | 0 | 0.5292 |
| clozapine | acute | mglur5 | frontal cortex | mrna levels | 2 | 10 | 10 | -0.651 | -1.551, 0.249 | 0.15624973 | -1.551, 0.249 | 0 | 0 | 0.7929 |
| clozapine | chronic | mglur1 | frontal cortex | mrna levels | 2 | 20 | 10 | 0.189 | -0.572, 0.95 | 0.62714207 | -0.572, 0.95 | 0 | 0 | 0.7714 |
| clozapine | chronic | mglur2 | frontal cortex | mrna levels | 3 | 25 | 15 | 0.106 | -0.552, 0.764 | 0.75178475 | -0.552, 0.764 | 0 | 0 | 0.3195 |
| clozapine | chronic | mglur5 | frontal cortex | mrna levels | 3 | 25 | 15 | 0.079 | -0.568, 0.727 | 0.81067085 | -0.568, 0.727 | 0 | 0 | 0.9975 |
| clozapine | chronic | nr1 | dorsal striatum | mrna levels | 4 | 29 | 29 | 0.89 | -0.876, 2.656 | 0.3230521 | -2.828, 4.609 | 88.91 | 2.79 | 0.0019 |
| clozapine | chronic | nr1 | dorsal striatum | protein levels | 2 | 17 | 17 | 0.761 | 0.065, 1.457 | 0.03219395 | 0.065, 1.457 | 0 | 0 | 0.9034 |
| clozapine | chronic | nr1 | frontal cortex | mrna levels | 4 | 35 | 35 | 0.138 | -0.545, 0.822 | 0.69196945 | -1.043, 1.319 | 49.79 | 0.24 | 0.1153 |
| clozapine | chronic | nr1 | frontal cortex | protein levels | 2 | 17 | 17 | -0.566 | -1.255, 0.123 | 0.10753823 | -1.255, 0.123 | 0 | 0 | 0.4038 |
| clozapine | chronic | nr1 | hippocampus | mrna levels | 3 | 24 | 24 | 0.656 | 0.007, 1.304 | 0.04744702 | -0.14, 1.451 | 16.55 | 0.06 | 0.2679 |
| clozapine | chronic | nr1 | hippocampus | protein levels | 4 | 28 | 28 | -0.285 | -0.989, 0.418 | 0.42680278 | -1.395, 0.824 | 37.1 | 0.19 | 0.1764 |
| clozapine | chronic | nr1 | ventral striatum | protein levels | 2 | 17 | 17 | 0.076 | -0.736, 0.888 | 0.85489505 | -0.929, 1.08 | 23.83 | 0.09 | 0.2519 |
| clozapine | chronic | nr2a | dorsal striatum | mrna levels | 2 | 15 | 15 | 0.445 | -0.719, 1.609 | 0.45363294 | -1.26, 2.15 | 56.34 | 0.4 | 0.1302 |
| clozapine | chronic | nr2a | frontal cortex | mrna levels | 2 | 21 | 21 | -0.565 | -1.182, 0.052 | 0.07251751 | -1.182, 0.052 | 0 | 0 | 0.9514 |
| clozapine | chronic | nr2a | hippocampus | mrna levels | 2 | 21 | 21 | -0.344 | -0.953, 0.266 | 0.26964694 | -0.953, 0.266 | 0 | 0 | 0.685 |
| clozapine | chronic | nr2a | hippocampus | protein levels | 2 | 11 | 11 | -0.29 | -1.137, 0.556 | 0.50104712 | -1.137, 0.556 | 0 | 0 | 0.4319 |
| clozapine | chronic | nr2b | dorsal striatum | mrna levels | 2 | 15 | 15 | 0.895 | -0.007, 1.797 | 0.0517365 | -0.218, 2.008 | 23.35 | 0.11 | 0.2534 |
| clozapine | chronic | nr2b | hippocampus | protein levels | 2 | 11 | 11 | -0.773 | -2.516, 0.969 | 0.38441126 | -3.501, 1.955 | 72.22 | 1.15 | 0.0578 |
| haloperidol | acute | ania3 | associative cortex | mrna levels | 3 | 17 | 17 | 0.018 | -0.977, 1.013 | 0.97178106 | -1.565, 1.601 | 51.03 | 0.39 | 0.1294 |
| haloperidol | acute | ania3 | cingulate cortex | mrna levels | 4 | 23 | 23 | 0.997 | -0.261, 2.255 | 0.12030827 | -1.478, 3.472 | 72.81 | 1.18 | 0.0125 |
| haloperidol | acute | ania3 | dorsal striatum | mrna levels | 5 | 28 | 29 | 2.471 | 1.076, 3.866 | 0.00051852 | -0.521, 5.463 | 73.64 | 1.82 | 0.0053 |
| haloperidol | acute | ania3 | frontal cortex | mrna levels | 3 | 17 | 17 | -0.136 | -0.817, 0.545 | 0.69530376 | -0.817, 0.545 | 0 | 0 | 0.4424 |
| haloperidol | acute | ania3 | limbic system | mrna levels | 2 | 11 | 11 | 0.615 | -0.241, 1.471 | 0.15884517 | -0.241, 1.471 | 0 | 0 | 0.8625 |
| haloperidol | acute | ania3 | sensory-motor areas | mrna levels | 2 | 11 | 11 | 0.384 | -0.901, 1.67 | 0.5578257 | -1.454, 2.223 | 50.8 | 0.45 | 0.154 |
| haloperidol | acute | ania3 | ventral striatum | mrna levels | 5 | 28 | 29 | 1.425 | 0.607, 2.243 | 0.00064121 | -0.056, 2.906 | 45.76 | 0.4 | 0.1156 |
| haloperidol | acute | arc | cingulate cortex | mrna levels | 5 | 46 | 32 | 0.022 | -0.932, 0.976 | 0.96388064 | -2.026, 2.07 | 72.9 | 0.85 | 0.0035 |
| haloperidol | acute | arc | dorsal striatum | mrna levels | 10 | 114 | 90 | 2.598 | 1.338, 3.857 | 5.2739E-05 | -1.292, 6.488 | 89.96 | 3.53 | 0 |
| haloperidol | acute | arc | frontal cortex | mrna levels | 5 | 68 | 58 | -0.949 | -1.327, -0.57 | 9.0674E-07 | -1.327, -0.57 | 0 | 0 | 0.3895 |
| haloperidol | acute | arc | limbic system | mrna levels | 5 | 46 | 32 | -0.212 | -1.183, 0.759 | 0.66867532 | -2.303, 1.879 | 73.57 | 0.89 | 0.0051 |
| haloperidol | acute | arc | sensory-motor areas | mrna levels | 5 | 46 | 32 | -0.36 | -1.163, 0.442 | 0.3789775 | -1.984, 1.263 | 62.44 | 0.52 | 0.0278 |
| haloperidol | acute | arc | ventral striatum | mrna levels | 7 | 71 | 57 | 1.73 | 1.138, 2.321 | 9.8977E-09 | 0.55, 2.909 | 43.97 | 0.27 | 0.0779 |
| haloperidol | acute | glua1 pser845 | dorsal striatum | protein levels | 4 | 66 | 30 | 2.562 | 0.681, 4.442 | 0.00758779 | -1.381, 6.505 | 89.74 | 3.13 | 0.0009 |
| haloperidol | acute | homer1a | associative cortex | mrna levels | 3 | 17 | 17 | -0.361 | -1.47, 0.749 | 0.52395279 | -2.203, 1.482 | 58.76 | 0.56 | 0.0945 |
| haloperidol | acute | homer1a | cingulate cortex | mrna levels | 11 | 98 | 74 | 0.151 | -0.266, 0.568 | 0.47875811 | -0.8, 1.101 | 39.03 | 0.19 | 0.0559 |
| haloperidol | acute | homer1a | dorsal striatum | mrna levels | 15 | 118 | 94 | 2.114 | 1.428, 2.8 | 1.5719E-09 | -0.097, 4.325 | 70.36 | 1.15 | 0 |
| haloperidol | acute | homer1a | frontal cortex | mrna levels | 3 | 17 | 17 | 0.033 | -0.647, 0.713 | 0.92422709 | -0.647, 0.713 | 0 | 0 | 0.4486 |
| haloperidol | acute | homer1a | limbic system | mrna levels | 9 | 86 | 62 | -0.488 | -1.101, 0.125 | 0.11839857 | -2.057, 1.081 | 64.51 | 0.54 | 0.0057 |
| haloperidol | acute | homer1a | sensory-motor areas | mrna levels | 9 | 86 | 62 | -0.081 | -0.526, 0.364 | 0.72227488 | -0.994, 0.832 | 36.58 | 0.17 | 0.0507 |
| haloperidol | acute | homer1a | ventral striatum | mrna levels | 14 | 113 | 89 | 1.81 | 1.143, 2.477 | 1.0368E-07 | -0.34, 3.96 | 72.04 | 1.09 | 0 |
| haloperidol | acute | homer1b-c | cingulate cortex | mrna levels | 3 | 35 | 21 | 0.527 | -0.319, 1.374 | 0.22198505 | -0.828, 1.883 | 52.17 | 0.29 | 0.1239 |
| haloperidol | acute | homer1b-c | dorsal striatum | mrna levels | 3 | 35 | 21 | 0.693 | 0.011, 1.375 | 0.04647557 | -0.223, 1.608 | 26.56 | 0.1 | 0.2561 |
| haloperidol | acute | homer1b-c | limbic system | mrna levels | 3 | 35 | 21 | 0.466 | -0.386, 1.318 | 0.28390108 | -0.905, 1.837 | 53.01 | 0.3 | 0.1196 |
| haloperidol | acute | homer1b-c | sensory-motor areas | mrna levels | 3 | 35 | 21 | 0.78 | 0.2, 1.359 | 0.0083775 | 0.2, 1.359 | 0 | 0 | 0.5732 |
| haloperidol | acute | homer1b-c | ventral striatum | mrna levels | 3 | 35 | 21 | 0.634 | -0.266, 1.534 | 0.16734847 | -0.848, 2.116 | 57.14 | 0.36 | 0.0967 |
| haloperidol | acute | nr2b ptyr1472 | dorsal striatum | protein levels | 2 | 10 | 10 | 0.611 | -0.784, 2.006 | 0.39087515 | -1.422, 2.644 | 56.07 | 0.57 | 0.1314 |
| haloperidol | acute | psd95 | cingulate cortex | mrna levels | 2 | 28 | 14 | -0.027 | -2.344, 2.29 | 0.98171794 | -3.906, 3.852 | 90.1 | 2.52 | 0.0015 |
| haloperidol | acute | psd95 | dorsal striatum | mrna levels | 3 | 33 | 19 | 0.388 | -0.802, 1.578 | 0.52284253 | -1.725, 2.5 | 72.12 | 0.79 | 0.0255 |
| haloperidol | acute | psd95 | limbic system | mrna levels | 2 | 28 | 14 | -0.155 | -0.818, 0.509 | 0.64800273 | -0.818, 0.509 | 0 | 0 | 0.898 |
| haloperidol | acute | psd95 | sensory-motor areas | mrna levels | 2 | 28 | 14 | 0.044 | -1.827, 1.914 | 0.96354758 | -3.037, 3.124 | 85.61 | 1.56 | 0.0084 |
| haloperidol | acute | psd95 | ventral striatum | mrna levels | 3 | 33 | 19 | -0.221 | -1.842, 1.399 | 0.78885952 | -3.254, 2.811 | 83.82 | 1.71 | 0.0037 |
| haloperidol | chronic | ania3 | associative cortex | mrna levels | 2 | 12 | 12 | -0.453 | -1.266, 0.36 | 0.275061 | -1.266, 0.36 | 0 | 0 | 0.5593 |
| haloperidol | chronic | ania3 | cingulate cortex | mrna levels | 2 | 11 | 11 | 0.825 | -0.053, 1.704 | 0.065472 | -0.053, 1.704 | 0 | 0 | 0.3797 |
| haloperidol | chronic | ania3 | dorsal striatum | mrna levels | 3 | 16 | 16 | 2.942 | 1.934, 3.951 | 1.0805E-08 | 1.934, 3.951 | 0 | 0 | 0.5769 |
| haloperidol | chronic | ania3 | frontal cortex | mrna levels | 2 | 12 | 12 | -0.59 | -1.41, 0.23 | 0.1586649 | -1.41, 0.23 | 0 | 0 | 0.5667 |
| haloperidol | chronic | ania3 | ventral striatum | mrna levels | 2 | 11 | 11 | 1.124 | 0.225, 2.024 | 0.01428023 | 0.225, 2.024 | 0 | 0 | 0.9443 |
| haloperidol | chronic | arc | cingulate cortex | mrna levels | 3 | 27 | 17 | -0.706 | -1.892, 0.48 | 0.24339115 | -2.782, 1.37 | 68.75 | 0.76 | 0.0413 |
| haloperidol | chronic | arc | dorsal striatum | mrna levels | 4 | 41 | 31 | 0.636 | -0.88, 2.151 | 0.41107803 | -2.551, 3.823 | 86.73 | 2.05 | 0 |
| haloperidol | chronic | arc | limbic system | mrna levels | 3 | 27 | 17 | -0.62 | -1.697, 0.457 | 0.2589732 | -2.449, 1.209 | 62.88 | 0.57 | 0.0679 |
| haloperidol | chronic | arc | sensory-motor areas | mrna levels | 3 | 27 | 17 | -1.186 | -2.82, 0.448 | 0.15500049 | -4.22, 1.848 | 81.71 | 1.7 | 0.0065 |
| haloperidol | chronic | arc | ventral striatum | mrna levels | 3 | 27 | 17 | 0.451 | -0.185, 1.087 | 0.16483476 | -0.185, 1.087 | 0 | 0 | 0.8622 |
| haloperidol | chronic | glua1 | dorsal striatum | mrna levels | 3 | 19 | 19 | 0.653 | -0.582, 1.888 | 0.30028948 | -1.497, 2.803 | 68.38 | 0.81 | 0.0599 |
| haloperidol | chronic | glua1 | frontal cortex | mrna levels | 2 | 13 | 13 | 0.03 | -0.74, 0.799 | 0.93986102 | -0.74, 0.799 | 0 | 0 | 0.7232 |
| haloperidol | chronic | glua1 | ventral striatum | mrna levels | 2 | 13 | 13 | 1.476 | -1.117, 4.069 | 0.26466368 | -2.79, 5.742 | 84.74 | 2.99 | 0.0105 |
| haloperidol | chronic | glua2 | dorsal striatum | mrna levels | 3 | 19 | 19 | 1.198 | -1.442, 3.838 | 0.37376296 | -3.864, 6.261 | 91.54 | 4.86 | 0.0012 |
| haloperidol | chronic | glua2 | frontal cortex | mrna levels | 3 | 19 | 19 | -0.401 | -1.182, 0.38 | 0.31473858 | -1.471, 0.67 | 29.24 | 0.14 | 0.2514 |
| haloperidol | chronic | glua2 | ventral striatum | mrna levels | 3 | 19 | 19 | 0.455 | -1.658, 2.568 | 0.67281193 | -3.567, 4.477 | 87.95 | 3.05 | 0.0003 |
| haloperidol | chronic | homer1a | associative cortex | mrna levels | 2 | 12 | 12 | -0.916 | -1.758, -0.073 | 0.03312873 | -1.758, -0.073 | 0 | 0 | 0.6848 |
| haloperidol | chronic | homer1a | cingulate cortex | mrna levels | 8 | 74 | 54 | 0.144 | -0.824, 1.113 | 0.76996713 | -2.491, 2.78 | 82.87 | 1.56 | 0 |
| haloperidol | chronic | homer1a | dorsal striatum | mrna levels | 9 | 79 | 59 | 1.635 | 1.05, 2.22 | 4.2298E-08 | 0.297, 2.973 | 48.79 | 0.38 | 0.0524 |
| haloperidol | chronic | homer1a | frontal cortex | mrna levels | 2 | 12 | 12 | -0.74 | -1.568, 0.088 | 0.07992892 | -1.568, 0.088 | 0 | 0 | 0.7272 |
| haloperidol | chronic | homer1a | limbic system | mrna levels | 7 | 67 | 47 | 0.134 | -0.886, 1.155 | 0.79647682 | -2.513, 2.781 | 82.51 | 1.55 | 0 |
| haloperidol | chronic | homer1a | sensory-motor areas | mrna levels | 7 | 67 | 47 | 0.263 | -0.956, 1.482 | 0.67253154 | -2.949, 3.475 | 87.01 | 2.3 | 0 |
| haloperidol | chronic | homer1a | ventral striatum | mrna levels | 8 | 74 | 54 | 1.089 | 0.681, 1.497 | 1.7065E-07 | 0.57, 1.608 | 7.67 | 0.03 | 0.5 |
| haloperidol | chronic | homer1b-c | cingulate cortex | mrna levels | 5 | 45 | 35 | 0.284 | -0.906, 1.473 | 0.64008612 | -2.334, 2.902 | 81.61 | 1.42 | 0.0123 |
| haloperidol | chronic | homer1b-c | dorsal striatum | mrna levels | 5 | 45 | 35 | 0.373 | -0.402, 1.148 | 0.34515992 | -1.156, 1.903 | 59.66 | 0.45 | 0.0409 |
| haloperidol | chronic | homer1b-c | limbic system | mrna levels | 5 | 45 | 35 | 0.702 | -0.219, 1.623 | 0.13538141 | -1.225, 2.628 | 69.72 | 0.75 | 0.01 |
| haloperidol | chronic | homer1b-c | sensory-motor areas | mrna levels | 5 | 45 | 35 | 0.122 | -0.49, 0.734 | 0.69527778 | -0.897, 1.141 | 36.14 | 0.17 | 0.0699 |
| haloperidol | chronic | homer1b-c | ventral striatum | mrna levels | 5 | 45 | 35 | 0.708 | -0.085, 1.5 | 0.08011383 | -0.867, 2.283 | 60.32 | 0.48 | 0.0318 |
| haloperidol | chronic | mglur1 | frontal cortex | mrna levels | 2 | 10 | 10 | 0.083 | -0.796, 0.962 | 0.85313923 | -0.796, 0.962 | 0 | 0 | 0.6545 |
| haloperidol | chronic | mglur2 | frontal cortex | mrna levels | 3 | 15 | 15 | -0.289 | -1.013, 0.436 | 0.43491399 | -1.013, 0.436 | 0 | 0 | 0.6678 |
| haloperidol | chronic | mglur5 | frontal cortex | mrna levels | 3 | 15 | 15 | 0.031 | -0.692, 0.754 | 0.93287782 | -0.692, 0.754 | 0 | 0 | 0.5543 |
| haloperidol | chronic | mglur5 | frontal cortex | protein levels | 2 | 12 | 12 | 0.103 | -0.698, 0.904 | 0.80090662 | -0.698, 0.904 | 0 | 0 | 0.7728 |
| haloperidol | chronic | norbin | frontal cortex | protein levels | 2 | 12 | 12 | -0.469 | -2.552, 1.614 | 0.65892406 | -3.863, 2.925 | 82.72 | 1.87 | 0.0161 |
| haloperidol | chronic | nr1 | dorsal striatum | mrna levels | 5 | 30 | 30 | 1.586 | 0.487, 2.684 | 0.00466178 | -0.708, 3.879 | 68.57 | 1.06 | 0.0153 |
| haloperidol | chronic | nr1 | dorsal striatum | protein levels | 3 | 23 | 23 | 0.666 | -0.379, 1.711 | 0.2115891 | -1.113, 2.445 | 63.49 | 0.54 | 0.0577 |
| haloperidol | chronic | nr1 | frontal cortex | mrna levels | 4 | 32 | 32 | 0.205 | -0.289, 0.7 | 0.4152762 | -0.289, 0.7 | 0 | 0 | 0.6654 |
| haloperidol | chronic | nr1 | frontal cortex | protein levels | 4 | 29 | 29 | -0.142 | -0.983, 0.7 | 0.74175033 | -1.661, 1.378 | 57.09 | 0.42 | 0.0795 |
| haloperidol | chronic | nr1 | hippocampus | mrna levels | 2 | 16 | 16 | 0.477 | -0.653, 1.606 | 0.40797822 | -1.187, 2.141 | 58.14 | 0.39 | 0.1222 |
| haloperidol | chronic | nr1 | hippocampus | protein levels | 4 | 29 | 29 | -0.264 | -0.786, 0.259 | 0.32257201 | -0.786, 0.259 | 0 | 0 | 0.4909 |
| haloperidol | chronic | nr1 | ventral striatum | mrna levels | 4 | 25 | 25 | 0.782 | -0.364, 1.927 | 0.18108743 | -1.433, 2.996 | 70.19 | 0.93 | 0.0245 |
| haloperidol | chronic | nr1 | ventral striatum | protein levels | 3 | 23 | 23 | 0.357 | -0.642, 1.357 | 0.48376684 | -1.315, 2.029 | 60.1 | 0.47 | 0.084 |
| haloperidol | chronic | nr2a | dorsal striatum | mrna levels | 2 | 15 | 15 | 0.148 | -2.97, 3.267 | 0.92566932 | -5.113, 5.41 | 92.28 | 4.67 | 0.0003 |
| haloperidol | chronic | nr2a | dorsal striatum | protein levels | 2 | 11 | 11 | 0.42 | -0.425, 1.265 | 0.32985018 | -0.425, 1.265 | 0 | 0 | 0.899 |
| haloperidol | chronic | nr2a | frontal cortex | mrna levels | 2 | 21 | 21 | -0.544 | -1.16, 0.072 | 0.08333199 | -1.16, 0.072 | 0 | 0 | 0.8752 |
| haloperidol | chronic | nr2a | frontal cortex | protein levels | 3 | 17 | 17 | -0.95 | -1.828, -0.072 | 0.03386986 | -2.176, 0.275 | 31.61 | 0.19 | 0.2228 |
| haloperidol | chronic | nr2a | hippocampus | mrna levels | 2 | 21 | 21 | -0.443 | -1.056, 0.169 | 0.15594529 | -1.056, 0.169 | 0 | 0 | 0.8803 |
| haloperidol | chronic | nr2a | hippocampus | protein levels | 3 | 17 | 17 | -0.551 | -1.33, 0.228 | 0.16574759 | -1.527, 0.426 | 18.89 | 0.09 | 0.2559 |
| haloperidol | chronic | nr2a | ventral striatum | protein levels | 2 | 11 | 11 | 0.086 | -0.755, 0.927 | 0.84077202 | -0.755, 0.927 | 0 | 0 | 0.4828 |
| haloperidol | chronic | nr2b | dorsal striatum | mrna levels | 2 | 15 | 15 | 1.346 | -0.218, 2.909 | 0.09155766 | -1.093, 3.785 | 71.56 | 0.91 | 0.0608 |
| haloperidol | chronic | nr2b | frontal cortex | protein levels | 2 | 11 | 11 | 0.35 | -0.936, 1.635 | 0.5939771 | -1.508, 2.207 | 54.2 | 0.47 | 0.1395 |
| haloperidol | chronic | nr2b | hippocampus | protein levels | 3 | 17 | 17 | -1.031 | -2.132, 0.069 | 0.06625326 | -2.817, 0.754 | 54.49 | 0.51 | 0.1101 |
| haloperidol | chronic | preso1 | frontal cortex | protein levels | 2 | 12 | 12 | 0.041 | -0.76, 0.842 | 0.91960294 | -0.76, 0.842 | 0 | 0 | 0.73 |
| haloperidol | chronic | psd95 | cingulate cortex | mrna levels | 5 | 45 | 35 | 0.251 | -1.452, 1.954 | 0.7726198 | -3.719, 4.221 | 90.03 | 3.35 | 0 |
| haloperidol | chronic | psd95 | dorsal striatum | mrna levels | 5 | 45 | 35 | 0.351 | -0.821, 1.524 | 0.55668069 | -2.261, 2.964 | 81.2 | 1.42 | 0.0002 |
| haloperidol | chronic | psd95 | frontal cortex | protein levels | 3 | 16 | 16 | -0.25 | -0.947, 0.446 | 0.48087516 | -0.947, 0.446 | 0 | 0 | 0.9381 |
| haloperidol | chronic | psd95 | limbic system | mrna levels | 5 | 45 | 35 | -0.301 | -1.306, 0.704 | 0.55702181 | -2.478, 1.876 | 75.36 | 0.97 | 0.0016 |
| haloperidol | chronic | psd95 | sensory-motor areas | mrna levels | 5 | 45 | 35 | 0.09 | -1.156, 1.337 | 0.88692017 | -2.723, 2.904 | 83.33 | 1.66 | 0.0001 |
| haloperidol | chronic | psd95 | ventral striatum | mrna levels | 5 | 45 | 35 | 0.162 | -0.739, 1.064 | 0.72411068 | -1.746, 2.071 | 70.57 | 0.74 | 0.0037 |
| haloperidol | chronic | tamalin | frontal cortex | protein levels | 2 | 12 | 12 | 0.135 | -0.666, 0.936 | 0.74079611 | -0.666, 0.936 | 0 | 0 | 0.9777 |
| haloperidol decanoate | chronic | glua2 | dorsal striatum | mrna levels | 2 | 13 | 13 | 0.973 | -0.106, 2.052 | 0.07706322 | -0.482, 2.429 | 40.37 | 0.25 | 0.1953 |
| olanzapine | acute | arc | dorsal striatum | mrna levels | 3 | 48 | 48 | 1.41 | 0.374, 2.446 | 0.00766289 | -0.482, 3.302 | 78.54 | 0.65 | 0.0162 |
| olanzapine | acute | arc | frontal cortex | mrna levels | 2 | 41 | 41 | -2.154 | -2.699, -1.61 | 8.71E-15 | -2.699, -1.61 | 0 | 0 | 0.737 |
| olanzapine | acute | arc | ventral striatum | mrna levels | 2 | 27 | 27 | 1.486 | 0.882, 2.091 | 1.4558E-06 | 0.882, 2.091 | 0 | 0 | 0.387 |
| olanzapine | acute | homer1a | cingulate cortex | mrna levels | 2 | 12 | 12 | 0.701 | -0.967, 2.368 | 0.41011729 | -1.918, 3.32 | 73.34 | 1.06 | 0.0528 |
| olanzapine | acute | homer1a | dorsal striatum | mrna levels | 4 | 22 | 22 | 3.442 | -1.378, 8.261 | 0.16162121 | -6.974, 13.857 | 97.2 | 22.19 | 0.0001 |
| olanzapine | acute | homer1a | limbic system | mrna levels | 2 | 12 | 12 | 0.947 | -0.16, 2.053 | 0.09368945 | -0.535, 2.428 | 39.54 | 0.25 | 0.1984 |
| olanzapine | acute | homer1a | sensory-motor areas | mrna levels | 2 | 12 | 12 | 0.641 | -0.674, 1.957 | 0.33920261 | -1.298, 2.581 | 58.66 | 0.53 | 0.1199 |
| olanzapine | acute | homer1a | ventral striatum | mrna levels | 4 | 22 | 22 | 1.845 | 0.27, 3.419 | 0.02164256 | -1.318, 5.007 | 77.74 | 1.96 | 0.0086 |
| olanzapine | acute | psd95 | dorsal striatum | mrna levels | 2 | 12 | 12 | 2.066 | -0.591, 4.723 | 0.12756396 | -2.294, 6.426 | 84.44 | 3.11 | 0.0113 |
| olanzapine | acute | psd95 | frontal cortex | protein levels | 6 | 36 | 36 | 2.827 | -0.45, 6.105 | 0.09088374 | -5.553, 11.208 | 96.66 | 15.49 | 0 |
| olanzapine | acute | psd95 | hippocampus | protein levels | 6 | 36 | 36 | -0.136 | -0.619, 0.348 | 0.58238693 | -0.685, 0.414 | 4.85 | 0.02 | 0.3512 |
| olanzapine | acute | psd95 | ventral striatum | mrna levels | 2 | 12 | 12 | 1.438 | -1.025, 3.9 | 0.25247242 | -2.608, 5.483 | 84.92 | 2.68 | 0.01 |
| olanzapine | chronic | arc | dorsal striatum | mrna levels | 2 | 19 | 19 | -1.597 | -2.328, -0.867 | 1.8322E-05 | -2.328, -0.867 | 0 | 0 | 0.7685 |
| olanzapine | chronic | homer1a | cingulate cortex | mrna levels | 2 | 10 | 10 | 0.2 | -0.686, 1.085 | 0.65825126 | -0.686, 1.085 | 0 | 0 | 0.4347 |
| olanzapine | chronic | homer1a | dorsal striatum | mrna levels | 2 | 10 | 10 | 0.342 | -0.631, 1.314 | 0.49105315 | -0.767, 1.45 | 15 | 0.07 | 0.2781 |
| olanzapine | chronic | homer1a | limbic system | mrna levels | 2 | 10 | 10 | -0.245 | -1.127, 0.636 | 0.58547176 | -1.127, 0.636 | 0 | 0 | 0.6992 |
| olanzapine | chronic | homer1a | sensory-motor areas | mrna levels | 2 | 10 | 10 | 0.322 | -0.789, 1.432 | 0.57038596 | -1.121, 1.764 | 34.37 | 0.22 | 0.2171 |
| olanzapine | chronic | homer1a | ventral striatum | mrna levels | 2 | 10 | 10 | 1.185 | -1.506, 3.875 | 0.38814612 | -3.237, 5.606 | 84.85 | 3.2 | 0.0102 |
| olanzapine | chronic | mglur5 | frontal cortex | protein levels | 2 | 12 | 12 | 0.222 | -0.581, 1.025 | 0.58811116 | -0.581, 1.025 | 0 | 0 | 0.7776 |
| olanzapine | chronic | norbin | frontal cortex | protein levels | 2 | 12 | 12 | -0.039 | -0.84, 0.763 | 0.92462699 | -0.84, 0.763 | 0 | 0 | 0.6702 |
| olanzapine | chronic | nr1 | frontal cortex | protein levels | 2 | 12 | 12 | 0.461 | -1.201, 2.124 | 0.58652779 | -2.158, 3.081 | 74.09 | 1.07 | 0.0495 |
| olanzapine | chronic | nr1 | hippocampus | mrna levels | 2 | 11 | 11 | -0.403 | -2.188, 1.381 | 0.65770231 | -3.225, 2.418 | 74.89 | 1.24 | 0.046 |
| olanzapine | chronic | nr1 | hippocampus | protein levels | 3 | 18 | 18 | -0.292 | -1.01, 0.426 | 0.42533578 | -1.143, 0.559 | 13.47 | 0.05 | 0.3056 |
| olanzapine | chronic | nr2a | frontal cortex | protein levels | 2 | 12 | 12 | -0.064 | -1.2, 1.071 | 0.91150552 | -1.656, 1.528 | 48.25 | 0.32 | 0.1645 |
| olanzapine | chronic | nr2a | hippocampus | mrna levels | 2 | 11 | 11 | 0.247 | -0.599, 1.093 | 0.56745235 | -0.599, 1.093 | 0 | 0 | 0.3813 |
| olanzapine | chronic | nr2a | hippocampus | protein levels | 3 | 18 | 18 | -0.089 | -0.749, 0.57 | 0.7907244 | -0.749, 0.57 | 0 | 0 | 0.5197 |
| olanzapine | chronic | nr2b | hippocampus | mrna levels | 2 | 11 | 11 | 0.659 | -0.203, 1.52 | 0.13399263 | -0.203, 1.52 | 0 | 0 | 0.5523 |
| olanzapine | chronic | nr2b | hippocampus | protein levels | 3 | 18 | 18 | -0.278 | -1.261, 0.704 | 0.57862304 | -1.856, 1.299 | 52.55 | 0.4 | 0.122 |
| olanzapine | chronic | preso1 | frontal cortex | protein levels | 2 | 12 | 12 | 0.065 | -0.736, 0.865 | 0.87449228 | -0.736, 0.865 | 0 | 0 | 0.8204 |
| olanzapine | chronic | psd95 | frontal cortex | protein levels | 3 | 16 | 16 | -0.095 | -1.294, 1.103 | 0.87593782 | -2.136, 1.945 | 63.3 | 0.71 | 0.0667 |
| olanzapine | chronic | tamalin | frontal cortex | protein levels | 2 | 12 | 12 | 0.399 | -0.413, 1.212 | 0.33558894 | -0.413, 1.212 | 0 | 0 | 0.4663 |
| quetiapine | acute | homer1a | dorsal striatum | mrna levels | 2 | 24 | 12 | 0.436 | -0.264, 1.136 | 0.22239809 | -0.264, 1.136 | 0 | 0 | 0.9673 |
| quetiapine | chronic | homer1a | dorsal striatum | mrna levels | 2 | 19 | 12 | 0.648 | -0.822, 2.119 | 0.38764893 | -1.624, 2.921 | 68.25 | 0.78 | 0.076 |

## Sensitivity analyses based on animal models of psychosis

| **drug** | **model** | **psd type** | **region** | **outcome type** | **n_studies** | **n_group_1** | **n_group_2** | **beta** | **CI** | **p_val** | **PI** | **i_sq** | **tau_sq** | **q_p_val** |
| --- | --- | --- | --- | --- | --- | --- | --- | --- | --- | --- | --- | --- | --- | --- |
| aripiprazole | acute | arc | dorsal striatum | mrna levels | 2 | 35 | 25 | 1.006 | -2.546, 4.558 | 0.5788137 | -5.071, 7.083 | 96.33 | 6.33 | 0 |
| aripiprazole | acute | arc | frontal cortex | mrna levels | 2 | 35 | 25 | -2.268 | -4.586, 0.051 | 0.05521776 | -6.151, 1.616 | 90.29 | 2.53 | 0.0013 |
| aripiprazole | chronic | homer1a | ventral striatum | protein levels | 2 | 12 | 12 | 2.574 | 0.261, 4.886 | 0.02914093 | -1.097, 6.245 | 75.35 | 2.12 | 0.044 |
| aripiprazole | chronic | homer1b-c | ventral striatum | protein levels | 2 | 12 | 12 | -0.623 | -3.312, 2.065 | 0.6495305 | -5.101, 3.854 | 88.6 | 3.34 | 0.0031 |
| aripiprazole | chronic | mglur1 | ventral striatum | protein levels | 2 | 12 | 12 | -0.371 | -1.178, 0.436 | 0.36756978 | -1.178, 0.436 | 0 | 0 | 0.9855 |
| aripiprazole | chronic | mglur5 | ventral striatum | protein levels | 2 | 12 | 12 | 0.12 | -1.864, 2.104 | 0.90559419 | -3.1, 3.34 | 81.63 | 1.67 | 0.0197 |
| aripiprazole | chronic | norbin | ventral striatum | protein levels | 2 | 12 | 12 | 1.725 | 0.782, 2.667 | 0.00033353 | 0.782, 2.667 | 0 | 0 | 0.4768 |
| aripiprazole | chronic | nr1 | frontal cortex | protein levels | 2 | 12 | 12 | -0.002 | -0.802, 0.798 | 0.99583721 | -0.802, 0.798 | 0 | 0 | 0.9958 |
| aripiprazole | chronic | nr1 | ventral striatum | protein levels | 2 | 12 | 12 | 0.321 | -1.858, 2.501 | 0.77254555 | -3.25, 3.892 | 84.25 | 2.08 | 0.0117 |
| asenapine | acute | homer1a | cingulate cortex | mrna levels | 3 | 41 | 17 | 0.526 | -0.168, 1.22 | 0.13730916 | -0.4, 1.452 | 25.69 | 0.1 | 0.2855 |
| asenapine | acute | homer1a | dorsal striatum | mrna levels | 3 | 41 | 17 | 1.14 | 0.522, 1.758 | 0.0003011 | 0.522, 1.758 | 0 | 0 | 0.4428 |
| asenapine | acute | homer1a | limbic system | mrna levels | 3 | 41 | 17 | 0.546 | -0.042, 1.133 | 0.06870066 | -0.042, 1.133 | 0 | 0 | 0.8186 |
| asenapine | acute | homer1a | sensory-motor areas | mrna levels | 3 | 41 | 17 | 0.558 | -0.032, 1.148 | 0.06378405 | -0.032, 1.148 | 0 | 0 | 0.527 |
| asenapine | acute | homer1a | ventral striatum | mrna levels | 3 | 41 | 17 | 0.858 | 0.073, 1.643 | 0.03219248 | -0.298, 2.014 | 38.78 | 0.19 | 0.2052 |
| asenapine | chronic | homer1a | cingulate cortex | mrna levels | 2 | 30 | 10 | -0.064 | -0.78, 0.652 | 0.86107453 | -0.78, 0.652 | 0 | 0 | 0.861 |
| asenapine | chronic | homer1a | dorsal striatum | mrna levels | 2 | 30 | 10 | 0.137 | -0.782, 1.057 | 0.76951282 | -1.083, 1.358 | 38.08 | 0.17 | 0.2038 |
| asenapine | chronic | homer1a | limbic system | mrna levels | 2 | 30 | 10 | -1.231 | -2.076, -0.385 | 0.0043296 | -2.208, -0.253 | 16.8 | 0.06 | 0.2729 |
| asenapine | chronic | homer1a | sensory-motor areas | mrna levels | 2 | 30 | 10 | -0.06 | -0.776, 0.657 | 0.86965689 | -0.776, 0.657 | 0 | 0 | 0.6934 |
| asenapine | chronic | homer1a | ventral striatum | mrna levels | 2 | 30 | 10 | 0.431 | -0.458, 1.32 | 0.34183304 | -0.713, 1.576 | 32.81 | 0.14 | 0.2225 |
| clozapine | acute | arc | dorsal striatum | mrna levels | 3 | 32 | 32 | -0.135 | -0.703, 0.434 | 0.64262337 | -0.843, 0.574 | 16.22 | 0.05 | 0.3683 |
| clozapine | acute | arc | frontal cortex | mrna levels | 3 | 32 | 32 | -0.914 | -2.08, 0.252 | 0.12460827 | -3.011, 1.183 | 75.01 | 0.79 | 0.0149 |
| clozapine | acute | arc | ventral striatum | mrna levels | 2 | 25 | 25 | -0.019 | -1.872, 1.834 | 0.98390548 | -3.065, 3.027 | 84.34 | 1.52 | 0.0115 |
| clozapine | acute | homer1a | cingulate cortex | mrna levels | 2 | 12 | 12 | 2.119 | 1.119, 3.119 | 3.2667E-05 | 1.119, 3.119 | 0 | 0 | 0.9358 |
| clozapine | acute | homer1a | dorsal striatum | mrna levels | 3 | 17 | 17 | 1.131 | 0.406, 1.856 | 0.00223067 | 0.406, 1.856 | 0 | 0 | 0.9033 |
| clozapine | acute | homer1a | ventral striatum | mrna levels | 3 | 17 | 17 | 1.478 | 0.716, 2.239 | 0.00014279 | 0.716, 2.239 | 0 | 0 | 0.7652 |
| clozapine | acute | nr1 | frontal cortex | mrna levels | 2 | 11 | 11 | 1.085 | 0.185, 1.984 | 0.01808607 | 0.185, 1.984 | 0 | 0 | 0.5107 |
| clozapine | acute | nr1 | hippocampus | mrna levels | 2 | 11 | 11 | -0.417 | -1.435, 0.602 | 0.42270928 | -1.686, 0.852 | 26.82 | 0.15 | 0.2424 |
| clozapine | acute | nr2a | frontal cortex | mrna levels | 2 | 11 | 11 | 0.89 | -0.029, 1.809 | 0.05778657 | -0.088, 1.867 | 6.35 | 0.03 | 0.3014 |
| clozapine | acute | nr2a | hippocampus | mrna levels | 2 | 11 | 11 | 0.337 | -0.504, 1.179 | 0.43217977 | -0.504, 1.179 | 0 | 0 | 0.9318 |
| clozapine | acute | nr2b | frontal cortex | mrna levels | 2 | 11 | 11 | 0.631 | -0.228, 1.49 | 0.14982678 | -0.228, 1.49 | 0 | 0 | 0.6033 |
| clozapine | acute | nr2b | hippocampus | mrna levels | 2 | 11 | 11 | 0.104 | -0.734, 0.941 | 0.80787175 | -0.734, 0.941 | 0 | 0 | 0.7161 |
| clozapine | chronic | mglur2 | frontal cortex | mrna levels | 2 | 15 | 10 | 0.266 | -0.973, 1.504 | 0.67426526 | -1.52, 2.051 | 53.47 | 0.43 | 0.1426 |
| clozapine | chronic | mglur5 | frontal cortex | mrna levels | 2 | 15 | 10 | 0.097 | -0.715, 0.909 | 0.81540844 | -0.715, 0.909 | 0 | 0 | 0.998 |
| clozapine | chronic | nr1 | dorsal striatum | mrna levels | 3 | 22 | 22 | 1.424 | -0.833, 3.682 | 0.21623755 | -2.86, 5.708 | 89.75 | 3.45 | 0.0029 |
| clozapine | chronic | nr1 | dorsal striatum | protein levels | 2 | 17 | 17 | 0.761 | 0.065, 1.457 | 0.03219395 | 0.065, 1.457 | 0 | 0 | 0.9034 |
| clozapine | chronic | nr1 | frontal cortex | mrna levels | 3 | 28 | 28 | -0.097 | -0.75, 0.557 | 0.77164787 | -1.018, 0.824 | 32.71 | 0.11 | 0.2061 |
| clozapine | chronic | nr1 | frontal cortex | protein levels | 2 | 17 | 17 | -0.566 | -1.255, 0.123 | 0.10753823 | -1.255, 0.123 | 0 | 0 | 0.4038 |
| clozapine | chronic | nr1 | hippocampus | mrna levels | 2 | 17 | 17 | 0.362 | -0.316, 1.039 | 0.29560784 | -0.316, 1.039 | 0 | 0 | 0.9952 |
| clozapine | chronic | nr1 | hippocampus | protein levels | 3 | 23 | 23 | -0.008 | -0.592, 0.575 | 0.97754408 | -0.592, 0.575 | 0 | 0 | 0.4264 |
| clozapine | chronic | nr1 | ventral striatum | protein levels | 2 | 17 | 17 | 0.076 | -0.736, 0.888 | 0.85489505 | -0.929, 1.08 | 23.83 | 0.09 | 0.2519 |
| clozapine | chronic | nr2a | dorsal striatum | mrna levels | 2 | 15 | 15 | 0.445 | -0.719, 1.609 | 0.45363294 | -1.26, 2.15 | 56.34 | 0.4 | 0.1302 |
| clozapine | chronic | nr2a | frontal cortex | mrna levels | 2 | 21 | 21 | -0.565 | -1.182, 0.052 | 0.07251751 | -1.182, 0.052 | 0 | 0 | 0.9514 |
| clozapine | chronic | nr2a | hippocampus | mrna levels | 2 | 21 | 21 | -0.344 | -0.953, 0.266 | 0.26964694 | -0.953, 0.266 | 0 | 0 | 0.685 |
| clozapine | chronic | nr2a | hippocampus | protein levels | 2 | 11 | 11 | -0.29 | -1.137, 0.556 | 0.50104712 | -1.137, 0.556 | 0 | 0 | 0.4319 |
| clozapine | chronic | nr2b | dorsal striatum | mrna levels | 2 | 15 | 15 | 0.895 | -0.007, 1.797 | 0.0517365 | -0.218, 2.008 | 23.35 | 0.11 | 0.2534 |
| clozapine | chronic | nr2b | hippocampus | protein levels | 2 | 11 | 11 | -0.773 | -2.516, 0.969 | 0.38441126 | -3.501, 1.955 | 72.22 | 1.15 | 0.0578 |
| flupenthixol | chronic | nr1 | associative cortex | mrna levels | 2 | 16 | 16 | -1.687 | -2.495, -0.88 | 4.1643E-05 | -2.495, -0.88 | 0 | 0 | 0.8821 |
| flupenthixol | chronic | nr1 | cerebellum | mrna levels | 2 | 16 | 16 | -1.441 | -2.22, -0.662 | 0.0002901 | -2.22, -0.662 | 0 | 0 | 0.6117 |
| flupenthixol | chronic | nr1 | frontal cortex | mrna levels | 2 | 16 | 16 | -1.505 | -2.367, -0.643 | 0.00062435 | -2.492, -0.518 | 15.35 | 0.06 | 0.2771 |
| flupenthixol | chronic | nr1 | subcortical areas | mrna levels | 2 | 16 | 16 | -1.338 | -2.139, -0.538 | 0.00105306 | -2.191, -0.485 | 6.69 | 0.02 | 0.3006 |
| flupenthixol | chronic | nr2b | associative cortex | mrna levels | 2 | 16 | 16 | -0.116 | -1.377, 1.144 | 0.8566824 | -2.054, 1.821 | 68.16 | 0.56 | 0.0764 |
| flupenthixol | chronic | nr2b | cerebellum | mrna levels | 2 | 16 | 16 | 0.593 | -0.12, 1.305 | 0.10316132 | -0.12, 1.305 | 0 | 0 | 0.3593 |
| flupenthixol | chronic | nr2b | frontal cortex | mrna levels | 2 | 16 | 16 | -0.063 | -0.76, 0.634 | 0.85994183 | -0.76, 0.634 | 0 | 0 | 0.3978 |
| flupenthixol | chronic | nr2b | subcortical areas | mrna levels | 2 | 16 | 16 | 0.076 | -1.266, 1.417 | 0.91200128 | -2.017, 2.169 | 71.73 | 0.67 | 0.06 |
| flupenthixol | chronic | nr2c | associative cortex | mrna levels | 2 | 16 | 16 | -0.067 | -0.762, 0.628 | 0.84984417 | -0.762, 0.628 | 0 | 0 | 0.5402 |
| flupenthixol | chronic | nr2c | cerebellum | mrna levels | 2 | 16 | 16 | -0.047 | -1.612, 1.519 | 0.95318765 | -2.56, 2.466 | 78.84 | 1.01 | 0.0297 |
| flupenthixol | chronic | nr2c | frontal cortex | mrna levels | 2 | 16 | 16 | -0.305 | -1.671, 1.061 | 0.66166713 | -2.441, 1.832 | 72.38 | 0.7 | 0.0571 |
| flupenthixol | chronic | nr2c | subcortical areas | mrna levels | 2 | 16 | 16 | 0.779 | 0.056, 1.503 | 0.03474787 | 0.056, 1.503 | 0 | 0 | 0.3628 |
| flupenthixol | chronic | nr2d | associative cortex | mrna levels | 2 | 16 | 16 | -1.595 | -4.331, 1.142 | 0.25337054 | -6.173, 2.984 | 89.86 | 3.51 | 0.0017 |
| flupenthixol | chronic | nr2d | cerebellum | mrna levels | 2 | 16 | 16 | 0.899 | 0.041, 1.757 | 0.0399452 | -0.163, 1.961 | 26.48 | 0.1 | 0.2435 |
| flupenthixol | chronic | nr2d | frontal cortex | mrna levels | 2 | 16 | 16 | -0.019 | -0.713, 0.675 | 0.95734427 | -0.713, 0.675 | 0 | 0 | 0.6725 |
| flupenthixol | chronic | nr2d | subcortical areas | mrna levels | 2 | 16 | 16 | 0.669 | -0.1, 1.438 | 0.08834587 | -0.193, 1.531 | 12.74 | 0.04 | 0.2844 |
| haloperidol | acute | ania3 | associative cortex | mrna levels | 3 | 17 | 17 | 0.018 | -0.977, 1.013 | 0.97178106 | -1.565, 1.601 | 51.03 | 0.39 | 0.1294 |
| haloperidol | acute | ania3 | cingulate cortex | mrna levels | 4 | 23 | 23 | 0.997 | -0.261, 2.255 | 0.12030827 | -1.478, 3.472 | 72.81 | 1.18 | 0.0125 |
| haloperidol | acute | ania3 | dorsal striatum | mrna levels | 5 | 28 | 29 | 2.471 | 1.076, 3.866 | 0.00051852 | -0.521, 5.463 | 73.64 | 1.82 | 0.0053 |
| haloperidol | acute | ania3 | frontal cortex | mrna levels | 3 | 17 | 17 | -0.136 | -0.817, 0.545 | 0.69530376 | -0.817, 0.545 | 0 | 0 | 0.4424 |
| haloperidol | acute | ania3 | limbic system | mrna levels | 2 | 11 | 11 | 0.615 | -0.241, 1.471 | 0.15884517 | -0.241, 1.471 | 0 | 0 | 0.8625 |
| haloperidol | acute | ania3 | sensory-motor areas | mrna levels | 2 | 11 | 11 | 0.384 | -0.901, 1.67 | 0.5578257 | -1.454, 2.223 | 50.8 | 0.45 | 0.154 |
| haloperidol | acute | ania3 | ventral striatum | mrna levels | 5 | 28 | 29 | 1.425 | 0.607, 2.243 | 0.00064121 | -0.056, 2.906 | 45.76 | 0.4 | 0.1156 |
| haloperidol | acute | arc | cingulate cortex | mrna levels | 4 | 39 | 25 | 0.193 | -0.968, 1.354 | 0.7440593 | -2.14, 2.526 | 76.63 | 1.07 | 0.0024 |
| haloperidol | acute | arc | dorsal striatum | mrna levels | 9 | 107 | 83 | 2.657 | 1.244, 4.069 | 0.00022803 | -1.534, 6.848 | 91.22 | 4.05 | 0 |
| haloperidol | acute | arc | frontal cortex | mrna levels | 5 | 68 | 58 | -0.949 | -1.327, -0.57 | 9.0674E-07 | -1.327, -0.57 | 0 | 0 | 0.3895 |
| haloperidol | acute | arc | limbic system | mrna levels | 4 | 39 | 25 | -0.212 | -1.479, 1.054 | 0.74234798 | -2.796, 2.372 | 79.84 | 1.32 | 0.0021 |
| haloperidol | acute | arc | sensory-motor areas | mrna levels | 4 | 39 | 25 | -0.231 | -1.217, 0.754 | 0.64566191 | -2.131, 1.669 | 68.64 | 0.69 | 0.0177 |
| haloperidol | acute | arc | ventral striatum | mrna levels | 6 | 64 | 50 | 1.811 | 1.124, 2.498 | 2.3845E-07 | 0.448, 3.174 | 50.83 | 0.36 | 0.0611 |
| haloperidol | acute | glua1 pser845 | dorsal striatum | protein levels | 3 | 60 | 24 | 3.187 | 0.9, 5.474 | 0.00630518 | -1.113, 7.487 | 90.08 | 3.45 | 0.0026 |
| haloperidol | acute | homer1a | associative cortex | mrna levels | 3 | 17 | 17 | -0.361 | -1.47, 0.749 | 0.52395279 | -2.203, 1.482 | 58.76 | 0.56 | 0.0945 |
| haloperidol | acute | homer1a | cingulate cortex | mrna levels | 10 | 91 | 67 | 0.208 | -0.243, 0.658 | 0.36649689 | -0.81, 1.225 | 42.1 | 0.22 | 0.0516 |
| haloperidol | acute | homer1a | dorsal striatum | mrna levels | 14 | 111 | 87 | 2.236 | 1.622, 2.849 | 9.1893E-13 | 0.478, 3.993 | 58.01 | 0.71 | 0 |
| haloperidol | acute | homer1a | frontal cortex | mrna levels | 3 | 17 | 17 | 0.033 | -0.647, 0.713 | 0.92422709 | -0.647, 0.713 | 0 | 0 | 0.4486 |
| haloperidol | acute | homer1a | limbic system | mrna levels | 8 | 79 | 55 | -0.527 | -1.255, 0.201 | 0.15574225 | -2.376, 1.322 | 71.43 | 0.75 | 0.003 |
| haloperidol | acute | homer1a | sensory-motor areas | mrna levels | 8 | 79 | 55 | -0.034 | -0.539, 0.472 | 0.89633796 | -1.096, 1.029 | 44.23 | 0.23 | 0.0408 |
| haloperidol | acute | homer1a | ventral striatum | mrna levels | 13 | 106 | 82 | 1.945 | 1.342, 2.548 | 2.6185E-10 | 0.2, 3.689 | 61.4 | 0.7 | 0.0009 |
| haloperidol | acute | homer1b-c | cingulate cortex | mrna levels | 2 | 28 | 14 | 0.319 | -0.965, 1.604 | 0.62609657 | -1.682, 2.32 | 71.15 | 0.61 | 0.0626 |
| haloperidol | acute | homer1b-c | dorsal striatum | mrna levels | 2 | 28 | 14 | 0.548 | -0.514, 1.61 | 0.31193467 | -1.014, 2.111 | 57.95 | 0.34 | 0.1231 |
| haloperidol | acute | homer1b-c | limbic system | mrna levels | 2 | 28 | 14 | 0.253 | -1.04, 1.545 | 0.70177393 | -1.764, 2.27 | 71.52 | 0.62 | 0.061 |
| haloperidol | acute | homer1b-c | sensory-motor areas | mrna levels | 2 | 28 | 14 | 0.833 | 0.133, 1.532 | 0.01963505 | 0.112, 1.553 | 2.97 | 0.01 | 0.31 |
| haloperidol | acute | homer1b-c | ventral striatum | mrna levels | 2 | 28 | 14 | 0.467 | -0.972, 1.906 | 0.52480593 | -1.825, 2.759 | 76.66 | 0.83 | 0.0384 |
| haloperidol | acute | nr1 | frontal cortex | mrna levels | 2 | 11 | 11 | 0.184 | -0.659, 1.027 | 0.6686164 | -0.659, 1.027 | 0 | 0 | 0.4539 |
| haloperidol | acute | nr1 | hippocampus | mrna levels | 2 | 11 | 11 | -0.111 | -0.948, 0.725 | 0.794491 | -0.948, 0.725 | 0 | 0 | 0.8946 |
| haloperidol | acute | nr2a | frontal cortex | mrna levels | 2 | 11 | 11 | 0.245 | -1.111, 1.602 | 0.72292343 | -1.746, 2.237 | 57.17 | 0.55 | 0.1265 |
| haloperidol | acute | nr2a | hippocampus | mrna levels | 2 | 11 | 11 | 0.362 | -1.763, 2.488 | 0.73838549 | -3.08, 3.805 | 81.05 | 1.91 | 0.0216 |
| haloperidol | acute | nr2b | frontal cortex | mrna levels | 2 | 11 | 11 | 0.215 | -0.631, 1.061 | 0.61891263 | -0.631, 1.061 | 0 | 0 | 0.3677 |
| haloperidol | acute | nr2b | hippocampus | mrna levels | 2 | 11 | 11 | 0.669 | -0.242, 1.58 | 0.15033063 | -0.316, 1.653 | 8.1 | 0.04 | 0.2969 |
| haloperidol | acute | psd95 | cingulate cortex | mrna levels | 2 | 28 | 14 | -0.027 | -2.344, 2.29 | 0.98171794 | -3.906, 3.852 | 90.1 | 2.52 | 0.0015 |
| haloperidol | acute | psd95 | dorsal striatum | mrna levels | 3 | 33 | 19 | 0.388 | -0.802, 1.578 | 0.52284253 | -1.725, 2.5 | 72.12 | 0.79 | 0.0255 |
| haloperidol | acute | psd95 | limbic system | mrna levels | 2 | 28 | 14 | -0.155 | -0.818, 0.509 | 0.64800273 | -0.818, 0.509 | 0 | 0 | 0.898 |
| haloperidol | acute | psd95 | sensory-motor areas | mrna levels | 2 | 28 | 14 | 0.044 | -1.827, 1.914 | 0.96354758 | -3.037, 3.124 | 85.61 | 1.56 | 0.0084 |
| haloperidol | acute | psd95 | ventral striatum | mrna levels | 3 | 33 | 19 | -0.221 | -1.842, 1.399 | 0.78885952 | -3.254, 2.811 | 83.82 | 1.71 | 0.0037 |
| haloperidol | chronic | ania3 | associative cortex | mrna levels | 2 | 12 | 12 | -0.453 | -1.266, 0.36 | 0.275061 | -1.266, 0.36 | 0 | 0 | 0.5593 |
| haloperidol | chronic | ania3 | cingulate cortex | mrna levels | 2 | 11 | 11 | 0.825 | -0.053, 1.704 | 0.065472 | -0.053, 1.704 | 0 | 0 | 0.3797 |
| haloperidol | chronic | ania3 | dorsal striatum | mrna levels | 3 | 16 | 16 | 2.942 | 1.934, 3.951 | 1.0805E-08 | 1.934, 3.951 | 0 | 0 | 0.5769 |
| haloperidol | chronic | ania3 | frontal cortex | mrna levels | 2 | 12 | 12 | -0.59 | -1.41, 0.23 | 0.1586649 | -1.41, 0.23 | 0 | 0 | 0.5667 |
| haloperidol | chronic | ania3 | ventral striatum | mrna levels | 2 | 11 | 11 | 1.124 | 0.225, 2.024 | 0.01428023 | 0.225, 2.024 | 0 | 0 | 0.9443 |
| haloperidol | chronic | arc | cingulate cortex | mrna levels | 2 | 21 | 11 | -0.851 | -2.854, 1.153 | 0.40521206 | -4.124, 2.423 | 83.51 | 1.75 | 0.0138 |
| haloperidol | chronic | arc | dorsal striatum | mrna levels | 3 | 35 | 25 | 0.213 | -1.501, 1.926 | 0.80770181 | -3.042, 3.468 | 87.79 | 1.99 | 0.0006 |
| haloperidol | chronic | arc | limbic system | mrna levels | 2 | 21 | 11 | -0.932 | -2.474, 0.609 | 0.23592979 | -3.348, 1.483 | 72.73 | 0.9 | 0.0555 |
| haloperidol | chronic | arc | sensory-motor areas | mrna levels | 2 | 21 | 11 | -1.659 | -4.09, 0.772 | 0.18101122 | -5.678, 2.36 | 86.64 | 2.67 | 0.0062 |
| haloperidol | chronic | arc | ventral striatum | mrna levels | 2 | 21 | 11 | 0.545 | -0.223, 1.313 | 0.16408633 | -0.223, 1.313 | 0 | 0 | 0.7383 |
| haloperidol | chronic | glua1 | dorsal striatum | mrna levels | 3 | 19 | 19 | 0.653 | -0.582, 1.888 | 0.30028948 | -1.497, 2.803 | 68.38 | 0.81 | 0.0599 |
| haloperidol | chronic | glua1 | frontal cortex | mrna levels | 2 | 13 | 13 | 0.03 | -0.74, 0.799 | 0.93986102 | -0.74, 0.799 | 0 | 0 | 0.7232 |
| haloperidol | chronic | glua1 | subcortical areas | protein levels | 2 | 14 | 14 | -0.246 | -0.99, 0.498 | 0.51739157 | -0.99, 0.498 | 0 | 0 | 0.7722 |
| haloperidol | chronic | glua1 | ventral striatum | mrna levels | 2 | 13 | 13 | 1.476 | -1.117, 4.069 | 0.26466368 | -2.79, 5.742 | 84.74 | 2.99 | 0.0105 |
| haloperidol | chronic | glua2 | dorsal striatum | mrna levels | 3 | 19 | 19 | 1.198 | -1.442, 3.838 | 0.37376296 | -3.864, 6.261 | 91.54 | 4.86 | 0.0012 |
| haloperidol | chronic | glua2 | frontal cortex | mrna levels | 3 | 19 | 19 | -0.401 | -1.182, 0.38 | 0.31473858 | -1.471, 0.67 | 29.24 | 0.14 | 0.2514 |
| haloperidol | chronic | glua2 | ventral striatum | mrna levels | 3 | 19 | 19 | 0.455 | -1.658, 2.568 | 0.67281193 | -3.567, 4.477 | 87.95 | 3.05 | 0.0003 |
| haloperidol | chronic | homer1a | associative cortex | mrna levels | 2 | 12 | 12 | -0.916 | -1.758, -0.073 | 0.03312873 | -1.758, -0.073 | 0 | 0 | 0.6848 |
| haloperidol | chronic | homer1a | cingulate cortex | mrna levels | 8 | 75 | 55 | 0.157 | -0.809, 1.123 | 0.75026541 | -2.475, 2.789 | 83.12 | 1.56 | 0 |
| haloperidol | chronic | homer1a | dorsal striatum | mrna levels | 9 | 80 | 60 | 1.73 | 1.122, 2.337 | 2.3713E-08 | 0.305, 3.155 | 51.92 | 0.43 | 0.0369 |
| haloperidol | chronic | homer1a | frontal cortex | mrna levels | 2 | 12 | 12 | -0.74 | -1.568, 0.088 | 0.07992892 | -1.568, 0.088 | 0 | 0 | 0.7272 |
| haloperidol | chronic | homer1a | limbic system | mrna levels | 7 | 68 | 48 | 0.203 | -0.805, 1.211 | 0.69281807 | -2.411, 2.817 | 82.49 | 1.51 | 0 |
| haloperidol | chronic | homer1a | sensory-motor areas | mrna levels | 7 | 68 | 48 | 0.418 | -0.773, 1.609 | 0.49134902 | -2.714, 3.551 | 86.68 | 2.19 | 0 |
| haloperidol | chronic | homer1a | ventral striatum | mrna levels | 8 | 75 | 55 | 1.245 | 0.803, 1.686 | 3.2718E-08 | 0.556, 1.934 | 17.98 | 0.07 | 0.3222 |
| haloperidol | chronic | homer1a | ventral striatum | protein levels | 2 | 12 | 12 | 0.501 | -0.315, 1.317 | 0.22904963 | -0.315, 1.317 | 0 | 0 | 0.5335 |
| haloperidol | chronic | homer1b-c | cingulate cortex | mrna levels | 5 | 46 | 36 | 0.351 | -0.822, 1.525 | 0.55727179 | -2.231, 2.934 | 81.7 | 1.38 | 0.0113 |
| haloperidol | chronic | homer1b-c | dorsal striatum | mrna levels | 5 | 46 | 36 | 0.49 | -0.276, 1.256 | 0.21020332 | -1.023, 2.003 | 59.66 | 0.44 | 0.0436 |
| haloperidol | chronic | homer1b-c | limbic system | mrna levels | 5 | 46 | 36 | 0.562 | -0.449, 1.572 | 0.27602183 | -1.622, 2.745 | 75.71 | 0.98 | 0.0029 |
| haloperidol | chronic | homer1b-c | sensory-motor areas | mrna levels | 5 | 46 | 36 | 0.182 | -0.463, 0.827 | 0.5804984 | -0.958, 1.322 | 43.73 | 0.23 | 0.0653 |
| haloperidol | chronic | homer1b-c | ventral striatum | mrna levels | 5 | 46 | 36 | 0.721 | -0.061, 1.502 | 0.07063019 | -0.832, 2.274 | 60.36 | 0.47 | 0.0328 |
| haloperidol | chronic | homer1b-c | ventral striatum | protein levels | 2 | 12 | 12 | -0.13 | -1.579, 1.318 | 0.85999021 | -2.349, 2.088 | 67.33 | 0.74 | 0.0802 |
| haloperidol | chronic | mglur1 | ventral striatum | protein levels | 2 | 12 | 12 | -0.518 | -1.333, 0.296 | 0.21198665 | -1.333, 0.296 | 0 | 0 | 0.7791 |
| haloperidol | chronic | mglur2 | frontal cortex | mrna levels | 2 | 10 | 10 | -0.243 | -1.132, 0.645 | 0.59174003 | -1.132, 0.645 | 0 | 0 | 0.3779 |
| haloperidol | chronic | mglur5 | frontal cortex | mrna levels | 2 | 10 | 10 | 0.311 | -0.571, 1.192 | 0.4899342 | -0.571, 1.192 | 0 | 0 | 0.9553 |
| haloperidol | chronic | mglur5 | frontal cortex | protein levels | 2 | 12 | 12 | 0.103 | -0.698, 0.904 | 0.80090662 | -0.698, 0.904 | 0 | 0 | 0.7728 |
| haloperidol | chronic | mglur5 | ventral striatum | protein levels | 2 | 12 | 12 | 0.138 | -0.668, 0.945 | 0.73677055 | -0.668, 0.945 | 0 | 0 | 0.4317 |
| haloperidol | chronic | norbin | frontal cortex | protein levels | 2 | 12 | 12 | -0.469 | -2.552, 1.614 | 0.65892406 | -3.863, 2.925 | 82.72 | 1.87 | 0.0161 |
| haloperidol | chronic | norbin | ventral striatum | protein levels | 2 | 12 | 12 | 1.004 | -0.129, 2.137 | 0.0823441 | -0.531, 2.539 | 41.51 | 0.28 | 0.191 |
| haloperidol | chronic | nr1 | dorsal striatum | mrna levels | 5 | 30 | 30 | 1.586 | 0.487, 2.684 | 0.00466178 | -0.708, 3.879 | 68.57 | 1.06 | 0.0153 |
| haloperidol | chronic | nr1 | dorsal striatum | protein levels | 3 | 23 | 23 | 0.666 | -0.379, 1.711 | 0.2115891 | -1.113, 2.445 | 63.49 | 0.54 | 0.0577 |
| haloperidol | chronic | nr1 | frontal cortex | mrna levels | 4 | 32 | 32 | 0.205 | -0.289, 0.7 | 0.4152762 | -0.289, 0.7 | 0 | 0 | 0.6654 |
| haloperidol | chronic | nr1 | frontal cortex | protein levels | 4 | 29 | 29 | -0.142 | -0.983, 0.7 | 0.74175033 | -1.661, 1.378 | 57.09 | 0.42 | 0.0795 |
| haloperidol | chronic | nr1 | hippocampus | mrna levels | 2 | 16 | 16 | 0.477 | -0.653, 1.606 | 0.40797822 | -1.187, 2.141 | 58.14 | 0.39 | 0.1222 |
| haloperidol | chronic | nr1 | hippocampus | protein levels | 4 | 29 | 29 | -0.264 | -0.786, 0.259 | 0.32257201 | -0.786, 0.259 | 0 | 0 | 0.4909 |
| haloperidol | chronic | nr1 | subcortical areas | protein levels | 2 | 14 | 14 | 0.092 | -0.65, 0.833 | 0.8087153 | -0.65, 0.833 | 0 | 0 | 0.8083 |
| haloperidol | chronic | nr1 | ventral striatum | mrna levels | 4 | 25 | 25 | 0.782 | -0.364, 1.927 | 0.18108743 | -1.433, 2.996 | 70.19 | 0.93 | 0.0245 |
| haloperidol | chronic | nr1 | ventral striatum | protein levels | 3 | 23 | 23 | 0.357 | -0.642, 1.357 | 0.48376684 | -1.315, 2.029 | 60.1 | 0.47 | 0.084 |
| haloperidol | chronic | nr2a | dorsal striatum | mrna levels | 2 | 15 | 15 | 0.148 | -2.97, 3.267 | 0.92566932 | -5.113, 5.41 | 92.28 | 4.67 | 0.0003 |
| haloperidol | chronic | nr2a | dorsal striatum | protein levels | 2 | 11 | 11 | 0.42 | -0.425, 1.265 | 0.32985018 | -0.425, 1.265 | 0 | 0 | 0.899 |
| haloperidol | chronic | nr2a | frontal cortex | mrna levels | 2 | 21 | 21 | -0.544 | -1.16, 0.072 | 0.08333199 | -1.16, 0.072 | 0 | 0 | 0.8752 |
| haloperidol | chronic | nr2a | frontal cortex | protein levels | 3 | 17 | 17 | -0.95 | -1.828, -0.072 | 0.03386986 | -2.176, 0.275 | 31.61 | 0.19 | 0.2228 |
| haloperidol | chronic | nr2a | hippocampus | mrna levels | 2 | 21 | 21 | -0.443 | -1.056, 0.169 | 0.15594529 | -1.056, 0.169 | 0 | 0 | 0.8803 |
| haloperidol | chronic | nr2a | hippocampus | protein levels | 3 | 17 | 17 | -0.551 | -1.33, 0.228 | 0.16574759 | -1.527, 0.426 | 18.89 | 0.09 | 0.2559 |
| haloperidol | chronic | nr2a | ventral striatum | protein levels | 2 | 11 | 11 | 0.086 | -0.755, 0.927 | 0.84077202 | -0.755, 0.927 | 0 | 0 | 0.4828 |
| haloperidol | chronic | nr2b | dorsal striatum | mrna levels | 2 | 15 | 15 | 1.346 | -0.218, 2.909 | 0.09155766 | -1.093, 3.785 | 71.56 | 0.91 | 0.0608 |
| haloperidol | chronic | nr2b | frontal cortex | protein levels | 2 | 11 | 11 | 0.35 | -0.936, 1.635 | 0.5939771 | -1.508, 2.207 | 54.2 | 0.47 | 0.1395 |
| haloperidol | chronic | nr2b | hippocampus | protein levels | 3 | 17 | 17 | -1.031 | -2.132, 0.069 | 0.06625326 | -2.817, 0.754 | 54.49 | 0.51 | 0.1101 |
| haloperidol | chronic | preso1 | frontal cortex | protein levels | 2 | 12 | 12 | 0.041 | -0.76, 0.842 | 0.91960294 | -0.76, 0.842 | 0 | 0 | 0.73 |
| haloperidol | chronic | psd95 | cingulate cortex | mrna levels | 4 | 39 | 29 | 0.583 | -1.472, 2.639 | 0.57816219 | -3.824, 4.99 | 91.34 | 3.96 | 0 |
| haloperidol | chronic | psd95 | dorsal striatum | mrna levels | 4 | 39 | 29 | 0.594 | -0.815, 2.003 | 0.40881273 | -2.318, 3.506 | 83.7 | 1.69 | 0.0003 |
| haloperidol | chronic | psd95 | frontal cortex | protein levels | 2 | 11 | 11 | -0.329 | -1.171, 0.512 | 0.44322913 | -1.171, 0.512 | 0 | 0 | 0.8848 |
| haloperidol | chronic | psd95 | limbic system | mrna levels | 4 | 39 | 29 | 0.013 | -0.991, 1.017 | 0.98030312 | -1.943, 1.968 | 71.13 | 0.73 | 0.0107 |
| haloperidol | chronic | psd95 | sensory-motor areas | mrna levels | 4 | 39 | 29 | 0.343 | -1.14, 1.827 | 0.64988362 | -2.753, 3.44 | 85.43 | 1.92 | 0.0001 |
| haloperidol | chronic | psd95 | ventral striatum | mrna levels | 4 | 39 | 29 | 0.173 | -0.963, 1.31 | 0.7652217 | -2.114, 2.461 | 77.14 | 1.03 | 0.0016 |
| haloperidol | chronic | tamalin | frontal cortex | protein levels | 2 | 12 | 12 | 0.135 | -0.666, 0.936 | 0.74079611 | -0.666, 0.936 | 0 | 0 | 0.9777 |
| haloperidol decanoate | chronic | glua2 | dorsal striatum | mrna levels | 2 | 13 | 13 | 0.973 | -0.106, 2.052 | 0.07706322 | -0.482, 2.429 | 40.37 | 0.25 | 0.1953 |
| olanzapine | acute | arc | dorsal striatum | mrna levels | 3 | 48 | 48 | 1.41 | 0.374, 2.446 | 0.00766289 | -0.482, 3.302 | 78.54 | 0.65 | 0.0162 |
| olanzapine | acute | arc | frontal cortex | mrna levels | 2 | 41 | 41 | -2.154 | -2.699, -1.61 | 8.71E-15 | -2.699, -1.61 | 0 | 0 | 0.737 |
| olanzapine | acute | arc | ventral striatum | mrna levels | 2 | 27 | 27 | 1.486 | 0.882, 2.091 | 1.4558E-06 | 0.882, 2.091 | 0 | 0 | 0.387 |
| olanzapine | acute | homer1a | cingulate cortex | mrna levels | 2 | 12 | 12 | 0.701 | -0.967, 2.368 | 0.41011729 | -1.918, 3.32 | 73.34 | 1.06 | 0.0528 |
| olanzapine | acute | homer1a | dorsal striatum | mrna levels | 4 | 22 | 22 | 3.442 | -1.378, 8.261 | 0.16162121 | -6.974, 13.857 | 97.2 | 22.19 | 0.0001 |
| olanzapine | acute | homer1a | limbic system | mrna levels | 2 | 12 | 12 | 0.947 | -0.16, 2.053 | 0.09368945 | -0.535, 2.428 | 39.54 | 0.25 | 0.1984 |
| olanzapine | acute | homer1a | sensory-motor areas | mrna levels | 2 | 12 | 12 | 0.641 | -0.674, 1.957 | 0.33920261 | -1.298, 2.581 | 58.66 | 0.53 | 0.1199 |
| olanzapine | acute | homer1a | ventral striatum | mrna levels | 4 | 22 | 22 | 1.845 | 0.27, 3.419 | 0.02164256 | -1.318, 5.007 | 77.74 | 1.96 | 0.0086 |
| olanzapine | acute | psd95 | dorsal striatum | mrna levels | 2 | 12 | 12 | 2.066 | -0.591, 4.723 | 0.12756396 | -2.294, 6.426 | 84.44 | 3.11 | 0.0113 |
| olanzapine | acute | psd95 | frontal cortex | protein levels | 2 | 12 | 12 | 0.484 | -0.433, 1.401 | 0.30107096 | -0.598, 1.566 | 19.57 | 0.09 | 0.2648 |
| olanzapine | acute | psd95 | hippocampus | protein levels | 2 | 12 | 12 | -0.497 | -1.893, 0.899 | 0.48531777 | -2.606, 1.612 | 64 | 0.65 | 0.0956 |
| olanzapine | acute | psd95 | ventral striatum | mrna levels | 2 | 12 | 12 | 1.438 | -1.025, 3.9 | 0.25247242 | -2.608, 5.483 | 84.92 | 2.68 | 0.01 |
| olanzapine | chronic | arc | dorsal striatum | mrna levels | 2 | 19 | 19 | -1.597 | -2.328, -0.867 | 1.8322E-05 | -2.328, -0.867 | 0 | 0 | 0.7685 |
| olanzapine | chronic | homer1a | cingulate cortex | mrna levels | 2 | 10 | 10 | 0.2 | -0.686, 1.085 | 0.65825126 | -0.686, 1.085 | 0 | 0 | 0.4347 |
| olanzapine | chronic | homer1a | dorsal striatum | mrna levels | 2 | 10 | 10 | 0.342 | -0.631, 1.314 | 0.49105315 | -0.767, 1.45 | 15 | 0.07 | 0.2781 |
| olanzapine | chronic | homer1a | limbic system | mrna levels | 2 | 10 | 10 | -0.245 | -1.127, 0.636 | 0.58547176 | -1.127, 0.636 | 0 | 0 | 0.6992 |
| olanzapine | chronic | homer1a | sensory-motor areas | mrna levels | 2 | 10 | 10 | 0.322 | -0.789, 1.432 | 0.57038596 | -1.121, 1.764 | 34.37 | 0.22 | 0.2171 |
| olanzapine | chronic | homer1a | ventral striatum | mrna levels | 2 | 10 | 10 | 1.185 | -1.506, 3.875 | 0.38814612 | -3.237, 5.606 | 84.85 | 3.2 | 0.0102 |
| olanzapine | chronic | mglur5 | frontal cortex | protein levels | 2 | 12 | 12 | 0.222 | -0.581, 1.025 | 0.58811116 | -0.581, 1.025 | 0 | 0 | 0.7776 |
| olanzapine | chronic | norbin | frontal cortex | protein levels | 2 | 12 | 12 | -0.039 | -0.84, 0.763 | 0.92462699 | -0.84, 0.763 | 0 | 0 | 0.6702 |
| olanzapine | chronic | nr1 | frontal cortex | protein levels | 2 | 12 | 12 | 0.461 | -1.201, 2.124 | 0.58652779 | -2.158, 3.081 | 74.09 | 1.07 | 0.0495 |
| olanzapine | chronic | nr1 | hippocampus | mrna levels | 2 | 11 | 11 | -0.403 | -2.188, 1.381 | 0.65770231 | -3.225, 2.418 | 74.89 | 1.24 | 0.046 |
| olanzapine | chronic | nr1 | hippocampus | protein levels | 3 | 18 | 18 | -0.292 | -1.01, 0.426 | 0.42533578 | -1.143, 0.559 | 13.47 | 0.05 | 0.3056 |
| olanzapine | chronic | nr2a | frontal cortex | protein levels | 2 | 12 | 12 | -0.064 | -1.2, 1.071 | 0.91150552 | -1.656, 1.528 | 48.25 | 0.32 | 0.1645 |
| olanzapine | chronic | nr2a | hippocampus | mrna levels | 2 | 11 | 11 | 0.247 | -0.599, 1.093 | 0.56745235 | -0.599, 1.093 | 0 | 0 | 0.3813 |
| olanzapine | chronic | nr2a | hippocampus | protein levels | 3 | 18 | 18 | -0.089 | -0.749, 0.57 | 0.7907244 | -0.749, 0.57 | 0 | 0 | 0.5197 |
| olanzapine | chronic | nr2b | hippocampus | mrna levels | 2 | 11 | 11 | 0.659 | -0.203, 1.52 | 0.13399263 | -0.203, 1.52 | 0 | 0 | 0.5523 |
| olanzapine | chronic | nr2b | hippocampus | protein levels | 3 | 18 | 18 | -0.278 | -1.261, 0.704 | 0.57862304 | -1.856, 1.299 | 52.55 | 0.4 | 0.122 |
| olanzapine | chronic | preso1 | frontal cortex | protein levels | 2 | 12 | 12 | 0.065 | -0.736, 0.865 | 0.87449228 | -0.736, 0.865 | 0 | 0 | 0.8204 |
| olanzapine | chronic | psd95 | frontal cortex | protein levels | 2 | 11 | 11 | -0.65 | -1.517, 0.216 | 0.14130485 | -1.517, 0.216 | 0 | 0 | 0.3406 |
| olanzapine | chronic | tamalin | frontal cortex | protein levels | 2 | 12 | 12 | 0.399 | -0.413, 1.212 | 0.33558894 | -0.413, 1.212 | 0 | 0 | 0.4663 |
| quetiapine | acute | homer1a | dorsal striatum | mrna levels | 2 | 24 | 12 | 0.436 | -0.264, 1.136 | 0.22239809 | -0.264, 1.136 | 0 | 0 | 0.9673 |
| quetiapine | chronic | homer1a | dorsal striatum | mrna levels | 2 | 19 | 12 | 0.648 | -0.822, 2.119 | 0.38764893 | -1.624, 2.921 | 68.25 | 0.78 | 0.076 |

## Sensitivity analyses based on animal sex

| **drug** | **model** | **psd type** | **region** | **outcome type** | **n_studies** | **n_group_1** | **n_group_2** | **beta** | **CI** | **p_val** | **PI** | **i_sq** | **tau_sq** | **q_p_val** |
| --- | --- | --- | --- | --- | --- | --- | --- | --- | --- | --- | --- | --- | --- | --- |
| amisulpride | chronic | arc | cingulate cortex | mrna levels | 2 | 12 | 12 | 0.22 | -0.583, 1.024 | 0.59115941 | -0.583, 1.024 | 0 | 0 | 0.7398 |
| amisulpride | chronic | arc | dorsal striatum | mrna levels | 2 | 12 | 12 | 1.235 | 0.359, 2.111 | 0.00573881 | 0.359, 2.111 | 0 | 0 | 0.5518 |
| amisulpride | chronic | arc | limbic system | mrna levels | 2 | 12 | 12 | 0.454 | -0.485, 1.393 | 0.34349765 | -0.684, 1.592 | 23.38 | 0.11 | 0.2533 |
| amisulpride | chronic | arc | sensory-motor areas | mrna levels | 2 | 12 | 12 | 0.12 | -0.685, 0.925 | 0.77032363 | -0.685, 0.925 | 0 | 0 | 0.476 |
| amisulpride | chronic | arc | ventral striatum | mrna levels | 2 | 12 | 12 | 0.825 | -0.01, 1.66 | 0.05270135 | -0.01, 1.66 | 0 | 0 | 0.6841 |
| amisulpride | chronic | homer1a | cingulate cortex | mrna levels | 2 | 12 | 12 | 1.355 | -1.757, 4.466 | 0.39347591 | -3.848, 6.557 | 89.66 | 4.53 | 0.0019 |
| amisulpride | chronic | homer1a | dorsal striatum | mrna levels | 2 | 12 | 12 | 1.804 | -0.412, 4.02 | 0.11065992 | -1.764, 5.372 | 79.2 | 2.04 | 0.0283 |
| amisulpride | chronic | homer1a | limbic system | mrna levels | 2 | 12 | 12 | 2.678 | -3.045, 8.401 | 0.3590189 | -7.041, 12.397 | 94.06 | 16.06 | 0 |
| amisulpride | chronic | homer1a | sensory-motor areas | mrna levels | 2 | 12 | 12 | 0.575 | -2.134, 3.285 | 0.67732768 | -3.94, 5.091 | 88.81 | 3.4 | 0.0028 |
| amisulpride | chronic | homer1a | ventral striatum | mrna levels | 2 | 12 | 12 | 0.982 | -0.103, 2.067 | 0.07613854 | -0.449, 2.413 | 36.76 | 0.23 | 0.2086 |
| amisulpride | chronic | homer1b-c | cingulate cortex | mrna levels | 2 | 12 | 12 | 0.189 | -1.742, 2.12 | 0.84805566 | -2.933, 3.31 | 80.66 | 1.57 | 0.023 |
| amisulpride | chronic | homer1b-c | dorsal striatum | mrna levels | 2 | 12 | 12 | 0.972 | -2.829, 4.772 | 0.61623365 | -5.454, 7.398 | 92.92 | 6.99 | 0.0002 |
| amisulpride | chronic | homer1b-c | limbic system | mrna levels | 2 | 12 | 12 | 0.388 | -2.039, 2.814 | 0.7542967 | -3.627, 4.402 | 86.82 | 2.66 | 0.0059 |
| amisulpride | chronic | homer1b-c | sensory-motor areas | mrna levels | 2 | 12 | 12 | 0.282 | -1.513, 2.078 | 0.75789314 | -2.589, 3.154 | 77.85 | 1.31 | 0.0336 |
| amisulpride | chronic | homer1b-c | ventral striatum | mrna levels | 2 | 12 | 12 | 1.215 | -0.913, 3.344 | 0.26305651 | -2.232, 4.663 | 80.98 | 1.91 | 0.0218 |
| amisulpride | chronic | psd95 | cingulate cortex | mrna levels | 2 | 12 | 12 | -0.006 | -0.806, 0.794 | 0.9882636 | -0.806, 0.794 | 0 | 0 | 0.883 |
| amisulpride | chronic | psd95 | dorsal striatum | mrna levels | 2 | 12 | 12 | -0.438 | -1.825, 0.949 | 0.53623677 | -2.53, 1.655 | 63.79 | 0.64 | 0.0966 |
| amisulpride | chronic | psd95 | limbic system | mrna levels | 2 | 12 | 12 | -0.507 | -1.337, 0.324 | 0.23176349 | -1.355, 0.341 | 2.11 | 0.01 | 0.3121 |
| amisulpride | chronic | psd95 | sensory-motor areas | mrna levels | 2 | 12 | 12 | -0.063 | -0.864, 0.737 | 0.87678829 | -0.864, 0.737 | 0 | 0 | 0.8767 |
| amisulpride | chronic | psd95 | ventral striatum | mrna levels | 2 | 12 | 12 | -0.08 | -0.882, 0.722 | 0.844924 | -0.882, 0.722 | 0 | 0 | 0.6302 |
| aripiprazole | acute | arc | dorsal striatum | mrna levels | 2 | 35 | 25 | 1.006 | -2.546, 4.558 | 0.5788137 | -5.071, 7.083 | 96.33 | 6.33 | 0 |
| aripiprazole | acute | arc | frontal cortex | mrna levels | 2 | 35 | 25 | -2.268 | -4.586, 0.051 | 0.05521776 | -6.151, 1.616 | 90.29 | 2.53 | 0.0013 |
| aripiprazole | chronic | homer1a | ventral striatum | protein levels | 2 | 12 | 12 | 2.574 | 0.261, 4.886 | 0.02914093 | -1.097, 6.245 | 75.35 | 2.12 | 0.044 |
| aripiprazole | chronic | homer1b-c | ventral striatum | protein levels | 2 | 12 | 12 | -0.623 | -3.312, 2.065 | 0.6495305 | -5.101, 3.854 | 88.6 | 3.34 | 0.0031 |
| aripiprazole | chronic | mglur1 | ventral striatum | protein levels | 2 | 12 | 12 | -0.371 | -1.178, 0.436 | 0.36756978 | -1.178, 0.436 | 0 | 0 | 0.9855 |
| aripiprazole | chronic | mglur5 | ventral striatum | protein levels | 2 | 12 | 12 | 0.12 | -1.864, 2.104 | 0.90559419 | -3.1, 3.34 | 81.63 | 1.67 | 0.0197 |
| aripiprazole | chronic | norbin | ventral striatum | protein levels | 2 | 12 | 12 | 1.725 | 0.782, 2.667 | 0.00033353 | 0.782, 2.667 | 0 | 0 | 0.4768 |
| aripiprazole | chronic | nr1 | frontal cortex | protein levels | 2 | 12 | 12 | -0.002 | -0.802, 0.798 | 0.99583721 | -0.802, 0.798 | 0 | 0 | 0.9958 |
| aripiprazole | chronic | nr1 | ventral striatum | protein levels | 2 | 12 | 12 | 0.321 | -1.858, 2.501 | 0.77254555 | -3.25, 3.892 | 84.25 | 2.08 | 0.0117 |
| aripiprazole | chronic | psd95 | frontal cortex | protein levels | 2 | 10 | 10 | 1.46 | 0.47, 2.45 | 0.00385209 | 0.47, 2.45 | 0 | 0 | 0.5864 |
| asenapine | acute | homer1a | cingulate cortex | mrna levels | 4 | 46 | 22 | 0.122 | -0.794, 1.038 | 0.79451752 | -1.593, 1.836 | 63.3 | 0.55 | 0.0438 |
| asenapine | acute | homer1a | dorsal striatum | mrna levels | 4 | 46 | 22 | 1.019 | 0.464, 1.574 | 0.00031943 | 0.464, 1.574 | 0 | 0 | 0.4963 |
| asenapine | acute | homer1a | hippocampus | mrna levels | 2 | 10 | 10 | -0.751 | -3.629, 2.127 | 0.60926092 | -5.524, 4.023 | 87.51 | 3.78 | 0.0047 |
| asenapine | acute | homer1a | limbic system | mrna levels | 4 | 46 | 22 | 0.274 | -0.344, 0.893 | 0.38436891 | -0.584, 1.133 | 22.98 | 0.09 | 0.2417 |
| asenapine | acute | homer1a | sensory-motor areas | mrna levels | 4 | 46 | 22 | 0.21 | -0.557, 0.978 | 0.59128363 | -1.104, 1.525 | 48.69 | 0.3 | 0.1174 |
| asenapine | acute | homer1a | ventral striatum | mrna levels | 4 | 46 | 22 | 0.57 | -0.239, 1.379 | 0.16711397 | -0.857, 1.997 | 53.13 | 0.36 | 0.0923 |
| asenapine | chronic | homer1a | cingulate cortex | mrna levels | 2 | 30 | 10 | -0.064 | -0.78, 0.652 | 0.86107453 | -0.78, 0.652 | 0 | 0 | 0.861 |
| asenapine | chronic | homer1a | dorsal striatum | mrna levels | 2 | 30 | 10 | 0.137 | -0.782, 1.057 | 0.76951282 | -1.083, 1.358 | 38.08 | 0.17 | 0.2038 |
| asenapine | chronic | homer1a | limbic system | mrna levels | 2 | 30 | 10 | -1.231 | -2.076, -0.385 | 0.0043296 | -2.208, -0.253 | 16.8 | 0.06 | 0.2729 |
| asenapine | chronic | homer1a | sensory-motor areas | mrna levels | 2 | 30 | 10 | -0.06 | -0.776, 0.657 | 0.86965689 | -0.776, 0.657 | 0 | 0 | 0.6934 |
| asenapine | chronic | homer1a | ventral striatum | mrna levels | 2 | 30 | 10 | 0.431 | -0.458, 1.32 | 0.34183304 | -0.713, 1.576 | 32.81 | 0.14 | 0.2225 |
| clozapine | acute | arc | dorsal striatum | mrna levels | 3 | 32 | 32 | -0.135 | -0.703, 0.434 | 0.64262337 | -0.843, 0.574 | 16.22 | 0.05 | 0.3683 |
| clozapine | acute | arc | frontal cortex | mrna levels | 3 | 32 | 32 | -0.914 | -2.08, 0.252 | 0.12460827 | -3.011, 1.183 | 75.01 | 0.79 | 0.0149 |
| clozapine | acute | arc | ventral striatum | mrna levels | 2 | 25 | 25 | -0.019 | -1.872, 1.834 | 0.98390548 | -3.065, 3.027 | 84.34 | 1.52 | 0.0115 |
| clozapine | acute | homer1a | cingulate cortex | mrna levels | 2 | 12 | 12 | 2.119 | 1.119, 3.119 | 3.2667E-05 | 1.119, 3.119 | 0 | 0 | 0.9358 |
| clozapine | acute | homer1a | dorsal striatum | mrna levels | 3 | 17 | 17 | 1.131 | 0.406, 1.856 | 0.00223067 | 0.406, 1.856 | 0 | 0 | 0.9033 |
| clozapine | acute | homer1a | ventral striatum | mrna levels | 3 | 17 | 17 | 1.478 | 0.716, 2.239 | 0.00014279 | 0.716, 2.239 | 0 | 0 | 0.7652 |
| clozapine | acute | mglur1 | frontal cortex | mrna levels | 2 | 10 | 10 | 0.931 | -0.036, 1.898 | 0.05904279 | -0.095, 1.957 | 6.29 | 0.03 | 0.3016 |
| clozapine | acute | mglur2 | frontal cortex | mrna levels | 2 | 10 | 10 | 0.042 | -0.839, 0.923 | 0.92540925 | -0.839, 0.923 | 0 | 0 | 0.5292 |
| clozapine | acute | mglur5 | frontal cortex | mrna levels | 2 | 10 | 10 | -0.651 | -1.551, 0.249 | 0.15624973 | -1.551, 0.249 | 0 | 0 | 0.7929 |
| clozapine | acute | nr1 | frontal cortex | mrna levels | 3 | 15 | 15 | 1.186 | 0.406, 1.966 | 0.00288038 | 0.406, 1.966 | 0 | 0 | 0.7299 |
| clozapine | acute | nr1 | hippocampus | mrna levels | 3 | 15 | 15 | -0.336 | -1.067, 0.394 | 0.36697868 | -1.067, 0.394 | 0 | 0 | 0.4477 |
| clozapine | acute | nr2a | frontal cortex | mrna levels | 3 | 15 | 15 | 1.01 | 0.241, 1.778 | 0.01000978 | 0.241, 1.778 | 0 | 0 | 0.5164 |
| clozapine | acute | nr2a | hippocampus | mrna levels | 3 | 15 | 15 | 0.049 | -0.677, 0.776 | 0.89395175 | -0.677, 0.776 | 0 | 0 | 0.4126 |
| clozapine | acute | nr2b | frontal cortex | mrna levels | 3 | 15 | 15 | 0.742 | -0.001, 1.485 | 0.0504208 | -0.001, 1.485 | 0 | 0 | 0.7696 |
| clozapine | acute | nr2b | hippocampus | mrna levels | 3 | 15 | 15 | 0.016 | -0.701, 0.734 | 0.96426836 | -0.701, 0.734 | 0 | 0 | 0.8651 |
| clozapine | chronic | mglur1 | frontal cortex | mrna levels | 2 | 20 | 10 | 0.189 | -0.572, 0.95 | 0.62714207 | -0.572, 0.95 | 0 | 0 | 0.7714 |
| clozapine | chronic | mglur2 | frontal cortex | mrna levels | 3 | 25 | 15 | 0.106 | -0.552, 0.764 | 0.75178475 | -0.552, 0.764 | 0 | 0 | 0.3195 |
| clozapine | chronic | mglur5 | frontal cortex | mrna levels | 3 | 25 | 15 | 0.079 | -0.568, 0.727 | 0.81067085 | -0.568, 0.727 | 0 | 0 | 0.9975 |
| clozapine | chronic | nr1 | dorsal striatum | mrna levels | 4 | 29 | 29 | 0.89 | -0.876, 2.656 | 0.3230521 | -2.828, 4.609 | 88.91 | 2.79 | 0.0019 |
| clozapine | chronic | nr1 | dorsal striatum | protein levels | 2 | 17 | 17 | 0.761 | 0.065, 1.457 | 0.03219395 | 0.065, 1.457 | 0 | 0 | 0.9034 |
| clozapine | chronic | nr1 | frontal cortex | mrna levels | 4 | 35 | 35 | 0.138 | -0.545, 0.822 | 0.69196945 | -1.043, 1.319 | 49.79 | 0.24 | 0.1153 |
| clozapine | chronic | nr1 | frontal cortex | protein levels | 2 | 17 | 17 | -0.566 | -1.255, 0.123 | 0.10753823 | -1.255, 0.123 | 0 | 0 | 0.4038 |
| clozapine | chronic | nr1 | hippocampus | mrna levels | 3 | 24 | 24 | 0.656 | 0.007, 1.304 | 0.04744702 | -0.14, 1.451 | 16.55 | 0.06 | 0.2679 |
| clozapine | chronic | nr1 | hippocampus | protein levels | 4 | 28 | 28 | -0.285 | -0.989, 0.418 | 0.42680278 | -1.395, 0.824 | 37.1 | 0.19 | 0.1764 |
| clozapine | chronic | nr1 | ventral striatum | protein levels | 2 | 17 | 17 | 0.076 | -0.736, 0.888 | 0.85489505 | -0.929, 1.08 | 23.83 | 0.09 | 0.2519 |
| clozapine | chronic | nr2a | dorsal striatum | mrna levels | 2 | 15 | 15 | 0.445 | -0.719, 1.609 | 0.45363294 | -1.26, 2.15 | 56.34 | 0.4 | 0.1302 |
| clozapine | chronic | nr2a | frontal cortex | mrna levels | 2 | 21 | 21 | -0.565 | -1.182, 0.052 | 0.07251751 | -1.182, 0.052 | 0 | 0 | 0.9514 |
| clozapine | chronic | nr2a | hippocampus | mrna levels | 2 | 21 | 21 | -0.344 | -0.953, 0.266 | 0.26964694 | -0.953, 0.266 | 0 | 0 | 0.685 |
| clozapine | chronic | nr2a | hippocampus | protein levels | 2 | 11 | 11 | -0.29 | -1.137, 0.556 | 0.50104712 | -1.137, 0.556 | 0 | 0 | 0.4319 |
| clozapine | chronic | nr2b | dorsal striatum | mrna levels | 2 | 15 | 15 | 0.895 | -0.007, 1.797 | 0.0517365 | -0.218, 2.008 | 23.35 | 0.11 | 0.2534 |
| clozapine | chronic | nr2b | hippocampus | protein levels | 2 | 11 | 11 | -0.773 | -2.516, 0.969 | 0.38441126 | -3.501, 1.955 | 72.22 | 1.15 | 0.0578 |
| flupenthixol | chronic | nr1 | associative cortex | mrna levels | 2 | 16 | 16 | -1.687 | -2.495, -0.88 | 4.1643E-05 | -2.495, -0.88 | 0 | 0 | 0.8821 |
| flupenthixol | chronic | nr1 | cerebellum | mrna levels | 2 | 16 | 16 | -1.441 | -2.22, -0.662 | 0.0002901 | -2.22, -0.662 | 0 | 0 | 0.6117 |
| flupenthixol | chronic | nr1 | frontal cortex | mrna levels | 2 | 16 | 16 | -1.505 | -2.367, -0.643 | 0.00062435 | -2.492, -0.518 | 15.35 | 0.06 | 0.2771 |
| flupenthixol | chronic | nr1 | subcortical areas | mrna levels | 2 | 16 | 16 | -1.338 | -2.139, -0.538 | 0.00105306 | -2.191, -0.485 | 6.69 | 0.02 | 0.3006 |
| flupenthixol | chronic | nr2b | associative cortex | mrna levels | 2 | 16 | 16 | -0.116 | -1.377, 1.144 | 0.8566824 | -2.054, 1.821 | 68.16 | 0.56 | 0.0764 |
| flupenthixol | chronic | nr2b | cerebellum | mrna levels | 2 | 16 | 16 | 0.593 | -0.12, 1.305 | 0.10316132 | -0.12, 1.305 | 0 | 0 | 0.3593 |
| flupenthixol | chronic | nr2b | frontal cortex | mrna levels | 2 | 16 | 16 | -0.063 | -0.76, 0.634 | 0.85994183 | -0.76, 0.634 | 0 | 0 | 0.3978 |
| flupenthixol | chronic | nr2b | subcortical areas | mrna levels | 2 | 16 | 16 | 0.076 | -1.266, 1.417 | 0.91200128 | -2.017, 2.169 | 71.73 | 0.67 | 0.06 |
| flupenthixol | chronic | nr2c | associative cortex | mrna levels | 2 | 16 | 16 | -0.067 | -0.762, 0.628 | 0.84984417 | -0.762, 0.628 | 0 | 0 | 0.5402 |
| flupenthixol | chronic | nr2c | cerebellum | mrna levels | 2 | 16 | 16 | -0.047 | -1.612, 1.519 | 0.95318765 | -2.56, 2.466 | 78.84 | 1.01 | 0.0297 |
| flupenthixol | chronic | nr2c | frontal cortex | mrna levels | 2 | 16 | 16 | -0.305 | -1.671, 1.061 | 0.66166713 | -2.441, 1.832 | 72.38 | 0.7 | 0.0571 |
| flupenthixol | chronic | nr2c | subcortical areas | mrna levels | 2 | 16 | 16 | 0.779 | 0.056, 1.503 | 0.03474787 | 0.056, 1.503 | 0 | 0 | 0.3628 |
| flupenthixol | chronic | nr2d | associative cortex | mrna levels | 2 | 16 | 16 | -1.595 | -4.331, 1.142 | 0.25337054 | -6.173, 2.984 | 89.86 | 3.51 | 0.0017 |
| flupenthixol | chronic | nr2d | cerebellum | mrna levels | 2 | 16 | 16 | 0.899 | 0.041, 1.757 | 0.0399452 | -0.163, 1.961 | 26.48 | 0.1 | 0.2435 |
| flupenthixol | chronic | nr2d | frontal cortex | mrna levels | 2 | 16 | 16 | -0.019 | -0.713, 0.675 | 0.95734427 | -0.713, 0.675 | 0 | 0 | 0.6725 |
| flupenthixol | chronic | nr2d | subcortical areas | mrna levels | 2 | 16 | 16 | 0.669 | -0.1, 1.438 | 0.08834587 | -0.193, 1.531 | 12.74 | 0.04 | 0.2844 |
| haloperidol | acute | ania3 | associative cortex | mrna levels | 3 | 17 | 17 | 0.018 | -0.977, 1.013 | 0.97178106 | -1.565, 1.601 | 51.03 | 0.39 | 0.1294 |
| haloperidol | acute | ania3 | cingulate cortex | mrna levels | 4 | 23 | 23 | 0.997 | -0.261, 2.255 | 0.12030827 | -1.478, 3.472 | 72.81 | 1.18 | 0.0125 |
| haloperidol | acute | ania3 | dorsal striatum | mrna levels | 5 | 28 | 29 | 2.471 | 1.076, 3.866 | 0.00051852 | -0.521, 5.463 | 73.64 | 1.82 | 0.0053 |
| haloperidol | acute | ania3 | frontal cortex | mrna levels | 3 | 17 | 17 | -0.136 | -0.817, 0.545 | 0.69530376 | -0.817, 0.545 | 0 | 0 | 0.4424 |
| haloperidol | acute | ania3 | limbic system | mrna levels | 2 | 11 | 11 | 0.615 | -0.241, 1.471 | 0.15884517 | -0.241, 1.471 | 0 | 0 | 0.8625 |
| haloperidol | acute | ania3 | sensory-motor areas | mrna levels | 2 | 11 | 11 | 0.384 | -0.901, 1.67 | 0.5578257 | -1.454, 2.223 | 50.8 | 0.45 | 0.154 |
| haloperidol | acute | ania3 | ventral striatum | mrna levels | 5 | 28 | 29 | 1.425 | 0.607, 2.243 | 0.00064121 | -0.056, 2.906 | 45.76 | 0.4 | 0.1156 |
| haloperidol | acute | arc | cingulate cortex | mrna levels | 5 | 46 | 32 | 0.022 | -0.932, 0.976 | 0.96388064 | -2.026, 2.07 | 72.9 | 0.85 | 0.0035 |
| haloperidol | acute | arc | dorsal striatum | mrna levels | 10 | 114 | 90 | 2.598 | 1.338, 3.857 | 5.2739E-05 | -1.292, 6.488 | 89.96 | 3.53 | 0 |
| haloperidol | acute | arc | frontal cortex | mrna levels | 5 | 68 | 58 | -0.949 | -1.327, -0.57 | 9.0674E-07 | -1.327, -0.57 | 0 | 0 | 0.3895 |
| haloperidol | acute | arc | limbic system | mrna levels | 5 | 46 | 32 | -0.212 | -1.183, 0.759 | 0.66867532 | -2.303, 1.879 | 73.57 | 0.89 | 0.0051 |
| haloperidol | acute | arc | sensory-motor areas | mrna levels | 5 | 46 | 32 | -0.36 | -1.163, 0.442 | 0.3789775 | -1.984, 1.263 | 62.44 | 0.52 | 0.0278 |
| haloperidol | acute | arc | ventral striatum | mrna levels | 7 | 71 | 57 | 1.73 | 1.138, 2.321 | 9.8977E-09 | 0.55, 2.909 | 43.97 | 0.27 | 0.0779 |
| haloperidol | acute | glua1 pser845 | dorsal striatum | protein levels | 4 | 66 | 30 | 2.562 | 0.681, 4.442 | 0.00758779 | -1.381, 6.505 | 89.74 | 3.13 | 0.0009 |
| haloperidol | acute | homer1a | associative cortex | mrna levels | 3 | 17 | 17 | -0.361 | -1.47, 0.749 | 0.52395279 | -2.203, 1.482 | 58.76 | 0.56 | 0.0945 |
| haloperidol | acute | homer1a | cingulate cortex | mrna levels | 11 | 98 | 74 | 0.151 | -0.266, 0.568 | 0.47875811 | -0.8, 1.101 | 39.03 | 0.19 | 0.0559 |
| haloperidol | acute | homer1a | dorsal striatum | mrna levels | 15 | 118 | 94 | 2.114 | 1.428, 2.8 | 1.5719E-09 | -0.097, 4.325 | 70.36 | 1.15 | 0 |
| haloperidol | acute | homer1a | frontal cortex | mrna levels | 3 | 17 | 17 | 0.033 | -0.647, 0.713 | 0.92422709 | -0.647, 0.713 | 0 | 0 | 0.4486 |
| haloperidol | acute | homer1a | limbic system | mrna levels | 9 | 86 | 62 | -0.488 | -1.101, 0.125 | 0.11839857 | -2.057, 1.081 | 64.51 | 0.54 | 0.0057 |
| haloperidol | acute | homer1a | sensory-motor areas | mrna levels | 9 | 86 | 62 | -0.081 | -0.526, 0.364 | 0.72227488 | -0.994, 0.832 | 36.58 | 0.17 | 0.0507 |
| haloperidol | acute | homer1a | ventral striatum | mrna levels | 14 | 113 | 89 | 1.81 | 1.143, 2.477 | 1.0368E-07 | -0.34, 3.96 | 72.04 | 1.09 | 0 |
| haloperidol | acute | homer1b-c | cingulate cortex | mrna levels | 3 | 35 | 21 | 0.527 | -0.319, 1.374 | 0.22198505 | -0.828, 1.883 | 52.17 | 0.29 | 0.1239 |
| haloperidol | acute | homer1b-c | dorsal striatum | mrna levels | 3 | 35 | 21 | 0.693 | 0.011, 1.375 | 0.04647557 | -0.223, 1.608 | 26.56 | 0.1 | 0.2561 |
| haloperidol | acute | homer1b-c | limbic system | mrna levels | 3 | 35 | 21 | 0.466 | -0.386, 1.318 | 0.28390108 | -0.905, 1.837 | 53.01 | 0.3 | 0.1196 |
| haloperidol | acute | homer1b-c | sensory-motor areas | mrna levels | 3 | 35 | 21 | 0.78 | 0.2, 1.359 | 0.0083775 | 0.2, 1.359 | 0 | 0 | 0.5732 |
| haloperidol | acute | homer1b-c | ventral striatum | mrna levels | 3 | 35 | 21 | 0.634 | -0.266, 1.534 | 0.16734847 | -0.848, 2.116 | 57.14 | 0.36 | 0.0967 |
| haloperidol | acute | nr1 | frontal cortex | mrna levels | 3 | 15 | 15 | 0.173 | -0.547, 0.893 | 0.63795846 | -0.547, 0.893 | 0 | 0 | 0.7545 |
| haloperidol | acute | nr1 | hippocampus | mrna levels | 3 | 15 | 15 | -0.067 | -0.783, 0.649 | 0.85489032 | -0.783, 0.649 | 0 | 0 | 0.9714 |
| haloperidol | acute | nr2a | frontal cortex | mrna levels | 3 | 15 | 15 | 0.104 | -0.818, 1.026 | 0.82474506 | -1.217, 1.425 | 34.85 | 0.23 | 0.2292 |
| haloperidol | acute | nr2a | hippocampus | mrna levels | 3 | 15 | 15 | 0.288 | -0.982, 1.559 | 0.65652057 | -1.878, 2.454 | 63.64 | 0.8 | 0.0607 |
| haloperidol | acute | nr2b | frontal cortex | mrna levels | 3 | 15 | 15 | 0.132 | -0.59, 0.854 | 0.7207997 | -0.59, 0.854 | 0 | 0 | 0.6226 |
| haloperidol | acute | nr2b | hippocampus | mrna levels | 3 | 15 | 15 | 0.485 | -0.252, 1.222 | 0.19671704 | -0.252, 1.222 | 0 | 0 | 0.4179 |
| haloperidol | acute | nr2b ptyr1472 | dorsal striatum | protein levels | 2 | 10 | 10 | 0.611 | -0.784, 2.006 | 0.39087515 | -1.422, 2.644 | 56.07 | 0.57 | 0.1314 |
| haloperidol | acute | psd95 | cingulate cortex | mrna levels | 2 | 28 | 14 | -0.027 | -2.344, 2.29 | 0.98171794 | -3.906, 3.852 | 90.1 | 2.52 | 0.0015 |
| haloperidol | acute | psd95 | dorsal striatum | mrna levels | 3 | 33 | 19 | 0.388 | -0.802, 1.578 | 0.52284253 | -1.725, 2.5 | 72.12 | 0.79 | 0.0255 |
| haloperidol | acute | psd95 | limbic system | mrna levels | 2 | 28 | 14 | -0.155 | -0.818, 0.509 | 0.64800273 | -0.818, 0.509 | 0 | 0 | 0.898 |
| haloperidol | acute | psd95 | sensory-motor areas | mrna levels | 2 | 28 | 14 | 0.044 | -1.827, 1.914 | 0.96354758 | -3.037, 3.124 | 85.61 | 1.56 | 0.0084 |
| haloperidol | acute | psd95 | ventral striatum | mrna levels | 3 | 33 | 19 | -0.221 | -1.842, 1.399 | 0.78885952 | -3.254, 2.811 | 83.82 | 1.71 | 0.0037 |
| haloperidol | chronic | ania3 | associative cortex | mrna levels | 2 | 12 | 12 | -0.453 | -1.266, 0.36 | 0.275061 | -1.266, 0.36 | 0 | 0 | 0.5593 |
| haloperidol | chronic | ania3 | cingulate cortex | mrna levels | 2 | 11 | 11 | 0.825 | -0.053, 1.704 | 0.065472 | -0.053, 1.704 | 0 | 0 | 0.3797 |
| haloperidol | chronic | ania3 | dorsal striatum | mrna levels | 3 | 16 | 16 | 2.942 | 1.934, 3.951 | 1.0805E-08 | 1.934, 3.951 | 0 | 0 | 0.5769 |
| haloperidol | chronic | ania3 | frontal cortex | mrna levels | 2 | 12 | 12 | -0.59 | -1.41, 0.23 | 0.1586649 | -1.41, 0.23 | 0 | 0 | 0.5667 |
| haloperidol | chronic | ania3 | ventral striatum | mrna levels | 2 | 11 | 11 | 1.124 | 0.225, 2.024 | 0.01428023 | 0.225, 2.024 | 0 | 0 | 0.9443 |
| haloperidol | chronic | arc | cingulate cortex | mrna levels | 3 | 27 | 17 | -0.706 | -1.892, 0.48 | 0.24339115 | -2.782, 1.37 | 68.75 | 0.76 | 0.0413 |
| haloperidol | chronic | arc | dorsal striatum | mrna levels | 4 | 41 | 31 | 0.636 | -0.88, 2.151 | 0.41107803 | -2.551, 3.823 | 86.73 | 2.05 | 0 |
| haloperidol | chronic | arc | limbic system | mrna levels | 3 | 27 | 17 | -0.62 | -1.697, 0.457 | 0.2589732 | -2.449, 1.209 | 62.88 | 0.57 | 0.0679 |
| haloperidol | chronic | arc | sensory-motor areas | mrna levels | 3 | 27 | 17 | -1.186 | -2.82, 0.448 | 0.15500049 | -4.22, 1.848 | 81.71 | 1.7 | 0.0065 |
| haloperidol | chronic | arc | ventral striatum | mrna levels | 3 | 27 | 17 | 0.451 | -0.185, 1.087 | 0.16483476 | -0.185, 1.087 | 0 | 0 | 0.8622 |
| haloperidol | chronic | glua1 | dorsal striatum | mrna levels | 3 | 19 | 19 | 0.653 | -0.582, 1.888 | 0.30028948 | -1.497, 2.803 | 68.38 | 0.81 | 0.0599 |
| haloperidol | chronic | glua1 | frontal cortex | mrna levels | 2 | 13 | 13 | 0.03 | -0.74, 0.799 | 0.93986102 | -0.74, 0.799 | 0 | 0 | 0.7232 |
| haloperidol | chronic | glua1 | subcortical areas | protein levels | 2 | 14 | 14 | -0.246 | -0.99, 0.498 | 0.51739157 | -0.99, 0.498 | 0 | 0 | 0.7722 |
| haloperidol | chronic | glua1 | ventral striatum | mrna levels | 2 | 13 | 13 | 1.476 | -1.117, 4.069 | 0.26466368 | -2.79, 5.742 | 84.74 | 2.99 | 0.0105 |
| haloperidol | chronic | glua2 | dorsal striatum | mrna levels | 3 | 19 | 19 | 1.198 | -1.442, 3.838 | 0.37376296 | -3.864, 6.261 | 91.54 | 4.86 | 0.0012 |
| haloperidol | chronic | glua2 | frontal cortex | mrna levels | 3 | 19 | 19 | -0.401 | -1.182, 0.38 | 0.31473858 | -1.471, 0.67 | 29.24 | 0.14 | 0.2514 |
| haloperidol | chronic | glua2 | ventral striatum | mrna levels | 3 | 19 | 19 | 0.455 | -1.658, 2.568 | 0.67281193 | -3.567, 4.477 | 87.95 | 3.05 | 0.0003 |
| haloperidol | chronic | homer1a | associative cortex | mrna levels | 2 | 12 | 12 | -0.916 | -1.758, -0.073 | 0.03312873 | -1.758, -0.073 | 0 | 0 | 0.6848 |
| haloperidol | chronic | homer1a | cingulate cortex | mrna levels | 9 | 81 | 61 | 0.139 | -0.69, 0.967 | 0.74260251 | -2.192, 2.47 | 79.55 | 1.24 | 0.0001 |
| haloperidol | chronic | homer1a | dorsal striatum | mrna levels | 10 | 86 | 66 | 1.692 | 1.146, 2.238 | 1.2267E-09 | 0.413, 2.972 | 46.47 | 0.35 | 0.0581 |
| haloperidol | chronic | homer1a | frontal cortex | mrna levels | 2 | 12 | 12 | -0.74 | -1.568, 0.088 | 0.07992892 | -1.568, 0.088 | 0 | 0 | 0.7272 |
| haloperidol | chronic | homer1a | limbic system | mrna levels | 8 | 74 | 54 | 0.145 | -0.738, 1.027 | 0.74798984 | -2.246, 2.535 | 79.91 | 1.28 | 0 |
| haloperidol | chronic | homer1a | sensory-motor areas | mrna levels | 8 | 74 | 54 | 0.296 | -0.745, 1.338 | 0.57722923 | -2.579, 3.171 | 84.78 | 1.87 | 0 |
| haloperidol | chronic | homer1a | ventral striatum | mrna levels | 9 | 81 | 61 | 1.182 | 0.771, 1.593 | 1.7094E-08 | 0.547, 1.817 | 15.48 | 0.06 | 0.342 |
| haloperidol | chronic | homer1a | ventral striatum | protein levels | 2 | 12 | 12 | 0.501 | -0.315, 1.317 | 0.22904963 | -0.315, 1.317 | 0 | 0 | 0.5335 |
| haloperidol | chronic | homer1b-c | cingulate cortex | mrna levels | 6 | 52 | 42 | 0.025 | -0.406, 0.456 | 0.90882645 | -0.406, 0.456 | 0 | 0 | 0.023 |
| haloperidol | chronic | homer1b-c | dorsal striatum | mrna levels | 6 | 52 | 42 | 0.418 | -0.213, 1.049 | 0.19438711 | -0.833, 1.668 | 50.07 | 0.3 | 0.0694 |
| haloperidol | chronic | homer1b-c | limbic system | mrna levels | 6 | 52 | 42 | 0.517 | -0.294, 1.329 | 0.21142841 | -1.294, 2.329 | 68.43 | 0.68 | 0.0063 |
| haloperidol | chronic | homer1b-c | sensory-motor areas | mrna levels | 6 | 52 | 42 | 0.117 | -0.309, 0.543 | 0.59035646 | -0.309, 0.543 | 0 | 0 | 0.1156 |
| haloperidol | chronic | homer1b-c | ventral striatum | mrna levels | 6 | 52 | 42 | 0.684 | 0.034, 1.334 | 0.03906996 | -0.623, 1.991 | 51.75 | 0.33 | 0.059 |
| haloperidol | chronic | homer1b-c | ventral striatum | protein levels | 2 | 12 | 12 | -0.13 | -1.579, 1.318 | 0.85999021 | -2.349, 2.088 | 67.33 | 0.74 | 0.0802 |
| haloperidol | chronic | mglur1 | frontal cortex | mrna levels | 2 | 10 | 10 | 0.083 | -0.796, 0.962 | 0.85313923 | -0.796, 0.962 | 0 | 0 | 0.6545 |
| haloperidol | chronic | mglur1 | ventral striatum | protein levels | 2 | 12 | 12 | -0.518 | -1.333, 0.296 | 0.21198665 | -1.333, 0.296 | 0 | 0 | 0.7791 |
| haloperidol | chronic | mglur2 | frontal cortex | mrna levels | 3 | 15 | 15 | -0.289 | -1.013, 0.436 | 0.43491399 | -1.013, 0.436 | 0 | 0 | 0.6678 |
| haloperidol | chronic | mglur5 | frontal cortex | mrna levels | 3 | 15 | 15 | 0.031 | -0.692, 0.754 | 0.93287782 | -0.692, 0.754 | 0 | 0 | 0.5543 |
| haloperidol | chronic | mglur5 | frontal cortex | protein levels | 2 | 12 | 12 | 0.103 | -0.698, 0.904 | 0.80090662 | -0.698, 0.904 | 0 | 0 | 0.7728 |
| haloperidol | chronic | mglur5 | ventral striatum | protein levels | 2 | 12 | 12 | 0.138 | -0.668, 0.945 | 0.73677055 | -0.668, 0.945 | 0 | 0 | 0.4317 |
| haloperidol | chronic | norbin | frontal cortex | protein levels | 2 | 12 | 12 | -0.469 | -2.552, 1.614 | 0.65892406 | -3.863, 2.925 | 82.72 | 1.87 | 0.0161 |
| haloperidol | chronic | norbin | ventral striatum | protein levels | 2 | 12 | 12 | 1.004 | -0.129, 2.137 | 0.0823441 | -0.531, 2.539 | 41.51 | 0.28 | 0.191 |
| haloperidol | chronic | nr1 | dorsal striatum | mrna levels | 5 | 30 | 30 | 1.586 | 0.487, 2.684 | 0.00466178 | -0.708, 3.879 | 68.57 | 1.06 | 0.0153 |
| haloperidol | chronic | nr1 | dorsal striatum | protein levels | 3 | 23 | 23 | 0.666 | -0.379, 1.711 | 0.2115891 | -1.113, 2.445 | 63.49 | 0.54 | 0.0577 |
| haloperidol | chronic | nr1 | frontal cortex | mrna levels | 4 | 32 | 32 | 0.205 | -0.289, 0.7 | 0.4152762 | -0.289, 0.7 | 0 | 0 | 0.6654 |
| haloperidol | chronic | nr1 | frontal cortex | protein levels | 4 | 29 | 29 | -0.142 | -0.983, 0.7 | 0.74175033 | -1.661, 1.378 | 57.09 | 0.42 | 0.0795 |
| haloperidol | chronic | nr1 | hippocampus | mrna levels | 2 | 16 | 16 | 0.477 | -0.653, 1.606 | 0.40797822 | -1.187, 2.141 | 58.14 | 0.39 | 0.1222 |
| haloperidol | chronic | nr1 | hippocampus | protein levels | 4 | 29 | 29 | -0.264 | -0.786, 0.259 | 0.32257201 | -0.786, 0.259 | 0 | 0 | 0.4909 |
| haloperidol | chronic | nr1 | subcortical areas | protein levels | 2 | 14 | 14 | 0.092 | -0.65, 0.833 | 0.8087153 | -0.65, 0.833 | 0 | 0 | 0.8083 |
| haloperidol | chronic | nr1 | ventral striatum | mrna levels | 4 | 25 | 25 | 0.782 | -0.364, 1.927 | 0.18108743 | -1.433, 2.996 | 70.19 | 0.93 | 0.0245 |
| haloperidol | chronic | nr1 | ventral striatum | protein levels | 3 | 23 | 23 | 0.357 | -0.642, 1.357 | 0.48376684 | -1.315, 2.029 | 60.1 | 0.47 | 0.084 |
| haloperidol | chronic | nr2a | dorsal striatum | mrna levels | 2 | 15 | 15 | 0.148 | -2.97, 3.267 | 0.92566932 | -5.113, 5.41 | 92.28 | 4.67 | 0.0003 |
| haloperidol | chronic | nr2a | dorsal striatum | protein levels | 2 | 11 | 11 | 0.42 | -0.425, 1.265 | 0.32985018 | -0.425, 1.265 | 0 | 0 | 0.899 |
| haloperidol | chronic | nr2a | frontal cortex | mrna levels | 2 | 21 | 21 | -0.544 | -1.16, 0.072 | 0.08333199 | -1.16, 0.072 | 0 | 0 | 0.8752 |
| haloperidol | chronic | nr2a | frontal cortex | protein levels | 3 | 17 | 17 | -0.95 | -1.828, -0.072 | 0.03386986 | -2.176, 0.275 | 31.61 | 0.19 | 0.2228 |
| haloperidol | chronic | nr2a | hippocampus | mrna levels | 2 | 21 | 21 | -0.443 | -1.056, 0.169 | 0.15594529 | -1.056, 0.169 | 0 | 0 | 0.8803 |
| haloperidol | chronic | nr2a | hippocampus | protein levels | 3 | 17 | 17 | -0.551 | -1.33, 0.228 | 0.16574759 | -1.527, 0.426 | 18.89 | 0.09 | 0.2559 |
| haloperidol | chronic | nr2a | ventral striatum | protein levels | 2 | 11 | 11 | 0.086 | -0.755, 0.927 | 0.84077202 | -0.755, 0.927 | 0 | 0 | 0.4828 |
| haloperidol | chronic | nr2b | dorsal striatum | mrna levels | 2 | 15 | 15 | 1.346 | -0.218, 2.909 | 0.09155766 | -1.093, 3.785 | 71.56 | 0.91 | 0.0608 |
| haloperidol | chronic | nr2b | frontal cortex | protein levels | 2 | 11 | 11 | 0.35 | -0.936, 1.635 | 0.5939771 | -1.508, 2.207 | 54.2 | 0.47 | 0.1395 |
| haloperidol | chronic | nr2b | hippocampus | protein levels | 3 | 17 | 17 | -1.031 | -2.132, 0.069 | 0.06625326 | -2.817, 0.754 | 54.49 | 0.51 | 0.1101 |
| haloperidol | chronic | preso1 | frontal cortex | protein levels | 2 | 12 | 12 | 0.041 | -0.76, 0.842 | 0.91960294 | -0.76, 0.842 | 0 | 0 | 0.73 |
| haloperidol | chronic | psd95 | cingulate cortex | mrna levels | 5 | 45 | 35 | 0.251 | -1.452, 1.954 | 0.7726198 | -3.719, 4.221 | 90.03 | 3.35 | 0 |
| haloperidol | chronic | psd95 | dorsal striatum | mrna levels | 5 | 45 | 35 | 0.351 | -0.821, 1.524 | 0.55668069 | -2.261, 2.964 | 81.2 | 1.42 | 0.0002 |
| haloperidol | chronic | psd95 | frontal cortex | protein levels | 3 | 16 | 16 | -0.25 | -0.947, 0.446 | 0.48087516 | -0.947, 0.446 | 0 | 0 | 0.9381 |
| haloperidol | chronic | psd95 | limbic system | mrna levels | 5 | 45 | 35 | -0.301 | -1.306, 0.704 | 0.55702181 | -2.478, 1.876 | 75.36 | 0.97 | 0.0016 |
| haloperidol | chronic | psd95 | sensory-motor areas | mrna levels | 5 | 45 | 35 | 0.09 | -1.156, 1.337 | 0.88692017 | -2.723, 2.904 | 83.33 | 1.66 | 0.0001 |
| haloperidol | chronic | psd95 | ventral striatum | mrna levels | 5 | 45 | 35 | 0.162 | -0.739, 1.064 | 0.72411068 | -1.746, 2.071 | 70.57 | 0.74 | 0.0037 |
| haloperidol | chronic | tamalin | frontal cortex | protein levels | 2 | 12 | 12 | 0.135 | -0.666, 0.936 | 0.74079611 | -0.666, 0.936 | 0 | 0 | 0.9777 |
| haloperidol decanoate | chronic | glua2 | dorsal striatum | mrna levels | 2 | 13 | 13 | 0.973 | -0.106, 2.052 | 0.07706322 | -0.482, 2.429 | 40.37 | 0.25 | 0.1953 |
| olanzapine | acute | arc | dorsal striatum | mrna levels | 3 | 48 | 48 | 1.41 | 0.374, 2.446 | 0.00766289 | -0.482, 3.302 | 78.54 | 0.65 | 0.0162 |
| olanzapine | acute | arc | frontal cortex | mrna levels | 2 | 41 | 41 | -2.154 | -2.699, -1.61 | 8.71E-15 | -2.699, -1.61 | 0 | 0 | 0.737 |
| olanzapine | acute | arc | ventral striatum | mrna levels | 2 | 27 | 27 | 1.486 | 0.882, 2.091 | 1.4558E-06 | 0.882, 2.091 | 0 | 0 | 0.387 |
| olanzapine | acute | homer1a | cingulate cortex | mrna levels | 2 | 12 | 12 | 0.701 | -0.967, 2.368 | 0.41011729 | -1.918, 3.32 | 73.34 | 1.06 | 0.0528 |
| olanzapine | acute | homer1a | dorsal striatum | mrna levels | 4 | 22 | 22 | 3.442 | -1.378, 8.261 | 0.16162121 | -6.974, 13.857 | 97.2 | 22.19 | 0.0001 |
| olanzapine | acute | homer1a | limbic system | mrna levels | 2 | 12 | 12 | 0.947 | -0.16, 2.053 | 0.09368945 | -0.535, 2.428 | 39.54 | 0.25 | 0.1984 |
| olanzapine | acute | homer1a | sensory-motor areas | mrna levels | 2 | 12 | 12 | 0.641 | -0.674, 1.957 | 0.33920261 | -1.298, 2.581 | 58.66 | 0.53 | 0.1199 |
| olanzapine | acute | homer1a | ventral striatum | mrna levels | 4 | 22 | 22 | 1.845 | 0.27, 3.419 | 0.02164256 | -1.318, 5.007 | 77.74 | 1.96 | 0.0086 |
| olanzapine | acute | psd95 | dorsal striatum | mrna levels | 2 | 12 | 12 | 2.066 | -0.591, 4.723 | 0.12756396 | -2.294, 6.426 | 84.44 | 3.11 | 0.0113 |
| olanzapine | acute | psd95 | frontal cortex | protein levels | 3 | 18 | 18 | 0.436 | -0.257, 1.128 | 0.21788295 | -0.319, 1.19 | 6.19 | 0.02 | 0.3472 |
| olanzapine | acute | psd95 | hippocampus | protein levels | 3 | 18 | 18 | -0.099 | -0.759, 0.56 | 0.76842823 | -0.759, 0.56 | 0 | 0 | 0.5372 |
| olanzapine | acute | psd95 | ventral striatum | mrna levels | 2 | 12 | 12 | 1.438 | -1.025, 3.9 | 0.25247242 | -2.608, 5.483 | 84.92 | 2.68 | 0.01 |
| olanzapine | chronic | arc | dorsal striatum | mrna levels | 2 | 19 | 19 | -1.597 | -2.328, -0.867 | 1.8322E-05 | -2.328, -0.867 | 0 | 0 | 0.7685 |
| olanzapine | chronic | homer1a | cingulate cortex | mrna levels | 2 | 10 | 10 | 0.2 | -0.686, 1.085 | 0.65825126 | -0.686, 1.085 | 0 | 0 | 0.4347 |
| olanzapine | chronic | homer1a | dorsal striatum | mrna levels | 2 | 10 | 10 | 0.342 | -0.631, 1.314 | 0.49105315 | -0.767, 1.45 | 15 | 0.07 | 0.2781 |
| olanzapine | chronic | homer1a | limbic system | mrna levels | 2 | 10 | 10 | -0.245 | -1.127, 0.636 | 0.58547176 | -1.127, 0.636 | 0 | 0 | 0.6992 |
| olanzapine | chronic | homer1a | sensory-motor areas | mrna levels | 2 | 10 | 10 | 0.322 | -0.789, 1.432 | 0.57038596 | -1.121, 1.764 | 34.37 | 0.22 | 0.2171 |
| olanzapine | chronic | homer1a | ventral striatum | mrna levels | 2 | 10 | 10 | 1.185 | -1.506, 3.875 | 0.38814612 | -3.237, 5.606 | 84.85 | 3.2 | 0.0102 |
| olanzapine | chronic | mglur5 | frontal cortex | protein levels | 2 | 12 | 12 | 0.222 | -0.581, 1.025 | 0.58811116 | -0.581, 1.025 | 0 | 0 | 0.7776 |
| olanzapine | chronic | norbin | frontal cortex | protein levels | 2 | 12 | 12 | -0.039 | -0.84, 0.763 | 0.92462699 | -0.84, 0.763 | 0 | 0 | 0.6702 |
| olanzapine | chronic | nr1 | frontal cortex | protein levels | 2 | 10 | 10 | 0.005 | -0.894, 0.903 | 0.99213327 | -0.913, 0.922 | 2.08 | 0.01 | 0.3122 |
| olanzapine | chronic | nr1 | hippocampus | protein levels | 2 | 12 | 12 | -0.569 | -1.439, 0.3 | 0.19917456 | -1.52, 0.381 | 9.73 | 0.04 | 0.2926 |
| olanzapine | chronic | nr2a | hippocampus | protein levels | 2 | 12 | 12 | -0.284 | -1.094, 0.525 | 0.49146464 | -1.094, 0.525 | 0 | 0 | 0.4211 |
| olanzapine | chronic | nr2b | hippocampus | protein levels | 2 | 12 | 12 | -0.697 | -1.62, 0.225 | 0.13857461 | -1.774, 0.38 | 18.06 | 0.08 | 0.2693 |
| olanzapine | chronic | preso1 | frontal cortex | protein levels | 2 | 12 | 12 | 0.065 | -0.736, 0.865 | 0.87449228 | -0.736, 0.865 | 0 | 0 | 0.8204 |
| olanzapine | chronic | psd95 | frontal cortex | protein levels | 3 | 16 | 16 | -0.095 | -1.294, 1.103 | 0.87593782 | -2.136, 1.945 | 63.3 | 0.71 | 0.0667 |
| olanzapine | chronic | tamalin | frontal cortex | protein levels | 2 | 12 | 12 | 0.399 | -0.413, 1.212 | 0.33558894 | -0.413, 1.212 | 0 | 0 | 0.4663 |
| quetiapine | acute | homer1a | dorsal striatum | mrna levels | 2 | 24 | 12 | 0.436 | -0.264, 1.136 | 0.22239809 | -0.264, 1.136 | 0 | 0 | 0.9673 |
| quetiapine | chronic | homer1a | dorsal striatum | mrna levels | 2 | 19 | 12 | 0.648 | -0.822, 2.119 | 0.38764893 | -1.624, 2.921 | 68.25 | 0.78 | 0.076 |

Appendix 7

## Meta-regressions

| **Meta-regressions** | | | | | | | | | |
| --- | --- | --- | --- | --- | --- | --- | --- | --- | --- |
| **drug** | **model** | **psd type** | **region** | **outcome type** | **n_studies** | **Beta** | **CI** | **p_val** | **predictor** |
| haloperidol | acute | arc | dorsal striatum | mrna levels | 10 | -0.084 | -0.963, 0.796 | 0.852 | animal model |
| haloperidol | acute | arc | dorsal striatum | mrna levels | 10 | 0.447 | -0.53, 1.423 | 0.37 | animal type |
| haloperidol | acute | arc | dorsal striatum | mrna levels | 10 | 3.085 | 0.38, 5.791 | 0.025 | drug dose |
| haloperidol | acute | arc | dorsal striatum | mrna levels | 10 | 0.22 | -1.245, 1.686 | 0.768 | drug injection site |
| haloperidol | acute | arc | dorsal striatum | mrna levels | 10 | -0.411 | -1.021, 0.198 | 0.186 | lab technique |
| haloperidol | acute | homer1a | cingulate cortex | mrna levels | 11 | -0.121 | -0.415, 0.172 | 0.418 | animal model |
| haloperidol | acute | homer1a | cingulate cortex | mrna levels | 11 | 0.506 | -3.412, 4.425 | 0.8 | drug dose |
| haloperidol | acute | homer1a | cingulate cortex | mrna levels | 11 | -0.99 | -1.868, -0.111 | 0.027 | drug injection site |
| haloperidol | acute | homer1a | dorsal striatum | mrna levels | 15 | -0.476 | -0.886, -0.067 | 0.023 | animal model |
| haloperidol | acute | homer1a | dorsal striatum | mrna levels | 15 | 2.222 | -3.657, 8.101 | 0.459 | drug dose |
| haloperidol | acute | homer1a | dorsal striatum | mrna levels | 15 | 0.534 | -0.991, 2.058 | 0.493 | drug injection site |
| haloperidol | acute | homer1a | dorsal striatum | mrna levels | 15 | 0.421 | -1.501, 2.343 | 0.668 | time from last injection |
| haloperidol | acute | homer1a | ventral striatum | mrna levels | 14 | -0.459 | -0.867, -0.051 | 0.027 | animal model |
| haloperidol | acute | homer1a | ventral striatum | mrna levels | 14 | 0.877 | -4.861, 6.616 | 0.764 | drug dose |
| haloperidol | acute | homer1a | ventral striatum | mrna levels | 14 | 0.594 | -0.879, 2.067 | 0.429 | drug injection site |
| haloperidol | acute | homer1a | ventral striatum | mrna levels | 14 | 0.127 | -1.706, 1.96 | 0.892 | time from last injection |
| haloperidol | chronic | homer1a | dorsal striatum | mrna levels | 10 | -0.026 | -0.217, 0.165 | 0.791 | animal model |
| haloperidol | chronic | homer1a | dorsal striatum | mrna levels | 10 | 5.103 | 2.045, 8.162 | 0.001 | drug dose |
| haloperidol | chronic | homer1a | dorsal striatum | mrna levels | 10 | 0.159 | -0.931, 1.249 | 0.775 | drug injection site |
| haloperidol | chronic | homer1a | dorsal striatum | mrna levels | 10 | 0.004 | -0.085, 0.093 | 0.924 | number of administration |

# References

1. Ambesi-Impiombato A, Panariello F, Dell'aversano C, Tomasetti C, Muscettola G, de Bartolomeis A. Differential expression of Homer 1 gene by acute and chronic administration of antipsychotics and dopamine transporter inhibitors in the rat forebrain. *Synapse* 2007; **61**(6)**:** 429-439.

2. Barone A, Signoriello S, Latte G, Vellucci L, Giordano G, Avagliano C *et al.* Modulation of glutamatergic functional connectivity by a prototypical antipsychotic: Translational inference from a postsynaptic density immediate-early gene-based network analysis. *Behav Brain Res* 2021; **404:** 113160.

3. Barone A, De Simone G, Ciccarelli M, Buonaguro EF, Tomasetti C, Eramo A *et al.* A Postsynaptic Density Immediate Early Gene-Based Connectome Analysis of Acute NMDAR Blockade and Reversal Effect of Antipsychotic Administration. *Int J Mol Sci* 2023; **24**(5).

4. Baturina M, Beyer E, Baturin V, Popov A. Dependence of the severity of haloperidol catalepsy on the activity of dopaminergic and glutamatergic systems of the brain of rats with prolonged use of antipsychotics. *Medical news of the North Caucasus* 2020; **15**.

5. Brené S, Messer C, Nestler EJ. Expression of messenger RNAs encoding ionotropic glutamate receptors in rat brain: regulation by haloperidol. *Neuroscience* 1998; **84**(3)**:** 813-823.

6. Bruins Slot LA, Lestienne F, Grevoz-Barret C, Newman-Tancredi A, Cussac D. F15063, a potential antipsychotic with dopamine D(2)/D(3) receptor antagonist and 5-HT(1A) receptor agonist properties: influence on immediate-early gene expression in rat prefrontal cortex and striatum. *Eur J Pharmacol* 2009; **620**(1-3)**:** 27-35.

7. Buck T, Dong E, McCarthy M, Guidotti A, Sodhi M. Prenatal stress alters transcription of NMDA-type glutamate receptors in the hippocampus. *Neurosci Lett* 2024; **836:** 137886.

8. Buonaguro EF, Iasevoli F, Marmo F, Eramo A, Latte G, Avagliano C *et al.* Re-arrangements of gene transcripts at glutamatergic synapses after prolonged treatments with antipsychotics: A putative link with synaptic remodeling. *Prog Neuropsychopharmacol Biol Psychiatry* 2017; **76:** 29-41.

9. Buonaguro EF, Tomasetti C, Chiodini P, Marmo F, Latte G, Rossi R *et al.* Postsynaptic density protein transcripts are differentially modulated by minocycline alone or in add-on to haloperidol: Implications for treatment resistant schizophrenia. *J Psychopharmacol* 2017; **31**(4)**:** 406-417.

10. Chen AC, McDonald B, Moss SJ, Gurling HM. Gene expression studies of mRNAs encoding the NMDA receptor subunits NMDAR1, NMDAR2A, NMDAR2B, NMDAR2C, and NMDAR2D following long-term treatment with cis-and trans-flupenthixol as a model for understanding the mode of action of schizophrenia drug treatment. *Brain Res Mol Brain Res* 1998; **54**(1)**:** 92-100.

11. Chen AC, Gurling HM. D2 dopamine receptor but not AMPA and kainate glutamate receptor genes show altered expression in response to long term treatment with trans- and cis-flupenthixol in the rat brain. *Brain Res Mol Brain Res* 1999; **68**(1-2)**:** 14-21.

12. Chiba S, Hashimoto R, Hattori S, Yohda M, Lipska B, Weinberger DR *et al.* Effect of antipsychotic drugs on DISC1 and dysbindin expression in mouse frontal cortex and hippocampus. *J Neural Transm (Vienna)* 2006; **113**(9)**:** 1337-1346.

13. Choi YK, Gardner MP, Tarazi FI. Effects of risperidone on glutamate receptor subtypes in developing rat brain. *Eur Neuropsychopharmacol* 2009; **19**(2)**:** 77-84.

14. Choi YK, Adham N, Kiss B, Gyertyán I, Tarazi FI. Long-term effects of aripiprazole exposure on monoaminergic and glutamatergic receptor subtypes: comparison with cariprazine. *CNS Spectr* 2017; **22**(6)**:** 484-494.

15. Collins CM, Wood MD, Elliott JM. Chronic administration of haloperidol and clozapine induces differential effects on the expression of Arc and c-Fos in rat brain. *J Psychopharmacol* 2014; **28**(10)**:** 947-954.

16. de Bartolomeis A, Aloj L, Ambesi-Impiombato A, Bravi D, Caracò C, Muscettola G *et al.* Acute administration of antipsychotics modulates Homer striatal gene expression differentially. *Brain Res Mol Brain Res* 2002; **98**(1-2)**:** 124-129.

17. de Bartolomeis A, Marmo F, Buonaguro EF, Rossi R, Tomasetti C, Iasevoli F. Imaging brain gene expression profiles by antipsychotics: region-specific action of amisulpride on postsynaptic density transcripts compared to haloperidol. *Eur Neuropsychopharmacol* 2013; **23**(11)**:** 1516-1529.

18. de Bartolomeis A, Iasevoli F, Marmo F, Buonaguro EF, Eramo A, Rossi R *et al.* Progressive recruitment of cortical and striatal regions by inducible postsynaptic density transcripts after increasing doses of antipsychotics with different receptor profiles: insights for psychosis treatment. *Eur Neuropsychopharmacol* 2015; **25**(4)**:** 566-582.

19. de Bartolomeis A, Marmo F, Buonaguro EF, Latte G, Tomasetti C, Iasevoli F. Switching antipsychotics: Imaging the differential effect on the topography of postsynaptic density transcripts in antipsychotic-naïve vs. antipsychotic-exposed rats. *Prog Neuropsychopharmacol Biol Psychiatry* 2016; **70:** 24-38.

20. de Bartolomeis A, Iasevoli F, Marmo F, Buonaguro EF, Avvisati L, Latte G *et al.* Nicotine and caffeine modulate haloperidol-induced changes in postsynaptic density transcripts expression: Translational insights in psychosis therapy and treatment resistance. *Eur Neuropsychopharmacol* 2018; **28**(4)**:** 538-559.

21. Dell'aversano C, Tomasetti C, Iasevoli F, de Bartolomeis A. Antipsychotic and antidepressant co-treatment: effects on transcripts of inducible postsynaptic density genes possibly implicated in behavioural disorders. *Brain Res Bull* 2009; **79**(2)**:** 123-129.

22. Dutra-Tavares AC, Souza TP, Silva JO, Semeão KA, Mello FF, Filgueiras CC *et al.* Neonatal phencyclidine as a model of sex-biased schizophrenia symptomatology in adolescent mice. *Psychopharmacology (Berl)* 2023; **240**(10)**:** 2111-2129.

23. Eastwood SL, Story P, Burnet PW, Heath P, Harrison PJ. Differential changes in glutamate receptor subunit messenger RNAs in rat brain after haloperidol treatment. *J Psychopharmacol* 1994; **8**(4)**:** 196-203.

24. Eastwood SL, Porter RH, Harrison PJ. The effect of chronic haloperidol treatment on glutamate receptor subunit (GluR1, GluR2, KA1, KA2, NR1) mRNAs and glutamate binding protein mRNA in rat forebrain. *Neurosci Lett* 1996; **212**(3)**:** 163-166.

25. Fatemi SH, Reutiman TJ, Folsom TD, Bell C, Nos L, Fried P *et al.* Chronic olanzapine treatment causes differential expression of genes in frontal cortex of rats as revealed by DNA microarray technique. *Neuropsychopharmacology* 2006; **31**(9)**:** 1888-1899.

26. Fehér LZ, Kálmán J, Puskás LG, Gyülvészi G, Kitajka K, Penke B *et al.* Impact of haloperidol and risperidone on gene expression profile in the rat cortex. *Neurochem Int* 2005; **47**(4)**:** 271-280.

27. Fitzgerald LW, Deutch AY, Gasic G, Heinemann SF, Nestler EJ. Regulation of cortical and subcortical glutamate receptor subunit expression by antipsychotic drugs. *J Neurosci* 1995; **15**(3 Pt 2)**:** 2453-2461.

28. Fitzgerald LW, Ortiz J, Hamedani AG, Nestler EJ. Drugs of abuse and stress increase the expression of GluR1 and NMDAR1 glutamate receptor subunits in the rat ventral tegmental area: common adaptations among cross-sensitizing agents. *J Neurosci* 1996; **16**(1)**:** 274-282.

29. Fumagalli F, Frasca A, Racagni G, Riva MA. Dynamic regulation of glutamatergic postsynaptic activity in rat prefrontal cortex by repeated administration of antipsychotic drugs. *Mol Pharmacol* 2008; **73**(5)**:** 1484-1490.

30. Fumagalli F, Frasca A, Racagni G, Riva MA. Antipsychotic drugs modulate Arc expression in the rat brain. *Eur Neuropsychopharmacol* 2009; **19**(2)**:** 109-115.

31. Funk AJ, Mielnik CA, Koene R, Newburn E, Ramsey AJ, Lipska BK *et al.* Postsynaptic Density-95 Isoform Abnormalities in Schizophrenia. *Schizophr Bull* 2017; **43**(4)**:** 891-899.

32. Girgenti MJ, Nisenbaum LK, Bymaster F, Terwilliger R, Duman RS, Newton SS. Antipsychotic-induced gene regulation in multiple brain regions. *J Neurochem* 2010; **113**(1)**:** 175-187.

33. Gomes FV, Issy AC, Ferreira FR, Viveros MP, Del Bel EA, Guimarães FS. Cannabidiol attenuates sensorimotor gating disruption and molecular changes induced by chronic antagonism of NMDA receptors in mice. *Int J Neuropsychopharmacol* 2014; **18**(5).

34. Håkansson K, Galdi S, Hendrick J, Snyder G, Greengard P, Fisone G. Regulation of phosphorylation of the GluR1 AMPA receptor by dopamine D2 receptors. *J Neurochem* 2006; **96**(2)**:** 482-488.

35. Hanaoka T, Toyoda H, Mizuno T, Kikuyama H, Morimoto K, Takahata R *et al.* Alterations in NMDA receptor subunit levels in the brain regions of rats chronically administered typical or atypical antipsychotic drugs. *Neurochem Res* 2003; **28**(6)**:** 919-924.

36. Hattori K, Uchino S, Isosaka T, Maekawa M, Iyo M, Sato T *et al.* Fyn is required for haloperidol-induced catalepsy in mice. *J Biol Chem* 2006; **281**(11)**:** 7129-7135.

37. Healy DJ, Meador-Woodruff JH. Clozapine and haloperidol differentially affect AMPA and kainate receptor subunit mRNA levels in rat cortex and striatum. *Brain Res Mol Brain Res* 1997; **47**(1-2)**:** 331-338.

38. Hida H, Mouri A, Mori K, Matsumoto Y, Seki T, Taniguchi M *et al.* Blonanserin ameliorates phencyclidine-induced visual-recognition memory deficits: the complex mechanism of blonanserin action involving D₃-5-HT₂A and D₁-NMDA receptors in the mPFC. *Neuropsychopharmacology* 2015; **40**(3)**:** 601-613.

39. Hiraoka S, Kajii Y, Kuroda Y, Umino A, Nishikawa T. The development- and phencyclidine-regulated induction of synapse-associated protein-97 gene in the rat neocortex. *Eur Neuropsychopharmacol* 2010; **20**(3)**:** 176-186.

40. Iasevoli F, Fiore G, Cicale M, Muscettola G, de Bartolomeis A. Haloperidol induces higher Homer1a expression than risperidone, olanzapine and sulpiride in striatal sub-regions. *Psychiatry Res* 2010; **177**(1-2)**:** 255-260.

41. Iasevoli F, Tomasetti C, Marmo F, Bravi D, Arnt J, de Bartolomeis A. Divergent acute and chronic modulation of glutamatergic postsynaptic density genes expression by the antipsychotics haloperidol and sertindole. *Psychopharmacology (Berl)* 2010; **212**(3)**:** 329-344.

42. Iasevoli F, Ambesi-Impiombato A, Fiore G, Panariello F, Muscettola G, de Bartolomeis A. Pattern of acute induction of Homer1a gene is preserved after chronic treatment with first- and second-generation antipsychotics: effect of short-term drug discontinuation and comparison with Homer1a-interacting genes. *J Psychopharmacol* 2011; **25**(7)**:** 875-887.

43. Iasevoli F, Buonaguro EF, Sarappa C, Marmo F, Latte G, Rossi R *et al.* Regulation of postsynaptic plasticity genes' expression and topography by sustained dopamine perturbation and modulation by acute memantine: relevance to schizophrenia. *Prog Neuropsychopharmacol Biol Psychiatry* 2014; **54:** 299-314.

44. Iasevoli F, Buonaguro EF, Avagliano C, Barone A, Eramo A, Vellucci L *et al.* The Effects of Antipsychotics on the Synaptic Plasticity Gene Homer1a Depend on a Combination of Their Receptor Profile, Dose, Duration of Treatment, and Brain Regions Targeted. *Int J Mol Sci* 2020; **21**(15).

45. Kao AC, Spitzer S, Anthony DC, Lennox B, Burnet PWJ. Prebiotic attenuation of olanzapine-induced weight gain in rats: analysis of central and peripheral biomarkers and gut microbiota. *Transl Psychiatry* 2018; **8**(1)**:** 66.

46. Korlatowicz A, Kuśmider M, Szlachta M, Pabian P, Solich J, Dziedzicka-Wasylewska M *et al.* Identification of Molecular Markers of Clozapine Action in Ketamine-Induced Cognitive Impairment: A GPCR Signaling PathwayFinder Study. *Int J Mol Sci* 2021; **22**(22).

47. Krzystanek M, Bogus K, Pałasz A, Krzystanek E, Worthington JJ, Wiaderkiewicz R. Effects of long-term treatment with the neuroleptics haloperidol, clozapine and olanzapine on immunoexpression of NMDA receptor subunits NR1, NR2A and NR2B in the rat hippocampus. *Pharmacol Rep* 2015; **67**(5)**:** 965-969.

48. Krzystanek M, Bogus K, Pałasz A, Wiaderkiewicz A, Filipczyk Ł, Rojczyk E *et al.* Extended neuroleptic administration modulates NMDA-R subunit immunoexpression in the rat neocortex and diencephalon. *Pharmacol Rep* 2016; **68**(5)**:** 990-995.

49. Lan Y, Li A, Ding C, Xia J, Zhang X, Luo D. Mechanistic insights into Quetiapine's Protective effects on cognitive function and synaptic plasticity in epileptic rats. *Brain Res* 2025; **1850:** 149426.

50. Law AJ, Hutchinson LJ, Burnet PW, Harrison PJ. Antipsychotics increase microtubule-associated protein 2 mRNA but not spinophilin mRNA in rat hippocampus and cortex. *J Neurosci Res* 2004; **76**(3)**:** 376-382.

51. Lidow MS, Song ZM, Castner SA, Allen PB, Greengard P, Goldman-Rakic PS. Antipsychotic treatment induces alterations in dendrite- and spine-associated proteins in dopamine-rich areas of the primate cerebral cortex. *Biol Psychiatry* 2001; **49**(1)**:** 1-12.

52. Liu X, Feng S, Feng Z, Ma C, He Y, Li X *et al.* Protective Effects of Shi-Zhen-An-Shen Decoction on the Cognitive Impairment in MK801-Induced Schizophrenia Model. *J Integr Neurosci* 2022; **21**(6)**:** 164.

53. Lum JS, Pan B, Deng C, Huang XF, Ooi L, Newell KA. Effects of short- and long-term aripiprazole treatment on Group I mGluRs in the nucleus accumbens: Comparison with haloperidol. *Psychiatry Res* 2018; **260:** 152-157.

54. Luoni A, Fumagalli F, Racagni G, Riva MA. Repeated aripiprazole treatment regulates Bdnf, Arc and Npas4 expression under basal condition as well as after an acute swim stress in the rat brain. *Pharmacol Res* 2014; **80:** 1-8.

55. Luoni A, Rocha FF, Riva MA. Anatomical specificity in the modulation of activity-regulated genes after acute or chronic lurasidone treatment. *Prog Neuropsychopharmacol Biol Psychiatry* 2014; **50:** 94-101.

56. Mahmoud GS, Hosny G, Sayed SA. The protective effect of olanzapine on ketamine induced cognitive deficit and increased NR1 expression in rat model of schizophrenia. *Int J Physiol Pathophysiol Pharmacol* 2021; **13**(2)**:** 22-35.

57. Marchisella F, Paladini MS, Guidi A, Begni V, Brivio P, Spero V *et al.* Chronic treatment with the antipsychotic drug blonanserin modulates the responsiveness to acute stress with anatomical selectivity. *Psychopharmacology (Berl)* 2020; **237**(6)**:** 1783-1793.

58. Matosin N, Fernandez-Enright F, Fung SJ, Lum JS, Engel M, Andrews JL *et al.* Alterations of mGluR5 and its endogenous regulators Norbin, Tamalin and Preso1 in schizophrenia: towards a model of mGluR5 dysregulation. *Acta Neuropathol* 2015; **130**(1)**:** 119-129.

59. Matsumoto Y, Niwa M, Mouri A, Noda Y, Fukushima T, Ozaki N *et al.* Adolescent stress leads to glutamatergic disturbance through dopaminergic abnormalities in the prefrontal cortex of genetically vulnerable mice. *Psychopharmacology (Berl)* 2017; **234**(20)**:** 3055-3074.

60. Mavrikaki M, Schintu N, Kastellakis A, Nomikos GG, Svenningsson P, Panagis G. Effects of lithium and aripiprazole on brain stimulation reward and neuroplasticity markers in the limbic forebrain. *Eur Neuropsychopharmacol* 2014; **24**(4)**:** 630-638.

61. McCullumsmith RE, Stincic TL, Agrawal SM, Meador-Woodruff JH. Differential effects of antipsychotics on haloperidol-induced vacuous chewing movements and subcortical gene expression in the rat. *Eur J Pharmacol* 2003; **477**(2)**:** 101-112.

62. Meshul CK, Bunker GL, Mason JN, Allen C, Janowsky A. Effects of subchronic clozapine and haloperidol on striatal glutamatergic synapses. *J Neurochem* 1996; **67**(5)**:** 1965-1973.

63. Nakahara T, Kuroki T, Hashimoto K, Hondo H, Tsutsumi T, Motomura K *et al.* Effect of atypical antipsychotics on phencyclidine-induced expression of arc in rat brain. *Neuroreport* 2000; **11**(3)**:** 551-555.

64. O'Connor JA, Hasenkamp W, Horman BM, Muly EC, Hemby SE. Region specific regulation of NR1 in rhesus monkeys following chronic antipsychotic drug administration. *Biol Psychiatry* 2006; **60**(6)**:** 659-662.

65. Orsetti M, Di Brisco F, Rinaldi M, Dallorto D, Ghi P. Some molecular effectors of antidepressant action of quetiapine revealed by DNA microarray in the frontal cortex of anhedonic rats. *Pharmacogenet Genomics* 2009; **19**(8)**:** 600-612.

66. Pan B, Huang XF, Deng C. Chronic administration of aripiprazole activates GSK3β-dependent signalling pathways, and up-regulates GABAA receptor expression and CREB1 activity in rats. *Sci Rep* 2016; **6:** 30040.

67. Pei Q, Zetterström TS, Sprakes M, Tordera R, Sharp T. Antidepressant drug treatment induces Arc gene expression in the rat brain. *Neuroscience* 2003; **121**(4)**:** 975-982.

68. Polese D, de Serpis AA, Ambesi-Impiombato A, Muscettola G, de Bartolomeis A. Homer 1a gene expression modulation by antipsychotic drugs: involvement of the glutamate metabotropic system and effects of D-cycloserine. *Neuropsychopharmacology* 2002; **27**(6)**:** 906-913.

69. Riva MA, Tascedda F, Lovati E, Racagni G. Regulation of NMDA receptor subunit messenger RNA levels in the rat brain following acute and chronic exposure to antipsychotic drugs. *Brain Res Mol Brain Res* 1997; **50**(1-2)**:** 136-142.

70. Robbins MJ, Critchlow HM, Lloyd A, Cilia J, Clarke JD, Bond B *et al.* Differential expression of IEG mRNA in rat brain following acute treatment with clozapine or haloperidol: a semi-quantitative RT-PCR study. *J Psychopharmacol* 2008; **22**(5)**:** 536-542.

71. Sakuma K, Komatsu H, Maruyama M, Imaichi S, Habata Y, Mori M. Temporal and spatial transcriptional fingerprints by antipsychotic or propsychotic drugs in mouse brain. *PLoS One* 2015; **10**(2)**:** e0118510.

72. Schmitt A, Zink M, Müller B, May B, Herb A, Jatzko A *et al.* Effects of long-term antipsychotic treatment on NMDA receptor binding and gene expression of subunits. *Neurochem Res* 2003; **28**(2)**:** 235-241.

73. Segnitz N, Ferbert T, Schmitt A, Gass P, Gebicke-Haerter PJ, Zink M. Effects of chronic oral treatment with aripiprazole on the expression of NMDA receptor subunits and binding sites in rat brain. *Psychopharmacology (Berl)* 2011; **217**(1)**:** 127-142.

74. Seo MK, Lee CH, Cho HY, You YS, Lee BJ, Lee JG *et al.* Effects of antipsychotic drugs on the expression of synapse-associated proteins in the frontal cortex of rats subjected to immobilization stress. *Psychiatry Res* 2015; **229**(3)**:** 968-974.

75. Stan TL, Sousa VC, Zhang X, Ono M, Svenningsson P. Lurasidone and fluoxetine reduce novelty-induced hypophagia and NMDA receptor subunit and PSD-95 expression in mouse brain. *Eur Neuropsychopharmacol* 2015; **25**(10)**:** 1714-1722.

76. Tascedda F, Blom JM, Brunello N, Zolin K, Gennarelli M, Colzi A *et al.* Modulation of glutamate receptors in response to the novel antipsychotic olanzapine in rats. *Biol Psychiatry* 2001; **50**(2)**:** 117-122.

77. Tomasetti C, Dell'Aversano C, Iasevoli F, de Bartolomeis A. Homer splice variants modulation within cortico-subcortical regions by dopamine D2 antagonists, a partial agonist, and an indirect agonist: implication for glutamatergic postsynaptic density in antipsychotics action. *Neuroscience* 2007; **150**(1)**:** 144-158.

78. Tomasetti C, Dell'Aversano C, Iasevoli F, Marmo F, de Bartolomeis A. The acute and chronic effects of combined antipsychotic-mood stabilizing treatment on the expression of cortical and striatal postsynaptic density genes. *Prog Neuropsychopharmacol Biol Psychiatry* 2011; **35**(1)**:** 184-197.

79. Waters S, Ponten H, Edling M, Svanberg B, Klamer D, Waters N. The dopaminergic stabilizers pridopidine and ordopidine enhance cortico-striatal Arc gene expression. *J Neural Transm (Vienna)* 2014; **121**(11)**:** 1337-1347.

80. Zhang C, Fang Y, Xu L. Glutamate receptor 1 phosphorylation at serine 845 contributes to the therapeutic effect of olanzapine on schizophrenia-like cognitive impairments. *Schizophr Res* 2014; **159**(2-3)**:** 376-384.

81. Zhou X, Cai G, Mao S, Xu D, Xu X, Zhang R *et al.* Modulating NMDA receptors to treat MK-801-induced schizophrenic cognition deficit: effects of clozapine combining with PQQ treatment and possible mechanisms of action. *BMC Psychiatry* 2020; **20**(1)**:** 106.

82. Abbas AI, Yadav PN, Yao WD, Arbuckle MI, Grant SG, Caron MG *et al.* PSD-95 is essential for hallucinogen and atypical antipsychotic drug actions at serotonin receptors. *J Neurosci* 2009; **29**(22)**:** 7124-7136.

83. Abiero A, Perez Custodio RJ, Botanas CJ, Ortiz DM, Sayson LV, Kim M *et al.* 1-Phenylcyclohexan-1-amine hydrochloride (PCA HCl) alters mesolimbic dopamine system accompanied by neuroplastic changes: A neuropsychopharmacological evaluation in rodents. *Neurochem Int* 2021; **144:** 104962.

84. Adham NB, S. A. Young, J. W. Gyertyán, I. Kiss, B. Markou, A. The effects of the dopamine D2-family receptor partial agonists, cariprazine and aripiprazole, on PCP-induced deficits on attention assessed in the 5-choice serial reaction time task. 2015.

85. Ahn YM, Kang UG, Park JB, Kim YS. Effects of MK-801 and electroconvulsive shock on c-Fos expression in the rat hippocampus and frontal cortex. *Prog Neuropsychopharmacol Biol Psychiatry* 2002; **26**(3)**:** 513-517.

86. Ahn YM, Seo MS, Kim SH, Jeon WJ, Kim Y, Kang UG *et al.* Reduction in the protein level of c-Jun and phosphorylation of Ser73-c-Jun in rat frontal cortex after repeated MK-801 treatment. *Psychiatry Res* 2009; **167**(1-2)**:** 80-87.

87. Ambesi-Impiombato A, D'Urso G, Muscettola G, de Bartolomeis A. Method for quantitative in situ hybridization histochemistry and image analysis applied for Homer1a gene expression in rat brain. *Brain Res Brain Res Protoc* 2003; **11**(3)**:** 189-196.

88. Amin SN, Gamal SM, Esmail RS, Aziz TM, Rashed LA. Cognitive effects of acute restraint stress in male albino rats and the impact of pretreatment with quetiapine versus ghrelin. *J Integr Neurosci* 2014; **13**(4)**:** 669-692.

89. Amiri S, Jafari-Sabet M, Keyhanfar F, Falak R, Shabani M, Rezayof A. Hippocampal and prefrontal cortical NMDA receptors mediate the interactive effects of olanzapine and lithium in memory retention in rats: the involvement of CAMKII-CREB signaling pathways. *Psychopharmacology (Berl)* 2020; **237**(5)**:** 1383-1396.

90. Anastasio NC, Johnson KM. Atypical anti-schizophrenic drugs prevent changes in cortical N-methyl-D-aspartate receptors and behavior following sub-chronic phencyclidine administration in developing rat pups. *Pharmacol Biochem Behav* 2008; **90**(4)**:** 569-577.

91. Apam-Castillejos DJ, Tendilla-Beltrán H, Vázquez-Roque RA, Vázquez-Hernández AJ, Fuentes-Medel E, García-Dolores F *et al.* Second-generation antipsychotic olanzapine attenuates behavioral and prefrontal cortex synaptic plasticity deficits in a neurodevelopmental schizophrenia-related rat model. *J Chem Neuroanat* 2022; **125:** 102166.

92. Camilla A, Buonaguro E, Tomasetti C, Marmo F, Vellucci L, Iasevoli F *et al.* *F234. Typical and Atypical Antipsychotics’ D2r Affinity and Doses Influences Postsynaptic Density by Modulating the Spatial Expression of Homer1a a Gene Highly Implicated in Synaptic Plasticity and Psychosis*. Schizophr Bull. 2018 Apr;44(Suppl 1):S313. doi: 10.1093/schbul/sby017.765. Epub 2018 Apr 1.

93. Avvisati L, Rossi R, Latte G, Buonaguro E, Eramo A, Marmo F *et al.* 2251 – Cortical And Subcortical Gene-expression Imaging By Different n-methyl-daspartate Receptor (nmda-r) Antagonists At Glutammatergic Synapses: Implications For Dopamine-glutamate Interplay In Psychoses. *European Psychiatry* 2013; **28:** 1.

94. Bardgett ME, Griffith MS, Foltz RF, Hopkins JA, Massie CM, O'Connell SM. The effects of clozapine on delayed spatial alternation deficits in rats with hippocampal damage. *Neurobiol Learn Mem* 2006; **85**(1)**:** 86-94.

95. Barr AM, Young CE, Phillips AG, Honer WG. Selective effects of typical antipsychotic drugs on SNAP-25 and synaptophysin in the hippocampal trisynaptic pathway. *Int J Neuropsychopharmacol* 2006; **9**(4)**:** 457-463.

96. Begni V, Sanson A, Grayson B, Neill J, Riva MA. *T109. Modulatory Activity of the Novel Drug Sep-363856 on Brain Function: Potential Application for the Treatment of Schizophrenia*. Schizophr Bull. 2019 Apr;45(Suppl 2):S246. doi: 10.1093/schbul/sbz019.389. Epub 2019 Apr 9.

97. Bektaş E, Ulak G, Mutlu O, Komsuoglu Celikyurt I, Akar F, Erden B. P.1.j.002 Asenapine and paliperidone improve MK-801-induced memory deterioration in Morris water maze test in mice. *European Neuropsychopharmacology* 2014; **24:** S320.

98. Bertran-Gonzalez J, Bosch C, Maroteaux M, Matamales M, Hervé D, Valjent E *et al.* Opposing patterns of signaling activation in dopamine D1 and D2 receptor-expressing striatal neurons in response to cocaine and haloperidol. *J Neurosci* 2008; **28**(22)**:** 5671-5685.

99. Bjarnadottir M, Misner DL, Haverfield-Gross S, Bruun S, Helgason VG, Stefansson H *et al.* Neuregulin1 (NRG1) signaling through Fyn modulates NMDA receptor phosphorylation: differential synaptic function in NRG1+/- knock-outs compared with wild-type mice. *J Neurosci* 2007; **27**(17)**:** 4519-4529.

100. Borrell J. S.03.02 Maternal exposure to bacterial endotoxin during pregnancy in rats: implications for aetiopathogenesis of schizophrenia. *European Neuropsychopharmacology - EUR NEUROPSYCHOPHARMACOL* 2009; **19**.

101. Boulay D, Lacave M, Bergis O, Avenet P, Griebel G. P.3.d.004 Effects of the 5HT2 receptor antagonist, eplivanserin, and the mGluR2/3 agonist, LY404039, in mice models of hyperactivity. *European Neuropsychopharmacology - EUR NEUROPSYCHOPHARMACOL* 2011; **21**.

102. Bragina L, Melone M, Fattorini G, Torres-Ramos M, Vallejo-Illarramendi A, Matute C *et al.* GLT-1 down-regulation induced by clozapine in rat frontal cortex is associated with synaptophysin up-regulation. *J Neurochem* 2006; **99**(1)**:** 134-141.

103. Bristow G, Dong E, Nwabuisi-Heath E, Gentile S, Guidotti A, Sodhi M. *Deficits of Hippocampal RNA Editing and Social Interaction Resulting from Prenatal Stress are Mitigated by Clozapine*, 2021.

104. Bristow GS, M. AMPA receptor RNA editing may alleviate the long-term effects of prenatal stress. 2017.

105. Buonaguro EFM, F. Tomasetti, C. Iasevoli, F. Rossi, R. De Bartolomeis, A. Not all dopamine D2 receptor antagonists were created equal: Potential role of presynaptic versus postsynaptic dopamine D2 receptor blockade in early genes induction by antipsychotics. 2012.

106. Buonaguro E, Marmo F, Sarappa C, Eramo A, Tomasetti C, Iasevoli F *et al.* P.1.g.054 Gene-expression imaging by N-methyl-D-aspartate receptor antagonists: implications for dopamine–glutamate interplay in psychosis. *European Neuropsychopharmacology* 2013; **23:** S220-S221.

107. Buonaguro E, Tomasetti C, Marmo F, Latte G, Avvisati L, Iasevoli F *et al.* *Minocycline add-on to haloperidol: Brain topography of glutamatergic signaling transcripts and implications for psychosis treatment*, 2014, 65-66pp.

108. Buonaguro E, Latte G, Avvisati L, Marmo F, Iasevoli F, Tomasetti C *et al.* P.1.009 Effects of caffeine, nicotine and their combination with haloperidol on PSD molecules: relevance to psychiatric diseases. *European Neuropsychopharmacology* 2016; **26:** S10-S11.

109. Buonaguro E, Iasevoli F, Latte G, Avagliano C, Vellucci L, Marmo F *et al.* Analysis of Homer1a expression levels as a tool to investigate antipsychotic-related perturbation of synaptic plasticity: translational implications for schizophrenia treatment strategies. *European Neuropsychopharmacology* 2016; **26:** S233.

110. Buonanno A, Skirzewski M. *177.2 Mutations in the Neuregulin-ErbB4 Pathway Alter Dopaminergic Balance and Regulate Behaviors Relevant to Schizophrenia*. Schizophr Bull. 2017 Mar;43(Suppl 1):S92-3. doi: 10.1093/schbul/sbx021.249. Epub 2017 Mar 20.

111. Bustos G, Abarca J, Campusano J, Bustos V, Noriega V, Aliaga E. Functional interactions between somatodendritic dopamine release, glutamate receptors and brain-derived neurotrophic factor expression in mesencephalic structures of the brain. *Brain Res Brain Res Rev* 2004; **47**(1-3)**:** 126-144.

112. Castillo-Gómez E, Gómez-Climent MA, Varea E, Guirado R, Blasco-Ibáñez JM, Crespo C *et al.* Dopamine acting through D2 receptors modulates the expression of PSA-NCAM, a molecule related to neuronal structural plasticity, in the medial prefrontal cortex of adult rats. *Exp Neurol* 2008; **214**(1)**:** 97-111.

113. Chartoff EH, Ward RP, Dorsa DM. Role of adenosine and N-methyl-D-aspartate receptors in mediating haloperidol-induced gene expression and catalepsy. *J Pharmacol Exp Ther* 1999; **291**(2)**:** 531-537.

114. Chen Y, Bang S, McMullen MF, Kazi H, Talbot K, Ho MX *et al.* Neuronal Activity-Induced Sterol Regulatory Element Binding Protein-1 (SREBP1) is Disrupted in Dysbindin-Null Mice-Potential Link to Cognitive Impairment in Schizophrenia. *Mol Neurobiol* 2017; **54**(3)**:** 1699-1709.

115. Chen Y-C, Tsai F-M, Chen M-L. Antipsychotic Drugs Reverse MK801-Inhibited Cell Migration and F-actin Condensation by Modulating the Rho Signaling Pathway in B35 Cells. *Behavioural Neurology* 2020; **2020**(1)**:** 4163274.

116. Chhabra S, Nardi L, Leukel P, Sommer CJ, Schmeisser MJ. Striatal increase of dopamine receptor 2 density in idiopathic and syndromic mouse models of autism spectrum disorder. *Front Psychiatry* 2023; **14:** 1110525.

117. Clapcote SJ, Lipina TV, Millar JK, Mackie S, Christie S, Ogawa F *et al.* Behavioral phenotypes of Disc1 missense mutations in mice. *Neuron* 2007; **54**(3)**:** 387-402.

118. Critchlow HM, Maycox PR, Skepper JN, Krylova O. Clozapine and haloperidol differentially regulate dendritic spine formation and synaptogenesis in rat hippocampal neurons. *Mol Cell Neurosci* 2006; **32**(4)**:** 356-365.

119. de Bartolomeis AA, L. Ambesi, A. Bravi, D. Caraco, C. Cicale, M. Muscettola, G. Barone, P. Postsynaptic density protein gene expression after typical or atypical antipsychotics administration. 2000.

120. de Bartolomeis A, Ambesi A, Fiore G, Iasevoli F, Muscettola G. *Homer 1A induction: A novel signal transduction system differentially affected by typical and atypical antipsychotics? Implication for D2 receptor blockade*, 2004, 51-52pp.

121. de Bartolomeis A, Sarappa C, Buonaguro EF, Marmo F, Eramo A, Tomasetti C *et al.* Different effects of the NMDA receptor antagonists ketamine, MK-801, and memantine on postsynaptic density transcripts and their topography: role of Homer signaling, and implications for novel antipsychotic and pro-cognitive targets in psychosis. *Prog Neuropsychopharmacol Biol Psychiatry* 2013; **46:** 1-12.

122. de Bartolomeis A, Errico F, Aceto G, Tomasetti C, Usiello A, Iasevoli F. D-aspartate dysregulation in Ddo(-/-) mice modulates phencyclidine-induced gene expression changes of postsynaptic density molecules in cortex and striatum. *Prog Neuropsychopharmacol Biol Psychiatry* 2015; **62:** 35-43.

123. de Bartolomeis A, Avagliano C, Vellucci L, D'Ambrosio L, Manchia M, D'Urso G *et al.* Translating preclinical findings in clinically relevant new antipsychotic targets: focus on the glutamatergic postsynaptic density. Implications for treatment resistant schizophrenia. *Neurosci Biobehav Rev* 2019; **107:** 795-827.

124. de la Fuente Revenga M, Ibi D, Saunders JM, Cuddy T, Ijaz MK, Toneatti R *et al.* HDAC2-dependent Antipsychotic-like Effects of Chronic Treatment with the HDAC Inhibitor SAHA in Mice. *Neuroscience* 2018; **388:** 102-117.

125. Diana MC, Santoro ML, Xavier G, Santos CM, Spindola LN, Moretti PN *et al.* Low expression of Gria1 and Grin1 glutamate receptors in the nucleus accumbens of Spontaneously Hypertensive Rats (SHR). *Psychiatry Res* 2015; **229**(3)**:** 690-694.

126. Eastwood SL, Burnet PW, Harrison PJ. Striatal synaptophysin expression and haloperidol-induced synaptic plasticity. *Neuroreport* 1994; **5**(6)**:** 677-680.

127. Eastwood SL, Heffernan J, Harrison PJ. Chronic haloperidol treatment differentially affects the expression of synaptic and neuronal plasticity-associated genes. *Mol Psychiatry* 1997; **2**(4)**:** 322-329.

128. Eastwood SL, Burnet PW, Harrison PJ. Expression of complexin I and II mRNAs and their regulation by antipsychotic drugs in the rat forebrain. *Synapse* 2000; **36**(3)**:** 167-177.

129. Engel M, Snikeris P, Matosin N, Newell KA, Huang XF, Frank E. mGluR2/3 agonist LY379268 rescues NMDA and GABAA receptor level deficits induced in a two-hit mouse model of schizophrenia. *Psychopharmacology (Berl)* 2016; **233**(8)**:** 1349-1359.

130. Engmann O, Giralt A, Girault JA. Acute drug-induced spine changes in the nucleus accumbens are dependent on β-adducin. *Neuropharmacology* 2016; **110**(Pt A)**:** 333-342.

131. Ertuğrul A, Ozdemir H, Vural A, Dalkara T, Meltzer HY, Saka E. The influence of N-desmethylclozapine and clozapine on recognition memory and BDNF expression in hippocampus. *Brain Res Bull* 2011; **84**(2)**:** 144-150.

132. Fiore GC, M. Magara, S. Mondola, R. Muscettola, G. de Bartolomeis, A. Antipsychotics with different D2 dopamine receptor potency affect differently the postsynaptic density protein homer at glutamatergic metabotropic synapse. 2003.

133. Fosnaugh JS, Bhat RV, Yamagata K, Worley PF, Baraban JM. Activation of arc, a putative "effector" immediate early gene, by cocaine in rat brain. *J Neurochem* 1995; **64**(5)**:** 2377-2380.

134. Fry BR, Russell N, Gifford R, Robles CF, Manning CE, Sawa A *et al.* Assessing Reality Testing in Mice Through Dopamine-Dependent Associatively Evoked Processing of Absent Gustatory Stimuli. *Schizophr Bull* 2020; **46**(1)**:** 54-67.

135. Fu XZ, Zhang QG, Meng FJ, Zhang GY. NMDA receptor-mediated immediate Ser831 phosphorylation of GluR1 through CaMKIIalpha in rat hippocampus during early global ischemia. *Neurosci Res* 2004; **48**(1)**:** 85-91.

136. Fujimura M, Hashimoto K, Yamagami K. Effects of antipsychotic drugs on neurotoxicity, expression of fos-like protein and c-fos mRNA in the retrosplenial cortex after administration of dizocilpine. *Eur J Pharmacol* 2000; **398**(1)**:** 1-10.

137. Fukuyama K, Hasegawa T, Okada M. Cystine/Glutamate Antiporter and Aripiprazole Compensate NMDA Antagonist-Induced Dysfunction of Thalamocortical L-Glutamatergic Transmission. *Int J Mol Sci* 2018; **19**(11).

138. Gardoni F, Frasca A, Zianni E, Riva MA, Di Luca M, Fumagalli F. Repeated treatment with haloperidol, but not olanzapine, alters synaptic NMDA receptor composition in rat striatum. *Eur Neuropsychopharmacol* 2008; **18**(7)**:** 531-534.

139. Gentzel RC, Toolan D, Roberts R, Koser AJ, Kandebo M, Hershey J *et al.* The PDE10A inhibitor MP-10 and haloperidol produce distinct gene expression profiles in the striatum and influence cataleptic behavior in rodents. *Neuropharmacology* 2015; **99:** 256-263.

140. Gnegy ME, Agrawal A, Hewlett K, Yeung E, Yee S. Repeated haloperidol increases both calmodulin and a calmodulin-binding protein in rat striatum. *Brain Res Mol Brain Res* 1994; **27**(2)**:** 195-204.

141. Guo X, Hamilton PJ, Reish NJ, Sweatt JD, Miller CA, Rumbaugh G. Reduced expression of the NMDA receptor-interacting protein SynGAP causes behavioral abnormalities that model symptoms of Schizophrenia. *Neuropsychopharmacology* 2009; **34**(7)**:** 1659-1672.

142. Halff EF, Cotel MC, Natesan S, McQuade R, Ottley CJ, Srivastava DP *et al.* Effects of chronic exposure to haloperidol, olanzapine or lithium on SV2A and NLGN synaptic puncta in the rat frontal cortex. *Behav Brain Res* 2021; **405:** 113203.

143. Hamid EH, Hyde TM, Baca SM, Egan MF. Failure to down regulate NMDA receptors in the striatum and nucleus accumbens associated with neuroleptic-induced dyskinesia. *Brain Res* 1998; **796**(1-2)**:** 291-295.

144. Hara H, Oyagi A, Oida Y, Kakefuda K, Shimazawa M, Shioda N *et al.* P.3.b.004 Abnormal behaviours of conditional heparin-binding epidermal growth factor-like growth factor knockout mice. *European Neuropsychopharmacology - EUR NEUROPSYCHOPHARMACOL* 2009; **19**.

145. Hashimoto K, Tomitaka S, Narita N, Minabe Y, Iyo M. Induction of Fos protein by 3,4- methylenedioxymethamphetamine (Ecstasy) in rat brain: regional differences in pharmacological manipulation. *Addict Biol* 1997; **2**(3)**:** 317-326.

146. Healy DJK, R. E. Bovenkerk, K. A. Damask, S. P. MeadorWoodruff, J. H. Differential regulation of non-NMDA subunit mRNA by antipsychotics. 1996.

147. Hentschel K, Moore KE, Lookingland KJ. Effects of prolactin on expression of Fos-related antigens in tyrosine hydroxylase-immunoreactive neurons in subdivisions of the arcuate nucleus. *Brain Res* 2000; **857**(1-2)**:** 110-118.

148. Hussain N, Flumerfelt BA, Rajakumar N. Glutamatergic regulation of haloperidol-induced c-fos expression in the rat striatum and nucleus accumbens. *Neuroscience* 2001; **102**(2)**:** 391-399.

149. Hussain N, Flumerfelt BA, Rajakumar N. Muscarinic, adenosine A(2) and histamine H(3) receptor modulation of haloperidol-induced c-fos expression in the striatum and nucleus accumbens. *Neuroscience* 2002; **112**(2)**:** 427-438.

150. Iasevoli FF, G. Festa, G. Nappi, F. D'Amato, F. Muscettola, G. de Bartolomeis, A. Is antipsychotic-induced Homer 1a mRNA expression mediated by dopamine receptors? A potential link between antipsychotics, dopamine, and glutamate. 2005.

151. Iasevoli F, Tomasetti C, Bartolomeis A. *THE EXTENT OF STRIATAL DOPAMINE PERTURBATION BY ANTIPSYCHOTICS AFFECTS THE EXPRESSION OF THE GLUTAMATERGIC GENE HOMER1A: A QUANTITATIVE ANALYSIS OF MOLECULAR IMAGING STUDIES*, 2011, 107-107pp.

152. Iasevoli F, Sarappa C, Eramo A, Buonaguro E, Marmo F, Bartolomeis A. Poster #9 ANTAGONISTS AT N-METHYL-D-ASPARTATE RECEPTOR (NMDA-R) WITH DIFFERENT CLINICAL PROFILE AFFECT DIFFERENTLY THE TRANSCRIPTS OF CONSTITUTIVE AND INDUCIBLE EARLY GENES AT POSTSYNAPTIC DENSITY. *Schizophrenia Research* 2012; **136:** S188.

153. Iasevoli F, Tomasetti C, Buonaguro E, Latte G, Rossi R, Avvisati L *et al.* Poster #T100 MINOCYCLINE ADD-ON TO HALOPERIDOL BLUNTS HALOPERIDOL-MEDIATED EXPRESSION OF EARLY GENES IMPLICATED IN GLUTAMATERGIC NEUROTRANSMISSION IN BOTH VEHICLE AND KETAMINE-TREATED RATS. *Schizophrenia Research* 2014; **153:** S325.

154. Inada K, Farrington JS, Moy SS, Koller BH, Duncan GE. Assessment of NMDA receptor activation in vivo by Fos induction after challenge with the direct NMDA agonist (tetrazol-5-yl)glycine: effects of clozapine and haloperidol. *J Neural Transm (Vienna)* 2007; **114**(7)**:** 899-908.

155. Ito T, Yoshida M, Aida T, Kushima I, Hiramatsu Y, Ono M *et al.* Astrotactin 2 (ASTN2) regulates emotional and cognitive functions by affecting neuronal morphogenesis and monoaminergic systems. *J Neurochem* 2023; **165**(2)**:** 211-229.

156. Jevtić Dožudić G, Stojkovic T, Nikolic T, Velimirovic M, Petronijevic N. P.1.d.003 Alterations of oligodendroglial cells in the rat brain in phencyclidine animal model of schizophrenia: effects of antipsychotic treatment. *European Neuropsychopharmacology* 2015; **25:** S207-S208.

157. Johnson KM, Phillips M, Wang C, Kevetter GA. Chronic phencyclidine induces behavioral sensitization and apoptotic cell death in the olfactory and piriform cortex. *J Neurosci Res* 1998; **52**(6)**:** 709-722.

158. Kabbani N, Levenson R. Antipsychotic-induced alterations in D2 dopamine receptor interacting proteins within the cortex. *Neuroreport* 2006; **17**(3)**:** 299-301.

159. Kalman S, Pakaski M, Szucs S, Kalman J, Jr., Fazekas O, Santha P *et al.* [9-hydroxy-risperidone (9OHRIS) prevents stress-induced β-actin overexpression in rat hippocampus]. *Neuropsychopharmacol Hung* 2010; **12**(3)**:** 425-431.

160. Kalman Jr JK, S. Santha, P. Domokos, A. Fazekas, O. Szucs, Sz Pakaski, M. Szabo, Gy Janka, Z. Kalman, J. Effects of immobilization stress and sertindole on the transcription of Alzheimer's disease associated genes in rat brain. 2010.

161. Kessas MC, M. Nobrega, J. N. An examination of synaptic protein following chronic haloperidol in rat model of tardive dyskinesia. 2010.

162. Kim YHS, M. K. Cho, H. Y. Lee, C. H. Lee, J. G. Park, S. W. Effects of antipsychotic drugs on the expression of synapse-associated proteins in the frontal cortex of rats. 2014.

163. Klintzova AJ, Haselhorst U, Uranova NA, Schenk H, Istomin VV. The effects of haloperidol on synaptic plasticity in rat's medial prefrontal cortex. *J Hirnforsch* 1989; **30**(1)**:** 51-57.

164. Kontkanen O, Lakso M, Koponen E, Wong G, Castrén E. Molecular effects of the psychotropic NMDA receptor antagonist MK-801 in the rat entorhinal cortex: increases in AP-1 DNA binding activity and expression of Fos and Jun family members. *Ann N Y Acad Sci* 2000; **911:** 73-82.

165. Korostynski M, Piechota M, Dzbęk J, Mlynarski W, Szklarczyk K, Ziolkowska B *et al.* P.1.a.011 The prediction of pharmacological properties of psychotropic drugs based on transcriptional profiling in the brain. *European Neuropsychopharmacology* 2012; **22:** S159.

166. Kosugi SI, Y. Minabe, Y. Toda, S. Distinct subcellular distributions of GluR1 and GluR2 and their regulatory factors in the nucleus accumbens of repeatedly cocaine-administrated rats. 2010.

167. Kruyer A, Parrilla-Carrero J, Powell C, Brandt L, Gutwinski S, Angelis A *et al.* Accumbens D2-MSN hyperactivity drives antipsychotic-induced behavioral supersensitivity. *Mol Psychiatry* 2021; **26**(11)**:** 6159-6169.

168. Latte G, Iasevoli F, Buonaguro E, Tomasetti C, Marmo F, Avagliano C *et al.* Haloperidol modulates functional connectivity in the glutamate system: a postsynaptic density immediate-early gene-based network approach. *European Neuropsychopharmacology* 2016; **26:** S224-S225.

169. Lee J, Rajakumar N. Role of NR2B-containing N-methyl-D-aspartate receptors in haloperidol-induced c-Fos expression in the striatum and nucleus accumbens. *Neuroscience* 2003; **122**(3)**:** 739-745.

170. Lee J, Rushlow WJ, Rajakumar N. L-type calcium channel blockade on haloperidol-induced c-Fos expression in the striatum. *Neuroscience* 2007; **149**(3)**:** 602-616.

171. Lee MY, Lin BF, Chan MH, Chen HH. Increased behavioral and neuronal responses to a hallucinogenic drug after adolescent toluene exposure in mice: Effects of antipsychotic treatment. *Toxicology* 2020; **445:** 152602.

172. Leggieri A, García-González J, Torres-Perez JV, Havelange W, Hosseinian S, Mech AM *et al.* Ankk1 Loss of Function Disrupts Dopaminergic Pathways in Zebrafish. *Front Neurosci* 2022; **16:** 794653.

173. Lévesque C, Hernandez G, Mahmoudi S, Calon F, Gasparini F, Gomez-Mancilla B *et al.* Deficient striatal adaptation in aminergic and glutamatergic neurotransmission is associated with tardive dyskinesia in non-human primates exposed to antipsychotic drugs. *Neuroscience* 2017; **361:** 43-57.

174. Lidow MSK, P. O. Zhang, L. Castner, S. A. Allen, P. B. Greengard, P. Goldman-Rakic, P. S. Effects of chronic haloperidol treatment on microtubule-associated protein-2, spinophilin, and synaptophysin in the brain of young rhesus monkeys. 2003.

175. Lidsky TI, Yablonsky-Alter E, Zuck LG, Banerjee SP. Antipsychotic drug effects on glutamatergic activity. *Brain Res* 1997; **764**(1-2)**:** 46-52.

176. Liu X, Li J, Guo C, Wang H, Sun Y, Wang H *et al.* Olanzapine Reverses MK-801-Induced Cognitive Deficits and Region-Specific Alterations of NMDA Receptor Subunits. *Front Behav Neurosci* 2017; **11:** 260.

177. Loessner B, Bullock S, Rose SP. 411B: a monoclonal postsynaptic marker for modulations of synaptic connectivity in the rat brain. *J Neurochem* 1988; **51**(2)**:** 385-390.

178. MacDonald MG, M. Newman, J. Ding, Y. Fish, K. Lewis, D. Yates, N. Sweet, R. Synaptic protein alterations in schizophrenia are robust, highly organized, and suggest a link between altered glutamate and GABA receptor trafficking in dynamic spine loss. 2017.

179. Marchisella F, Paladini MS, Begni V, Brivio P, Sbrini G, Guidi A *et al.* P.1.13 Neuroplastic changes following chronic treatment with antipsychotic blonanserin in rats: Implications for schizophrenia. *European Neuropsychopharmacology* 2019; **29:** S642-S643.

180. Marin C, Tolosa E. Striatal synaptophysin levels are not indicative of dopaminergic supersensitivity. *Neuropharmacology* 1997; **36**(8)**:** 1115-1117.

181. Marmo FT, C. Iasevoli, F. Sarappa, C. Buonaguro, E. Giordano, S. Muscettola, G. De Bartolomeis, A. Homer and postsynaptic density: A putative target of antipsychotic treatment or a possible molecular marker of antipsychotic response? 2009.

182. Marmo FB, E. F. Latte, G. Iasevoli, F. De Bartolomeis, A. Immediate early and constitutive genes expression in antipsychotic switching paradigm: From clinical practice to animal model. 2014.

183. Martin MV, Mirnics K, Nisenbaum LK, Vawter MP. Olanzapine Reversed Brain Gene Expression Changes Induced by Phencyclidine Treatment in Non-Human Primates. *Mol Neuropsychiatry* 2015; **1**(2)**:** 82-93.

184. McCoy L, Cox C, Richfield EK. Chronic treatment with typical and atypical antipsychotics increases the AMPA-preferring form of AMPA receptor in rat brain. *Eur J Pharmacol* 1996; **318**(1)**:** 41-45.

185. McCoy L, Cox C, Richfield EK. Antipsychotic drug regulation of AMPA receptor affinity states and GluR1, GluR2 splice variant expression. *Synapse* 1998; **28**(3)**:** 195-207.

186. McKerchar CE, Morris BJ, Pratt J. *Acute and chronic PCP-induced chances in PSD95 mRNA expression: Modulation by clozapine and haloperidol*, 2002, A13-A13pp.

187. Corena-McLeod M, Walss-Bass C, Oliveros A, Gordillo Villegas A, Ceballos C, Charlesworth CM *et al.* New model of action for mood stabilizers: phosphoproteome from rat pre-frontal cortex synaptoneurosomal preparations. *PLoS One* 2013; **8**(5)**:** e52147.

188. Meltzer H. S187. Subchronic PCP Treatment in Mice Recapitulates Synaptic Proteomic Changes in Schizophrenia Cortex via Effects on CAMK2 and Microtubules and Rescue by Atypical Antipsychotic, Lurasidone. *Biological Psychiatry* 2019; **85**(10)**:** S369.

189. Meshul CK, Casey DE. Regional, reversible ultrastructural changes in rat brain with chronic neuroleptic treatment. *Brain Res* 1989; **489**(2)**:** 338-346.

190. Meshul CK, Stallbaumer RK, Taylor B, Janowsky A. Haloperidol-induced morphological changes in striatum are associated with glutamate synapses. *Brain Res* 1994; **648**(2)**:** 181-195.

191. Meshul CK, Tan SE. Haloperidol-induced morphological alterations are associated with changes in calcium/calmodulin kinase II activity and glutamate immunoreactivity. *Synapse* 1994; **18**(3)**:** 205-217.

192. Meshul CK, Stallbaumer RK, Allen C. GM1 ganglioside administration partially counteracts the morphological changes associated with haloperidol treatment within the dorsal striatum of the rat. *Psychopharmacology (Berl)* 1995; **121**(4)**:** 461-469.

193. Meshul CK, Andreassen OA, Allen C, Jørgensen HA. Correlation of vacuous chewing movements with morphological changes in rats following 1-year treatment with haloperidol. *Psychopharmacology (Berl)* 1996; **125**(3)**:** 238-247.

194. Mielnik CB, M. K. Islam, R. Milenkovic, M. Horsfall, W. Salahpour, A. Ramsey, A. Conditional rescue of NMDA receptor hypofunction to study the plasticity and circuitry of schizophrenia-relevant behaviours. 2015.

195. Mishra A, Singla R, Kumar R, Sharma A, Joshi R, Sarma P *et al.* Granulocyte Colony-Stimulating Factor Improved Core Symptoms of Autism Spectrum Disorder via Modulating Glutamatergic Receptors in the Prefrontal Cortex and Hippocampus of Rat Brains. *ACS Chem Neurosci* 2022; **13**(20)**:** 2942-2961.

196. Mohn AR, Gainetdinov RR, Caron MG, Koller BH. Mice with reduced NMDA receptor expression display behaviors related to schizophrenia. *Cell* 1999; **98**(4)**:** 427-436.

197. Nakahara T, Nakamura K, Tsutsumi T, Hashimoto K, Hondo H, Hisatomi S *et al.* Effect of chronic haloperidol treatment on synaptic protein mRNAs in the rat brain. *Brain Res Mol Brain Res* 1998; **61**(1-2)**:** 238-242.

198. Oda Y, Fujita Y, Oishi K, Nakata Y, Takase M, Niitsu T *et al.* Alterations in glutamatergic signaling in the brain of dopamine supersensitivity psychosis and non-supersensitivity psychosis model rats. *Psychopharmacology (Berl)* 2017; **234**(20)**:** 3027-3036.

199. O'Neill N, McLaughlin C, Komiyama N, Sylantyev S. Biphasic Modulation of NMDA Receptor Function by Metabotropic Glutamate Receptors. *J Neurosci* 2018; **38**(46)**:** 9840-9855.

200. Onimus O, Valjent E, Fisone G, Gangarossa G. Haloperidol-Induced Immediate Early Genes in Striatopallidal Neurons Requires the Converging Action of cAMP/PKA/DARPP-32 and mTOR Pathways. *Int J Mol Sci* 2022; **23**(19).

201. Onishi T, Sakamoto H, Namiki S, Hirose K. The Altered Supramolecular Structure of Dopamine D2 Receptors in Disc1-deficient Mice. *Sci Rep* 2018; **8**(1)**:** 1692.

202. Oretti RG, Spurlock G, Buckland PR, McGuffin P. Lack of effect of antipsychotic and antidepressant drugs on glutamate receptor mRNA levels in rat brains. *Neurosci Lett* 1994; **177**(1-2)**:** 39-43.

203. Ossowska K, Pietraszek M, Wardas J. Further evidence for the subsensitivity of striatal AMPA receptors, induced by chronic haloperidol administration: an autoradiographic study. *Naunyn Schmiedebergs Arch Pharmacol* 1996; **354**(3)**:** 384-388.

204. Ossowska K, Pietraszek M, Wardas J, Nowak G, Wolfarth S. Chronic haloperidol and clozapine administration increases the number of cortical NMDA receptors in rats. *Naunyn Schmiedebergs Arch Pharmacol* 1999; **359**(4)**:** 280-287.

205. Oyagi A, Oida Y, Kakefuda K, Shimazawa M, Shioda N, Moriguchi S *et al.* Generation and characterization of conditional heparin-binding EGF-like growth factor knockout mice. *PLoS One* 2009; **4**(10)**:** e7461.

206. Oyagi AO, Y. Kakefuda, K. Shimazawa, M. Shioda, N. Moriguchi, S. Kitaichi, K. Nanba, D. Furuta, Y. Fukunaga, K. Higashiyama, S. Hara, H. Focused Conference Group: P17 - Newapproaches and targets in psychiatry generation and characterization of conditional heparin-binding EGF-like growth factor knockout mice. 2010.

207. Ozdemir H, Ertugrul A, Basar K, Saka E. Differential effects of antipsychotics on hippocampal presynaptic protein expressions and recognition memory in a schizophrenia model in mice. *Prog Neuropsychopharmacol Biol Psychiatry* 2012; **39**(1)**:** 62-68.

208. Pan TY, Pan YJ, Tsai SJ, Tsai CW, Yang FY. Focused Ultrasound Stimulates the Prefrontal Cortex and Prevents MK-801-Induced Psychiatric Symptoms of Schizophrenia in Rats. *Schizophr Bull* 2024; **50**(1)**:** 120-131.

209. Park SW, Lee CH, Cho HY, Seo MK, Lee JG, Lee BJ *et al.* Effects of antipsychotic drugs on the expression of synaptic proteins and dendritic outgrowth in hippocampal neuronal cultures. *Synapse* 2013; **67**(5)**:** 224-234.

210. Pathak G, Ibrahim BA, McCarthy SA, Baker K, Kelly MP. Amphetamine sensitization in mice is sufficient to produce both manic- and depressive-related behaviors as well as changes in the functional connectivity of corticolimbic structures. *Neuropharmacology* 2015; **95:** 434-447.

211. Pei Q, Tordera R, Sprakes M, Sharp T. Glutamate receptor activation is involved in 5-HT2 agonist-induced Arc gene expression in the rat cortex. *Neuropharmacology* 2004; **46**(3)**:** 331-339.

212. Petronijevic N, Jevtić Dožudić G, Nikolic T, Mircic A, Stojkovic T, Velimirovic M. P.1.b.019 Effects of antipsychotic treatment on the proteins of glutamatergic synapse in the brain of rats perinatally treated with phencyclidine. *European Neuropsychopharmacology* 2015; **25:** S188-S189.

213. Picconi B, Gardoni F, Centonze D, Mauceri D, Cenci MA, Bernardi G *et al.* Abnormal Ca2+-calmodulin-dependent protein kinase II function mediates synaptic and motor deficits in experimental parkinsonism. *J Neurosci* 2004; **24**(23)**:** 5283-5291.

214. Pillai-Nair N, Panicker AK, Rodriguiz RM, Gilmore KL, Demyanenko GP, Huang JZ *et al.* Neural cell adhesion molecule-secreting transgenic mice display abnormalities in GABAergic interneurons and alterations in behavior. *J Neurosci* 2005; **25**(18)**:** 4659-4671.

215. Pisano I, Begni V, Creutzberg K, Marchisella F, Federico DR, Papp M *et al.* P.0552 The early therapeutic efficacy of lurasidone in a rodent model of depression: a behavioural and biomolecular study. *European Neuropsychopharmacology* 2021; **53:** S406.

216. Pizzagalli F, Martin-Facklam M, Hofmann C, Boetsch C, Ereshefsky L, Patat A *et al.* Poster #209 CENTRAL GLYCINE INCREASE IN RATS, MONKEYS AND HEALTHY VOLUNTEERS AFTER TWO GLYCINE REUPTAKE INHIBITORS, RG1678 AND RG7118. *Schizophrenia Research* 2012; **136:** S166.

217. Porteous D, Millar K. How DISC1 regulates postnatal brain development: girdin gets in on the AKT. *Neuron* 2009; **63**(6)**:** 711-713.

218. Purkayastha S, Ford J, Kanjilal B, Diallo S, Del Rosario Inigo J, Neuwirth L *et al.* Clozapine functions through the prefrontal cortex serotonin 1A receptor to heighten neuronal activity via calmodulin kinase II-NMDA receptor interactions. *J Neurochem* 2012; **120**(3)**:** 396-407.

219. Robinson S, Krentz L, Moore C, Meshul CK. Blockade of NMDA receptors by MK-801 reverses the changes in striatal glutamate immunolabeling in 6-OHDA-lesioned rats. *Synapse* 2001; **42**(1)**:** 54-61.

220. Rodriguez AL, Grier MD, Jones CK, Herman EJ, Kane AS, Smith RL *et al.* Discovery of novel allosteric modulators of metabotropic glutamate receptor subtype 5 reveals chemical and functional diversity and in vivo activity in rat behavioral models of anxiolytic and antipsychotic activity. *Mol Pharmacol* 2010; **78**(6)**:** 1105-1123.

221. Sanson A, Begni V, Luoni A, Zampar S, Longo L, Sensini F *et al.* P.471 Towards novel treatments for schizophrenia: molecular and behavioural signatures of putative antipsychotic drug SEP-363856. *European Neuropsychopharmacology* 2020; **40:** S267.

222. Santana N, Troyano-Rodriguez E, Mengod G, Celada P, Artigas F. Activation of thalamocortical networks by the N-methyl-D-aspartate receptor antagonist phencyclidine: reversal by clozapine. *Biol Psychiatry* 2011; **69**(10)**:** 918-927.

223. Scarr E, Dean B. Altered neuronal markers following treatment with mood stabilizer and antipsychotic drugs indicate an increased likelihood of neurotransmitter release. *Clin Psychopharmacol Neurosci* 2012; **10**(1)**:** 25-33.

224. Schalbetter SM, Mueller FS, Scarborough J, Richetto J, Weber-Stadlbauer U, Meyer U *et al.* Oral application of clozapine-N-oxide using the micropipette-guided drug administration (MDA) method in mouse DREADD systems. *Lab Anim (NY)* 2021; **50**(3)**:** 69-75.

225. Schmitt A, May B, Müller B, Zink M, Braus DF, Henn FA. [Effect of antipsychotics on glutaminergic neural transmission in the animal model]. *Nervenarzt* 2004; **75**(1)**:** 16-22.

226. Schmitt A, Gebicke-Haerter P, Sommer U, Heck M, Lex A, Herrera-Marschitz M *et al.* The Hypoxic Rat Model for Obstetric Complications in Schizophrenia. In: O'Donnell P (ed). *Animal Models of Schizophrenia and Related Disorders*. Humana Press: Totowa, NJ, 2011, pp 93-111.

227. Schroeder U, Schroeder H, Schwegler H, Sabel BA. Neuroleptics ameliorate phencyclidine-induced impairments of short-term memory. *Br J Pharmacol* 2000; **130**(1)**:** 33-40.

228. Sharp FR, Sagar SM. Alterations in gene expression as an index of neuronal injury: heat shock and the immediate early gene response. *Neurotoxicology* 1994; **15**(1)**:** 51-59.

229. Shen G, Han F, Shi WX. Effects of Low Doses of Ketamine on Pyramidal Neurons in Rat Prefrontal Cortex. *Neuroscience* 2018; **384:** 178-187.

230. Stan TLS, V. Zhang, X. Alvarsson, A. Ono, M. Svenningssson, P. Similarities in the biochemical modulation of DARPP-32, CREB, CamKII and AMPA receptors by lurasidone and fluoxetine. 2012.

231. Svane KC, Asis EK, Omelchenko A, Kunnath AJ, Brzustowicz LM, Silverstein SM *et al.* d-Serine administration affects nitric oxide synthase 1 adaptor protein and DISC1 expression in sex-specific manner. *Mol Cell Neurosci* 2018; **89:** 20-32.

232. Tarazi FI, Baldessarini RJ, Kula NS, Zhang K. Long-term effects of olanzapine, risperidone, and quetiapine on ionotropic glutamate receptor types: implications for antipsychotic drug treatment. *J Pharmacol Exp Ther* 2003; **306**(3)**:** 1145-1151.

233. Toua C, Brand L, Möller M, Emsley RA, Harvey BH. The effects of sub-chronic clozapine and haloperidol administration on isolation rearing induced changes in frontal cortical N-methyl-D-aspartate and D1 receptor binding in rats. *Neuroscience* 2010; **165**(2)**:** 492-499.

234. Uslaner JM, Parmentier-Batteur S, Flick RB, Surles NO, Lam JS, McNaughton CH *et al.* Dose-dependent effect of CDPPB, the mGluR5 positive allosteric modulator, on recognition memory is associated with GluR1 and CREB phosphorylation in the prefrontal cortex and hippocampus. *Neuropharmacology* 2009; **57**(5-6)**:** 531-538.

235. Vaisburd S, Shemer Z, Yeheskel A, Giladi E, Gozes I. Risperidone and NAP protect cognition and normalize gene expression in a schizophrenia mouse model. *Sci Rep* 2015; **5:** 16300.

236. Vellucci L, Iasevoli F, Filomena Buonaguro E, Latte G, Tomasetti C, Marmo F *et al.* *S205. A Translational Homer 1a-Based Network Approach: Imaging How Haloperidol Modulates Glutamate System Functional Connectivity*. Schizophr Bull. 2020 May;46(Suppl 1):S116-7. doi: 10.1093/schbul/sbaa031.271. Epub 2020 May 18.

237. Waters SK, D. Tedroff, J. Pontén, H. Sonesson, C. Gronier, B. Waters, N. Pridopidine - Effects on corticostriatal pathways controlling motor functions. 2011.

238. Yabuki Y, Nakagawasai O, Moriguchi S, Shioda N, Onogi H, Tan-No K *et al.* Decreased CaMKII and PKC activities in specific brain regions are associated with cognitive impairment in neonatal ventral hippocampus-lesioned rats. *Neuroscience* 2013; **234:** 103-115.

239. Yabuki Y, Wu L, Fukunaga K. Cognitive enhancer ST101 improves schizophrenia-like behaviors in neonatal ventral hippocampus-lesioned rats in association with improved CaMKII/PKC pathway. *J Pharmacol Sci* 2019; **140**(3)**:** 263-272.

240. Yanahashi S, Hashimoto K, Hattori K, Yuasa S, Iyo M. Role of NMDA receptor subtypes in the induction of catalepsy and increase in Fos protein expression after administration of haloperidol. *Brain Res* 2004; **1011**(1)**:** 84-93.

241. Yang SY, Hong CJ, Huang YH, Tsai SJ. The effects of glycine transporter I inhibitor, N-methylglycine (sarcosine), on ketamine-induced alterations in sensorimotor gating and regional brain c-Fos expression in rats. *Neurosci Lett* 2010; **469**(1)**:** 127-130.

242. Yasugawa S, Fukunaga K, Yamamoto H, Miyakawa T, Miyamoto E. Autophosphorylation of Ca2+/calmodulin-dependent protein kinase II: effects on interaction between enzyme and substrate. *Jpn J Pharmacol* 1991; **55**(2)**:** 263-274.

243. Zhang Q, Yu Y, Huang XF. Olanzapine Prevents the PCP-induced Reduction in the Neurite Outgrowth of Prefrontal Cortical Neurons via NRG1. *Sci Rep* 2016; **6:** 19581.

244. Zuo DY, Cao Y, Zhang L, Wang HF, Wu YL. Effects of acute and chronic administration of MK-801 on c-Fos protein expression in mice brain regions implicated in schizophrenia with or without clozapine. *Prog Neuropsychopharmacol Biol Psychiatry* 2009; **33**(2)**:** 290-295.

245. Zygmunt M, Hoinkis D, Piechota M, Rodriguez Parkitna J, Korostynski M. Gene expression signatures of psychotropic drugs in the prefrontal cortex and nucleus accumbens. *European Neuropsychopharmacology* 2016; **26:** S249.
